# Scenario analysis of supply‐ and demand‐side solutions for circular economy and climate change mitigation in the global building sector

**DOI:** 10.1111/jiec.13557

**Published:** 2024-10-08

**Authors:** Stefan Pauliuk, Fabio Carrer, Niko Heeren, Edgar G. Hertwich

**Affiliations:** ^1^ Faculty of Environment and Natural Resources University of Freiburg Freiburg Germany; ^2^ Industrial Ecology Program Norwegian University of Science and Technology Trondheim Norway

**Keywords:** circular economy, climate change mitigation, demand reduction, global, lightweight design, material efficiency, MFA, stock–flow analysis, wooden buildings

## Abstract

Residential and non‐residential buildings are a major contributor to human well‐being. At the same time, buildings cause 30% of final energy use, 18% of greenhouse gas emissions (GHGE), and about 65% of material accumulation globally. With electrification and higher energy efficiency of buildings, material‐related emissions gain relevance. The circular economy (CE) strategies, *narrow, slow, and close*, together with wooden buildings, can reduce material‐related emissions. We provide a comprehensive set of building stock transformation scenarios for 10 world regions until 2060, using the resource efficiency climate change model of the stock–flow–service nexus and including the full CE spectrum plus wood‐intensive buildings. The 2020–2050 global cumulative new construction ranges from 150 to 280 billion m^2^ for residential and 70‐120 billion m^2^ for non‐residential buildings. Ambitious CE reduces cumulative 2020–2050 primary material demand from 80 to 30 gigatons (Gt) for cement and from 35 to 15 Gt for steel. Lowering floor space demand by 1 m^2^ per capita leads to global savings of 800‐2500 megatons (Mt) of cement, 300‐1000 Mt of steel, and 3‐10 Gt CO_2_‐eq, depending on industry decarbonization and CE roll‐out. Each additional Mt of structural timber leads to savings of 0.4‐0.55 Mt of cement, 0.6‐0.85 Mt of steel, and 0.8‐1.8 Mt CO_2_‐eq of system‐wide GHGE. CE reduces 2020–2050 cumulative GHGE by up to 44%, where the highest contribution comes from the *narrow* CE strategies, that is, lower floorspace and lightweight buildings. Very low carbon emission trajectories are possible only when combining supply‐ and demand‐side strategies. This article met the requirements for a gold‐gold *JIE* data openness badge described at http://jie.click/badges.

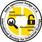

## 1 INTRODUCTION

Buildings are associated with 30% of global final energy consumption (IEA, 2023), 18% of greenhouse gas emissions (GHGE) (Ritchie, 2020), and about 65% of stock‐accumulating material consumption (Wiedenhofer et al., 2024). The building sector, residential and non‐residential, links human well‐being to environmental impacts in an energy and material service cascade (Kalt et al., 2019; Tanikawa et al., 2021), which provides multiple options for decoupling.

Material consumption is coupled to economic development (Pothen & Welsch, 2019), and the building sector, via population and floorspace growth, is a main contributor (Cheng et al., 2023; Fu et al., 2024; Soonsawad et al., 2024). Globally, we see an increase in demand for floorspace, driven by unmet needs (Rao et al., 2019) and economic prosperity. Demand for cooling and other building services grows, especially in emerging economies in the Global South (Mastrucci et al., 2019; Santamouris & Vasilakopoulou, 2021). The IEA (2019) and Wang et al. (2018) emphasize the role of the building sector in reaching global climate targets. Transforming how we design, construct, use, demolish, and recycle buildings is therefore a major component of sustainable development, and climate change mitigation and resource savings, in particular.

In the building sector, energy efficiency is a core decoupling strategy. Harvey (2014) projects future heating, cooling, and hot water demand and shows that with existing efficiency options, global building energy demand could be reduced by a factor of four. With gradually higher energy efficiency and electrification, the material‐related emissions of buildings gain relevance (Cabeza et al., 2013; Röck et al., 2020). Ren et al. (2022) demonstrate the magnitude of sand and gravel demand for construction and the lack of recycling options, and stress the need for material efficiency at the large scale (Cao et al., 2020).

There is a trade‐off in environmental pressure between material use in the production of buildings and energy use in their operation, requiring a careful systems analysis of scope 1, 2, and 3 elementary flows (Hertwich et al., 2019) in the stock–flow service nexus (Haberl et al., 2017) for buildings.

### 1.1 Literature review and state‐of‐the‐art

Studies highlight the role of electrification as a critical approach in the building sector's low carbon transition (Berrill et al., 2022; Wang et al., 2018), for example, via heat pumps (Lubjuhn & Venghaus, 2024). Building types matter for material use and embodied emissions, for example, moving away from single‐family housing to low‐rise multi‐unit residential buildings (Rankin et al., 2024). Resource, energy, and GHGE savings of CE strategies are documented at the building and material levels, including embodied energy savings for recycling (Gruhler & Schiller, 2023), savings of aggregates by concrete recycling (Mostert et al., 2021), more intensive use and lifetime extension of buildings (Hertwich et al., 2019), and re‐use of construction components and materials (Gallego‐Schmid et al., 2020; Lima et al., 2024; Mostert et al., 2021). Studies of the link between circular economy (CE) and GHGE mitigation strategies have focused on valuable but selective case studies (Cantzler et al., 2020). They provide quantitative potentials of the different CE options that can be scaled up in macro‐level and time‐explicit scenario analyses.

Traditionally, energy and scope 1 + 2 GHGE are the focus of high‐resolution scenario work, mostly by integrated assessment models. Mastrucci et al. (2021) report global residential building floorspace, energy, and GHGE scenarios for socioeconomic pathways (SSP) 1−3, focusing on heating and cooling demand in different climate zones and a detailed depiction of renovation. Given the urgency of climate change mitigation, the delay in mitigating GHGE from steel and cement production (Davis et al., 2018), the difficulties of meeting projected steel and cement demand within the global carbon budget (Watari, Cabrera Serrenho et al., 2023), and the environmental trade‐offs of large‐scale wood use (Johnston & Radeloff, 2019; O'Brien & Bringezu, 2017), there is a need to study supply‐ and demand‐side solutions (Creutzig et al., 2018) in combination (Xu et al., 2024). Berrill and Hertwich (2021) show for the United States that reduced floor area and more multi‐family homes lead to lower material requirements and emissions from construction. Mastrucci et al. (2023) stress the need to study low demand futures to formulate feasible transformation pathways with fewer trade‐offs on the environmental side.

On the material side, Krausmann et al. (2020) develop GDP‐ and population‐driven scenarios for global material stocks and find that providing essential services with a considerably lower level of material stocks could contribute to large reductions in global resource demand and GHGE. Zhang et al. (2022) study material and construction‐related GHGE for China and find that with reasonable floorspace, longer building life, and retrofitting, substantial reductions in material demand are possible. Mastrucci et al. (2024) confirm this finding. Zhong et al. (2021) study the material implications of the global building stock and report baseline (SSP2) material‐related GHGE between 3.6 and 4.8 Gt CO_2_‐eq/yr for 2020−2060, with a 2020−2060 cumulative GHGE reduction potential of lower floorspace of 55 Gt, 14 Gt for light‐weight design, and about 6 Gt CO_2_‐eq for lifetime extension and better material recovery, respectively. Pauliuk, Heeren et al. (2021) report a detailed scenario analysis for residential buildings and passenger vehicles combined but do not report much CE‐related details for buildings and do not cover non‐residential buildings. Olsson et al. (2023) conclude that “a combination of manufacturing and engineering decisions have the potential to reduce over 76% of the GHGE from cement and concrete production, equivalent to 3.6 Gt CO_2_‐eq lower emissions in 2100.” Mastrucci and van Ruijven (2023) study the low demand implications for the global residential building stock, including a post‐growth scenario centered around local communities and behavioral change.

Next to the *narrow, slow, and close* CE strategies (Bocken et al., 2016), large‐scale wooden construction is considered a key transformation strategy (Churkina et al., 2020), as buildings can be a carbon sink and the overall climate impact of the forest‐building system can be negative where forests continue to sequester carbon (Watari et al., 2024). Mishra et al. (2022) estimate that housing 90% of the new urban population globally in wood‐based urban mid‐rise buildings could save 106 Gt CO_2_ by 2100, if supplied by up to 150 Mha of new forest plantations.

### 1.2 Goal and scope of this work

A detailed and comprehensive building stock study that includes the full spectrum of the CE, *narrow, slow, and close*, as well as wood‐intensive buildings, is still lacking. Here, we address the following research questions (RQ), the first three of which cover building area stocks and flows, building material cycles, and energy and GHGE, while the latter two address decoupling and elasticities in the stock–flow–service nexus.

1. How large is the lock‐in from the existing building stock and what construction and demolition flows follow for the calibrated building lifetimes under different demand scenarios?
2. What is the impact of the major CE strategies—*narrow, slow, and close*—and wood‐intensive buildings on the consumption, recycling, and primary production of the major building materials cement, steel, and wood?
3. What are the energy and greenhouse gas implications of the different transformation strategies under different energy system and material production decarbonization scenarios?
4. What stages in the energy service cascade of buildings contribute most to decoupling benefits from impacts, and how do scope 1 + 2 GHGE compare to material‐related scope 3 GHGE for different regions and scenarios?
5. How large are the elasticities of the production of steel and cement as well as system‐wide GHGE under a changing degree of implementation of the most relevant transformation strategies identified?

The scope of the analysis is the global residential and non‐residential building sector, broken down into 10 large economies and world regions for the period 2016−2060.

## 2 METHOD AND DATA

**FIGURE 1:**
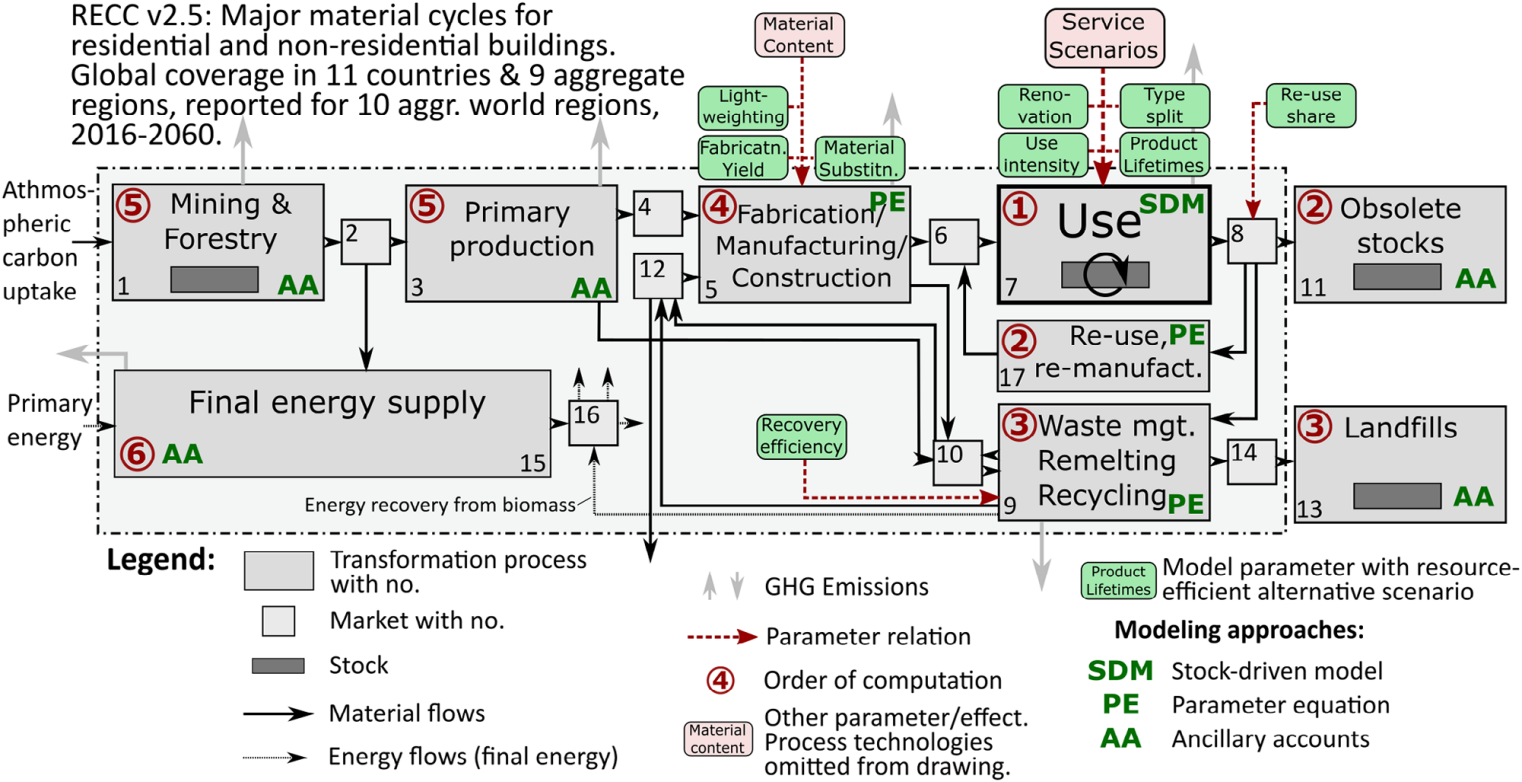
System definition of the resource efficiency climate change mitigation v2.5 model for global buildings. The model covers the use phase of residential and non‐residential buildings (scope 1); their energy supply for the three services heating, cooling, and hot water (scope 2); and the material production, recycling, and manufacturing activities associated with buildings (scope 3). The current model documentation can be found in Pauliuk (2023).

The research questions require the use of a detailed model of the stock–flow–service nexus (Haberl et al., 2017) for buildings in different world regions. A dynamic stock model of building area needs to be coupled to a material cycle model (Bergsdal et al., 2007) and to cradle‐to‐gate assessments of energy and material supply (Stephan et al., 2022). Here, the existing resource efficiency climate change (RECC) mitigation model framework (Pauliuk, Fishman et al., 2021) was chosen and expanded in coverage to include non‐residential buildings in six categories (office, commercial, health, education, hotels and restaurants, and other) along with residential buildings in four categories (single‐family houses [SFH], multi‐family houses [MFH], residential towers [RT], and informal buildings). The system definition of the RECC v2.5 model (Figure 1) shows the processes in the material cycles for steel, cement, wood, aluminum, copper, plastics, zinc, and bricks in the global building stock.

The world's countries are looked at in two single countries and eight aggregate regions, derived from the shared SSP framework (Riahi et al., 2017): China, India, Other Asia, Sub‐Saharan Africa (SSA), Latin America (LAM), Middle East and Northern Africa (MENA), the reforming economies in Europe and Asia (REF), other OEDC, the United States and Canada, the EU, and the United Kingdom.

### 2.1 RECC model drivers and parameters

The RECC model is driven by exogenous SSP‐compatible population and service demand (in m^2^/capita) scenarios (O'Neill et al., 2017), which were scaled down to the model regions (Fishman et al., 2021) (Table 1). Energy system futures (PBL, 2024) and assumptions on building type and energy carrier split are derived from major scenario exercises (IEA, 2022). Innovations in material production, including those based on hydrogen, are considered and scaled up. Energy–material consistent building archetypes address different energy standards, light‐weighting options, and wood use (Krych et al., 2024). Details can be found later, in the RECC model parameter database (link below), and the model documentation (Pauliuk, 2023).

**TABLE 1:** Central parameters for the stock–flow–service nexus of the use phase: Initial and future service level (per capita floorspace) for the different socioeconomic scenarios, and the typical building lifetime.

	**2015 per capita stock (m^2^)**		**2050 per capita stock, m^2^, LEMD/SSP1/SSP2**		**Typical building lifetime (years)**	
**Regions**	**Residential**	**Non‐residential**	**Residential**	**Non‐residential**	**Residential**	**Non‐residential**
SSA	11.4	0.8	19.4/26.9/33.4	7/10/12	50	45
LAM	34.4	3.0	30.3/34.4/44.3	7/10/12	50	45
EU_UK	37.7	12.5	31.2/40.1/46.2	12.8/16.1/20	100–180	60–80
China	36.1	10.8	31/40/50	13/16/20	27–40	30
India	11.7	0.8	25/28.7/38.1	7/10/12	50	45
Other_Asia	20.8	2.6	29.4/34.3/39	7.5/10.5/12.6	50	45
MNF	24.6	8.3	29.6/38.9/43.6	9/12/15	100	45
REF	23.5	5.9	29.5/38.9/43.5	9/12/15	120	60
Other_OEDC	38.0	6.5	30.5/39.9/44.5	9/12/15	100	50
USA_CAN	66.8	24.1	42.5/66.8/83.7	18/26/30	110	45

*Note*: Building lifetime can vary across age cohorts and here, typical values are indicated. Region and scenario acronyms are defined in the text.

### 2.2 Scenario assumptions and circular economy strategies

We study a multitude of possible parameter changes in a stock–flow consistent fashion. The main model driver, total floorspace demand by building type, is captured by the SSP1 and SSP2 floorspace target values (Fishman et al. (2021) (Table 1), supplemented by demand values adapted from the low energy and material demand (LEMD, *narrow* strategy) scenario by Grubler et al. (2018). For the energy carrier mix, energy supply GHGE intensity, and the share of low carbon material production technologies, two climate scenarios are depicted: “No new climate policy” (NoClimPol) and the RCP2.6 low carbon future (see supplementary material, section 1.4. for details). The future building energy carrier split directly reflects scenario results from the IEA's World Energy Outlook (2022) (Supplementary Table S4). Future shares of different electricity generation technologies were extracted from IMAGE model results (PBL, 2024) (Supplementary Table S6), which feature an almost complete phase‐out of fossil technologies with a limited amount of carbon capture and storage for RCP2.6. For calculating environmental pressure of total electricity demand, the scenario results for electricity demand from the different processes are summed up, divided into the contributions of individual generation technologies, and multiplied with technology‐specific pressure factors, which are mostly extracted from ecoinvent. For all other energy carriers, static pressure factors are used. Hydrogen‐based steel production and energy‐efficient aluminum production are assumed to be rolled out by 2050 in RCP2.6 (Supplementary Table S5). The environmental pressure of material production is determined from modified ecoinvent 3.8 product systems, where energy supply for material production was removed, adjusted to the scenario‐specific technology mix, and re‐routed to the system‐wide energy supply module.

Regarding materials, the extent of CE (*narrow* via light‐weighting, *slow, and close* strategies) and wood use is specified individually for each scenario run (Table 2), which allows us to study the impacts of the different CE strategies separately, in bundles, and for different demand (SSP dimension; SSP: shared socioeconomic pathway) and supply (RCP dimension; RCP: representative concentration pathway) scenarios. If a certain material‐related strategy is chosen, it is assumed to be ramped up to its currently identified technical potential (Table 2) by 2035.

**TABLE 2:** Potentials of circular economy (CE) strategies, their combination into the three CE aspects *narrow*, *slow*, *and close*, and definition of sub‐scenarios, which are then combined with the different socioeconomic pathway and RCP options.

Sub‐scenario	CE aspect	CE strategies	Potentials, including main references and parameter names link to files with further details
**_Base**		Current design, manufacturing, lifetime, and recycling practices	The base applies to the three different socioeconomic scenarios LEMD, SSP1, and SSP2, as well as to the two climate policy scenarios NoClimPol/Fossil and the RCP2.6 reference. Examples: SSP1_Base; SSP2_FullCE; LEMD_FullCE_HW (Fishman et al., 2021).
	**Narrow (demand‐side)**	More intense use (MIU), implemented separately from this ladder by lower per capita floorspaces in the low energy and material demand (LEMD) scenario	Future living and non‐residential building space is the central model driver, for which SSP‐compatible scenarios were built. See Table 1 and Table S3, 2_S_RECC_FinalProducts_Future_resbuildings_V2.7, and 2_S_RECC_FinalProducts_Future_NonResBuildings_V2.4 for details (Fishman et al., 2021).
**+ _Light**	**Narrow**	Using less material by design (ULD, light weighting). Implementing ULD only and not MIU leads to the _LIGHT scenarios, used for the sensitivity analysis	Building engineering model yields 15%–35% lower mass for lighter building design, still concrete‐based, wood use is considered separately below (MSU), see 3_MC_BuildingArchetypes_V2.1_LowWood; 3_MC_NonResBuildingArchetypes_V2.1_LowWood. Reduction of clinker content of cement of up to 15.6 %, see 3_SHA_CementContentReduction_V1.0 (Krych et al., 2024).
**+ _Slow**	**Slow**	Lifetime extension (LTE)	The building lifetime is a central parameter in the stock–flow–service nexus. The lifetime of the efficient and zero energy buildings is extended by up to 90% of the base value. See Table 1. See 6_PR_LifeTimeExtension_resbuildings_V2.3 and 6_PR_LifeTimeExtension_nonresbuildings_V1.1.
	**Slow**	Fabrication yield improvements (FYI)	5% yield loss during construction for steel, 3% (down to 1.5%) for concrete, and 10% for timber. See 4_PY_Manufacturing_V2.4 and 6_PR_FabricationYieldImprovement_V2.1 (Milford et al., 2011, 2013).
	**Slow**	Fabrication scrap diversion (FSD)	Fabrication scrap diversion to other uses of material without destructive recycling is implemented but currently has no quantified potential for building materials. See 6_PR_FabricationScrapDiversion_V1.2.
**+ _Close**	**Close**	Recovery rate improvements (EoL)	Steel from currently 85% to 95%. Concrete currently 0% to up to 25% for recycling into aggregates. See (Liu et al., 2013; Pauliuk et al., 2013), 4_PY_EoL_RecoveryRate_V2.6 and 6_PR_EoL_RR_Improvement_V2.4. Share of construction wood that is cascaded into material application for another 30 year increases from 15% to 30% for some and from 0% t0 15% for the remaining countries. See 6_PR_WoodCascading_Improvement.
	**Close**	Re‐use (ReU)	Up to 14% of wood and wood products, 27% of concrete elements, and 29% of steel components can be re‐used. See 6_PR_ReUse_Bld_V3.5 and 6_PR_ReUse_nonresBld_V1.4 (Mantau, 2015; Milford et al., 2013; Shanks et al., 2019).
**= _FullCE**		*Narrow, slow, and close* combined; MIU is represented separately from this ladder by lower per capita floorspace in the LEMD scenario	RECC V2.5 ramps up the different CE strategies by fully implementing their potential by 2035. Change in per capita floorspace as part of the *narrow* strategy (more intense use [MIU]) is implemented by lower per capita floorspaces in the low energy and material demand (LEMD) scenario.
**+ _Wood**	**HighWood**	Material substitution (MSU): More structural timber, less cement and steel. Using only MSU and no CE strategies leads to the _Wood or _HW scenarios	Building engineering model yields 65%–78% lower mass for wood‐intensive building design, see (Krych et al., 2024), and 3_MC_BuildingArchetypes_V2.1_LowWood; 3_MC_NonResBuildingArchetypes_V2.1_LowWood.
**= _FullCE** **_Wood**		All CE strategies rolled out, plus wood‐intensive new buildings	

*Note*: Each parameter file contains a list of references, more than a dozen for some, which were consulted to extract data for the different scenario aspects and circular economy strategies. The core references are listed here, and the parameter files are archived on Zenodo: https://zenodo.org/records/12752350

Along the three scenario dimensions, we analyze seven main scenarios: Each _Base and _FullCE sub‐scenario applies to the three different socioeconomic scenarios LEMD, SSP1, and SSP2. These are run with an RCP2.6‐compatible energy supply and material production, leading to six main scenarios, and LEMD_FullCE_Wood is considered as well. The sensitivity analyses cover another 15 scenarios, focusing on individual CE strategies as well as NoClimPol/fossil‐based energy supply and material production. Supplementary Table S7 contains a detailed overview of the different scenario narratives and the parameter values chosen.

RECC v2.5 contains a stylistic representation of forests (process 1 in Figure 1) as biogenic carbon pools of constant size, so that each wood harvest (*F*_1_2_) is compensated for by sequestration of atmospheric CO_2_ (*F*_0_1_) due to regrowth at the landscape level. All use of harvested wood thus has a negative climate impact in the year of harvest, and all release of biogenic C back to the atmosphere (after use and combustion) contributes to system‐wide GHGE in the year of combustion. Under this assumption, fuel wood is climate neutral, and construction wood delays biogenic emissions by the building and subsequent cascading lifetime, leading to the accumulation of biogenic C in buildings and net negative emissions of the combined system of forests and buildings. System‐wide GHGE are reported and plotted both with and without the assumed forest carbon sequestration, so that other assumptions and forest growth models can be applied easily.

### 2.3 Analysis of results

RQs 1−3 are addressed by showing results in commonly used stacked bar and area plots. The energy service cascade (Kalt et al., 2019) embeds the stock–flow–service nexus in a larger framework of linking well‐being to environmental impacts. To address RQ 4, we focus on the link between building services and GHGE (GWP100, including CO_2_, CH_4_, and N_2_O) in the cascade and study to IPAT‐style decomposition equations (IPAT: impact, population, affluence, technology), similar to and inspired by other frameworks in the literature (Carmona et al., 2022; Tanikawa et al., 2021), one for energy‐related GHGE per capita (Equation 1, scope 1 + 2) and one for material‐related GHGE (Equation 2, scope 3):

(1) $$\frac{\mathrm{Energy}\_\mathrm{GHG}}{\mathrm{capita}}=\frac{\mathrm{Energy}\_\mathrm{GHG}}{\mathrm{Final}\_\mathrm{Energy}}\times \frac{\mathrm{Final}\_\mathrm{Energy}}{\mathrm{Stock}}\times \frac{\mathrm{Stock}}{\mathrm{capita}}$$

(2) $$\frac{\mathrm{Material}\_\mathrm{GHG}}{\mathrm{capita}}=\frac{\mathrm{Material}\_\mathrm{GHG}}{\mathrm{Material}\_\mathrm{Cons}}\times \frac{\mathrm{Material}\_\mathrm{Cons}}{\mathrm{Inflow}}\times \frac{\mathrm{Inflow}}{\mathrm{Stock}}\times \frac{\mathrm{Stock}}{\mathrm{capita}}$$

To address research question 5, we run the model for high and low per capita floorspace and for high and low wood intensity, and derive the following elasticities (Equations 3 and 4):

(3) $$\begin{array}{ccc} & & \text{Material or GHGE saved per}\;m^{2}/\text{capita less}=(2020\text{-2050 cumulative material or GHGE},\\ & & \;\mathrm{SSP}2-2020\text{-2050 cumulative material or GHGE, LEMD})/(\left(2050m^{2},\;\text{SSP2 - 2050}\;m^{2},\mathrm{LEMD})*2050\;\text{population}\right) \end{array}$$

(4) $$\begin{array}{ccc} & & \text{Material or GHGE saved per Mt of additional wood used}=(2020\text{-2050}\;\text{cumulative material or GHGE, wood-intensive buildings}\\ & & \;-\;2020\text{-2050}\;\text{cumulative material or GHGE, concrete-intensive buildings})/(2020\text{-2050}\;\text{cumulative wood use, wood-intensive buildings}\\ & & \;-\;2020\text{-2050}\;\text{cumulative wood use, concrete-intensive buildings}) \end{array}$$

Table S7 in the supplement lists the exact scenario specification that was used to generate each of the result plots.

## 3 RESULTS

Presentation of results focusses on the global aggregate numbers with a few regional breakdowns. The figure supplement contains region‐specific versions of the plots presented here and the data supplement contains a full regional breakdown of all model results.

**FIGURE 2:**
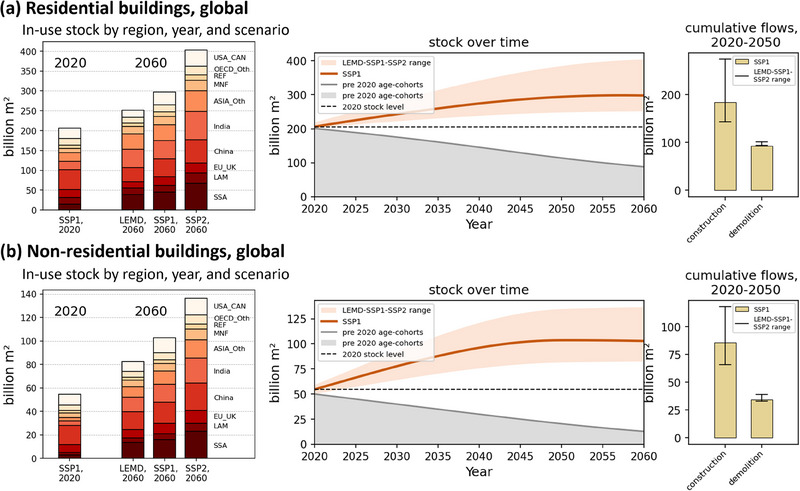
Addressing research question 1: Global total building stock (*S*_7_) by scenario, year, and region (left); time series of the in‐use stock (*S*_7_) with lock‐in from pre‐2020 age cohorts and range of future stock levels across scenarios (middle), and the range of the 2020–2050 cumulative new construction (*F*_6_7_) and demolition (*F*_7_8_) across scenarios (right), for residential buildings (a) and non‐residential buildings (b). The underlying data for this figure are available in Supporting Information S2.

The global building stock grows further in all scenarios, with largest increases in SSA, India, and Other Asia (Figure 2a). While the SSP2 scenario almost sees a doubling of the total stock, the LEMD increase is rather modest and even sees a decline in developed economies. The growth of non‐residential buildings is stronger in general, highlighting the growing global demand for the various service categories. Due to the long building lifetime, there is a substantial lock‐in (gray area in the center plots in Figure 2), with more than half of the 2020 residential buildings still standing in 2050 (more than 1/3 for non‐residential buildings), which underpins the need for deep building retrofits to reduce energy demand. Still, with current building lifetimes, globally, around 60% (residential) and 75% (non‐residential) buildings that stood in 2020 will be re‐built by 2060 according to our lifetime assumptions.

The 2020–2050 global cumulative new construction of residential buildings ranges between 150 and 280 billion m^2^ (70–120 billion m^2^ for non‐residential buildings), with total demolition volumes of around 100 billion m^2^ (residential) and around 35 billion m^2^ (non‐residential) during the same time range. The large gap between new construction and demolition highlights a major “circularity” gap in the global building stock: due to the large stock expansion (stock growth beyond the 2020 dashed line in the center of Figure 2), there is a large demand for construction materials that cannot be sourced from recovered material from demolished buildings. The total construction volume during this 30 year time period is roughly as large as the current stock of buildings.

**FIGURE 3:**
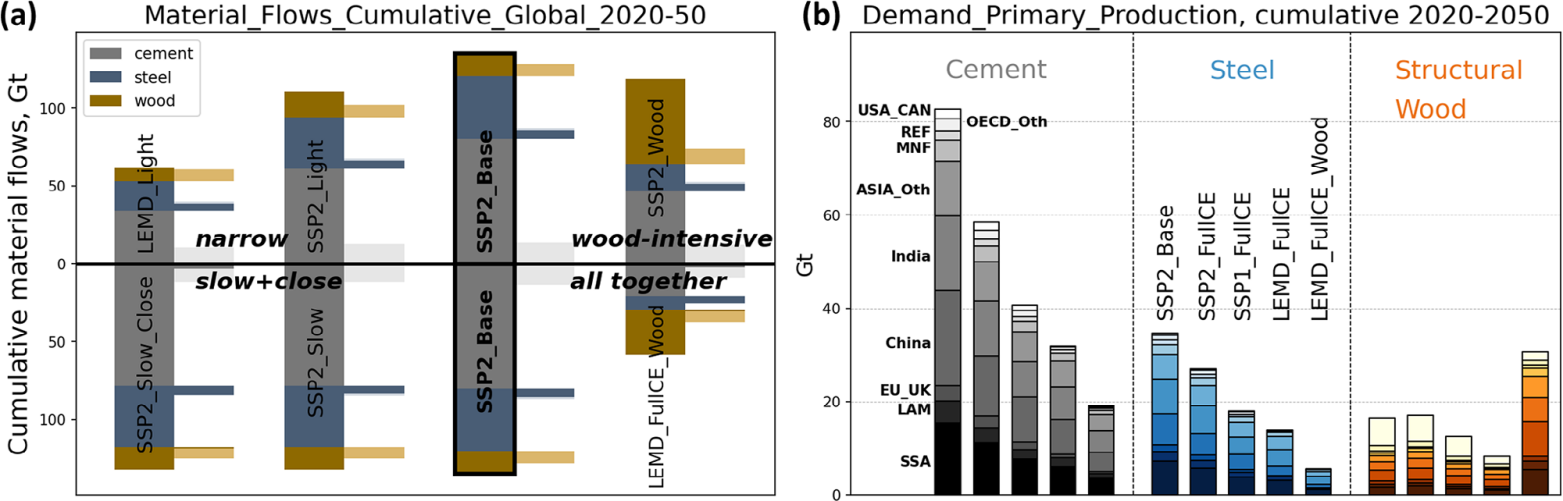
Addressing research question 2: Cumulative material flows. (a) Continuous bars: Cumulative material consumption in new buildings (*F*_6_7_), by material and circular economy strategy. Interrupted bars: cumulative material outflow from demolished buildings (*F*_7_8_, light colors, proxy for construction waste) and cumulative flows that are actually recycled (*F*_17_6_ *+ F*_9_12_, darker colors). This plot is a sensitivity analysis for different CE strategies in the four dimensions: *narrow*, *slow + close*, *wood intensive*, and *all together*. The SSP2_Base scenario is mirrored on both sides of the *x*‐axis. (b) Resulting demand for primary production by region (*F*_4_5_) and material for the SSP2_Base and the different full CE scenarios. Due to sawmill trimmings, the demand for industrial roundwood is roughly twice as high as the flow of structural wood and cannot be met by forest supply in most world regions (see Section 4). The underlying data for this figure are available in Supporting Information S2.

Cumulative 2020–2050 material demand for cement, steel, and wood amounts to about 130 Gt for the SSP2 scenario, and substantial lowering can be achieved by lightweight building design and substantially lower floorspace (Figure 3a, top left), a substantial shift to wooden buildings (top right), and a combination of all strategies (bottom right), with a total reduction potential of major material demand of 60%. This figure becomes much larger when concrete aggregates are included. The *slow and close* strategies (bottom left) show barely an effect, because the lifetime extension potential of existing buildings shows a limited effect due to the large trade‐off against more energy‐efficient new buildings and because the additional recycling potential for the major materials is limited. Due to massive stock expansion in emerging economies and long building lifetimes (large lock‐in) in developed economies, the available material outflow from construction (light colors) is small compared to the inflow demand in most world regions. The recycling and re‐use potential is almost entirely seized for steel and rather limited for cement (concrete re‐use only) and for wood (component re‐use and cascading). Recycling concrete by crushing is an established strategy to reduce demand for sand and gravel, but at least the same amount of cement is still needed as hydraulic binder (Kleijer et al., 2017; Knoeri et al., 2013; Mostert et al., 2021). Therefore, it has no climate benefit. For the period of analysis, a circular economy for major construction materials in the global building stock remains an illusion. This is due to the ongoing massive expansion of stocks in most world regions and the lack of recycling and re‐use opportunities for concrete and structural timber.

Still, primary resource demand, and subsequent GHGE, resource use, and land use impacts can be reduced a lot by light‐weighting, lower floorspace, and wood use strategies, in particular (Figure 3b). In a lower floorspace world with light‐weighted, long‐lived, and wood‐intensive buildings (LEMD_FullCE_Wood), cumulative cement demand could be less than 25% of the baseline figures, saving about 60 Gt of cement and 350 billion tons of aggregate extraction during 30 years. The reduction in primary steel demand is even more drastic, while cumulative structural wood demand roughly doubles, raising the question of sustainable supply of such large wood quantities in different world regions.

**FIGURE 4:**
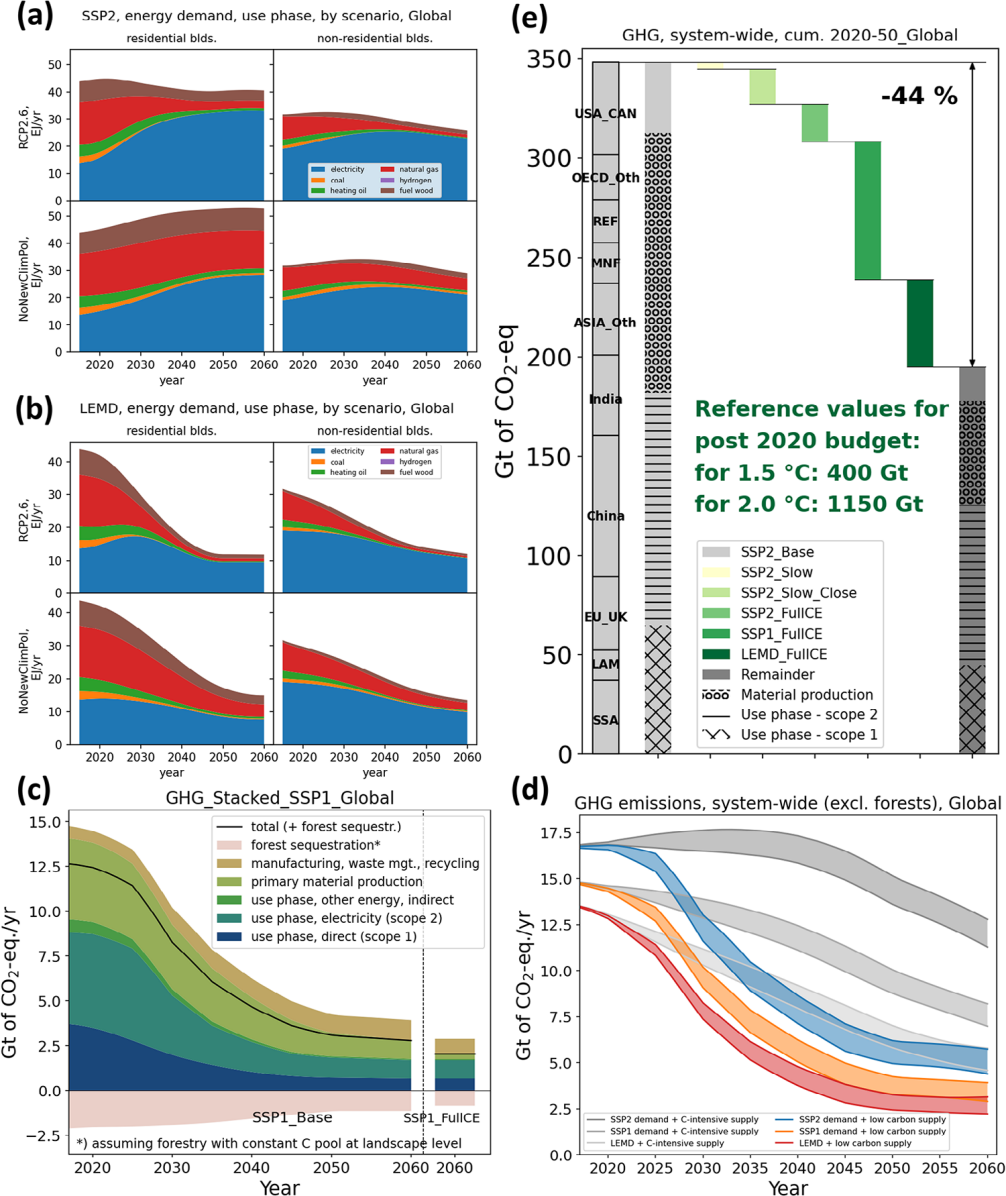
Addressing research question 3: Global final energy demand (*F*_16_7_) for the SSP2 base (a) and low energy and material demand scenario (b) by sub‐sector and climate policy scenario. Emissions by process for the SSP1_Base scenario and the 2060 SSP1_FullCE value for comparison (c). Emissions range (different circular economy [CE] options) for the six combinations of two supply‐side and three demand‐side scenarios (d). Cumulative greenhouse gas emissions by region and sector without and with a waterfall of additional CE strategies (e). The underlying data for this figure are available in Supporting Information S2.

According to the chosen energy system model and excepted building efficiency gains such as building insulation and use of heat pumps, final energy demand is bound to stabilize or decline in all scenarios except for SSP2 with no new climate policy (Figure 4a,b). There is a general trend toward using higher shares of electricity, and in the RCP2.6‐compatible energy supply, electricity will account for 80%–90% of final energy demand by buildings. GHGE in all system processes are bound to decline (Figure 4c, SSP1_Base results are shown as a representative example here), except for GHGE from manufacturing and recycling, where large flows of waste construction wood are incinerated for energy recovery. System‐wide GHGE for SSP1_Base decline to about 1/3 between 2020 and 2060 (Figure 4c, orange in Figure 4d), with future levels much lower than a baseline without new climate policy (medium gray in Figure 4d). Higher reductions down to around 2.5 Gt CO_2_‐eq/yr in 2060 (without forest sequestration or CCS) are only possible if ambitious supply‐side (energy conversion and material production) and demand‐side (lower floor space, energy efficiency, and light‐weighting) measures are combined with higher material recycling and re‐use rates (Figure 4d, LEMD_FullCE combined with RCP2.6). With moderate emissions mitigation in SSP2_Base, 2020–2050 cumulative GHGE from the building sector will account for almost the entire remaining emissions budget for the 1.5°C and about 1/3 of the remaining budget of the 2°C target (Figure 4e). To help tackle this clearly insufficient mitigation effort, the combination of supply‐ and demand‐side and CE measures can reduce system‐wide 2020–2050 cumulative GHGE by 44% (Figure 4e), which is quite a lot for the cumulative indicator that includes all the lock‐in and slow ramp‐up of strategies.

**FIGURE 5:**
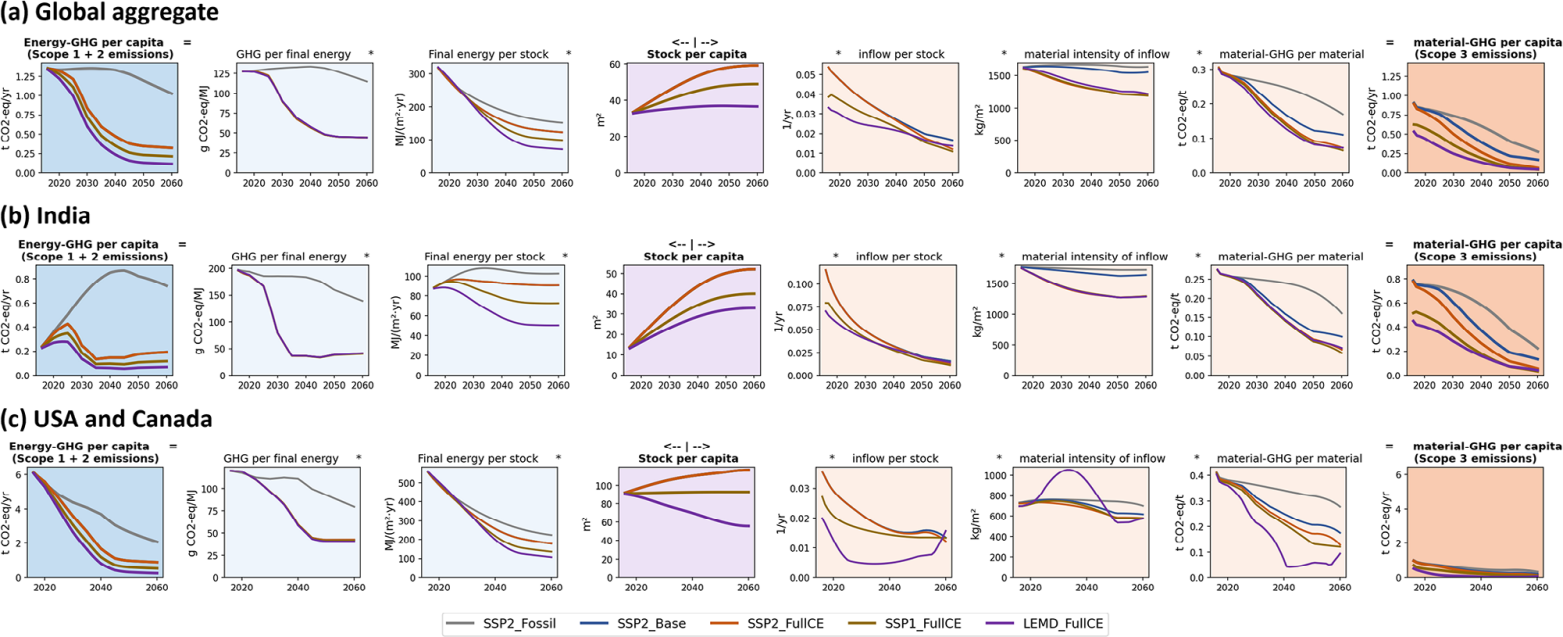
Addressing research question 4: Decomposition analysis according to Equations (1) and (2) for the energy service cascade for the global aggregate (a), India (b), and USA+Canada (c). The in‐use stock (lilac, center) is the basis for both the energy‐related greenhouse gas emission (GHGE) decomposition (scope 1 + 2, blue, left) and the material‐related GHGE decomposition (scope 3, red, right). For some plots, some scenarios have identical values which reduces the number of visible lines. The scope 1 + 2 (left) and scope 3 (right) GHGE scales are the same for each country (row) to facilitate comparison. The underlying data for this figure are available in Supporting Information S2.

Most scenarios show substantial decoupling of per capita GHGE for both scope 1 + 2 (energy supply) and scope 3 (material supply) for the building stock (Figure 5). Despite growing stock levels, energy‐related GHGE declines sharply by reducing the energy consumption per built‐up stock by half to two thirds and the GHGE intensity of energy supply down to a small fraction of current values (Figure 5, blue plots, left). Material‐related GHGE decline as well, driven by less inflow per stock (as stocks saturate and construction declines) and a decline of the GHGE intensity per ton of material supply by about two thirds (combined effect of more recycling/CE and less carbon‐intensive primary production) (Figure 5, red plots, right). Due to the high construction demand in some world regions (India chosen as representative region here), and the slow rise of low‐carbon materials, the material‐related scope 3 emissions are of similar size, and at times even larger, than the scope 1 + 2 emissions. This finding highlights the importance of including these embodied or “gray” emissions in a consistent model approach of the building sector and in the policy debate.

**FIGURE 6:**
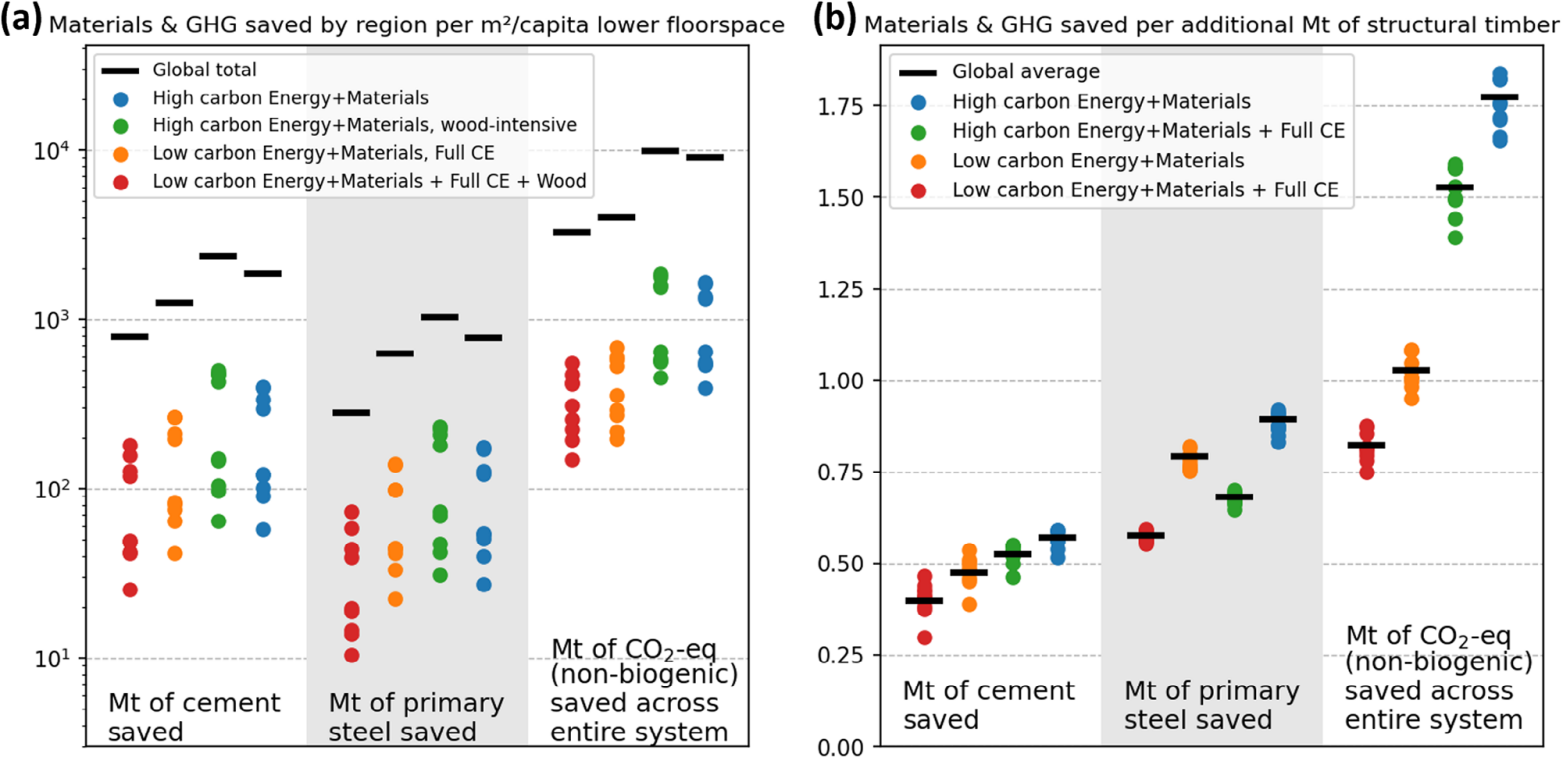
Addressing research question 5: System‐wide elasticities per region (colored dots) and global (black bar) for materials (*F*_4_5_) and greenhouse gas emissions (GHGE; system wide) saved per m^2^/cap of lower building stock level in 2050 (a) and per additional Mt of structural timber (*F*_4_5_) used in buildings (b). The GHGE savings of timber use are based on the assumption of constant carbon pools in forests. These indicators in this figure serve illustrative purposes; they are not meant to be scaled up to large changes in m^2^ of floorspace and Mt of wood. For upscaling, the actual resource efficiency climate change scenario results can be used. The underlying data for this figure are available in Supporting Information S2.

Targeting lower floor spaces of 1 m^2^ less per capita in 2050 will lead to global savings of 800−2500 Mt of cement, 300−1000 Mt of steel, and 3−10 Gt of GHGE, depending on the degree of industry decarbonization and CE roll‐out (Figure 6a). Since these numbers depend on the population, the region‐specific values are a fraction of the global ones (see the data supplement).

When ignoring carbon pool changes in forests, using 1 additional Mt of structural timber leads to average savings of 0.4–0.55 Mt of cement, 0.6–0.85 Mt of steel, and 0.8–1.8 Mt of system‐wide GHGE, depending on the degree of industry decarbonization and CE roll‐out (Figure 6b). While the CE roll‐out has some impact on primary steel demand, the largest difference is the reduced GHGE savings in a low‐carbon energy supply scenario compared to a baseline with now new climate policy.

## 4 DISCUSSION

The lock‐in from existing stocks and the stock expansion especially in countries of the Global South mean that deep decarbonization in the global building sector requires many strategies to work together. Building energy efficiency, floorspace demand reduction, more flexible building designs, low‐carbon energy supply, lightweight design, repurposing of buildings, material circularity, and—sustainable supply provided—low‐carbon building materials are all crucial. Without large existing in‐use stocks, the countries in the Global South face a double disadvantage in establishing a circular economy (Yang et al., 2024): first, due to the need to build up in‐use stocks from primary production and second, due to the lacking opportunity to recycle metals or aggregates from existing stocks (Watari, Giurco et al., 2023). In the Global North, continued floorspace growth hinders the closure of material cycles for buildings in some scenarios (Lotz et al., 2024; van Oorschot et al., 2023).

Material‐related scope 3 emissions contribute substantially to the climate impact of the provisioning system for buildings. In some regions, scope 3 GHGE even dominate. Overall, there is a substantial mitigation potential, up to 44% of cumulative 2020–2050 global sectoral GHGE, that lies in the *narrow, slow, and close* CE strategies. In contrast to often‐heard claims about the CE, for the building sector, the largest mitigation potential does not lie in better recycling or longer building life but in lightweight buildings, different building design, and urban forms that incentivises lower per capita floorspace. Such demand‐side strategies are a prerequisite for a circular low‐carbon economy (Song et al., 2023) or even a sufficiency or degrowth economy (Savini, 2023) and could reduce cumulative 2020–2050 cement and primary steel demand by about 2/3.

While Gallego‐Schmid et al. (2020) emphasize the potential of the *slow* strategies, especially of material re‐use, our work lists re‐use under *close* and shows that the additional GHGE and resource savings potential for major construction materials remains limited, because it is already largely used (for steel) or actually limited due to the material properties (cast‐in‐place concrete and deteriorating quality of wooden elements that require additional testing; Dong et al., 2024).

### 4.1 Comparison with findings in the literature

We agree with Zhong et al. (2021) in highlighting the role of material‐related GHGE in the global building sector, especially in regions with a huge stock growth potential and relatively low building energy demand due to climatic conditions. We find substantially higher building material‐related GHGE (about 3.4/4.4/6.1 Gt CO_2_‐eq/yr in 2020 for LEMD/SSP1/SSP2 vs. 3.6 Gt by Zhong et al. for SSP2), which is due to the larger floorspace and the higher material demand for stock expansion in different regions: we find 1.1–1.8 Gt CO_2_‐eq/yr from China alone, largely driven by cement demand.

Mishra et al. (2022) report a rising trend in material‐related GHGE for new buildings (their figure 3b.2, slope of cumulative emissions is rising), which is the opposite of what a dynamic stock model with increasing recycling and decarbonization yields (our Figure 4c). This difference in the trend is the result of the stylistic representation of building stock dynamics by Mishra et al. They report cumulative 2020–2050 cement and steel‐related GHGE for residential buildings of only 5 Gt CO_2_ max, which is clearly not enough given global stock expansion envisioned for SSP2.

Our final energy consumption results for residential buildings start at levels about 60% higher than those reported by Mastrucci et al. (2021) and remain at high levels in SSP2, because we include domestic hot water along with heating and cooling and calibrated against reported energy use.

Mastrucci and van Ruijven (2023) study future global residential floorspace levels of 200−300 billion m^2^, while this study yields a future range of 250−400 billion m^2^. Hence, their reported material demand for stock expansion is lower than what this study reports (Fig. SP7 in the figure supplement). Material demand scenarios are highly sensitive to future stock levels and the material composition of future buildings. Currently, and beneficial for the global GHGE balance, the world is not on an SSP2 track for building material stock expansion, because actual construction material production and apparent consumption by buildings is lower than the projected SSP2 values of around 6 Gt CO_2_‐eq/yr for building materials alone.

### 4.2 Impacts and limits of using structural timber

The elasticities (Figure 6b) provide a scale‐independent quantification of the benefit of wood use in buildings, including effects from all major changes in the energy system and material cycles. These figures help communicate the climate benefit of wood use, but they ignore forest carbon dynamics and assume that all structural timber is sourced from existing wood harvest from forests with constant carbon pools. They can thus not be used to scale up to levels much larger than what is harvested today. The global supply constraint of wood quickly renders wood‐intensive scenarios infeasible. For the SSP2 scenario, cumulative 2020–2050 industrial roundwood harvest demand ranges between 16 (standard design) and 61 (wood‐intensive design) Gt of C. In the LEMD scenario, the cumulative wood demand is about half as much, ranging from 8 (standard design) and 32 (wood‐intensive design) Gt of C (flow *F*_1_2_). For the wood‐intensive building scenarios, cumulative 2020–2050 construction wood demand is between 3 (LEMD) and 6 (SSP2) times larger than the volumes reported by Mishra et al. (2022), who focus on urban residential expansion to relatively low m^2^/cap values only and do not include the transformation of the entire building stock. When compared to recent extraction statistics and scenarios reported by Johnston and Radeloff (2019), we see that without massive changes in wood supply and the conversion efficiency from roundwood to structural timber products, only the low‐wood scenarios’ demand can be met in most world regions, and only if a much higher share of the harvested roundwood goes into structural timber than what is seen today.

The wood‐intensive scenarios for the entire building stock are clearly infeasible with current harvest rates in most regions, they would require 4−5 times (SSP2) and 2−3 times (LEMD) higher harvest volumes than projected by Johnston and Radeloff (2019). Wood remains a scare resource for construction, and its use in buildings should be capped at levels that can be supplied with reasonable climate, biodiversity, and water cycle impact. Since the conversion rate from roundwood into structural timber in the wood processing industry varies between 30% and 70%, large volumes for other applications of wood, such as packaging or paper, are available also in scenarios where more roundwood is used for structural timber.

### 4.3 Implications for industry and policy

For the major building materials and the material‐related GHGE in this sector, a simple “closing the loop” will not help much if stocks are still growing substantially, and a large part of the buildings and their components are not built for re‐use and recycling, which is the case for concrete and wooden building components, in particular. Destructive concrete recycling saves resources but not GHGE (Mostert et al., 2021). For substantial reductions in building material‐related GHGE, lower floorspace trajectories and light‐weight long‐lived buildings with modular components are a must. To deliver on the Paris Agreement goals, supply‐ *and* demand‐side GHGE mitigation and CE strategies need to become part of national climate and sustainability plans for the building sector, as no side alone can deliver reductions that are quick and large enough.

Due to differences in lifetime, the lock‐in from existing stocks is larger for residential buildings than non‐residential buildings. Promoting high energy efficiency is a crucial climate policy area, as around 70% of the floorspace expansion by 2050 will happen in countries with limited or no building energy regulations currently in place (IEA, 2022). The life cycle trade‐off between longer building lifetime and higher energy efficiency from new buildings needs to be assessed case specific, and incentives for renovation versus demolition or a possible conversion of offices into residential housing should be designed accordingly.

Given the magnitude of material‐related scope 3 emissions, material and circular economy codes for buildings also become necessary. As a first step, countries and regions need to monitor scope 3 emissions of their building sectors and debate about how to reduce these by incentives (industry emissions trading or carbon charges), regulation (light‐weighting standards), or facilitation of re‐use of building components, for example, by certification schemes. In GHGE accounting, building scope 3 will still be part of the industry, and industry mitigation targets need to be informed by and consistent with the expected implications of SDG pathways for the end‐use sectors buildings, transport, or infrastructure. The RECC model and others (Mastrucci & Ruijven, 2023; Zhong et al., 2021) can quantify such linkages for different socioeconomic and climate policy scenarios.

Our results are of immediate relevance for the construction sector and its material suppliers: All m^2^/yr building area and Mt/yr material flows directly show the future scale of industrial activities in the construction, demolition, material production, and recycling sectors. Future material demand directly depends on light‐weighting standards and floorspace levels; any policy on these areas will directly lead to large impacts for material producers and alleviate pressure from natural resources, especially climate, sand, and gravel. The sensitivity figure on floorspace (Figure 6a) highlights the per‐unit impact of low demand futures on material demand and GHGE. These factors can be used in communicating the relevance of demand‐side solutions, or the *narrow* CE strategy, to decouple service provision from resource and carbon‐intensive material production.

### 4.4 Limitations and research outlook

Despite being comprehensive in terms of geographical and system coverage (scopes 1 + 2 + 3), this work comes with a number of necessary simplifications and limitations, and increasing its relevance for policy and industry decision making warrants further inter‐ and trans‐disciplinary research.

For the stock–flow–service nexus, linking stock turnover to the commonly used policy targets demolition and renovation rate is needed, instead of the lifetime model, where future demolition and renovation are predetermined at the moment of construction(Berrill & Hertwich, 2021). Using these rates as parameters will also allow for including agent‐based modeling in renovation decision making (Niamir et al., 2024). Breaking down the scenarios for aggregated regions to the level of individual countries, similar to footprint calculations (Lenzen et al., 2017), will increase the direct policy relevance of the different scenarios.

Inter‐sectoral linkages are relevant at different stages of the energy service cascade. Integrated assessment models and energy system models, in particular, should increase consistency in their depiction of the industry sector by linking demand for industry output (especially primary materials and mining; Aramendia et al., 2023) to the demand from the main end‐use sectors, including buildings and add more technology detail on the end‐use side (Creutzig et al., 2023). Studying material flows across end‐use sectors can make scenarios more realistic. For example, correctly identifying the potential of buildings to act as a sink for secondary steel sourced from vehicles, or the potential for using construction waste in road infrastructure requires scenarios with cross‐sectoral material flows.

The stylistic treatment of forests ignores their dynamics, as it only captures the harvested fraction of the carbon in wood and assumes forest management aimed at constant C pools. A comprehensive treatment of forest carbon dynamics requires *F*_1_2_ to be coupled to a forest growth model, which is beyond RECC's scope. Moreover, the overall potential for using structural timber in global buildings is much larger than the potential supply. Therefore, eventually, a complete assessment of the climate impact of using the potentially available amounts of wood requires the coupling of three models: one for the building sector, one for the other wood applications and the cascade, and a wood supply and land‐use model. For the latter, stylistic models with linear or sigmoidal dynamic functions describing biomass growth (De Rosa et al., 2017, S. 201; Hansen et al., 2024; Le Noë et al., 2020) as well as process‐based models (Gupta & Sharma, 2019; Nölte et al., 2023) are available. Assessments of wood use need to include both perspectives: the net climate impact of the combined forest–harvest–cascade system (Watari et al., 2024) and the estimation of foregone emissions and sequestration in counter‐use scenarios (Aryapratama & Pauliuk, 2022), the so‐called carbon costs of wood harvest (Peng et al., 2023).

Local expert and stakeholder knowledge should be used in establishing the CE strategy potentials and formulating region‐specific building stock transformation scenarios in a co‐creation process. Especially the formulation of realistic LEMD scenarios would benefit from such an exchange between stakeholders and researchers, including working at higher spatial resolution and socioeconomic granularity (Mastrucci et al., 2023). New data, validated by stakeholders, such as the information provided by Lima et al. (2024), should inform region‐specific assessments.

The current archetypes (Krych et al., 2024) are rather generic and suitable to study the broad implications of global building stock expansion for cement, steel, and timber at a more aggregate regional scale. More accurate data on the material composition of the existing building stock (Fishman et al., 2024; Vilaysouk et al., 2022) and building archetypes with region‐specific features and conditions, such as local construction materials, temperatures, humidity, or earthquakes can lead to realistic country‐ and region‐level scenarios. To that end, the consistency between age–structure of the population, household size, and dwelling units needs to increase in future scenarios (Berrill et al., 2021, 2022), as well as the urban–rural split (Zhang et al., 2024) with urbanization as a key driver (Schiller & Roscher, 2023). Such density and demography‐consistent scenarios (Güneralp et al., 2017), even down to the city scale, will allow for linking building stock scenarios with extrapolations for vehicles (Pérez‐Sánchez et al., 2024), infrastructure (Rankin & Saxe, 2023), lifestyles (Pettifor et al., 2023), green spaces (Oorschot et al., 2024), urban climate (Ortlepp et al., 2021), inequality in building service access (Poblete‐Cazenave et al., 2021), and the re‐purposing of buildings (Opher et al., 2021). Such multi‐sector integration will be crucial for assessing urban and rural futures consistently and linking them to lifestyles and well‐being. Finally, expanding the material scope beyond the structural materials to include insulation, cladding, windows, piping, and wiring will allow for more accurate depiction of the technology material requirement of buildings.

## CONFLICT OF INTEREST STATEMENT

The authors declare no conflict of interest.

## Supporting information



• Supplementary material 1: RECC model brief, scenario details, and traceability of results

• Supplementary material 2: Data supplement with all numbers behind the figures and other results reported in the text.

• Supplementary material 3: Figure supplement: Region‐specific plots and supplementary figures.
• The RECC v2.5 model documentation: https://doi.org/10.6094/UNIFR/242061
• The RECC v2.5 model repository: https://github.com/IndEcol/RECC‐ODYM
• The RECC v2.5 global buildings input and results dataset: https://zenodo.org/records/12752350
• The BuildME modeling framework for building archetypes: https://github.com/nheeren/BuildME (Krych et al., 2024)

## Data Availability

This work is the result of a larger open science effort to depict the future of the energy and material service cascade in different sectors and regions. It comes with a number of supporting documents, datasets, and models that allow other researchers to reproduce our results or build their own scenarios. Supplementary material 1 provides the RECC model brief, scenario details, and result traceability, while Supplementary material 2 offers a data supplement containing all figures' numerical data and other results reported in the text. Region‐specific plots and additional figures are available in Supplementary material 3. The RECC v2.5 model documentation is accessible at https://doi.org/10.6094/UNIFR/242061, with the RECC v2.5 model repository and global buildings input and results dataset available at https://github.com/IndEcol/RECC‐ODYM and https://zenodo.org/records/12752350, respectively. The BuildME modeling framework for building archetypes can be found at https://github.com/nheeren/BuildME (Krych et al., 2024).
