# Supplementary material for: Scenario analysis of supply‐ and demand‐side solutions for circular economy and climate change mitigation in the global building sector
Source: J Ind Ecol. 2024 Oct 8;28(6):1699–715. doi: 10.1111/jiec.13557 (PMC11667659; doi:10.1111/jiec.13557)
Supplement: Supplementary file 1 — • Supplementary material 1: RECC model brief, scenario details, and traceability of results [file JIEC-28-1699-s001.docx]

*Freiburg, Germany, summer 2024*

*Scenario analysis of supply- and demand-side solutions for circular economy and climate change mitigation in the global building sector*

***Supplementary material 1: RECC model brief, scenario details, and traceability***

*Stefan PAULIUK^1,*^ Fabio CARRER, Niko HEEREN, Edgar G HERTWICH*

^1)^ Faculty of Environment and Natural Resources, University of Freiburg, Germany

^*)^ Address correspondence to

Stefan Pauliuk

Faculty of Environment and Natural Resources

University of Freiburg

Tennenbacher Strasse 4

D-79106 Freiburg, Germany

[stefan.pauliuk@indecol.uni-freiburg.de](mailto:stefan.pauliuk@indecol.uni-freiburg.de)

**Content:**

**Part 1: RECC model brief and scenario overview**

- 1.1. RECC model brief
- 1.2. RECC v2.5 global building stock model resolution
- 1.3. 2015 reference state of the global building stock, overview
- 1.4. Scenario narratives and parameter target values for the global building stock,

circular economy strategy potential implemented

- 1.5. Scenario definitions, result overview and tracing of numerical results

**Part 2: Part 2: RECC v2.5 model and database change log compared to RECC v2.5 documentation**

- 2.1. Changes to the database compared to RECC v2.5 base version
- 2.2. Model improvement and fixes compared to RECC v2.5 base version
- 2.3. Sankey diagram export
- References

**Part 1: RECC model brief and scenario overview**

- 1. **RECC model brief**

**RECC – Resource Efficiency – Climate Change mitigation framework**

**An open scenario development tool for material cycles and the circular economy**

**Overview:** The resource efficiency–climate change (RECC) mitigation model framework is a step towards the interdisciplinary scientific assessment of material efficiency and its links to service provision, material cycle management, and climate policy. RECC is based on dynamic material flow analysis and links the services provided (individual motorized transport and shelter) to the operation of in-use stocks of products (passenger vehicles and residential buildings), to their expansion and maintenance, and to their material cycles to model mitigation strategies and analyze trade-offs for environmental impacts along the products’ life cycle.

A key innovation of RECC is the upscaling of product archetypes with different degrees of material and energy efficiency, which are simulated with engineering tools. RECC scenarios are driven by parameters that augment the storylines of the shared socioeconomic pathways (SSP) to describe future service demand and associated material requirements. In its current implementation (model versions 2.5 (2023) and 2.6 (scheduled for 2024)), ten material efficiency strategies at different stages of the material cycle can be assessed individually or combined into bundles of strategies, by ramping up their implementation rates to the identified technical potentials.

RECC provides scenario results for the life cycle impacts of ambitious service–material decoupling concurrent with energy system decarbonization, giving detailed insights on the RECC mitigation nexus to policy-makers worldwide.

**RECC developers:** Stefan Pauliuk and the research group for industrial ecology and socio-metabolic research in Freiburg, Niko Heeren, Zurich municipal administration, Edgar Hertwich and the industrial ecology program in Trondheim, multiple users, software, and data contributors across the world

**Central RECC resource (landing page):**

<https://www.industrialecology.uni-freiburg.de/odym-recc>

**Core RECC features:**

• Start with a service scenario (m² buildings, vehicle-km), calculate the in-use stocks needed for service provision, the stock turnover, the material cycle, the energy and material supply for all processes, and env. impacts.

• Global coverage: single EU country resolution, large world economies, aggregated world regions, from 2020 to 2026

• High product detail: 13 residential and 24 non-residential building types, 6 drive technologies, 150 future building & 24 future vehicle archetypes

• 3 socio-economic (low demand, SSP1, SSP2) and two climate policy (no new policy and RCP2.6 compatible energy supply) scenarios available

• Up to 10 circular economy strategies on top of each scenario

• Implemented in Python, data input and output via excel files

**RECC system definition:**


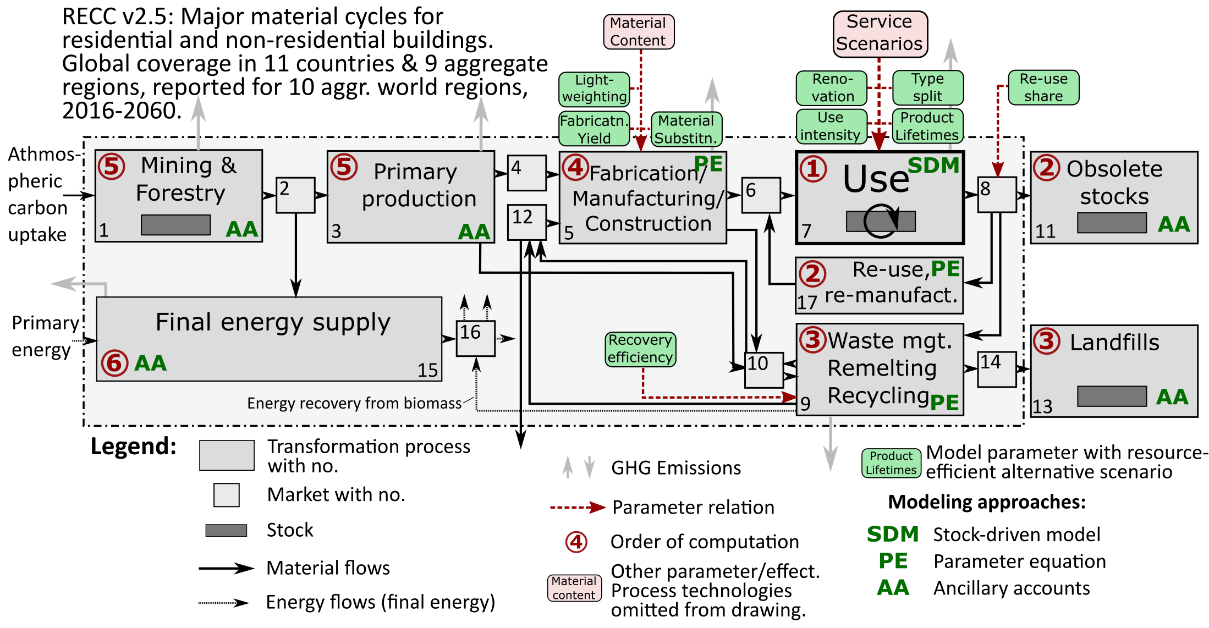


**Fig S1:** RECC v2.5 system definition.

**Central references:**

The complete RECC v2.5 model docu is available via <https://doi.org/10.6094/UNIFR/242061>.

Haberl, H., Wiedenhofer, D., Erb, K.-H., Görg, C., Krausmann, F., 2017. The Material Stock–Flow–Service Nexus: A New Approach for Tackling the Decoupling Conundrum. Sustainability 9, 1049. https://doi.org/10.3390/su9071049

Kalt, G., Wiedenhofer, D., Görg, C., Haberl, H., 2019. Conceptualizing energy services: A review of energy and well-being along the Energy Service Cascade. Energy Res. Soc. Sci. 53, 47–58. https://doi.org/10.1016/j.erss.2019.02.026

Pauliuk, S., Fishman, T., Heeren, N., Berrill, P., Tu, Q., Wolfram, P., Hertwich, E., 2021a. Linking service provision to material cycles: A new framework for studying the resource efficiency–climate change (RECC) nexus. J. Ind. Ecol. 25, 260–273. https://doi.org/10.1111/jiec.13023

Pauliuk, S., Heeren, N., Berrill, P., Fishman, T., Nistad, A., Tu, Q., Wolfram, P., Hertwich, E., 2021b. Global scenarios of resource and emission savings from material efficiency in residential buildings and cars. Nat. Commun. 12, 5097. https://doi.org/10.1038/s41467-021-25300-4

**Contact:**

[stefan.pauliuk@indecol.uni-freiburg.de](mailto:stefan.pauliuk@indecol.uni-freiburg.de)

Faculty of Environment and Natural Resources, University of Freiburg

Tennenbacher Strasse 4

D-79106 Freiburg, Germany.

- 1. **RECC v2.5 global building stock model resolution**

Table S1 shows the regional resolution of the study for the global building stock.

**Table S1:** RECCv2.5 global building stock study country and regional resolution. The left column introduces the region labels used in the paper, the right column shows the aggregation from the 20 RECC v2.5 native regions.

| **REGION** | **DESCRIPTION** | **COUNTRIES INCLUDED** | **RECC CODE** |
| --- | --- | --- | --- |
| **MENA / MNF** | Middle East and Northern Africa, excluding Israel (included in Rest of OECD region) | Bahrain, Kuwait, Oman, Qatar, Saudi Arabia, United Arab Emirates, Iran, Iraq, Jordan, Lebanon, Occupied Palestinian Territory, Syrian Arab Republic, Yemen, Algeria, Egypt, Libyan Arab Jamahiriya, Morocco, Tunisia, Western Sahara. | R5.2MNF_Other |
| **Sub-Saharan Africa / SSA** |  | Angola, Botswana, Equatorial Guinea, Gabon, Mauritius, Mayotte, Namibia, Réunion, Seychelles, Benin, Burkina Faso, Burundi, Cameroon, Cape Verde, Central African Republic, Chad, Comoros, Congo, Côte d`Ivoire, Democratic Republic of the Congo, Djibouti, Eritrea, Ethiopia, Gambia, Ghana, Guinea, Guinea-Bissau, Kenya, Lesotho, Liberia, Madagascar, Malawi, Mali, Mauritania, Mozambique, Niger, Nigeria, Rwanda, Sao Tome and Principe, Senegal, Sierra Leone, Somalia, South Sudan, Sudan, Swaziland, Togo, Uganda, United Republic of Tanzania, Zambia, Zimbabwe, South Africa. | R5.2SSA_Other |
| **Latin America / LAM** | Includes the countries of Latin America and the Caribbean, excluding Mexico and Chile (included in the rest of OECD region) | Antigua and Barbuda, Argentina, Bahamas, Barbados, Belize, Bermuda, Bolivia, Brazil, Chile, Colombia, Costa Rica, Cuba, Dominica, Dominican Republic, Ecuador, El Salvador, French Guiana, Grenada, Guadeloupe, Guatemala, Guyana, Haiti, Honduras, Jamaica, Martinique, Netherlands Antilles, Nicaragua, Panama, Paraguay, Peru, Saint Kitts and Nevis, Saint Lucia, Saint Vincent and the Grenadines, Suriname, Trinidad and Tobago, Uruguay, Venezuela. | R5.2LAM_Other |
| **Rest of Asia / ASIA_OTH** | Asian countries excluding China, India, (individual regions), the Middle East (included in MENA), and Former Soviet Union states (included in REF) | Indonesia, Pakistan, Taiwan, Cambodia, Lao People's Democratic Republic, Mongolia, Viet Nam, Bhutan, Brunei Darussalam, French Polynesia, Guam, Malaysia, Maldives, New Caledonia, Singapore, Sri Lanka, Thailand, Bangladesh, Democratic People's Republic of Korea, Fiji, Micronesia (Fed. States of), Myanmar, Nepal, Papua New Guinea, Philippines, Samoa, Solomon Islands, Timor-Leste, Tonga, Vanuatu | R5.2ASIA_Other |
| **Reforming Econ. / REF** | Reforming Economies of Eastern Europe and the Former Soviet Union, including those of central Asia, excluding EU countries (included in EU12-H and EU12-M) | Russian Federation, Albania, Bosnia and Herzegovina, Croatia, Montenegro, Serbia, The former Yugoslav Republic of Macedonia, Belarus, Republic of Moldova, Ukraine, Armenia, Azerbaijan, Georgia, Kazakhstan, Kyrgyzstan, Tajikistan, Turkmenistan, Uzbekistan. | R5.2REF_Other |
| **Rest of OECD / OECD_Oth** | Includes the OECD 90 and EU member states and candidates, excluding countries defined as individual regions or included in EU15, EU12-H, or EU12-M | Australia, New Zealand, Turkey, Mexico, Chile, Iceland, Norway, Switzerland, Israel, Republic of Korea (South Korea), Japan | R5.2OECD_Other  R32JPN |
| **China** |  | China, Hong Kong SAR, Macao SAR. | R32CHN |
| **India** |  | India | R32IND |
| **Canada and USA / USA_CAN** |  | United States of America, Puerto Rico, United States Virgin Islands, and Canada | R32USA  R32CAN |
| **EU and UK / EU_UK** | EU27 states (2023) and the UK | Austria, Belgium, Denmark, Finland, Greece, Ireland, Luxembourg, Netherlands, Portugal, Sweden, Cyprus, Czech Republic, Estonia, Hungary, Malta, Slovakia, Slovenia, Bulgaria, Latvia, Lithuania, Romania, France, Germany, Italy, Poland, Spain, UK | France  Germany  Italy  Poland  Spain, UK  Oth_R32EU15  Oth_R32EU12-H  R32EU12-M |

**List of residential building types covered:** For each residential building type, except for informal buildings, there are for building archetypes for future age-cohorts: one with standard design, one with light-weight design, one with material substitution (wood intensive), and one with light-weighting and material substitution combined. (SFH = single family house, MFH = multi-family house, RT = residential tower, ZEB: zero energy building):

- SFH_non-standard
- SFH_standard
- SFH_efficient
- SFH_ZEB (zero energy building)
- MFH_non-standard
- MFH_standard
- MFH_efficient
- MFH_ZEB
- RT_non-standard
- RT_standard
- RT_efficient
- RT_ZEB
- informal_non-standard

**List of non-residential building types covered:** For each non-residential building type, there are for building archetypes for future age-cohorts: one with standard design, one with light-weight design, one with material substitution (wood intensive), and one with light-weighting and material substitution combined. (ZEB: zero energy building):

- nonres_offices_non_standard
- nonres_offices_standard
- nonres_offices_efficient
- nonres_offices_ZEB
- nonres_commercial_non_standard
- nonres_commercial_standard
- nonres_commercial_efficient
- nonres_commercial_ZEB
- nonres_education_non_standard
- nonres_education_standard
- nonres_education_efficient
- nonres_education_ZEB
- nonres_health_non_standard
- nonres_health_standard
- nonres_health_efficient
- nonres_health_ZEB
- nonres_hotels_restaurants_non_standard
- nonres_hotels_restaurants_standard
- nonres_hotels_restaurants_efficient
- nonres_hotels_restaurants_ZEB
- nonres_other_non_standard
- nonres_other_standard
- nonres_other_efficient
- nonres_other_ZEB

**List of engineering materials covered:** The materials in bold font are in the focus of the analysis. The other materials are only partly covered and often use proxy data. Their production and recycling is included in the scope 3 / material cycle part of the analysis. (Al: aluminium)

- **construction grade steel**
- stainless steel
- cast iron
- wrought Al
- cast Al
- copper electric grade
- plastics
- **cement**
- **wood and wood products**
- zinc
- concrete
- **concrete aggregates**
- bricks
- glass
- insulation material
  1. **2015 reference state of the global building stock, overview**

This SI section summarizes the 2015 reference state of global buildings and the scenario narratives for the development of the global building stock. Here, an overview is given. All data and detailed data tracing can be found in the log sheets of the individual parameter files.

**Table S2 a-c:** Overview of the 2015 building stock per capita for residential (reb) and non-residential (nrb) buildings, its total material content, and its reported (calibrated) total energy consumption. Between 2050 and 2060, stock target values are left constant. A rationale for the values for the residential buildings is given by Fishman et al. (2021), and a rationale for non-residential buildings is given around Table S3 below.

| 1. **Scenario:** | **BASE** | **LEMD** | **SSP1** | **SSP2** |
| --- | --- | --- | --- | --- |
| **Region / year** | **2015** | **2050** | **2050** | **2050** |
| R5.2SSA (reb) | 0.83 | 7 | 10 | 12 |
| R5.2SSA (nrb) | 11.43 | 19.41 | 26.86 | 33.38 |
| R5.2LAM (reb) | 3 | 7 | 10 | 12 |
| R5.2LAM (nrb) | 34.43 | 30.30 | 34.43 | 44.28 |
| EU_UK (reb) | 12.55 | 12.78 | 16.08 | 19.99 |
| EU_UK (nrb) | 37.69 | 31.17 | 40.09 | 46.20 |
| China (reb) | 10.8 | 13 | 16 | 20 |
| China (nrb) | 36.05 | 31 | 40 | 50 |
| India (reb) | 0.83 | 7 | 10 | 12 |
| India (nrb) | 11.74 | 25.02 | 28.75 | 38.06 |
| R5.2ASIA_Oth (reb) | 2.60 | 7.47 | 10.54 | 12.63 |
| R5.2ASIA_Oth (nrb) | 20.78 | 29.42 | 34.32 | 39.00 |
| R5.2MNF (reb) | 8.3 | 9 | 12 | 15 |
| R5.2MNF (nrb) | 24,.57 | 29.63 | 38.94 | 43.60 |
| R5.2REF (reb) | 5.9 | 9 | 12 | 15 |
| R5.2REF (nrb) | 23.47 | 29.5 | 38.87 | 43.52 |
| R5.2OECD_Oth (reb) | 6.5 | 9 | 12 | 15 |
| R5.2OECD_Oth (nrb) | 37.97 | 30.5 | 39.86 | 44.52 |
| R32USACAN (reb) | 24.13 | 18 | 26 | 30 |
| R32USACAN (nrb) | 66.80 | 42.47 | 66.75 | 83.74 |
| **Global (reb)** | **6.11** | **9.11** | **12.56** | **15.05** |
| **Global (nrb)** | **26.43** | **27.74** | **35.65** | **43.01** |

| 1. **Energy cons., use phase, res+non-res buildings, EJ/yr** | | | | |  |
| --- | --- | --- | --- | --- | --- |
| **Region** | | **2015** | | |  |
| R5.2SSA | | 1.1 | | |  |
| R5.2LAM | | 2.1 | | |  |
| EU_UK | | 15.6 | | |  |
| China | | 12.6 | | |  |
| India | | 1.4 | | |  |
| R5.2ASIA_Other | | 6.8 | | |  |
| R5.2MNF | | 4.2 | | |  |
| R5.2REF | | 8.8 | | |  |
| R5.2OECD_Other | | 3.9 | | |  |
| R32USACAN | | 19.0 | | |  |
| **Global** | | **76.0** | | |  |
| 1. **2015 in-use stock of materials in buildings, Mt** | | | | | |
| **Region** | **Steel** | | **Struct. Timber** | **Cement** | |
| R5.2SSA | 341 | | 1371 | 654 | |
| R5.2LAM | 522 | | 2085 | 978 | |
| EU_UK | 1174 | | 567 | 2529 | |
| China | 2435 | | 2220 | 4565 | |
| India | 530 | | 1898 | 1024 | |
| R5.2ASIA_Other | 791 | | 2665 | 1754 | |
| R5.2MNF | 348 | | 1240 | 602 | |
| R5.2REF | 240 | | 583 | 463 | |
| R5.2OECD_Other | 180 | | 1157 | 736 | |
| R32USACAN | 311 | | 2524 | 1332 | |
| **Global** | **6873** | | **16309** | **14638** | |

- 1. **Scenario narratives and parameter target values for the global building stock, circular economy strategy potential implemented**

Global building demand is driven by exogenous scenarios for per capita floor space by region and socio-economic scenario. The starting point for residential buildings are the scenario values developed for RECC v2.4, described by Fishman et al. (2021) in section ‘3.1 Dwelling service provision scenarios’:

*“Per capita residential floor space varies widely across countries at similar levels of development and economic activity (Ellsworth-Krebs, 2020), shaped by tradition, urban form, as well as land use and building regulations. Scenarios for future floor space were based on the starting point of the country in question, past dynamics, and the climate-friendliness of the underlying scenario, resulting in distinct trends in each scenario (Figure 2).*

*SSP2 describes a scenario of continued growth of floor space per capita in all regions. Although there has been a large difference in per-capita living space in countries at a similar stage of development, there has been a general trend of increasing floor-space with growing GDP, followed by an eventual slowing of demand (OECD/IEA, 2017).We assumed that this trend continues for SSP2. The values obtained with the data-driven method for the USA and Japan conform to this trend. We used them in SSP2 for 2015–2060 and for formulating the scenario values of countries that were found to have had similar historical trends, for example, Canada and the USA, and Germany and Japan. In SSP2, regions generally converge towards 50 m²/cap by 2050–2060. The exceptions are the USA and Canada which have already exceeded this value, and conversely India and the rest of Asia region which reach an average of 40 m²/cap by 2060, and Sub-Saharan Africa reaching 35 m²/cap due to the infeasibility of them reaching 50m²/cap by 2060 based on their current levels.*

*In the SSP1 scenario, if a region has an average floor space per capita of over 40 m²/cap in 2015, its value is held fixed. Regions whose average floor space per capita is below 40m²/cap in 2015 grow towards this value by 2060 but remain below their projected SSP2 levels. The LED scenario calls for a global convergence of floor space per capita of 30 m²/cap by 2050 (Grubler et al., 2018), but does not provide detail on how that is to be achieved or whether this convergence varies by regions. We deemed all regions to either contract or enlarge their per-capita floor area towards this value of 30 m²/cap by 2060, and do so more rapidly after 2030. Regions that are far higher or lower from this value in 2015 do not reach it by 2060. Nevertheless, comparatively speaking their efforts (e.g., the reduction in the USA) are among the most ambitious. The portion of total floor space that is heated or cooled is assumed to remain the same as in 2015 or increase slightly by 2050 in all regions and scenarios.”*

This scenario specification for residential buildings is completed by an analogue narrative extension of the general SSP and LED narratives to non-residential buildings:

From the Grubler et al. (2018) LED scenario, we calculate typical future per capita stocks of 23 m²/cap for the Global North and 9 m²/cap for the Global South for non-residential buildings (nrb). These serve as orientation and reference for assigning target values to individual countries. Table S3 shows the assumed future target values for different groups of the 20 RECC v2.5 native regions and the rational for the values in these groups.

Overall, there are nrb-intensive countries and regions, with 2015 values between 15-25 m²/cap, nrb-lean regions with 2015 in-use stocks of about 10 m²/cap, and nrb-poor regions and countries with 2015 values of 3 m²/cap and below. We assume different target values for these groups for 2050.

**Table S3a:** Overview of 2050 target values for residential buildings at the RECC v2.5 native region level. Extracted from 2_S_RECC_FinalProducts_Future_resbuildings_V2.7.

| **m²/capita** | **2015** | **LEMD** | **SSP1** | **SSP2** |
| --- | --- | --- | --- | --- |
| R32USA | 67.5 | 43 | 67.5 | 85 |
| R32JPN | 39.0 | 32 | 39.0 | 44.7 |
| R32CAN | 60.7 | 38 | 60.7 | 73 |
| R32CHN | 36.1 | 31 | 40 | 50 |
| R32IND | 11.7 | 25 | 28.7 | 38.1 |
| France | 39.8 | 31.8 | 40.9 | 46.3 |
| Germany | 42.7 | 32.2 | 43.3 | 49.6 |
| Italy | 39.6 | 31.8 | 40.7 | 46.1 |
| Poland | 25.2 | 29.5 | 36.2 | 42 |
| Spain | 36.3 | 31.1 | 37.8 | 43.6 |
| UK | 40.8 | 31.8 | 40.9 | 47.3 |
| R32EU12-M | 26.3 | 29.5 | 37.2 | 43.7 |
| Oth_R32EU12-H | 36.2 | 30.5 | 37.9 | 44.4 |
| Oth_R32EU15 | 38.9 | 30.5 | 39.9 | 46.4 |
| R5.2OECD_Other | 38.0 | 30.5 | 39.9 | 44.5 |
| R5.2REF_Other | 23.5 | 29.5 | 38.9 | 43.5 |
| R5.2ASIA_Other | 18.4 | 29.2 | 33.9 | 38.5 |
| R5.2MNF_Other | 24.6 | 29.6 | 38.9 | 43.6 |
| R5.2LAM_Other | 34.4 | 30.3 | 34.4 | 44.3 |
| R5.2SSA_Other | 11.4 | 19.4 | 26.9 | 33.4 |

**Table S3b:** Overview of 2050 target values for non-residential buildings at the RECC v2.5 native region level. Extracted from 2_S_RECC_FinalProducts_Future_NonResBuildings_V2.4.

| **m²/capita** | **2015** | **LEMD** | **SSP1** | **SSP2** |
| --- | --- | --- | --- | --- |
| USA | 24,3 | 18 | 26 | 30 |
| Canada | 22,6 | 18 | 26 | 30 |
| Germany | 21,3 | 18 | 23 | 28 |
| nrb-intensive: efficient reduction to 18 m²/cap for LEMD, moderate increase for SSP1, 25%-50% increase for SSP2, reflecting further uncapped and unregulated expansion | | | | |
| Japan | 16 | 13 | 16 | 20 |
| China | 10,8 | 13 | 16 | 20 |
| France | 13,7 | 13 | 16 | 20 |
| Poland | 10 | 13 | 16 | 20 |
| UK | 11,55 | 13 | 16 | 20 |
| Oth_R32EU15 | 14 | 13 | 16 | 20 |
| Oth_R32EU12-H | 12,83 | 13 | 16 | 20 |
| nrb-lean: efficient reduction to 13 m²/cap for LEMD, moderate to large increase for SSP1, 25%-100% increase for SSP2: further uncapped & unregulated expansion | | | | |
| Italy | 7,4 | 9 | 12 | 15 |
| Spain | 7,5 | 9 | 12 | 15 |
| OECD_Other | 6,5 | 9 | 12 | 15 |
| REF_Other | 5,9 | 9 | 12 | 15 |
| MNF_Other | 8,3 | 9 | 12 | 15 |
| R32EU12-M | 5,87 | 9 | 12 | 15 |
| nrb-lean: efficient growth to 9 m²/cap for LEMD, moderate to large increase for SSP1, substantial to massive, 80%-260% increase for SSP2, reflecting further uncapped & unregulated expansion | | | | |
| India | 0,83 | 7 | 10 | 12 |
| LAM_Other | 3 | 7 | 10 | 12 |
| SSA_Other | 0,83 | 7 | 10 | 12 |
| ASIA_Other | 0,84 | 7 | 10 | 12 |
| nrb-poor: efficient growth to 7 m²/cap for LEMD, very large increase for SSP1 even a bit larger for SSP2, reflecting further uncapped & unregulated expansion | | | | |

These results enter the target table (Table S2a) above for residential and non-residential building stock in 2050 by region and socio-economic scenario for the ten aggregated countries and regions. Between 2050 and 2060, stock target values are left constant.

**Overview of CE strategy potentials:**

The different CE strategies implemented in the RECC model (Pauliuk 2023) are grouped into the three broad categories, narrow, slow, and close (Bocken u. a. 2016), for which the following potentials are applied: see Table 2 in the paper and the different parameter files linked there for details.

**Overview of product and energy mix:**

The mix of different end-use products in final consumption (here: the building type split and the energy technology mix of new and existing buildings) is part of the SSP-RCP scenario matrix.

The building type split (split of newly built residential and non-residential m² into different building types and energy standards) is documented in the log sheets of the parameter files for 3_SHA_TypeSplit_Buildings_V2.0 and 3_SHA_TypeSplit_NonResBuildings.

The future building energy carrier split is modelled to reflect scenario results from the IEA’s World Energy Outlook 2022 (IEA 2022). See the log sheet of the parameter files 3_SHA_EnergyCarrierSplit_Buildings and 3_SHA_EnergyCarrierSplit_NonResBuildings_V2.1 for details on how the data were extracted and converted. From Fig. 3.16 in IEA (2022), we see that the 2050 building heating and DHW energy is composed of renewables (bioenergy, solar thermal and geothermal), electricity, and district heating. From these values, the following default split is extracted.

**Table S4:** Future default energy carrier split of residential and non-residential buildings (final energy).

| **Energy carrier** | **2030** | **2050** |
| --- | --- | --- |
| coal | 0,01 | 0 |
| diesel | 0,09 | 0,03 |
| natural gas | 0,24 | 0,09 |
| electricity | 0,5 | 0,67 |
| fuel wood | 0,11 | 0,11 |
| Other | 0,05 | 0,1 |

In the RECC v.5 database, we replaced the existing energy carrier splits by the latest IEA values for all regions where a substantial bioenergy use in buildings is not really plausible for 2050 (only CAN, REF are expected to have large direct use of wood for heating).

The ‘other category’ is not included in the calculation of impacts from energy supply. The shares or the other categories don't add up to 100% but to 95% (2030) and 90% (2050) to account for a 10% contribution of geothermal energy, waste to energy, or solar thermal energy, for which no climate impact is accounted for.

**Table S5.** Overview of the future material production technologies implemented. Unit: 1. Extracted directly from 4_SHA_MaterialsTechnologyShare_V1.0. Materials not listed below have only one default production technology. The environmental pressure values for the different technologies are listed in 4_PE_ProcessExtensions_Materials_V1.2 (for material, land, and water use) and 4_EI_ProcessEnergyIntensity_V3.2 (for energy use).

| **Material production technology share in total primary production** | **2015 shares** | **2050 shares, NoClimPol** | **2050 shares, low carbon material production** |
| --- | --- | --- | --- |
| production of construction grade steel, primary | 1 | 1 | 0 |
| production of construction grade steel, H2 | 0 | 0 | 1 |
| production of stainless steel, primary | 1 | 1 | 0 |
| production of stainless steel, H2 | 0 | 0 | 1 |
| production of cast iron, primary | 1 | 1 | 0 |
| production of cast iron, H2 | 0 | 0 | 1 |
| production of wrought Al, primary | 1 | 1 | 0 |
| production of wrought Al, efficient | 0 | 0 | 1 |
| production of cast Al, primary | 1 | 1 | 0 |
| production of cast Al, efficient | 0 | 0 | 1 |

From previously used scenarios (see the parameter log), we have already high shares of bioenergy+CCS in electricity mix for CAN, CHN (only 5%) JPN, FRA, GER, ITA, SPN, UK, Oth_R32EU15, Other Asia, LAM, SSA: This can be fed partly with internal wood waste from buildings, which will be substantial.

Tables S5 and S6 report the shared of the future material production technologies implemented and the future electricity mix. See the parameter database for references.

**Table S6:** Overview of the future electricity mix implemented. Unit: %. Extracted directly from 4_SHA_ElectricityMix_World_V1.0. For 2020, the average of both climate scenarios is reported, as there is hardly any difference yet. For exact values, see 4_SHA_ElectricityMix_World_V1.0. For the region-specific electricity mixes, see 4_SHA_ElectricityMix_V1.0

| **Electricity generation technology** | **2020 shares** | **2050 shares, NoClimPol** | **2050 shares, low carbon el. mix** |
| --- | --- | --- | --- |
| solar photovoltaic power plant | 2.8 | 15.6 | 43.2 |
| concentrating solar power plant (CSP) | 0.04 | 0.02 | 0.09 |
| wind power plant onshore | 5.1 | 12.3 | 11.0 |
| wind power plant offshore | 0.2 | 1.4 | 1.5 |
| hydro power plant | 15.4 | 11.4 | 11.5 |
| nuclear power plant | 10.7 | 5.4 | 2.5 |
| coal power plant | 40.6 | 39.1 | 0.02 |
| coal power plant without abatement measures | 0 | 0 | 0 |
| bio powerplant | 1.7 | 2.8 | 0.6 |
| oil power plant | 3.8 | 3.1 | 0.9 |
| geothermal power plant | 0 | 0 | 0 |
| IGCC power plant | 0 | 0 | 0 |
| light oil combined cycle | 0 | 0 | 0 |
| gas combined cycle power plant | 19.7 | 8.8 | 4.7 |
| advanced coal power plant with CCS | 0 | 0 | 0 |
| coal power plant with CCS | 0 | 0 | 2.0 |
| biomass power plant with CCS | 0 | 0 | 5.4 |
| gas combined cycle power plant with CCS | 0 | 0 | 16.5 |

The numbers above represent the 2020 and 2050 values, the parameter files contain the entire time series. For calculating environmental pressure of total electricity demand, the scenario results for electricity demand from the different processes are summed up, divided into the contributions of individual generation technologies as shown here, and multiplied with technology-specific pressure factors, which are mostly extracted from ecoinvent and compiled in 4_PE_ProcessExtensions_Industry_V1.2.

- 1. **Scenario definitions, result overview and tracing of numerical results**

This section documents the scenarios defined, the information needed to reproduce the results, and the documentation of links between model results and indicators presented in the paper.

**Scenario definition:**

Table S7 lists the scenarios that were selected for the plots and tables. The darker colour indicates the main scenarios that appear as baseline or target line in the different plots. The light colour indicates ancillary scenario that are used for sensitivity analysis. The right column indicates the figure number where the results are shown.

**Table S7:** Overview of the scenarios defined for the evaluation of the model results.

| **Main scenarios** | **SSP** | **RCP** | **Description** | **Figures (paper)** |
| --- | --- | --- | --- | --- |
| LEMD_Base | LED | RCP2.6 | Low material and energy demand (LEMD), efficiency and ren. energy, no CE, low wood | 2a+b, 4b+d |
| SSP1_Base | SSP1 | RCP2.6 | SSP1, efficiency and ren. energy, no CE, low wood | 2a+b, 4c+d |
| SSP2_Base | SSP2 | RCP2.6 | SSP2 socio-economics, efficiency and low carbon renewable energy, no CE, low wood | 2a+b, 3a+b, 4a+d+e, 5, 6b |
| SSP2_FullCE | SSP2 | RCP2.6 | SSP2, eff. & ren. en., full CE: long life + reuse + recycling + light weighting, low wood | 3b, 4d+e, 5, 6a+b |
| SSP1_FullCE | SSP1 | RCP2.6 | SSP1, efficiency and ren. energy, full CE, low wood | 3b, 4c+d+e, 5 |
| LEMD_FullCE | LED | RCP2.6 | Low material and energy demand (LEMD), efficiency and ren. energy, Full CE, low wood | 3b, 4d+e, 5, 6a |
| **Sensitivity scenarios** | **SSP** | **RCP** | **Description** | **Figures** |
| SSP2_Light | SSP2 | RCP2.6 | SSP2 socioeconomics, efficiency and ren. energy, light-weighting, low wood | 3a |
| SSP2_Slow | SSP2 | RCP2.6 | SSP2 socioeconomics, efficiency and ren. energy, long lifetime, low wood | 3a, 4e |
| SSP2_Slow_Close | SSP2 | RCP2.6 | SSP2, efficiency and ren. energy, light-weighting + reuse + recycling, low wood | 3a, 4e |
| SSP2_Wood | SSP2 | RCP2.6 | SSP2 socioeconomics, efficiency and ren. energy, no CE, high wood | 3a, 6b |
| SSP2_FullCE_Wood | SSP2 | RCP2.6 | SSP2, efficiency and ren. energy, Full CE, ‘Wood’: high wood intensity in new buildings | 6a+b |
| LEMD_Light | LED | RCP2.6 | LEMD socioeconomics, efficiency and ren. energy, light-weighting, low wood | 3a |
| LEMD_FullCE_Wood | LED | RCP2.6 | LEMD, efficiency and ren. energy, Full CE, ‘Wood’: high wood intensity in new buildings | 3a+b, 6a |
| SSP2_Fossil | SSP2 | NoClimPol | SSP2 socioeconomics, fossil-intensive reference, no CE, low wood | 4a+d, 5, 6a+b |
| SSP2_Fossil_FullCE | SSP2 | NoClimPol | SSP2 socioeconomics, fossil-intensive reference, full CE, low wood | 4d, 6b |
| SSP2_Fossil_Wood | SSP2 | NoClimPol | SSP2 socioeconomics, fossil-intensive reference, no CE, wood-intensive buildings | 6a+b |
| SSP2_Fossil_FullCE_Wood | SSP2 | NoClimPol | SSP2, fossil-intensive reference, Full CE, ‘Wood’: high wood intensity in new buildings | 6b |
| LEMD_Fossil | LED | NoClimPol | LEMD socioeconomics, fossil-intensive reference, no CE, low wood | 4b+d, 6a |
| LEMD_Fossil_FullCE | LED | NoClimPol | LEMD socioeconomics, fossil-intensive reference, full CE, low wood | 4d |
| LEMD_Fossil_Wood | LED | NoClimPol | LEMD socioeconomics, fossil-intensive reference, no CE, wood-intensive buildings | 6a |
| SSP1_Fossil | SSP1 | NoClimPol | SSP1 socioeconomics, fossil-intensive reference, no CE, low wood | 4d |
| SSP1_Fossil_FullCE | SSP1 | NoClimPol | SSP1 socioeconomics, fossil-intensive reference, full CE, low wood | 4d |

**Traceability of results:**

Here, we document five major data processing and results reproducibility steps:

**1. Ensure the original data used can be found and retrieved, by linking data to their original sources:**

This requirement is met by (1) listing all references to raw data in the different parameter files and (2) compiling hard to obtain data files, conversion scripts, and conversion workbooks in an internal raw data archive.

The RECC v2.5 global buildings study database consists of 121 model parameters, each stored in a separate Excel workbook, and each with a separate log sheet, documenting all data processing that happened to compile the parameter data into the model format and a *reference sheet*, documenting all data sources to that each data source used is documented for each parameter.

For some input data, additional data sources and conversion scripts are needed, and these are documented and archived in the RECC 2.5 global building *raw data archive*, the research data archive of the Industrial Ecology group in Freiburg, hosted on the network drives by the university’s IT centre. The material in this database is less structured than the information in the parameter workbooks. It is available upon request.

**2. Link processed data to original data, assumptions, and a documentation of the data conversion process**

This requirement is met by documenting the different data processes that include the extraction, aggregation/disaggregation, and reformatting of data from the original sources into the RECC model parameter workbooks.

The data processes are documented along with the reformatted data, using separate sheets in the parameter files. Each of the 121 model parameter files comes with a separate log sheet, documenting all data processing that happened to compile the parameter data into the model format and a reference sheet, documenting all data sources. Each individual number can be traced back to the *data process* during which the values from the original sources were extracted, aggregated or disaggregated, and reformatted to the shape of the data given by the parameter file. Here, a new format, the ODYM data process, was developed that links each individual number to a detailed and reproducible description of how the data were converted from the original sources to the parameter file.

**3. Combine model parameters into a database and version-manage your project’s database**

This requirement is met by (1) compiling and archiving the entire model database (121 parameter files) into a single folder and (2) by specifying for each parameter its name and version number.

The collection of model parameter files that is needed to run the model is archived as model input database. The entire model input database for the RECC v2.5 global buildings study is archived on Zenodo under <https://zenodo.org/records/12752350>. The database consists of 121 model parameters, each with individual version numbers, stored in a separate Excel workbook, and each with a separate log sheet (see above for details), documenting all data processing that happened to compile the parameter data into the model format and a reference sheet, documenting all data sources.

All 70 model runs (7 CE/material choice scenarios for 10 global regions) were run with the same model input database available from Zenodo: <https://zenodo.org/records/12752350>

**4. Document each relevant calculation / model run so that model or assessment results can be linked to specific versions of the input data and to specific tools, so that results can be reproduced**

This requirement is met by (1) documenting for each model run the combination of model version and database version used to calculate results, and (2) by compiling the input information (which data and model configuration is used) and the results of each model run in a separate folder.

The model run for this paper was carried out on March. 8^th^, 2024 and can be reproduced by using and linking the following elements:

- The RECC v2.5 model input database for the global buildings study, as archived on Zenodo under <https://zenodo.org/records/12752350>.
- The RECC model as available on Github, with the following commit for model run: db05fdd, available via <https://github.com/IndEcol/RECC-ODYM>
- The results were generated by running the script RECC_ScenarioControl.py with the setting in line 25 to read the list of region + scenario combinations from the sheet “Buildings_Global_Config_list” of the scenario definition workbook “RECC_ModelConfig_List.xlsx”, which is part of the RECC v2.5 global building study database archive. This script calls the main model script for each scenario run.

For details on how to run the RECC model, please see section 7 of the RECC v2.5 model documentation available under <https://doi.org/10.6094/UNIFR/242061>.

For each RECC model run, the script ODYM_RECC_ScenarioControl.py writes the model configuration details defined in the scenario definition workbook “RECC_ModelConfig_List.xlsx” to the RECC model config file, which also contains a list of all model parameters and their version numbers. The results of each model run are archived in a separate folder, which includes a copy of the config file used.

**5. Make final results in reports and papers traceable by linking them to a specific model run and input database**

This requirement is met by (1) providing a list of where all numerical values mentioned in the paper are taken from, (2) providing a list of exactly what model result indicators are plotted in each figure or reported in each table and for what scenario, (3) providing a description of how the figures and tables were generated and compiled.

The lists mentioned under points (1) and (2) are provided below in the sub-section “Additional documentation of links between model results and indicators presented in the paper”.

The full model results (70 folders, plus the summary file RECCv2.5_EXPORT_Combine_Select.xlsx) are archived on Zenodo: <https://zenodo.org/records/12752350> All figures, tables, and numerical values presented in the paper and the different supplements have been generated/compiled from these results:

- The generation of the standard and paper-specific plots and tables, generated by calling the following scripts from the RECC model from the following commit ID: db05fdd, available via <https://github.com/IndEcol/RECC-ODYM>
  - ODYM_RECC_Export_xlxs_Combine_Select.py with setting “Buildings_Global_Config_list” in the result compilation workbook RECCv2.5_EXPORT_Combine_Select.xlsx
  - RECC_JIE_Global_Building_Plots.py
  - RECC_ESC_Plot_v1.py

The script RECC_Combine_Select.py generated a compilation of selected results across multiple scenarios in a single excel workbook ‘Results_Extracted_RECCv2.5_10Regs_sep.xlsx’, which is also part of the result archive.

- The two plotting scripts generate a larger number of plots for different indicators and regions, some of which are compiled into the figures presented in the paper and most of which are compiled into the supplementary figure compilation. The details are listed under point ‘List of where all numerical values mentioned in the paper and the supplementary material are taken from’ below.

For details on how to run the RECC model, please see section 7 of the RECC v2.5 model documentation available under <https://doi.org/10.6094/UNIFR/242061>.

**Additional documentation of links between model results and indicators presented in the paper:**

**List of exactly what model result indicators are plotted in each figure or reported in each table and for what scenario:**

Table S8 below lists the names of the raw plots and the displayed model result indicators for the chosen scenarios and regions. The raw plots are the ones generated from the result compilation file ‘RECCv2.5_EXPORT_Combine_Select.xlsx’ by the scripts ‘RECC_JIE_Global_Building_Plots.py’ and ‘RECC_ESC_Plot_v1.py’. The indicators are labelled as in the result compilation file and not as in the RECC results files, where longer and more descriptive names are sometimes used. The correspondence between the two label lists is given in the result compilation workbook RECCv2.5_EXPORT_Combine_Select.xlsx, sheet ‘Export_GlobalBld_Figs’, section ‘Indicator list’.

**Table S8:** Overview of which indicators, scenarios, and regions are plotted from which figures generated by the plotting scripts. All plots are archived under the study’s result archive on Zenodo, see link above. The supplementary plots are available as pdf in the figure supplement or as individual figures in the Zenodo archive.

| **Figure** | **Name of raw plot(s)** | **Indicators (I), scenarios (S), regions (R) plotted** |
| --- | --- | --- |
| Fig. 2a+b,  Fig. SP5 | Stock_Growth_Global_reb.png  Stock_Growth_Global_nrb.png  stock pattern_reb_Global.png  stock pattern_nrb_Global.png | **I:** In-use stock, res. Buildings (same for non-res blds).  final consumption (use phase inflow), all res. building types together (same for non-res. blds.)  Stock curve of all pre 2021 age-cohorts, res. blds.  decommissioned buildings (use phase outflow), all res. building types together (same for non-res. blds.)  **S:** stacked plots: SSP1_Base;LEMD_Base;SSP1_Base;SSP2_Base  For stock area and outflow plots: SSP2_Base;SSP1_FullCE;LEMD_FullCE  **R:** All ten plus global aggregate |
| Fig. 3a,  Fig. SP8 | Material_Flows_  Cumulative_Global_2020-50.png | **I:** Inflows: Final consumption of materials: cement','Final consumption of materials: construction grade steel','Final consumption of materials: wood and wood products'  Outflows: 'Outflow of materials from use phase, cement','Outflow of materials from use phase, construction grade steel','Outflow of materials from use phase, wood and wood products'  Recycled+Reused: 'ReUse of materials in products, concrete','ReUse of materials in products, construction grade steel','ReUse of materials in products, wood and wood products', 'Potential for secondary construction steel from EoL products'  **S:** LEMD_Light;SSP2_Light;SSP2_Base;SSP2_Wood;SSP2_Slow_Close; SSP2_Slow;SSP2_Base;LEMD_FullCE_Wood  **R:** Global aggregate |
| Fig. 3b | Demand_Primary_Production.png | **I:** Cement production', 'Primary steel production', 'Construction wood, structural, from industrial roundwood  **S:** SSP2_Base;SSP2_FullCE;SSP1_FullCE;LEMD_FullCE;  LEMD_FullCE_Wood  **R:** All ten countries and regions |
| Fig. 4 a+b, Fig. SP9 | Energy_Cons_Base_Global.png  Energy_Cons_LEMD_Global.png | **I:** energy consumption, use phase, reb: electricity;energy consumption, use phase, reb: coal, hard coal;energy consumption, use phase, reb: heating oil;energy consumption, use phase, reb: natural gas;energy consumption, use phase, reb: hydrogen;energy consumption, use phase, reb: fuel wood;energy consumption, use phase, nrb: electricity;energy consumption, use phase, nrb: coal, hard coal;energy consumption, use phase, nrb: heating oil;energy consumption, use phase, nrb: natural gas;energy consumption, use phase, nrb: hydrogen;energy consumption, use phase, nrb: fuel wood;  **S:** SSP2_Fossil;SSP2_Base;SSP1_Fossil;SSP1_Base;LEMD_Fossil; LEMD_FullCE  **R:** All ten countries and regions plus the global aggregate |
| Fig. 4c, Fig. SP10 | GHG_Stacked_Global.png | **I:** 'GHG emissions, buildings, use phase','GHG emissions, use phase scope 2 (electricity)','GHG emissions, use phase other indirect (non-el.)','GHG emissions, primary material production','GHG emissions, manufact, wast mgt., remelting and indirect','GHG sequestration by forests (w. neg. sign)','GHG emissions, system-wide (incl. forests)'  **S:** SSP2_Fossil;SSP2_Base;SSP1_Fossil;SSP1_Base;LEMD_Fossil; LEMD_FullCE  **R:** All ten countries and regions plus the global aggregate |
| Fig. 4d,  Fig. SP11 | GHG_by_CE_Range_Global_Combined.png | **I:** GHG emissions, system-wide (excl. forests)  **S:** SSP2_Fossil_FullCE;SSP2_Fossil;SSP1_Fossil;SSP1_Fossil_FullCE; LEMD_Fossil;LEMD_Fossil_FullCE;SSP2_Base;SSP2_FullCE;SSP1_Base;  SSP1_FullCE;LEMD_Base;LEMD_FullCE  **R:** All ten countries and regions plus the global aggregate |
| Fig. 4e, Fig. SP13 | CascadeGHG, system-wide, cum. 2020-50_Global.png | **I:** GHG emissions, system-wide (excl. forests);GHG emissions, buildings, use phase;GHG emissions, res+non-res buildings, energy supply;GHG emissions, primary material production  **S:** SSP2_Base;SSP2_Slow;SSP2_Slow_Close;SSP2_FullCE;SSP1_FullCE;  LEMD_FullCE  **R:** All ten countries and regions plus the global aggregate |
| Fig. SP12 | CascadeGHG, system-wide, 2050_Global.png | **I:** GHG emissions, system-wide (excl. forests); GHG emissions, buildings, use phase; GHG emissions, res+non-res buildings, energy supply; GHG emissions, primary material production  **S:** SSP2_Base;SSP2_Slow;SSP2_Slow_Close;SSP2_FullCE;SSP1_FullCE;  LEMD_FullCE  **R:** All ten countries and regions plus the global aggregate |
| Fig. 5, Fig. SP14 | Energy service cascade_GHG_Global.png | **I:** In-use stock, res. buildings; In-use stock, nonres. buildings, Energy cons., use phase, res+non-res buildings, GHG emissions, res. buildings, use phase, GHG emissions, non-res. buildings, use phase, GHG emissions, res+non-res buildings, energy supply, final consumption (use phase inflow), all res. building types together, final consumption (use phase inflow), all nonres. building types together, Final consumption of materials, GHG emissions, primary material production  **S:** SSP2_Fossil;SSP2_Base;SSP2_FullCE;SSP1_FullCE;LEMD_FullCE  **R:** All ten countries and regions plus the global aggregate |
| Fig. 6a | LEMDInd.png | **I:** Cement production','Primary steel production','GHG emissions, non-biogenic, In-use stock, res. buildings','In-use stock, nonres. buildings','Population’  **S:** SSP2_Fossil;LEMD_Fossil;SSP2_Fossil_Wood;LEMD_Fossil_Wood; SSP2_FullCE;LEMD_FullCE;SSP2_FullCE_Wood;LEMD_FullCE_Wood  **R:** All ten countries and regions plus the global aggregate |
| Fig. 6b | WoodSubstInd.png | **I:** Construction wood, structural, from industrial roundwood','Cement production','Primary steel production','GHG emissions, primary material production','GHG emissions, non-biogenic'  **S:** SSP2_Fossil_Wood;SSP2_Fossil;SSP2_Fossil_FullCE_Wood; SSP2_Fossil_FullCE;SSP2_Wood;SSP2_Base;SSP2_FullCE_Wood; SSP2_FullCE  **R:** All ten countries and regions plus the global aggregate |
| Fig. SP6 | Material_Flow_reb_nrb_Global.png | **I:** Final consumption of materials: cement;Final consumption of materials: construction grade steel;Final consumption of materials: wood and wood products  **S:** SSP2_Base;SSP2_FullCE;SSP2_FullCE_Wood;LEMD_Fossil; LEMD_FullCE; LEMD_FullCE_Wood  **R:** All ten countries and regions plus the global aggregate |
| Fig. SP7 | Material_Stock_reb_Global.png  Material_Stock_nrb_Global.png | **I:** In-use stock, cement;In-use stock, construction grade steel;In-use stock, wood and wood products  **S:** SSP2_Base;SSP2_FullCE;SSP2_FullCE_Wood;LEMD_Fossil; LEMD_FullCE;LEMD_FullCE_Wood  **R:** All ten countries and regions plus the global aggregate |
| Fig. SP15 | Energy service cascade_RMI_Global.png | **I:** In-use stock, res. buildings; In-use stock, nonres. buildings, final consumption (use phase inflow), all res. building types together, final consumption (use phase inflow), all nonres. building types together, Final consumption of materials, Material footprint, metal ores, system-wide, Material footprint, non-metallic minerals, system-wide, Material footprint, biomass (dry weight), system-wide  **S:** SSP2_Fossil;SSP2_Base;SSP2_FullCE;SSP1_FullCE;LEMD_FullCE  **R:** All ten countries and regions plus the global aggregate |
| Fig. SP16 | See section “Sankey diagram export” below at the bottom of this document for details. | See section “Sankey diagram export” below at the bottom of this document for details. |
| Fig. SP17 | Floorspace by building type,  FloorSpace_Stock_Type_reb_Global.png | **I:** In-use stock, reb, informal;In-use stock, reb, SFH;In-use stock, reb, MFH;In-use stock, reb, RT; same for nrb  **S:** LEMD_Fossil;SSP2_Fossil;LEMD_Base;SSP2_Base  **R:** All ten countries and regions plus the global aggregate |
| Fig. SP18 | Floorspace by energy standard,  FloorSpace_Stock_EnergyStandard_reb_Global.png | **I:** In-use stock, reb, no_standard;In-use stock, reb, standard;In-use stock, reb, efficient;In-use stock, reb, ZEB; same for nrb  **S:** LEMD_Fossil;SSP2_Fossil;LEMD_Base;SSP2_Base  **R:** All ten countries and regions plus the global aggregate |

The energy service cascade embeds the stock-flow-service nexus in a larger framework of linking wellbeing to environmental impacts (Fig. S2). To study decoupling in the different steps of the cascade, we focus on the link between building services and GHG pressure in the cascade and study to IPAT-Style decomposition equations, one for energy-related GHG per capita (eq. 1, scope 1+2) and one for material-related GHG (eq. 2, scope 3):


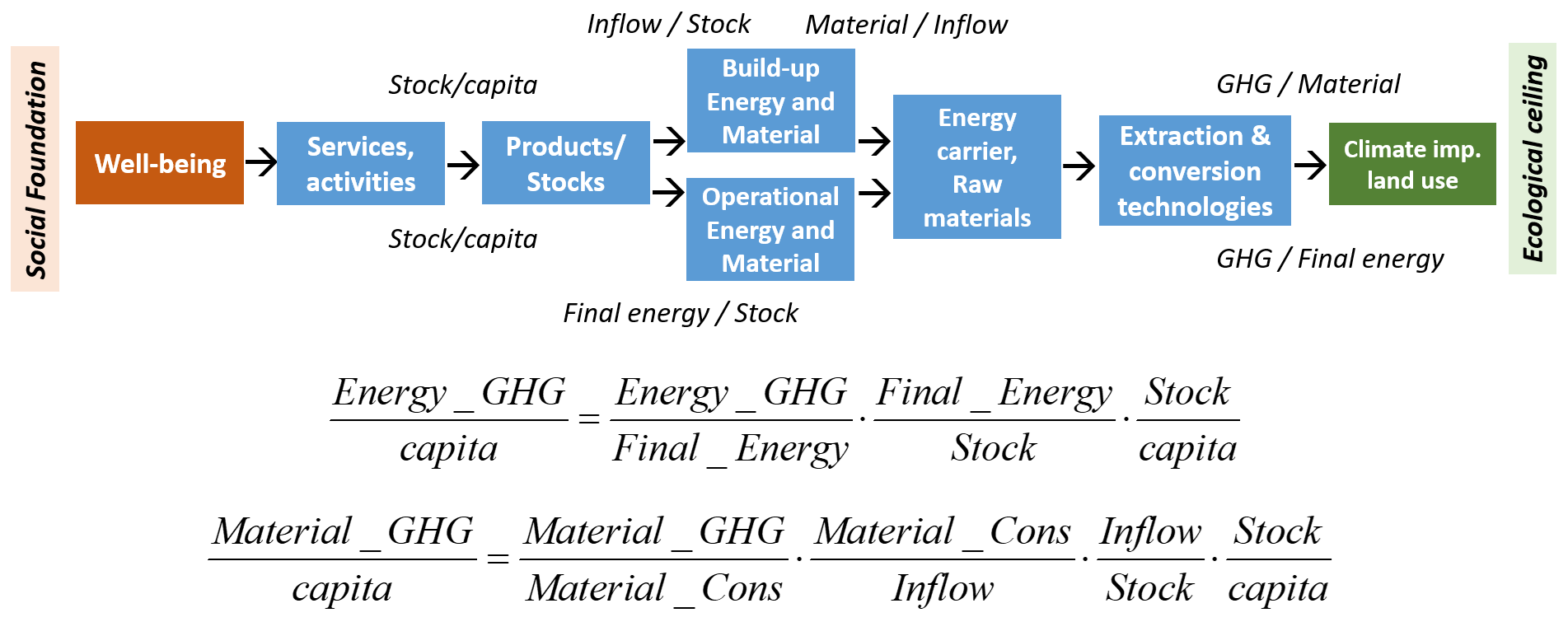


**Fig S2:** Indicator definition in the energy service cascade.

Numerical results are shown in Fig. 5 in the paper and in the figure supplement.

**List of all mentions of numerical values in the paper and the supplementary material and their origin** (references appear in the main paper and are not repeated here)

***Abstract and Introduction:*** *“buildings cause 30% of final energy consumption, 18% of greenhouse gas (GHG) emissions, and about 65% of material consumption globally.”*

🡪 The basic energy flows (133 EJ/yr for buildings and 442 EJ/yr for the global total final energy consumption in 2022), leading to a share of buildings of 30%, are reported by the IEA WEO 2023 at several places, e.g., in section ‘3.3 Total final energy consumption’ on p107.

The 18% were rounded up from the reported 17.5% share of GHG associated with energy use in buildings in 2016, compiled by Ritchie (2020). This figure refers to scope 1+2 only. Scope 3 emissions related to material production for buildings are not included here.

The 65% for material consumption are the rounded ratio of steel and concrete going into residential and non-residential buildings over the total final consumption of stock-building materials in 2016.

All figures have global scope.

*“The 2020-50 global cumulative new construction ranges from 150 to 280 billion m² for residential and 70-120 billion m² for non-residential buildings.”* 🡪 Read from Fig. 2a+b, left.

*“Ambitious CE reduces cumulative 2020-2050 primary material demand from 80 to 30 gigatons (Gt) for cement and from 35 to 15 Gt for steel.”* 🡪 Read from Fig. 3b, indicating the range from SSP2_Base to LEMD_FullCE, excluding the wood-intensive scenario, since that would require additional explanation for which there is no space in the abstract.

*“Lower floor spaces of 1 m² less per capita lead to global savings of 800-2500 Mt of cement, 300-1000 Mt of steel, and 3-10 Gt CO2-eq, depending on industry decarbonization and CE roll-out.” “Each additional Mt of structural timber leads to savings of 0.4 0.55 Mt of cement, 0.6 0.85 Mt of steel, and 0.8 1.8 Mt of system-wide GHG emissions.”* 🡪 Read from Fig. 6 and taken from the results section.

*“CE reduces 2020-2050 cumulative GHG by up to 44%, …”* 🡪 Read from Fig. 4e.

***Introduction:*** *“Buildings are associated with 30% of global final energy consumption (IEA, 2023), 18% of greenhouse gas (GHG) emissions (Ritchie, 2020), and about 65% of stock-accumulating material consumption (Wiedenhofer et al., 2024) globally.”* 🡪 See above for ‘abstract’, same statement.

*“Zhong et al. (2021) study the material implications of the global building stock and report baseline (SSP2) material-related GHG between 3.6 and 4.8 Gt CO2-eq/yr for 2020-2060, with a 2020-2060 cumulative GHG reduction potential of lower floorspace of 55 Gt, 14 Gt for light-weight design, and about 6 Gt CO2-eq for lifetime extension and better material recovery, respectively.”* 🡪 Total material GHG read from their figure 1a, single strategy contribution from their figure 2. Numbers are approximate values from visual inspection of the figures.

*“Mishra et al. (2022) estimate that housing 90% of the new urban population globally in wood-based urban mid-rise buildings could save 106 Gt CO2 by 2100, supplied by up to 150 Mha of new forest plantations.”* 🡪 Numbers taken from their abstract.

***Results:*** *“Due to the long building lifetime, there is a substantial lock-in (grey area in the center plots in Fig. 2), with more than half of the 2020 residential buildings still standing in 2050 (more than 1/3 for non-residential buildings), which underpins the need for deep building retrofits to reduce energy demand. Still, with current building lifetimes, globally, around 60% (residential) and 75% (non-residential) buildings that stood in 2020 will have to be re-built by 2060.” “The 2020-50 global cumulative new construction of residential buildings ranges between 150 and 280 billion m² (70...120 billion m² for nonresidential buildings), with total demolition volumes of around 100 billion m² (residential) and around 35 billion m² (non-residential) during the same time range.”* 🡪 Visual inspection of Figure 2.

*“Cumulative 2020-50 material demand for cement, steel, and wood amounts to about 130 Gt for the SSP2 scenario, and substantial lowering can be achieved by lightweight building design and substantially lower floorspace (Fig. 3a, top left), a substantial shift to wooden buildings (top right), and a combination of all strategies (bottom right), with a total reduction potential of major material demand of 60%.”* 🡪 Read from Fig. 3a, visual inspection.

*“In a lower floorspace world with light-weighted, long-lived, and wood-intensive buildings (LEMD_FullCE_Wood), cumulative cement demand could be less than 25% of the baseline figures, saving about 60 Gt of cement and 350 billion tons of aggregate extraction during 30 years.”* 🡪 Read from Fig. 3, the delta between the largest and the smallest grey bar for cement is about 60 Gt, and with a typical cement content of concrete of 15%, the total mass of this saved concrete would be 400 Gt, of which 60 Gt is cement and 340 Gt is aggregates, which is reported as rounded figure (350 Gt) in this statement.

*“in the RCP2.6-compatible energy supply, electricity will account for 80-90% of final energy demand by buildings”* 🡪 Read from Fig. 4a+b, visual inspection.

*“System-wide GHG emissions for SSP1_Base decline to about 1/3 between 2020 and 2060 (Fig. 4c, orange in Fig. 4d), with future levels much lower than a baseline without new climate policy (medium grey in Fig. 4d). Higher reductions down to around 2.5 Gt CO_2_-eq/yr in 2060 (without forest sequestration or CCS) are only possible…”* 🡪 Read from Fig. 4c, visual inspection, and comparison with ranges shown in Fig. 4d.

*“With moderate emissions mitigation in SSP2_Base, 2020-2050 cumulative GHG from the building sector will account for almost the entire remaining emissions budget for the 1.5°C and about 1/3 of the remaining budget of the 2°C target (Fig 4e).” and “the combination of supply and demand-side and CE measures can reduce system-wide 2020-50 cumulative GHG emissions by 44%”* 🡪 Read from Fig. 4e, putting in relation the total reported cumulative GHG (ca. 350 Gt) to the reported budget figures of 400 and 1150 Gt for 1.5°C and 2°C, respectively.

*“Targeting lower floor spaces of 1 m² less per capita in 2050 will lead to global savings of 800…2500 Mt of cement, 300…1000 Mt of steel, and 3…10 Gt of GHG, depending on the degree of industry decarbonization and CE roll-out”* 🡪 Read from Fig. 6a, visual inspection

*“When ignoring carbon pool changes in forests, using 1 additional Mt of structural timber leads to average savings of 0.4-0.55 Mt of cement, 0.6-0.85 Mt of steel, and 0.8-1.8 Mt of system-wide GHG emissions”* 🡪 Read from Fig. 6b, visual inspection

***Discussion:*** *“Overall, there is a substantial mitigation potential, up to 44% of cumulative 2020-50 global sectoral GHG”* 🡪 Number directly shown in Fig. 4e.

*“could reduce cumulative 2020-50 cement and primary steel demand by about 2/3”* 🡪 Approximate range shown in Fig. 3b.

*“We find substantially higher building material-related GHG (about 3.4/4.4/6.1 Gt CO2-eq/yr in 2020 for LEMD/SSP1/SSP2 vs. 3.6 Gt by Zhong et al. for SSP2), which is due to the larger floorspace and the higher material demand for stock expansion in different regions: we find 1.1…1.8 Gt CO2-eq/yr from China alone, largely driven by cement demand.”* 🡪 Our numbers read from, Zhong et al. number read from their Fig. 1a (approximate value for 2020).

*“Mishra et al. (2022) report a rising trend in material-related GHG for new buildings (their figure 3b.2, slope of cumulative emissions is rising), which is the opposite of what a dynamic stock model with increasing recycling and decarbonisation yields (our fig. 4c). This difference in the trend is the result of the stylistic representation of building stock dynamics by Mishra et al. They report cumulative 2020-50 cement and steel-related GHG for residential buildings of only 5 Gt CO2 max, which is clearly not enough given global stock expansion envisioned for SSP2.”* 🡪 Directly read from Fig. 3b.2 in Mishra et al.

*“Our final energy consumption results for residential buildings start at levels about 60% higher than those reported by Mastrucci et al. (2021) and remain at high levels in SSP2, because we include domestic hot water along with heating and cooling and calibrated against reported energy use.”* 🡪 Our staring value (Fig 4a, top left, for 2016) is about 43 EJ/yr, while Mastrucci et al. (2021) report about 27 EJ/yr (25 for heating and 2 for cooling) in their Fig 6A (left side for World).

*“Mastrucci and van Ruijven (2023) study future global residential floorspace levels between 200 and 300 billion m², while this study covers a yield a range of 250…400 billion m².”* 🡪 Our number taken from the range shown in Fig. 2a (left), Mastrucci and van Ruijven number directly taken from their Figure 1.

*“the world is not on an SSP2 track for building material stock expansion, because actual construction material production and apparent consumption by buildings is lower than the projected SSP2 values of around 6 Gt CO_2_-eq/yr for building materials alone”*  🡪 Read from the supplementary figure SP10 (GHG time series by sector and region), visual inspection, page 59 of 89, figure “GHG_Stacked_SSP2_Global”, representative value for the years 2016-2025.

*“For the SSP2 scenario, cumulative 2020-2050 industrial roundwood harvest demand ranges between 16 (standard design) and 61 (wood-intensive design) Gt of C. In the LEMD scenario, the cumulative wood demand is about half as much, ranging from 8 (standard design) and 32 (wood-intensive design) Gt of C (flow F1_2).”* 🡪 Numbers on demand for industrial roundwood in Mt/yr of Carbon are driven by demand for structural timber and include wood for other applications (cascading) and fuel food (residues) only as a byproduct from the main output sawnwood for structural timber. The values for F_1_2_ are taken from the main result compilation table: Results_Extracted_RECCv2.5_10Regs_sep.xlsx”, sheet “Results_Cumulative”, indicator “Demand for industrial roundwood, hard and softwood” for the following scenarios:

LEMD_Base: 8.3 Gt of C for the 2020-50 cumulative flow.

SSP2_Base: 16.1 Gt of C for the 2020-50 cumulative flow.

LEMD_FullCE_Wood: 31.6 Gt of C for the 2020-50 cumulative flow.

SSP2_Wood: 61.3 Gt of C for the 2020-50 cumulative flow.

*”For the wood-intensive building scenarios, cumulative 2020-50 construction wood demand is between 3 (LEMD) and 6 (SSP2) times larger than the volumes reported by Mishra et al. (2022), who focus on urban residential expansion to relatively low m²/cap values only and do not include the transformation of the entire building stock.”*

🡪 The RECC values for F_3_4_ are taken from the main result compilation table “Results_Extracted_RECCv2.5_10Regs_sep.xlsx”, sheet “Results_Cumulative”, indicator “Demand for industrial roundwood, hard and softwood” for the following scenarios:

LEMD_Base: 8.3 Gt of C for the 2020-50 cumulative flow.

SSP2_Base: 16.4 Gt of C for the 2020-50 cumulative flow.

LEMD_FullCE_Wood: 30.7 Gt of C for the 2020-50 cumulative flow.

SSP2_Wood: 60.3 Gt of C for the 2020-50 cumulative flow.

In their supplementary figure 1, “Supplementary Figure 1: Estimated engineered wood demand”, Mishra et al. (2022) report the global engineered wood demand for the low wood, medium wood, and wood-intensive scenarios (10, 50, or 90% o new urban buildings are wooden buildings). Accumulating their results for the 2020-2050 period roughly leads to the following numbers, using an average density of 0.5 for mostly dry construction wood:

Low-wood (10%): 2250 Mm³ or 1.1 Gt engineered wood for 2020-2050.

Medium-wood (50%): 11500 Mm³ or 5.8 Gt engineered wood for 2020-2050.

High-wood (90%): 20300 Mm³ or 10.1 Gt engineered wood for 2020-2050.

This gives the ratios of SSP2_Wood/High-wood of 6 and for LEMD_FullCE_Wood to High-wood of 3.

*“**When comparing our results to recent extraction statistics and the scenarios reported by Johnston and Radeloff (2019),* *we see that without massive changes in wood supply, only the low-wood scenarios’ demand can be met in most world regions.”* 🡪 In their figure 1A, Johnston and Radeloff (2019) report recent global industrial roundwood harvest volumes of around 1.8 billion m³/yr and project a mid-century increase for SSP2 to around 2.4 billion m³/yr, resulting in 2020-50 harvest volumes of around 54 billion m³ (for recent value kept constant) and around 70 billion m³ (rough integration of growth curve), with a carbon content of around 0.23 Mg C/m³ (Zhang u. a. 2019), this amounts to 12…16 Gt of C, which roughly matches the LEMD_Base and SSP2_Base demand.

*“The wood-intensive scenarios for the entire building stock are clearly infeasible with current harvest rates in most regions, they would require 4-5 times (SSP2) and 2-3 times (LEMD) higher harvest volumes than projected by Johnston and Radeloff (2019).”* 🡪 Derived from comparing the above-reported results for LEMD_FullCE_Wood (31 Gt C) and SSP2_Wood (60 Gt C) with the projected supply (12…16 Gt C) and calculating their approx. ratios.

“Since the conversion rate from roundwood into structural timber in the wood processing industry varies between 30 and 70%” 🡪 Range of possible values for structural timber from industrial roundwood (process yield factor of the sawmills), see the *log* and *ref* sheets of RECC model parameter 4_PY_TimberRoundWood_V1.1.xlsx for details, archived under <https://zenodo.org/records/12752350>.

**Part 2: RECC v2.5 model and database change log compared to RECC v2.5 documentation**

The text below documents changes in relation to the RECC v2.5 model documentation (<https://doi.org/10.6094/UNIFR/242061>) and the v2.5 base database used as reference for this model documentation. The data sources and data processing for the individual parameter files are documented on the log sheet of each parameter file.

**2.1. Changes to the database compared to RECC v2.5 base version**

Overview only. For details, see the respective parameter files. Latest config file in \Dropbox\RECC_v2_5_v2_6\data\CURRENT_V2_5\RECC_Config.xlsx, sheet ‘Buildings_Global’

- Add recycling of concrete aggregates and steel recycling, use reference values from Switzerland as target: 4_PY_MaterialProductionRemelting_V2.5, also 4_PY_EoL_RecoveryRate_V2.6 and 6_PR_EoL_RR_Improvement_V2.4
- Move full CE implementation from 2040 to 2035 with new parameter 3_SHA_RECC_REStrategyScaleUp_V3.4
- Added RCP aspect to 3_SHA_TypeSplit_Buildings_V2.0 and 3_SHA_TypeSplit_NonResBuildings_V3.0. Make clearer distinction between the share of efficient buildings (focus of current climate policy) and of ZEB (focus of RCP 2.6 climate policy).
- 3_SHA_EnergyCarrierSplit_Buildings has hardly any change between NoClimPol and RCP2.6! Changes to: 3_SHA_EnergyCarrierSplit_Buildings_V3.1 and 3_SHA_EnergyCarrierSplit_NonResBuildings_V2.1. Use data from Table 2.1 and Figure 2.11 in IEA 2019: <https://www.iea.org/reports/the-critical-role-of-buildings> as well as detailed results from the IEA WEO 2021 and 2022.
- Low Wood building archetypes: In the new default parameters 3_MC_BuildingArchetypes_V2.1_LowWood and 3_MC_NonResBuildingArchetypes_V2.1_LowWood, the difference in material content (kg/m²) between the hitherto used baseline design (which is a medium wood content building design), and the wood-intensive buildings, was applied in the other direction. Here, the difference from high wood to medium wood is subtracted from medium wood to obtain a low wood alternative to estimate the wood and concrete content of a low-wood alternative. This sensitivity analysis into a low-wood direction yielded plausible values all throughout building types and regions, and is now used as baseline, while the medium wood buildings can be chosen by using the old parameter versions and the high wood alternatives by setting the MSU flag to *True*.
- Add RCP aspect to res. and non-res. building downsizing and light-weighting share: Added RCP aspect to 3_SHA_DownSizing_Buildings, 3_SHA_LightWeighting_Buildings, 3_SHA_DownSizing_NonResBuildings, and 3_SHA_LightWeighting_NonResBuildings. Use LED values so far for all RCP2.6 scenarios, keep existing values for LED-SSP1-SSP2 for the baseline. Leads to larger model update, as both 3_MC and 3_IE parameters now have an R aspect all throughout the model!

**2.2. Model improvement and fixes compared to RECC v2.5 base version**

**Consistent accounting of biogenic CO_2_ emissions and of the effect of energy substitution from using internal wood waste. This affects the main model results for GHG by process only, not the RECC carbon cycle flows, which are already reported consistently.**

Until now, total GHG didn’t add up to total by sector, because we have energy savings from using internal wood waste for combustion and energy recovery (electricity generation). This leads to an actual reduced energy demand for process 15 and thus lower actual emissions.

We assume and implement the following substitution of wood waste: First fuel wood, then natural gas, then electricity.

Now, these emissions savings are subtracted from the indirect building sector emissions (SysExt_Impacts_UsePhase_7i_Scope2_El and SysExt_Impacts_UsePhase_7i_OtherIndir) as most of the wood waste is used here to generate heat (substitutes natural gas) or electricity.

We added the wood waste C emissions to the system-wide emissions in the waste management sector, but only the fraction that is not already accounted for as use phase carbon emissions from fuel wood use.

**These specifications are summarized in the following accounting and modelling principles for biogenic CO_2_ and emissions from the burning of waste wood for energy recovery (WtE):**

- Emissions are accounted for where they occur, i.e., the process in which they occur. In particular, the CO_2_ emissions resulting from the burning of waste wood for energy recovery (WtE) are accounted for as direct emissions of the waste management sector (variable SysVar_WtE_CO2_xt).
- Emissions reductions due to energy savings by using internal wood waste are calculated before the GHG breakdown is established.
- Emissions from final energy supply (process 15, without WtE) are reported as total, after subtracting the energy demand that is covered by WtE. They are also broken down by consuming sector and attributed as indirect (scope 2) emissions of that sector (indicated by the subscript ‘i’). Electricity-related GHG are reported separately from other energy carriers’ supply emissions.
- All emissions, including biogenic CO_2_, are included in the sector-specific emissions breakdown.
- Wood from forests enters the system as carbon flow F_1_2, and, depending on the forest growth model chosen, there is also a carbon sequestration by forests, F_0_1, which is added to the total system emissions Impacts_System_13579di but not to Impacts_System_3579di.
- Biogenic C leading to biogenic CO_2_ emissions are reported separately in three categories: SysVar_WoodWasteIncineration (biogenic C released from the use phase (structural timber and cascade) as well as waste wood from processing industrial roundwood), Carbon_Fuelwood_bld (carbon in fuelwood for use in buildings), and Carbon_Fuelwood_el (carbon in fuelwood for use in electricity generation).

**The new biogenic carbon accounting leads to the following breakdown of carbon flows and emissions scopes (Tables S9 and S10):**

**Table S9:** Overview of the different breakdowns for environmental extensions by process. The number indicates the process number in the RECC model system definition. The index ‘d’ stands for direct (fuel-related and process) emissions from the different processes, and the index ‘I’ stands for the indirect (energy-related) emissions and resource use (process 15: energy supply).

| **Impacts_System_3579di** | **SysExt_Impacts_UsePhase_7d**  (incl. fuelwood-related CO_2_ direct ems.) | **SysExt_Impacts_UsePhase_7d**  (incl. fuelwood-related CO_2_ direct ems.) |
| --- | --- | --- |
|  | **SysExt_Impacts_UsePhase_7i_Scope2_El** | **SysExt_Impacts_UsePhase_7i_Scope2_El** |
|  | **SysExt_Impacts_UsePhase_7i_OtherIndir** | **SysExt_Impacts_UsePhase_7i_OtherIndir** |
|  | **SysExt_Impacts_PrimaryMaterial_3di** | **SysExt_Impacts_PrimaryMaterial_3di** |
|  | **SysExt_Impacts_MaterialCycle_5di_9di**  (incl. WtE-related CO_2_ direct ems.) | **SysExt_Impacts_Manufacturing_5di** |
|  |  | **SysExt_Impacts_WasteMgtRemelting_9di**  (incl. WtE-related CO_2_ direct ems.) |

The fuelwood-related CO_2_ direct ems. are given by the indicator *Carbon_Fuelwood_bld*.

The WtE-related CO_2_ direct ems. are given by the indicator *SysVar_WoodWasteIncineration*.

The parameter *Carbon_Fuelwood_el* (carbon in fuelwood for use in electricity generation), denotes the biomass-related Carbon that is part of F_15_0 (impacts of energy supply). These energy supply-related GHG again are re-allocated as part of the indirect emissions ‘i’ of all other processes, and reported there according to the amount and type of energy carrier: see 3i, 5i, 7i, 9i in the table S9 above.

The total impacts of the system, including a simplified representation of forest carbon uptake, is given by:

*Impacts_System_13579di = Impacts_System_3579di + SysExt_CO2UptakeImpacts_Forests*

**Other indicators:**

**Table S10:** Overview of the different breakdown of biogenic carbon flows.

| **SysVar_WoodWasteIncineration**  **(Mt C / yr)** (All C in wood waste: immediate, end-of-life, and post-cascade. Part of total techno-sphere biogenic carbon release, the other part is the emissions from burning fuel wood (both buildings use and electricity gen.) | **SysVar_WoodWasteFuelWoodSubst_tr (Mt C / yr)** (Wood waste that substitutes fuel wood):  -> Reduce fuel wood demand from SysVar_EnergySupply_15_16 and from Carbon_Fuelwood_bld  -> Reduce the fuel wood demand from SysVar_Carbon_FuelWood_2_7_net_tr  -> Biogenic carbon from burning fuel wood are already part of use phase direct emissions. |
| --- | --- |
|  | **SysVar_WoodWaste_Gas_El (Mt C / yr)** (Wood waste for waste to energy (WtE)):  -> Carbon in wood waste for energy recovery  -> Part of waste mgt. (process 9) direct emissions |

**Impacts_Energy_Supply_All:** All energy supply related impacts associated with processes 15 (energy supply) and 9 (WtE), sums up all indirect (‘i’) contributions from the other processes. This number includes energy supply impacts AFTER subtracting the avoided impacts of reduced energy supply from process 15 by recovering heat and electricity from the incineration of system-internal waste wood (waste to energy WtE).

**Carbon cycle model and CRAFT model interface update (Fig. S2):**


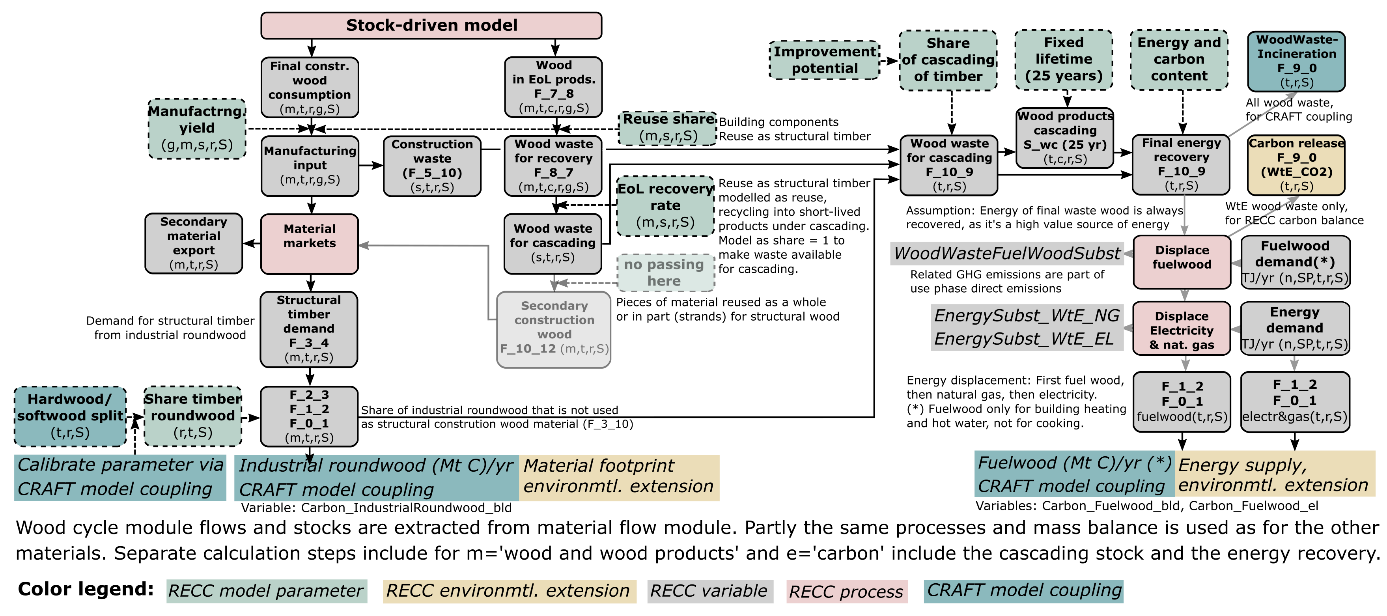


**Fig S3: Update of Fig. 6.6 in the RECC v2.5 model docu:** The RECC carbon cycle module. The module is part of the RECC model equations and included in the mass balance of the processes in the system definition for the chemical element carbon. The carbon cycle module was developed for coupling with the CRAFT model of biomass supply from forests (Le Noë et al. (2020): <https://doi.org/10.1111/gcb.15004>).

**Biogenic carbon flows from atmosphere to Forests, in Mt C / yr:**

- *SysExt_CO2UptakeImpacts_Forests,* part of F_0_1. Here, by setting the ‘ForestryModel’ flag in the config file, one can choose between a simple forest growth model (with two different lifetimes for construction wood/industrial roundwood and fuelwood, RECC v2.4 default) and the assumption of forestry with constant carbon pools (RECC v2.5 default). In the latter case, forestry with constant carbon pools is assumed at the landscape level, and the carbon flow in F_0_1 is equal to the one in F_1_2, which leads to a large net sequestration of forests, which is part of Impacts_System_13579di. This accounting is the default of building sector studies. For more detailed assessments RECC needs to be soft-coupled with a forest growth model, such as CRAFT at the country and global level.

**Biogenic carbon flows from CRAFT to RECC, in Mt C / yr:**

- *Carbon_IndustrialRoundwood_bld* (biogenic C in industrial roundwood), part of F_1_2,
- *Carbon_Fuelwood_bld* (carbon in fuelwood for use in buildings), part of F_1_2, and
- *Carbon_Fuelwood_el* (carbon in fuelwood for use in electricity generation), part of F_1_2.

**Biogenic carbon flows from RECC to the atmosphere, in Mt C / yr:**

- *SysVar_WoodWasteIncineration* (biogenic C released from the use phase (structural timber and cascade) as well as waste wood from processing industrial roundwood), part of F_9_0,
- *Carbon_Fuelwood_bld* (carbon in fuelwood for use in buildings), part of F_7_0, and
- *Carbon_Fuelwood_el* (carbon in fuelwood for use in electricity generation), part of F_15_0.

**RECC system (techno-sphere) impacts that are not related to biogenic carbon flows:**

As a good proxy for the non-biogenic GHG emissions affected by different levels of biomass use, the GHG impact of primary material production, *SysExt_Impacts_PrimaryMaterial_3di*, can be used, as this flow changes substantially when wood is substituted for steel and cement.

The complete (energy and material supply, direct emissions) extension variable that covers all flows not associated with the C flows from the forestry sector (covered by the CRAFT model) is:

- *SysExt_TotalImpacts_3579di – 44/12 * (SysVar_WoodWasteIncineration + Carbon_Fuelwood_bld + Carbon_Fuelwood_el)*

While the carbon flows are used to calculate the net climate impact of wood use, this quantity is used to calculate the avoided impacts by increase wood use.

**Biogenic C flows in exported RECC system variables:**

- *SysVar_WoodWasteIncineration*, part of F_9_0: accounts for the direct emissions of process 9, 9d, part of *SysExt_Impacts_WasteMgtRemelting_9di*.
- *Carbon_Fuelwood_bld*, part of F_7_0: part of *SysExt_Impacts_UsePhase_7d*.
- *Carbon_Fuelwood_el* (carbon in fuelwood for use in electricity generation), part of F_15_0: part of the indirect emissions ‘i’ of all other processes, and reported there according to the amount and type of energy carrier: 3i, 5i, 7i, 9i.

**Reporting in papers/reports**

- Reporting of biogenic C flows and stocks must fit to research question and system consistency principles.
- The mid-term goal is to couple RECC to CRAFT (SEC@BoKu) to have at least a default forest carbon sequestration scenario plus one or two alternative forest growth scenarios to check the robustness of the results.
- For the global building stock RECC standalone study, the following is proposed:
  - In the main results, report all flows as in Table S9, including biogenic carbon flows (esp. the large one in F_9_0)
  - Add F_0_1 (with the assumption of forestry with constant carbon pools) as a negative contribution (add to area plots!), and report Impacts_System_13579di as the new total (solid line for the total in area plots and final results).

This way, we have all emissions accounted for in the processes where they actually occur and have a reasonable assumption on the role of forest in biomass supply for the biomass levels that are being used in the different scenarios (except for HighWood SSP2, which is likely not feasible in terms of supply). Also, we can account for the impact of carbon storage in buildings and cascaded wood products on the climate.

**2.3. Sankey diagram export:**

**RECC v2.5 exports data that can be used to draw the now commonly used material flow accounting-based Sankey diagram first developed by Haas et al. (2015), Fig. S4:**


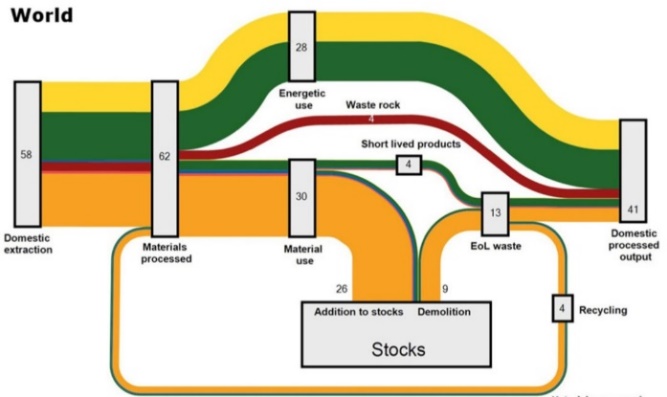

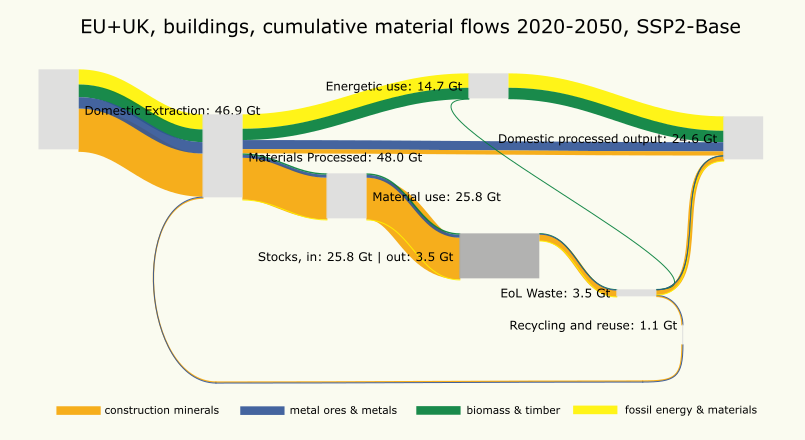


**Figure S4:** Haas et al. (2015) original (left) and suggested implementation for an auto-generated Sankey via a web application (right): <https://www.visualisation.industrialecology.uni-freiburg.de/>

The following list of indicators is used to build the Sankey. Each line from the visualisation generation code is matched with the corresponding indicator:

**Material extraction (left) and energy flows fossil and biomass on top:**

*Material footprint, biomass (dry weight), system-wide _3579di*

*Material footprint, fossil fuels, system-wide _3579di*

*Material footprint, metal ores, system-wide _3579di*

*Material footprint, non-metallic minerals, system-wide _3579di*

**Final consumption (left of use phase):**

*Final consumption of metals (aggregate materials group)*

*Final consumption of non-metallic minerals (aggregate materials group)*

*Final consumption of biomaterials/wood (aggregate materials group) + Wood for cascading (inflow)*

*Final consumption of plastics (aggregate materials group)*

**Outflow of material flows from use phase (right of use phase):**

*Use phase outflow of metals (aggregate materials group)*

*Use phase outflow of non-metallic minerals (aggregate materials group)*

*Use phase outflow of biomaterials/wood (aggregate materials group) + Outflow of cascading stock*

*Use phase outflow of plastics (aggregate materials group)*

**Recycling EoL material flows (bottom of graph):**

*Secondary production of metals (aggregate materials group)*

*Secondary production of non-metallic mineralic materials (aggregate materials group)*

*Secondary production of biomaterials/wood (aggregate materials group)*

*Secondary production of plastics (aggregate materials group)*

**to these values, add:**

*Reuse of metals (aggregate materials group)*

*Reuse of non-metallic minerals (aggregate materials group)*

*Reuse of biomaterials/wood (aggregate materials group)*

*Reuse of plastics (aggregate materials group)*

**to these values, add:**

*Wood cascading inflow from EoL construction wood.*

**Energy recovery from wood waste:**

*Outflow of cascading stock*

**All other flows:**

*Calculated endogenously from mass balance!*

**Note:** The Python RECC model code does not generate the Sankey diagram itself, only a text file with coded text that can be inserted into the Circular Sankey App available under the link below.

Further Sankey examples can be found as Fig. SP16 in the figure supplement to this work.

Plots for other regions can be generated by looking up the Sankey config files (text files) in the RECC v2.5 global buildings result dataset (see Zenodo link above) and by copy-pasting their content into Industrial Ecology Freiburg’s Circular Sankey App:

<https://www.visualisation.industrialecology.uni-freiburg.de/frmCircularSankey.aspx>

**References**

Bocken, Nancy M. P., Ingrid de Pauw, Conny Bakker, und Bram van der Grinten. 2016. „Product design and business model strategies for a circular economy“. *Journal of Industrial and Production Engineering* 33(5):308–20. doi: 10.1080/21681015.2016.1172124.

Fishman, Tomer, Niko Heeren, Stefan Pauliuk, Peter Berrill, Qingshi Tu, Paul Wolfram, und Edgar Hertwich. 2021. „Developing scenarios of resource efficiency and climate change: from conception to operation“. *Journal of Industrial Ecology* 25(2):305–20. doi: 10.1111/jiec.13122.

Grubler, Arnulf, Charlie Wilson, Nuno Bento, Benigna Boza-Kiss, Volker Krey, David L. McCollum, Narasimha D. Rao, Keywan Riahi, Joeri Rogelj, Simon De Stercke, Jonathan Cullen, Stefan Frank, Oliver Fricko, Fei Guo, Matt Gidden, Petr Havlík, Daniel Huppmann, Gregor Kiesewetter, Peter Rafaj, Wolfgang Schoepp, und Hugo Valin. 2018. „A Low Energy Demand Scenario for Meeting the 1.5 °C Target and Sustainable Development Goals without Negative Emission Technologies“. *Nature Energy* 3(6):515–27. doi: 10.1038/s41560-018-0172-6.

IEA. 2022. *World Energy Outlook 2022*. Paris, France.

Pauliuk, Stefan. 2023. *Documentation of the RECC model v2.5 - Open Dynamic Material Systems Model for the Resource Efficiency-Climate Change (RECC) Nexus*. 1(2023). Freiburg, DE.

Zhang, Luyang, Yankun Sun, Tianyuan Song, und Jiaqi Xu. 2019. „Harvested Wood Products as a Carbon Sink in China, 1900–2016“. *International Journal of Environmental Research and Public Health* 16(3):445. doi: 10.3390/ijerph16030445.
