# Supplementary material for: Scenario analysis of supply‐ and demand‐side solutions for circular economy and climate change mitigation in the global building sector
Source: J Ind Ecol. 2024 Oct 8;28(6):1699–715. doi: 10.1111/jiec.13557 (PMC11667659; doi:10.1111/jiec.13557)
Supplement: Supplementary file 3 — • Supplementary material 3: Figure supplement: Region‐specific plots and supplementary figures. • The RECC v2.5 model documentation: https://doi.org/10.6094/UNIFR/242061 • The RECC v2.5 model repository: https://github.com/IndEcol/RECC‐ODYM • The RECC v2.5 global buildings input and results dataset: https://zenodo.org/records/12752350 • The BuildME modeling framework for building archetypes: https://github.com/nheeren/BuildME (Krych et al., 2024) [file JIEC-28-1699-s002.pdf]

universität freiburg

Collection of additional result plots

Scenario analysis of supply- and demand-side solutions for circular economy and climate change mitigation in the global building sector

Supplementary material 3 - Figure supplement: Region-specific plots and supplementary figures

Stefan Pauliuk(*), Fabio Carrer, Niko Heeren, and Edgar Hertwich

*) stefan.pauliuk@indecol.uni-freiburg.de

Industrial Ecology Freiburg
Research group at the Faculty of Environment and Natural Resources

Overview

Links to publication and data

Dematerializing construction: The emissions and resource savings potential for circular economy, lightweight design, and material substitution in the global building sector, by Pauliuk et al., submitted for peer review to the Journal of Industrial Ecology.

This document contains the visualization of the results of a larger open science effort to depict the future of the energy and material service cascade in different sectors and regions. The focus of this work is the global building stock (residential and non-residential buildings).

Next to the journal publication, this work comes with a number of supporting documents, datasets, and models:

- Supplementary material 1: Scenario details and traceability of results
- Supplementary material 2: Core numerical results as reported in paper
- Supplementary material 3: Region-specific plots
- The RECC v2.5 model documentation: https://doi.org/10.6094/UNIFR/242061
- The RECC v2.5 model repository: https://github.com/IndEcol/RECC-ODYM
- The RECC v2.5 global buildings input dataset via Zenodo: https://zenodo.org/records/12752350
- The RECC v2.5 global buildings result dataset via Zenodo: https://zenodo.org/records/12752350

→ **The result plots shown here are available via the RECC v2.5 global buildings result dataset on Zenodo.**

Figure SP1: System definition RECC v2.5 global buildings

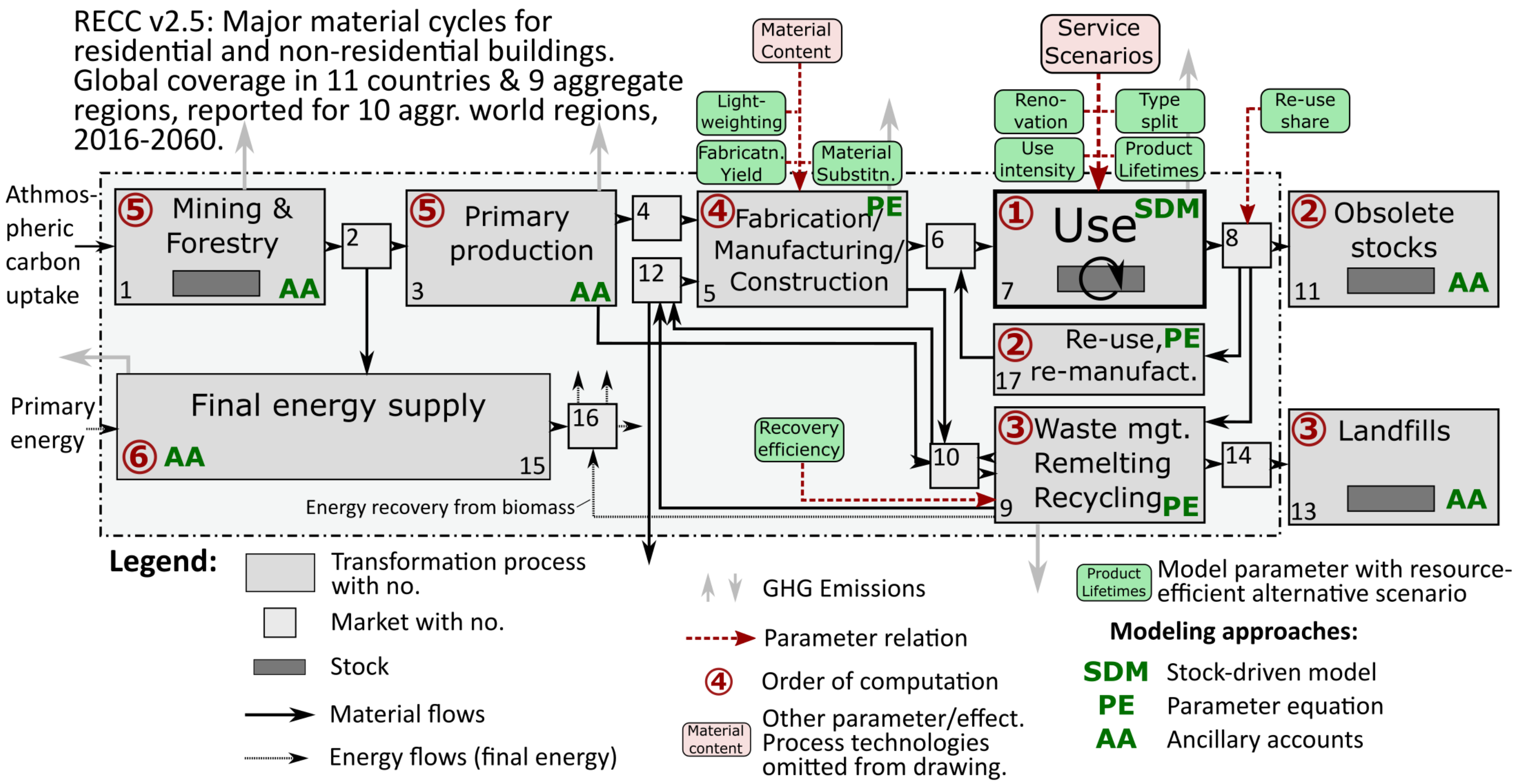

Figure SP1: System definition of the RECC model v2.5. The model covers the use phase of residential and non-residential buildings (scope 1); their energy supply for the three services heating, cooling, and hot water (scope 2); and the material production, recycling, and manufacturing activities associated with buildings (scope 3).

Source: This paper (own illustration)

Table SP1a: Stock of residential buildings (reb), m²/cap

m²/capita	2015	2050, LED	2050, SSP1	2050, SSP2	
**USA, Can**	60-70	37	65	80	very reb-intensive: 60-80 m²/cap
**China, Japan, France, Germany, Italy**	35-45	30	40	48	reb-intensive: 60-80 m²/cap
**UK, Latin America, MENA, Poland**	25-35	30	40	45	reb-intensive: 15-25 m²/cap
**India, Sub-Saharan Africa, South Asia**	12-18	20-30	30-35	40	reb-lean: about 15 m²/cap

Socio-economic scenarios:
Low Energy Demand (LED, Grübler et al. 2018)
and shared socio-economic pathways SSP1 (easy mitigation and adaptation) and SSP2 (moderate adaptation and mitigation)
Energy supply scenarios:
Baseline with no further climate policy and a RCP2.6-compatible energy supply system

Table SP1b: Stock of non-residential bldgs. (nrb), m²/cap

m²/capita	2015	2050, LED	2050, SSP1	2050, SSP2	
**USA, Can, Ger**	21-24	18	26	30	nrb-intensive: 15-25 m²/cap
**Japan, China, France, Poland, UK, Other EU**	10-16	13	16	20	nrb-intensive: 15-25 m²/cap
**Italy, Spain, Mexico, Turkey, Russia**	5-9	9	12	15	nrb-intensive: 15-25 m²/cap
**India, Sub-Saharan Africa, South-East Asia**	1-3	7	10	12	nrb-lean: about 10 m²/cap

Socio-economic scenarios:
Low Energy Demand (LED, Grübler et al. 2018)
and shared socio-economic pathways SSP1 (easy mitigation and adaptation) and SSP2 (moderate adaptation and mitigation)
Energy supply scenarios:
Baseline with no further climate policy and a RCP2.6-compatible energy supply system

UNI FREIBURG

Figure SP2: Material efficiency strategies for buildings

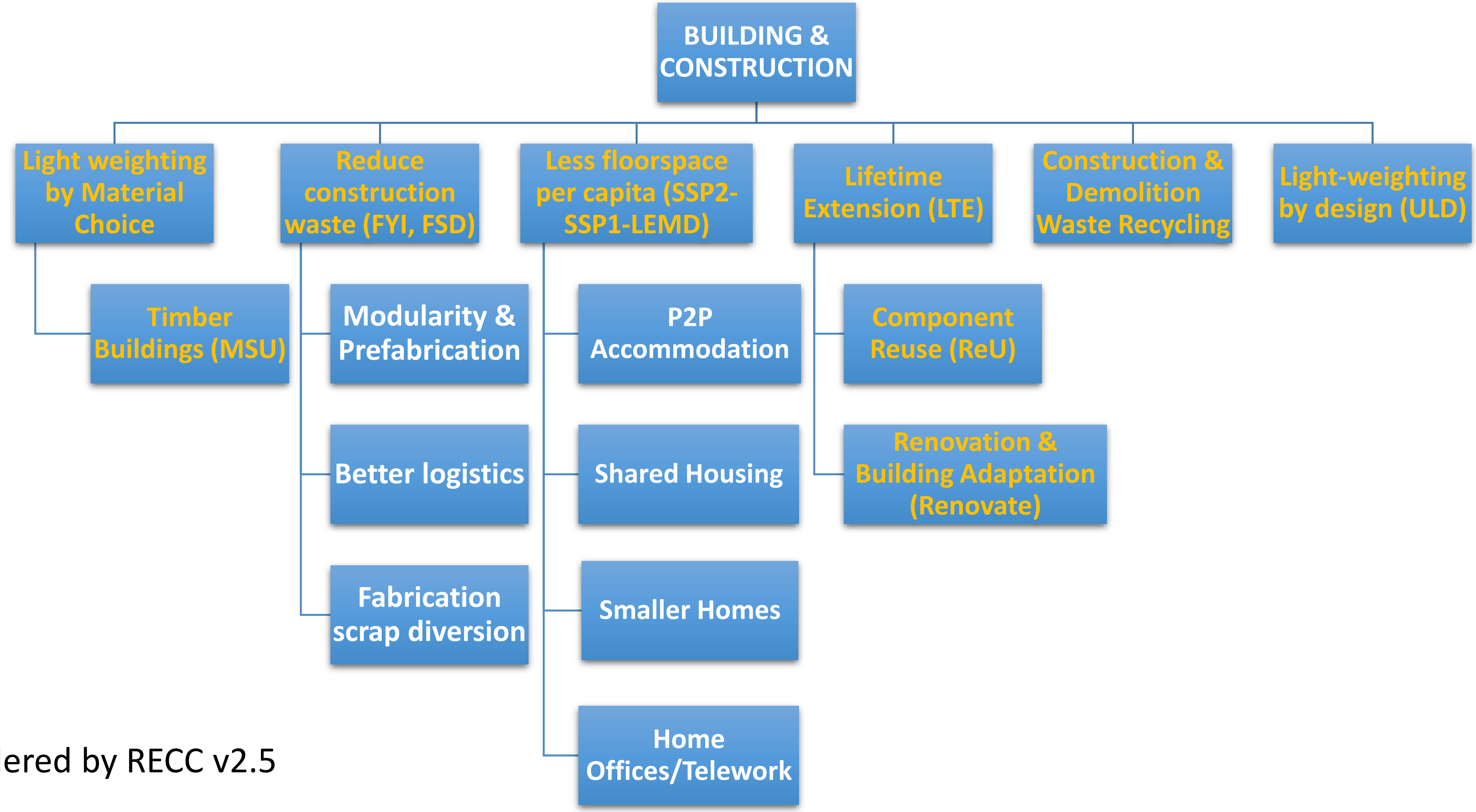

Yellow: Considered by RECC v2.5

The RECC team, in particular: Peter Berrill, Reid Lifset, and Niko Heeren

Source: IRP (2020). Resource Efficiency and Climate Change: Material Efficiency Strategies for a Low-Carbon Future. Summary for Policy makers. DOI: 10.5281/zenodo.3542680

Table SP2: Scenario definition

Main scenarios	SSP	RCP	Description	Figures (paper)
LEMD_Base	LED	RCP2.6	Low material and energy demand (LEMD), efficiency and ren. energy, no CE, low wood	2a+b, 4b+d
SSP1_Base	SSP1	RCP2.6	SSP1, efficiency and ren. energy, no CE, low wood	2a+b, 4c+d
SSP2_Base	SSP2	RCP2.6	SSP2 socio-economics, efficiency and low carbon renewable energy, no CE, low wood	2a+b, 3a+b, 4a+d+e, 5, 6b
SSP2_FullCE	SSP2	RCP2.6	SSP2, eff. & ren. en., full CE: long life + reuse + recycling + light weighting, low wood	3b, 4d+e, 5, 6a+b
SSP1_FullCE	SSP1	RCP2.6	SSP1, efficiency and ren. energy, full CE, low wood	3b, 4c+d+e, 5
LEMD_FullCE	LED	RCP2.6	Low material and energy demand (LEMD), efficiency and ren. energy, Full CE, low wood	3b, 4d+e, 5, 6a
**Sensitivity scenarios**	**SSP**	**RCP**	**Description**	**Figures**
SSP2_Light	SSP2	RCP2.6	SSP2 socioeconomics, efficiency and ren. energy, light-weighting, low wood	3a
SSP2_Slow	SSP2	RCP2.6	SSP2 socioeconomics, efficiency and ren. energy, long lifetime, low wood	3a, 4e
SSP2_Slow_Close	SSP2	RCP2.6	SSP2, efficiency and ren. energy, light-weighting + reuse + recycling, low wood	3a, 4e
SSP2_Wood	SSP2	RCP2.6	SSP2 socioeconomics, efficiency and ren. energy, no CE, high wood	3a, 6b
SSP2_FullCE_Wood	SSP2	RCP2.6	SSP2, efficiency and ren. energy, Full CE, ‘Wood’: high wood intensity in new buildings	6a+b
LEMD_Light	LED	RCP2.6	LEMD socioeconomics, efficiency and ren. energy, light-weighting, low wood	3a
LEMD_FullCE_Wood	LED	RCP2.6	LEMD, efficiency and ren. energy, Full CE, ‘Wood’: high wood intensity in new buildings	3a+b, 6a
SSP2_Fossil	SSP2	NoClimPol	SSP2 socioeconomics, fossil-intensive reference, no CE, low wood	4a+d, 5, 6a+b
SSP2_Fossil_FullCE	SSP2	NoClimPol	SSP2 socioeconomics, fossil-intensive reference, full CE, low wood	4d, 6b
SSP2_Fossil_Wood	SSP2	NoClimPol	SSP2 socioeconomics, fossil-intensive reference, no CE, wood-intensive buildings	6a+b
SSP2_Fossil_FullCE_Wood	SSP2	NoClimPol	SSP2, fossil-intensive reference, Full CE, ‘Wood’: high wood intensity in new buildings	6b
LEMD_Fossil	LED	NoClimPol	LEMD socioeconomics, fossil-intensive reference, no CE, low wood	4b+d, 6a
LEMD_Fossil_FullCE	LED	NoClimPol	LEMD socioeconomics, fossil-intensive reference, full CE, low wood	4d
LEMD_Fossil_Wood	LED	NoClimPol	LEMD socioeconomics, fossil-intensive reference, no CE, wood-intensive buildings	6a
SSP1_Fossil	SSP1	NoClimPol	SSP1 socioeconomics, fossil-intensive reference, no CE, low wood	4d
SSP1_Fossil_FullCE	SSP1	NoClimPol	SSP1 socioeconomics, fossil-intensive reference, full CE, low wood	4d

Fig. SP3: Overall decoupling of GHG in the energy service cascade

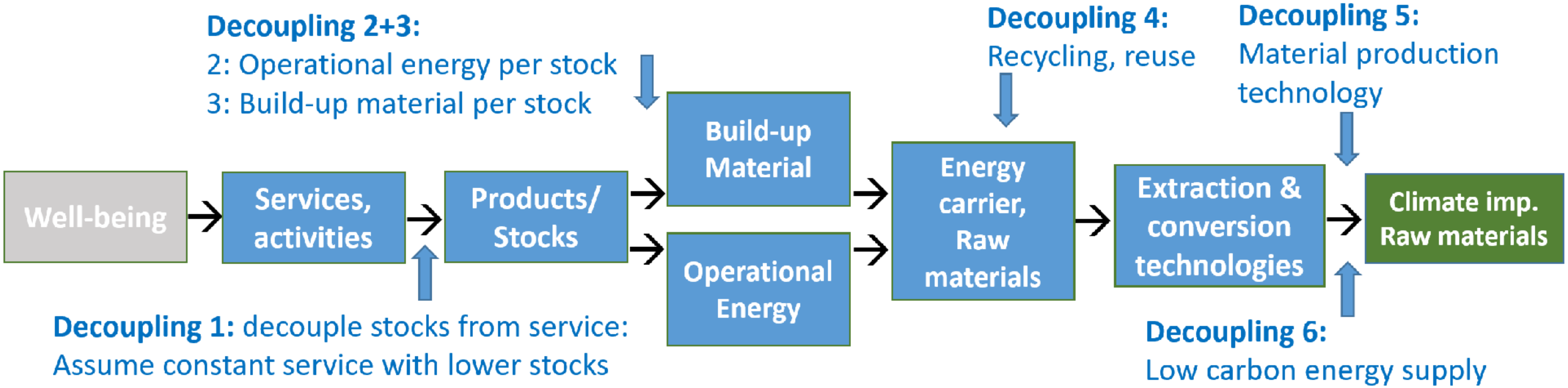

Source: This paper (own illustration)

Fig. SP4: Overall decoupling of GHG in the energy service cascade

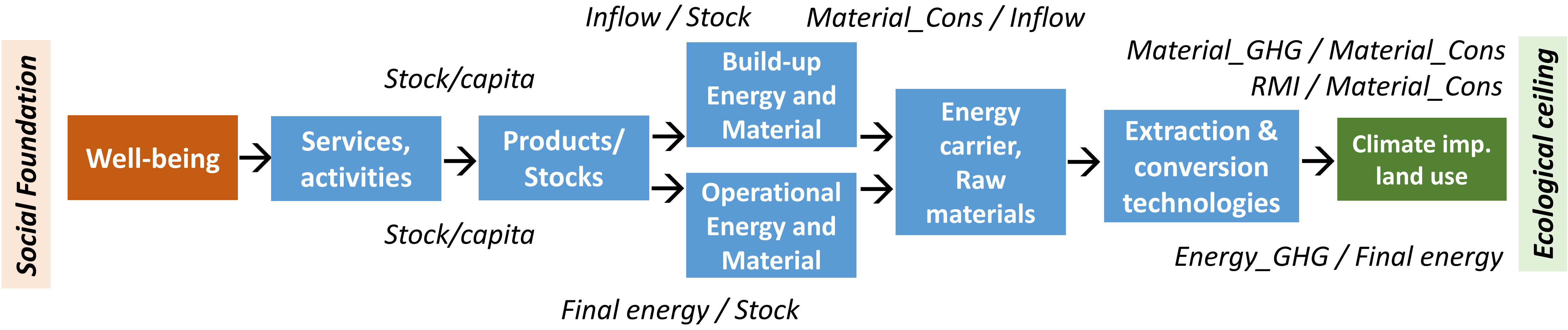

$$\frac{Energy\_GHG}{capita} = \frac{Energy\_GHG}{Final\_Energy} \cdot \frac{Final\_Energy}{Stock} \cdot \frac{Stock}{capita}$$

$$\frac{Material\_GHG}{capita} = \frac{Material\_GHG}{Material\_Cons} \cdot \frac{Material\_Cons}{Inflow} \cdot \frac{Inflow}{Stock} \cdot \frac{Stock}{capita}$$

$$\frac{RMI}{capita} = \frac{RMI}{Material\_Cons} \cdot \frac{Material\_Cons}{Inflow} \cdot \frac{Inflow}{Stock} \cdot \frac{Stock}{capita}$$

Supplementary Results

Fig. SP5: m² stocks and flows for all regions

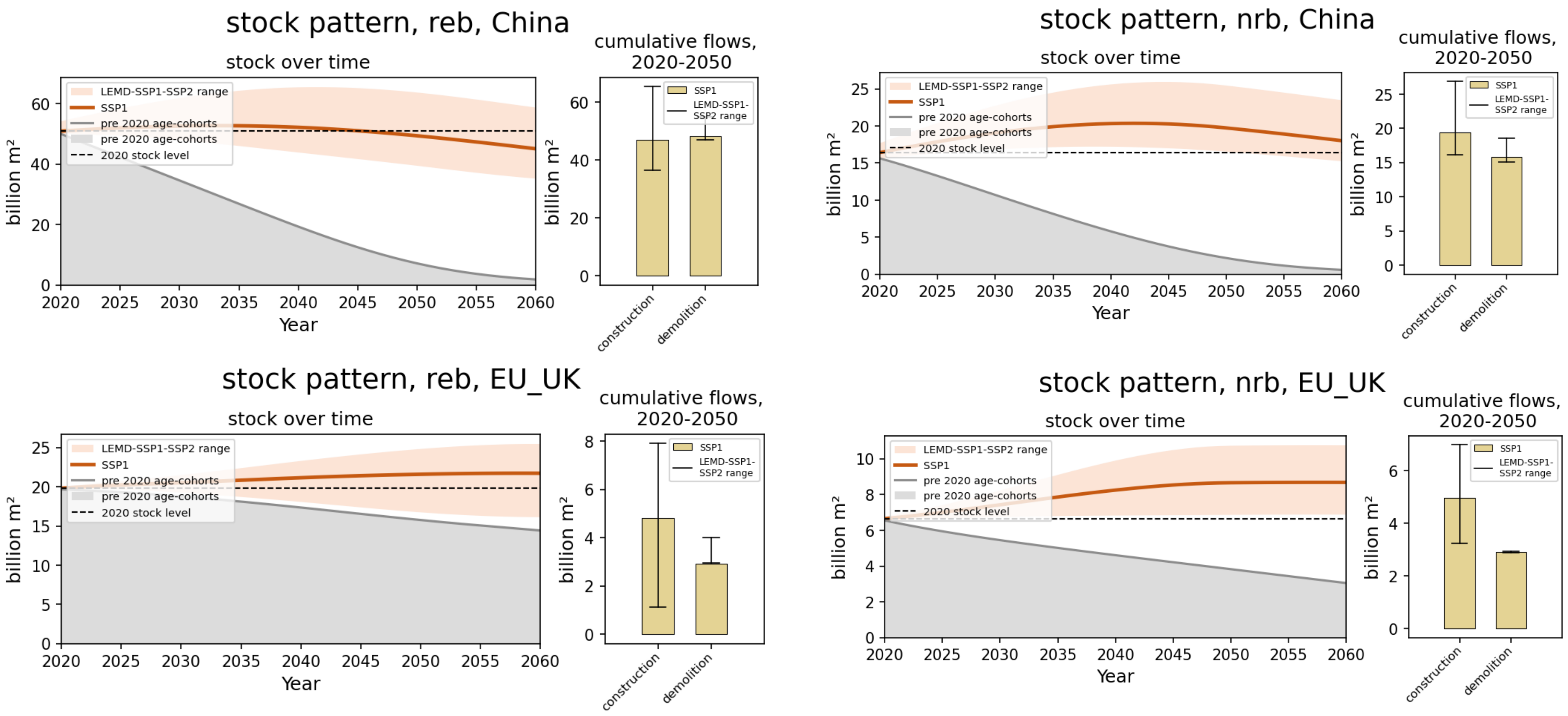

Figure SP5: Global total building stock (S_7) by scenario, year, and region (left); time series of the in-use stock (S_7) with lock-in from pre-2020 age-cohorts and range of future stock levels across scenarios (middle), and the range of the 2020-2050 cumulative new construction (F_6_7) and demolition (F_7_8) across scenarios (right), for residential buildings (a) and non-residential buildings (b).

Source: RECCv2.5 model calculation

Fig. SP5: m² stocks and flows for all regions

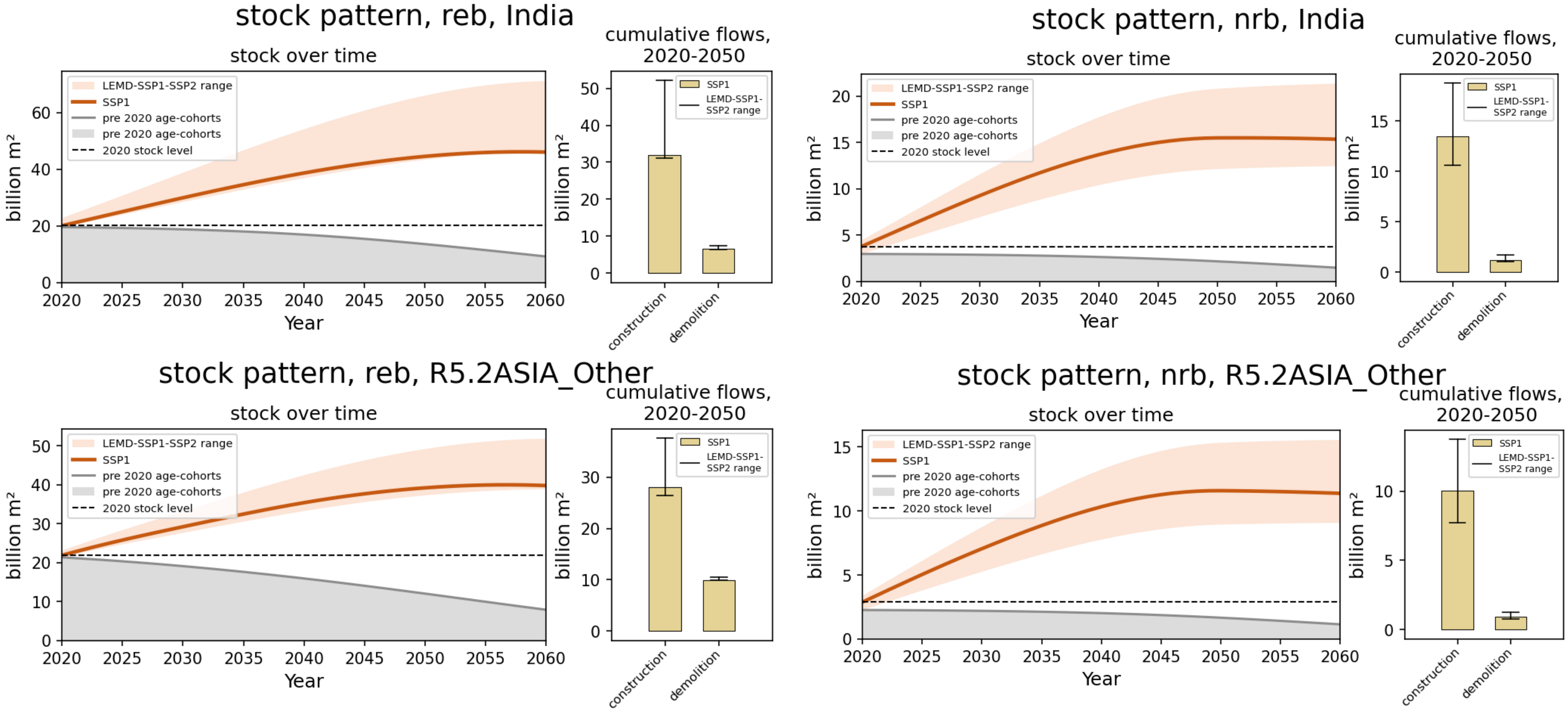

Figure SP5: Global total building stock (S_7) by scenario, year, and region (left); time series of the in-use stock (S_7) with lock-in from pre-2020 age-cohorts and range of future stock levels across scenarios (middle), and the range of the 2020-2050 cumulative new construction (F_6_7) and demolition (F_7_8) across scenarios (right), for residential buildings (a) and non-residential buildings (b).

Source: RECCv2.5 model calculation

Fig. SP5: m² stocks and flows for all regions

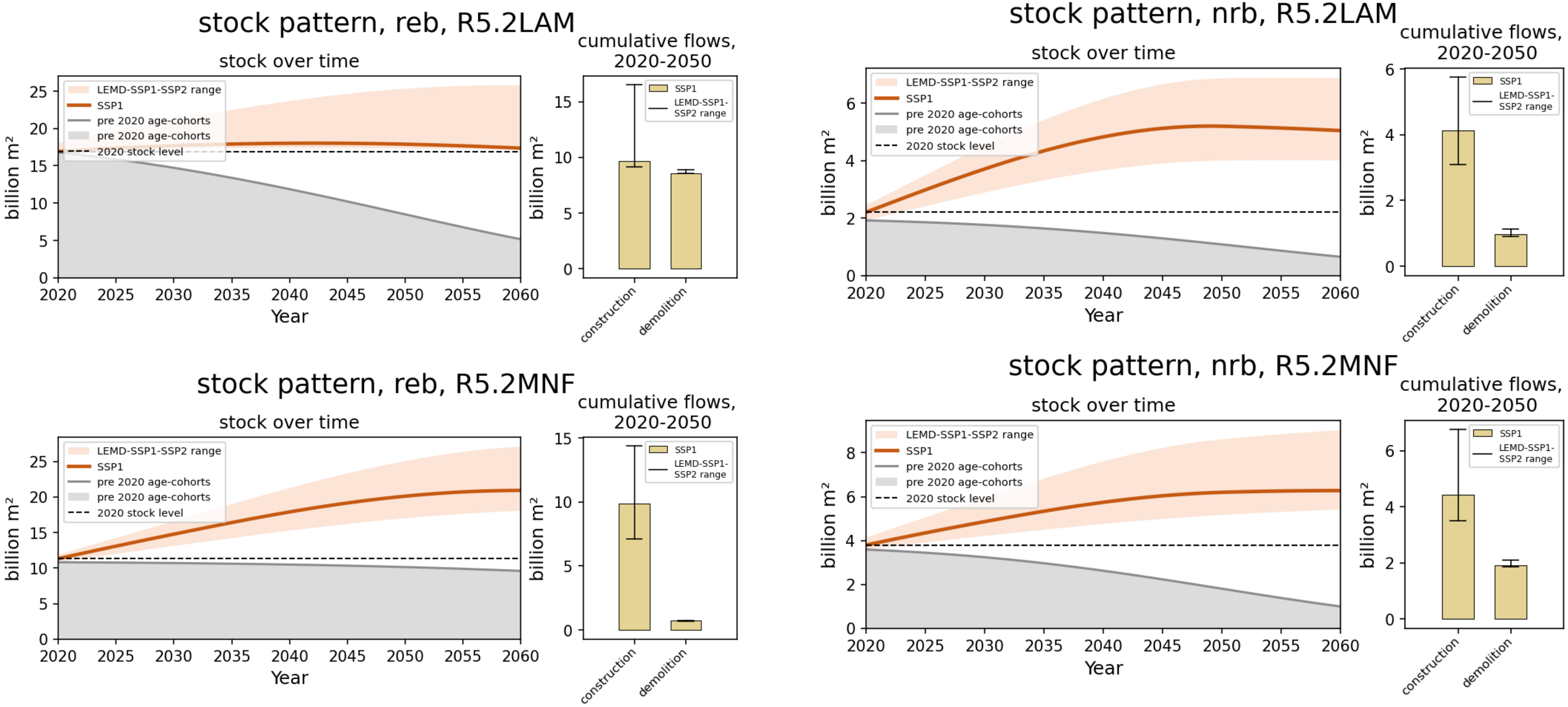

Figure SP5: Global total building stock (S_7) by scenario, year, and region (left); time series of the in-use stock (S_7) with lock-in from pre-2020 age-cohorts and range of future stock levels across scenarios (middle), and the range of the 2020-2050 cumulative new construction (F_6_7) and demolition (F_7_8) across scenarios (right), for residential buildings (a) and non-residential buildings (b).

Source: RECCv2.5 model calculation

Fig. SP5: m² stocks and flows for all regions

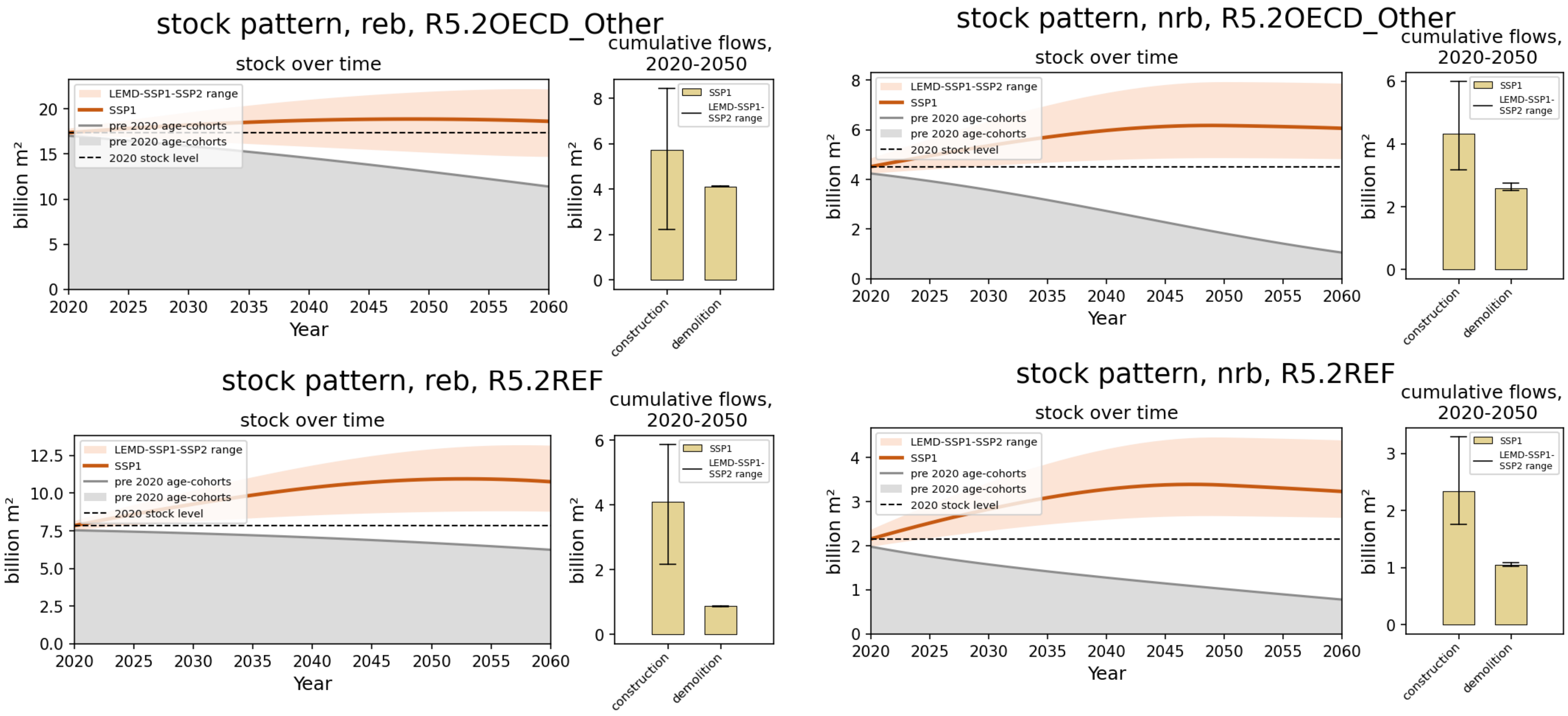

Figure SP5: Global total building stock (S_7) by scenario, year, and region (left); time series of the in-use stock (S_7) with lock-in from pre-2020 age-cohorts and range of future stock levels across scenarios (middle), and the range of the 2020-2050 cumulative new construction (F_6_7) and demolition (F_7_8) across scenarios (right), for residential buildings (a) and non-residential buildings (b).

Source: RECCv2.5 model calculation

Fig. SP5: m² stocks and flows for all regions

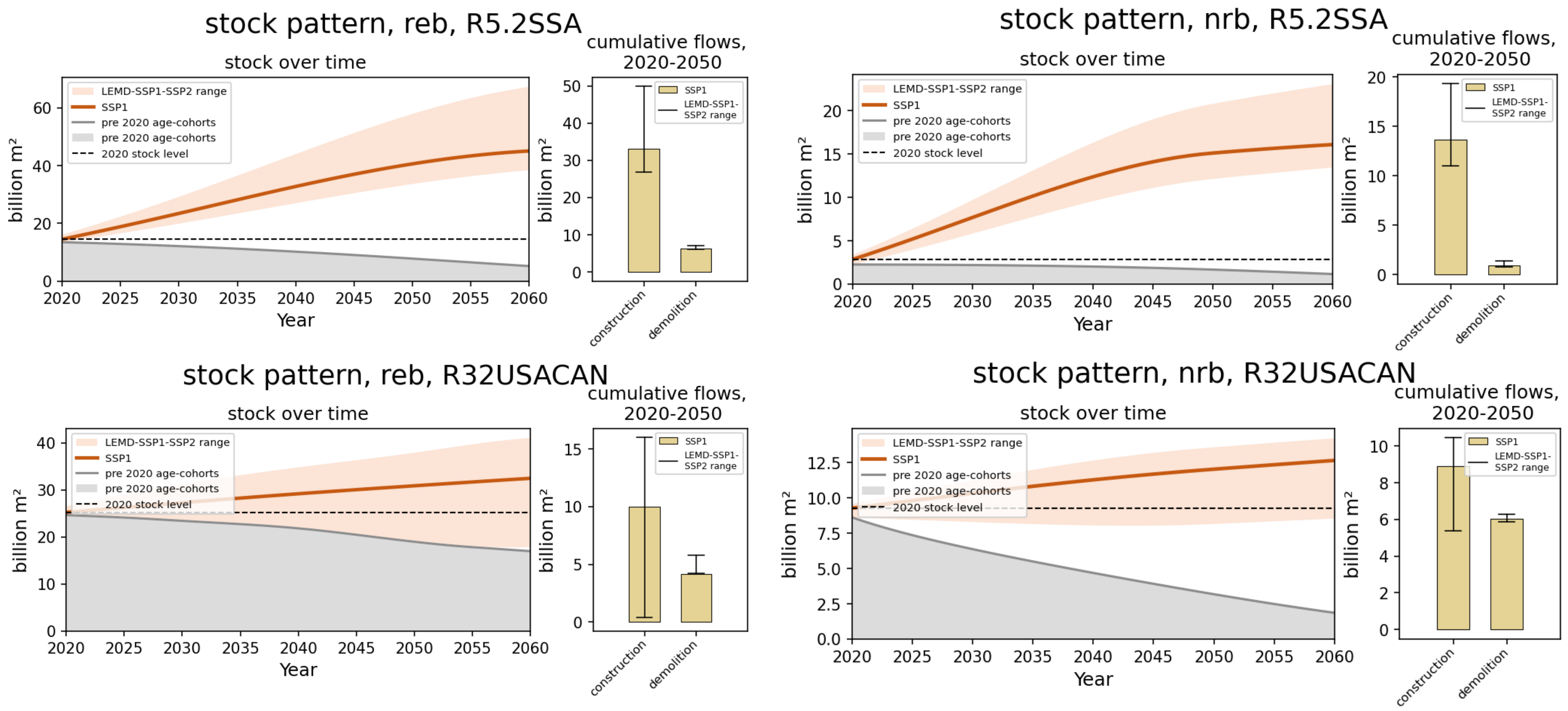

Figure SP5: Global total building stock (S_7) by scenario, year, and region (left); time series of the in-use stock (S_7) with lock-in from pre-2020 age-cohorts and range of future stock levels across scenarios (middle), and the range of the 2020-2050 cumulative new construction (F_6_7) and demolition (F_7_8) across scenarios (right), for residential buildings (a) and non-residential buildings (b).

Source: RECCv2.5 model calculation

Overview time series of material inflow by region, material, sector, and scenario (Fig. SP6)

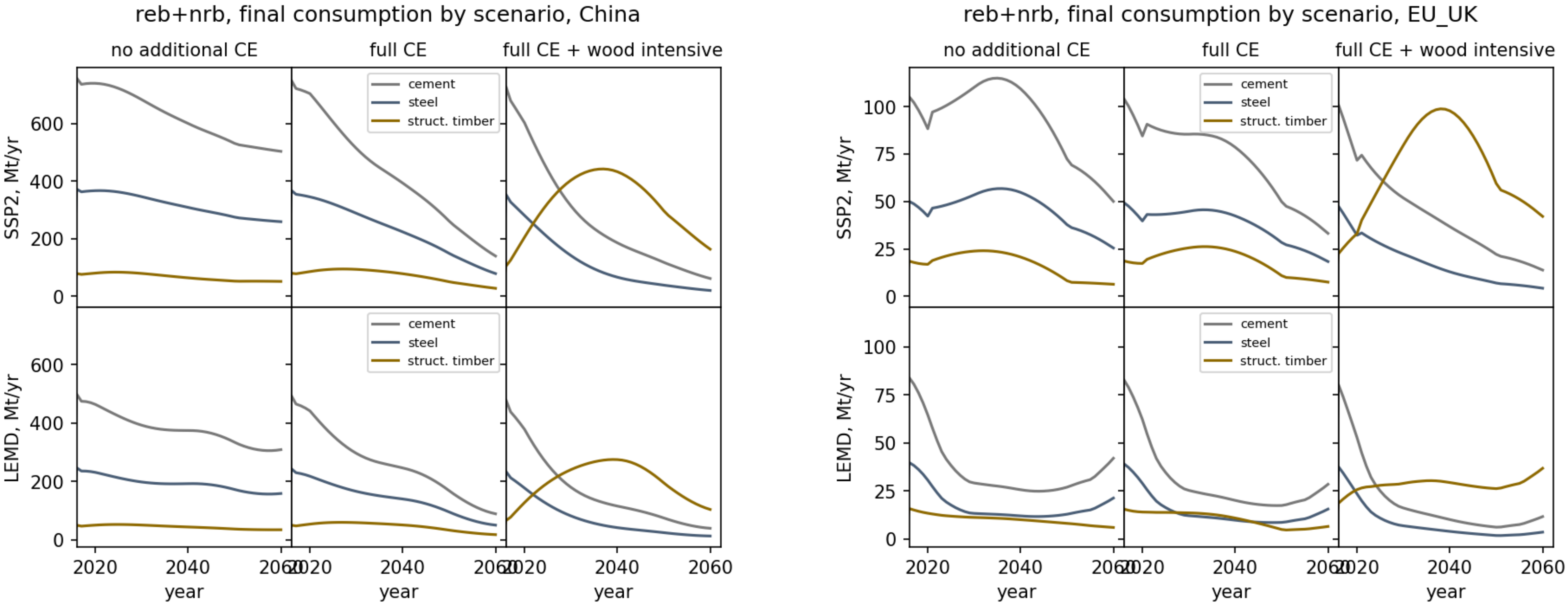

Overview time series of material inflow by region, material, sector, and scenario (Fig. SP6)

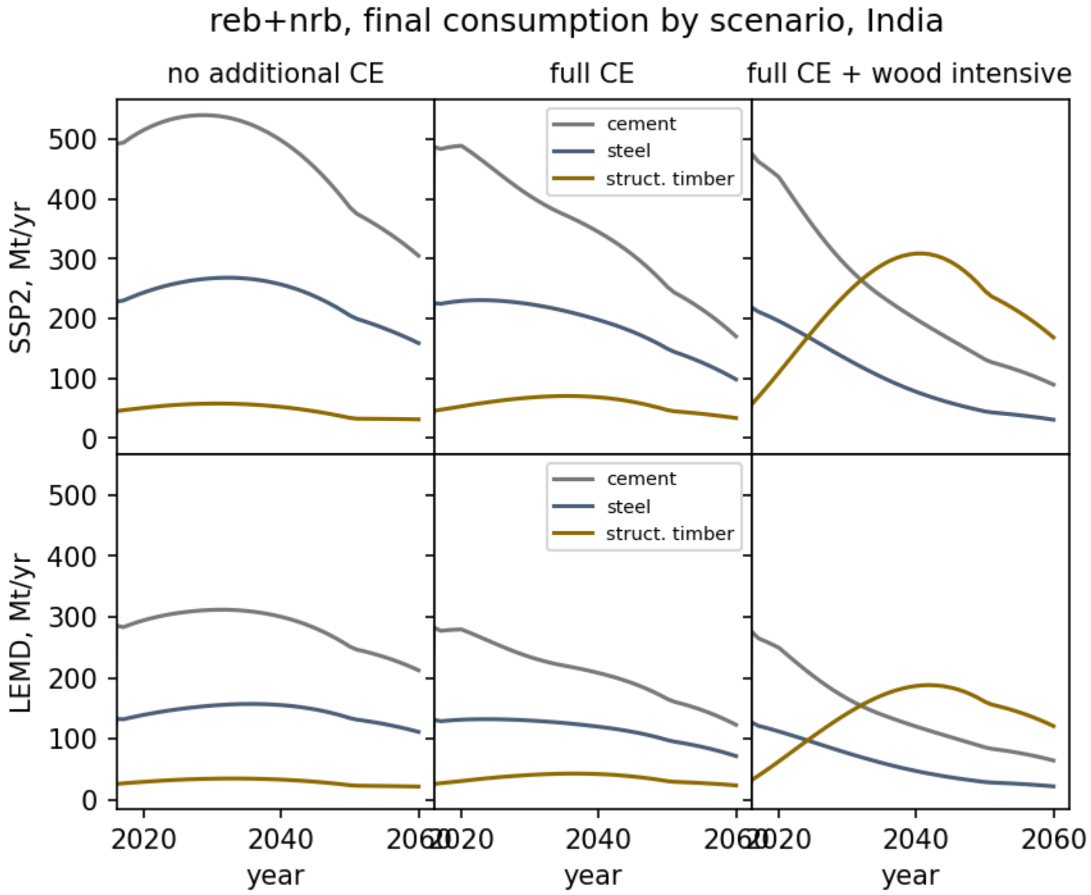

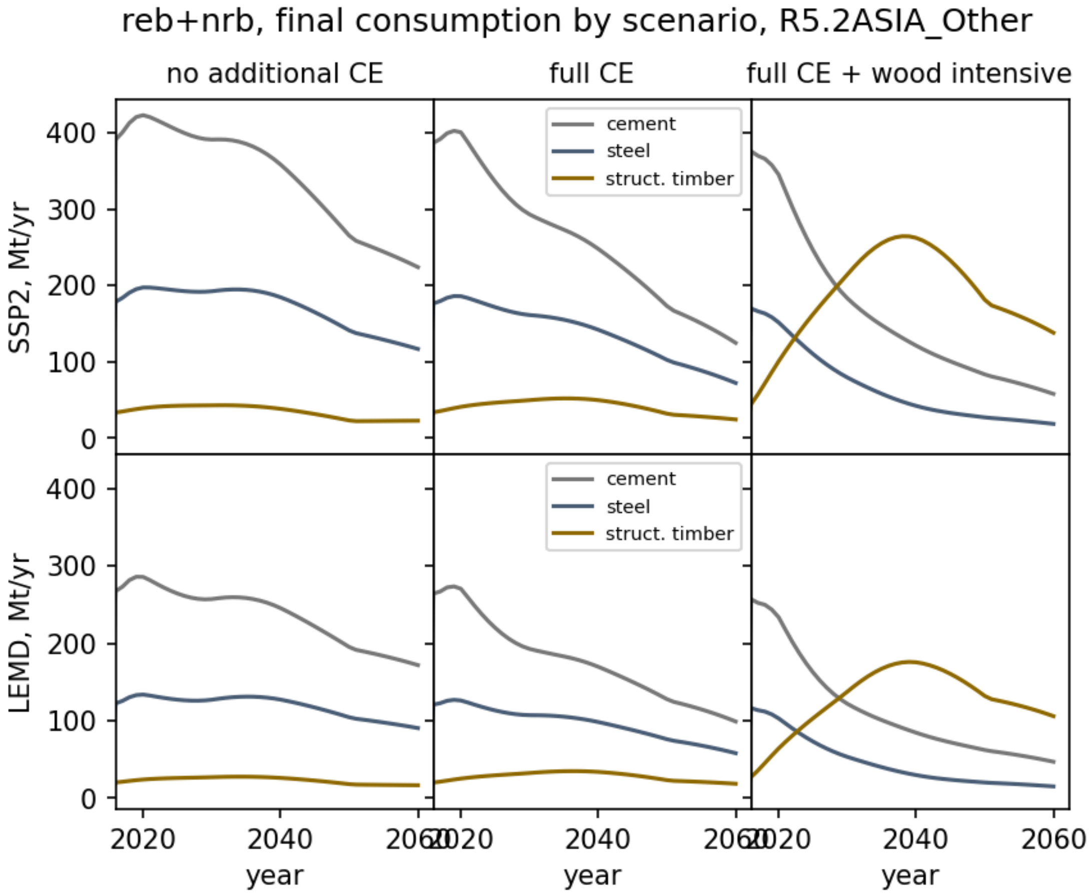

Overview time series of material inflow by region, material, sector, and scenario (Fig. SP6)

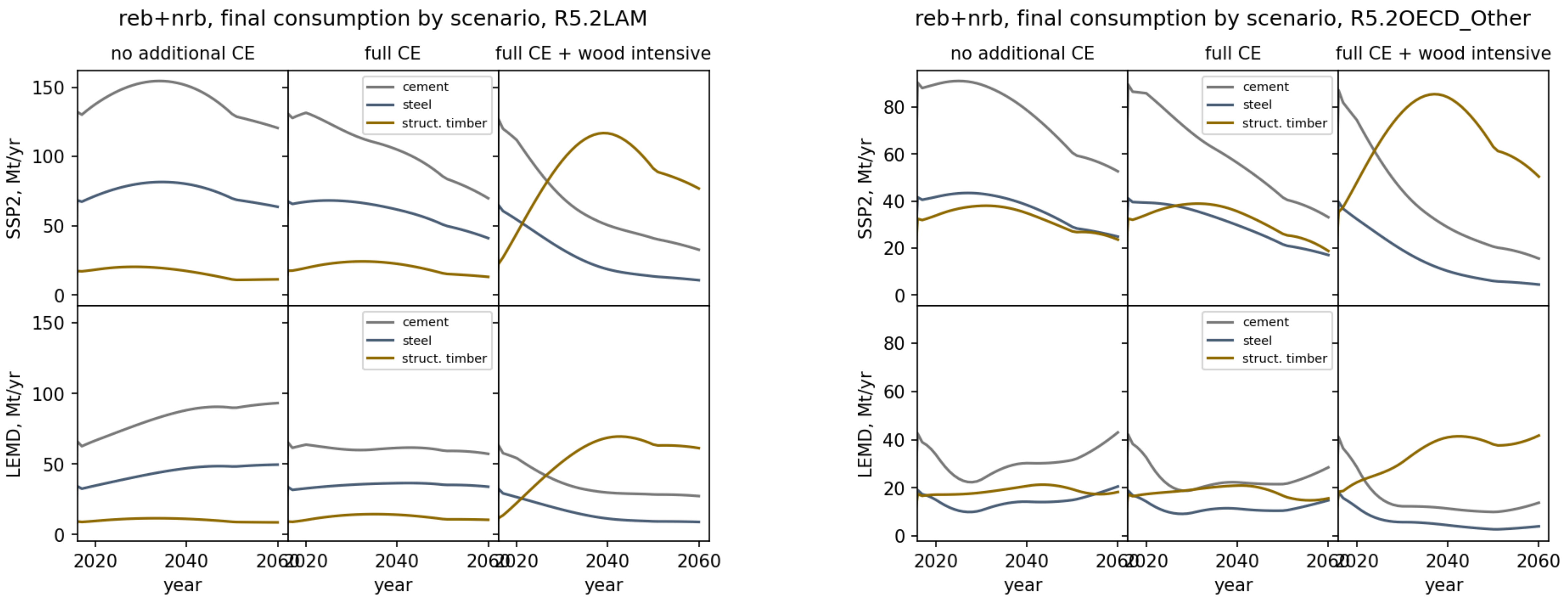

Overview time series of material inflow by region, material, sector, and scenario (Fig. SP6)

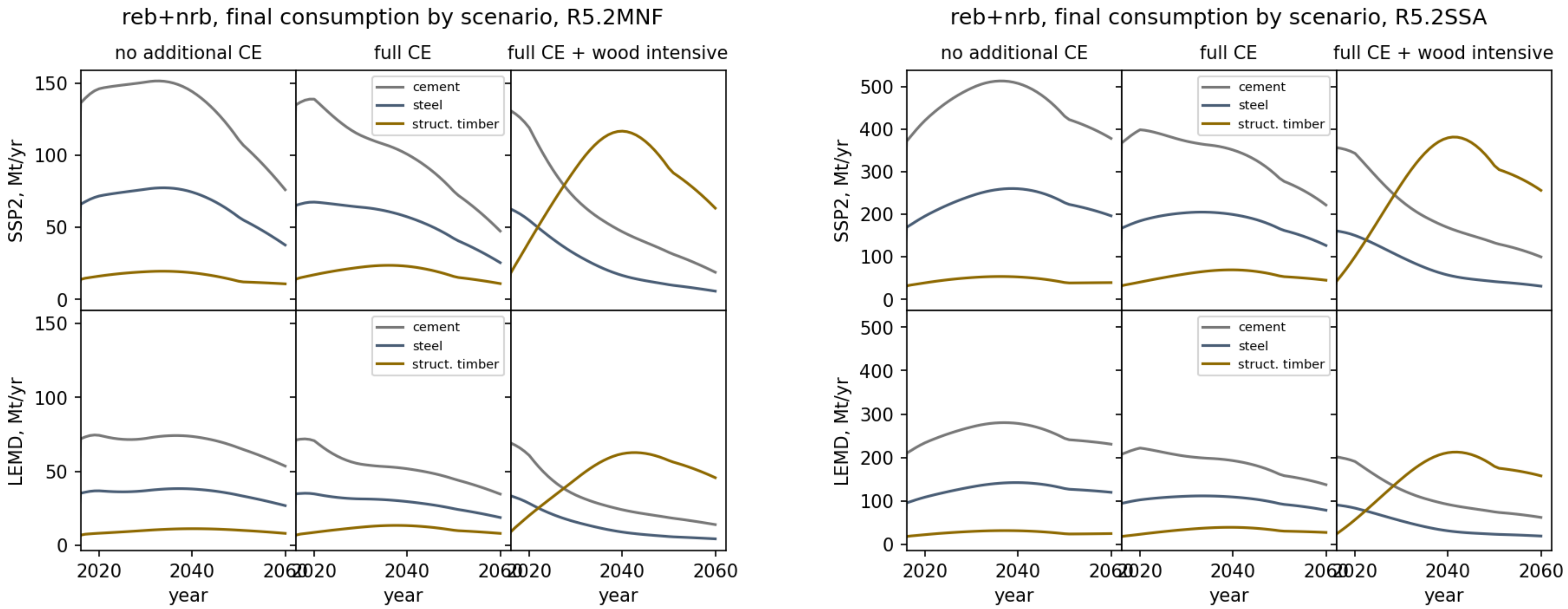

Overview time series of material inflow by region, material, sector, and scenario (Fig. SP6)

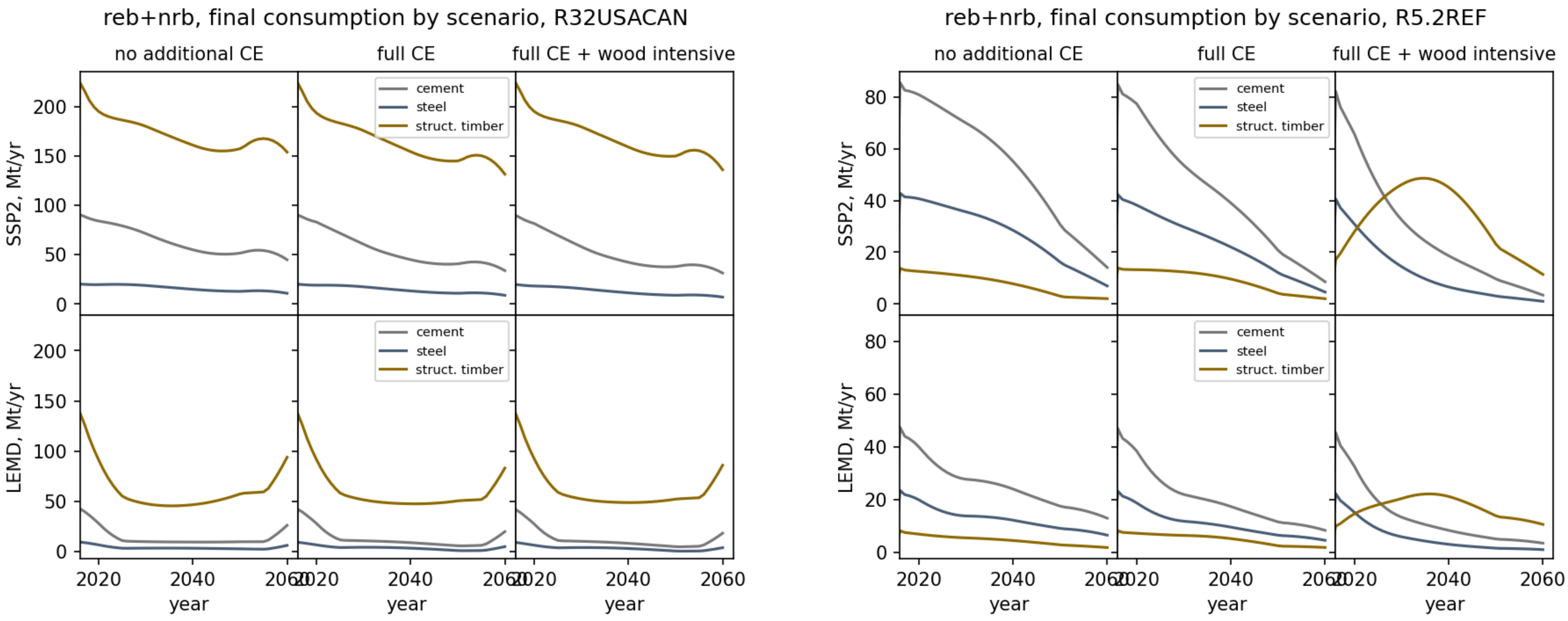

Overview time series of material inflow by region, material, sector, and scenario (Fig. SP6)

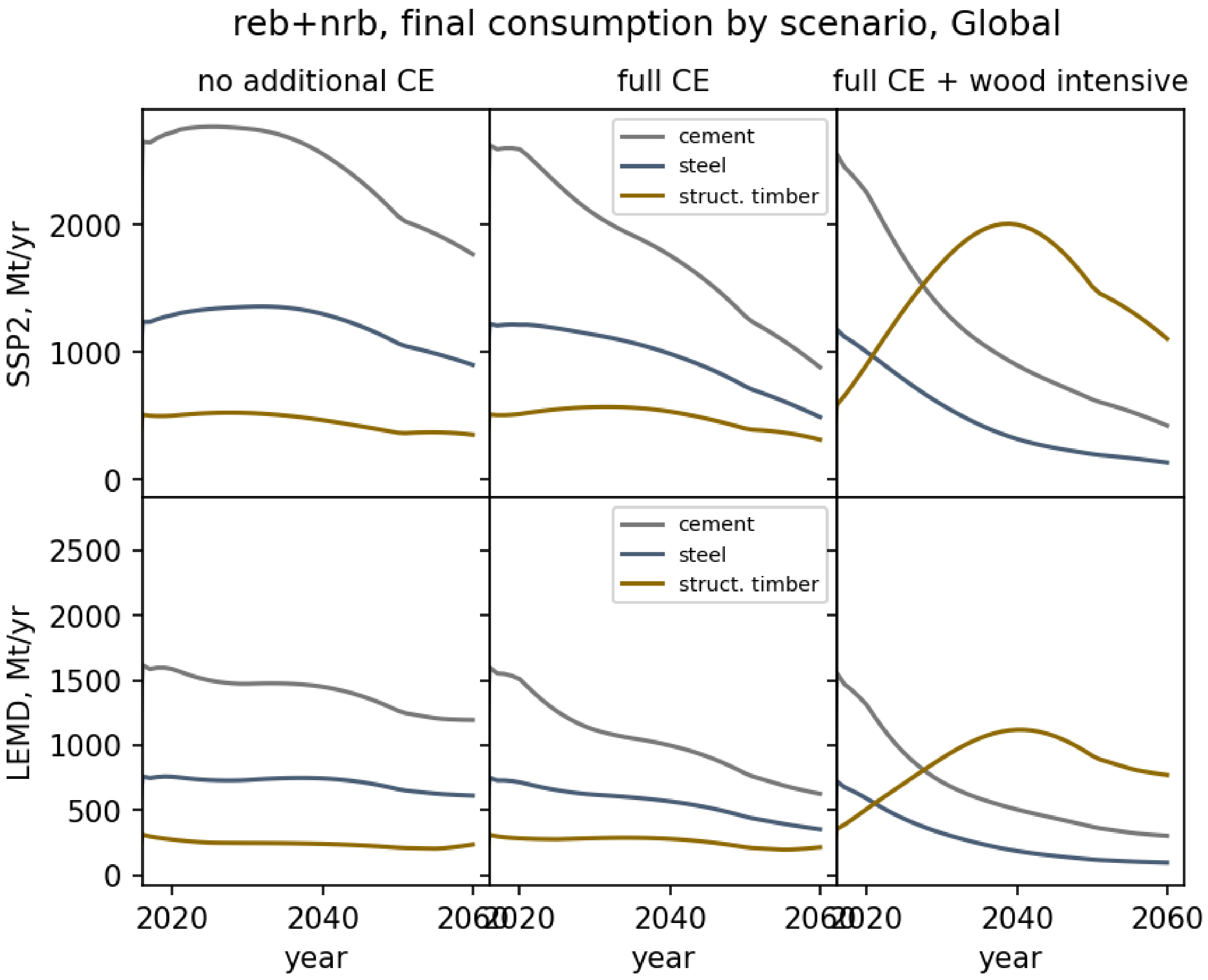

Overview time series of material stock by region, material, sector, and scenario (Fig. SP7)

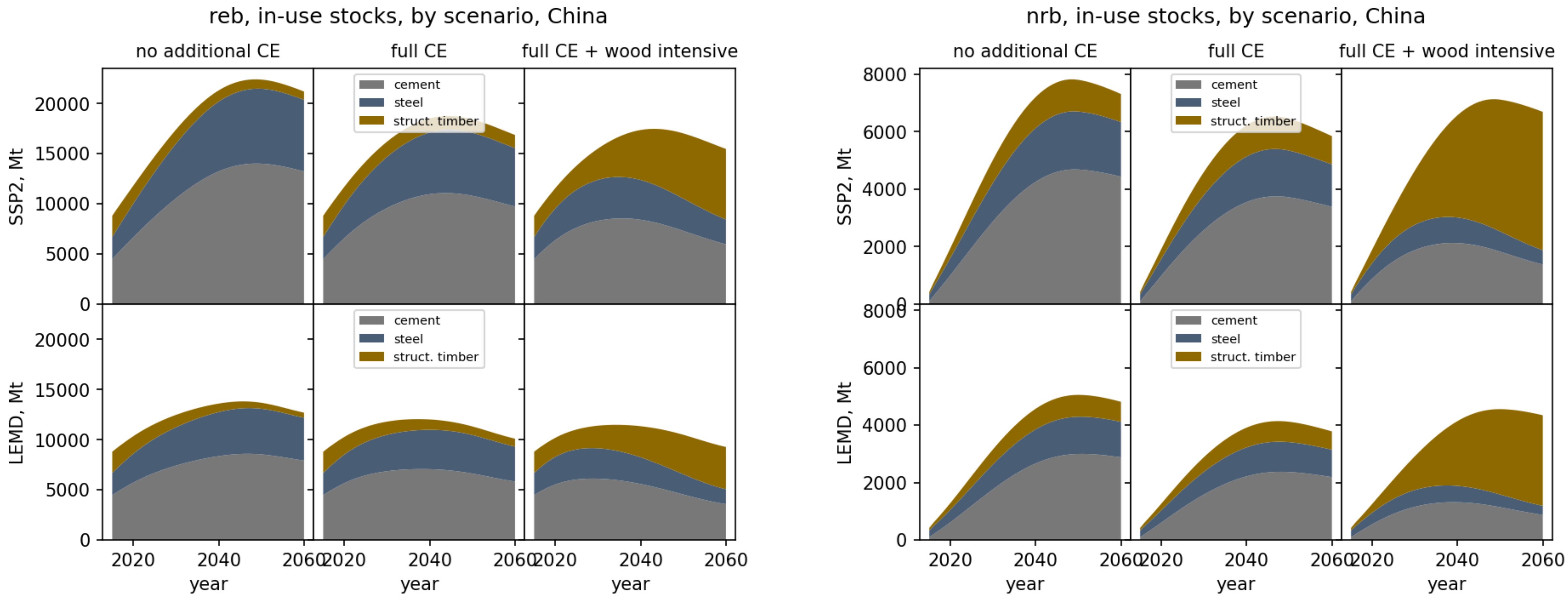

Overview time series of material stock by region, material, sector, and scenario (Fig. SP7)

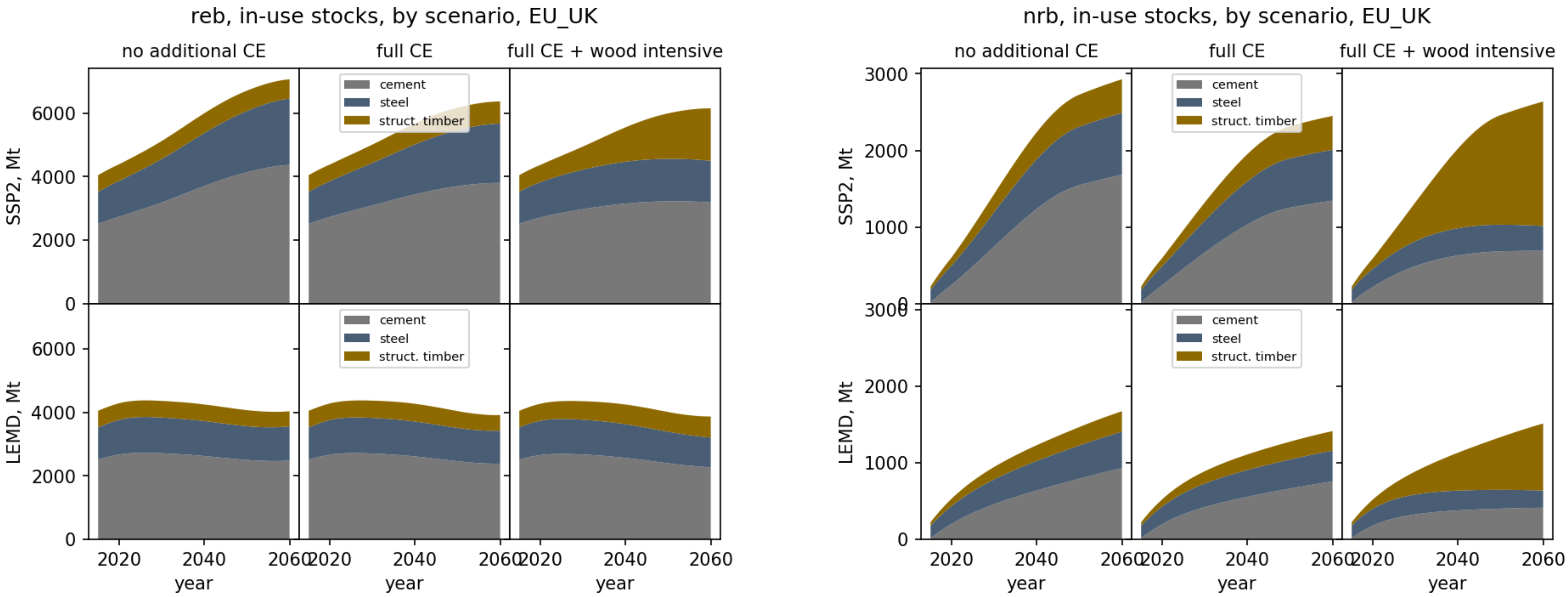

Overview time series of material stock by region, material, sector, and scenario (Fig. SP7)

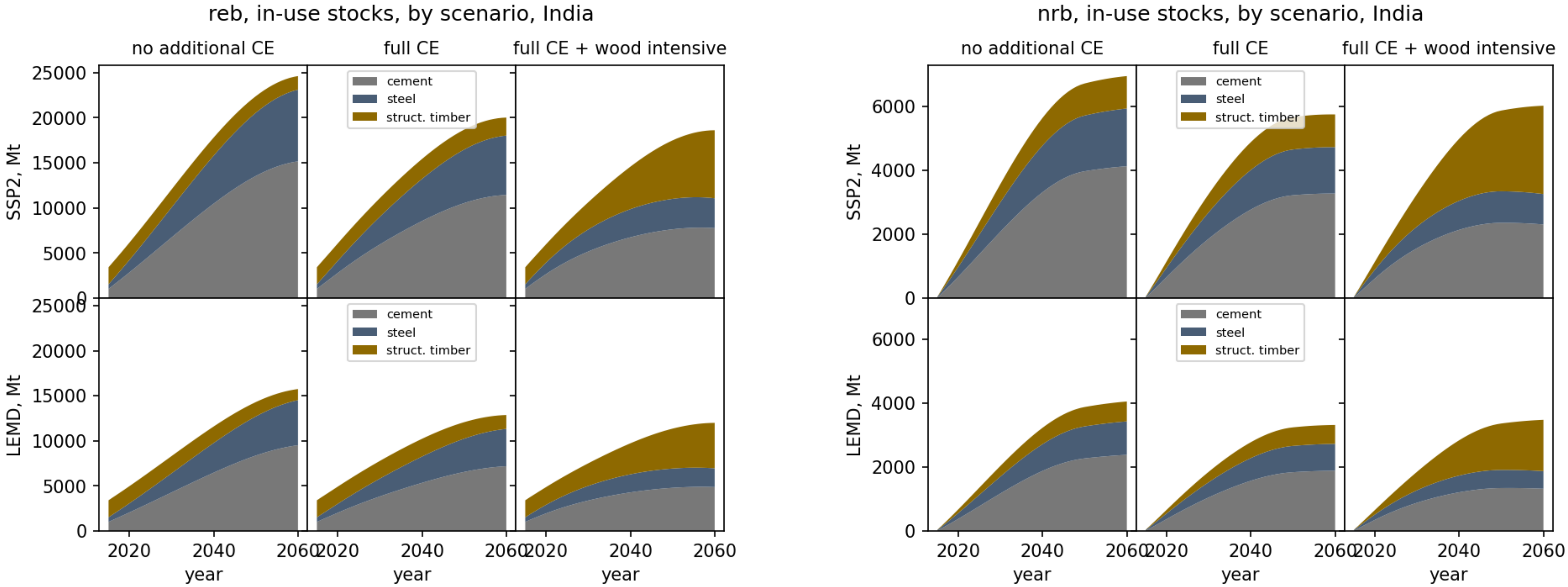

Overview time series of material stock by region, material, sector, and scenario (Fig. SP7)

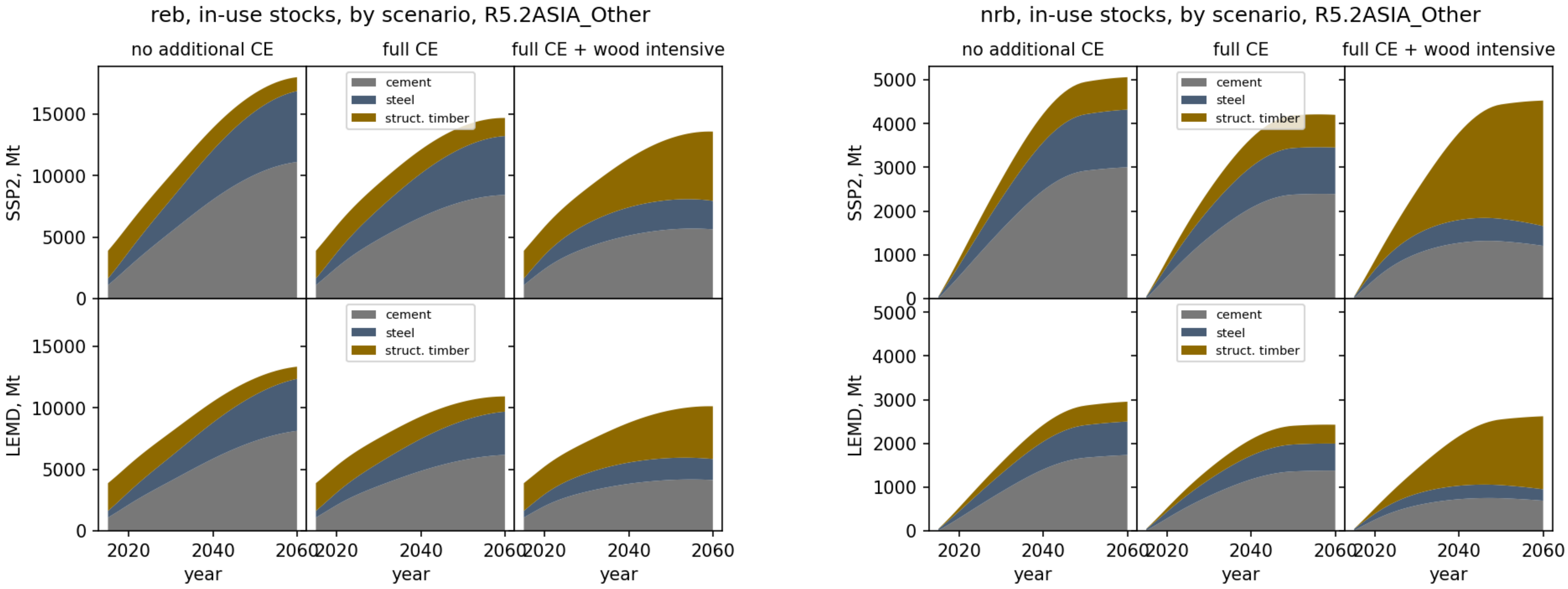

Overview time series of material stock by region, material, sector, and scenario (Fig. SP7)

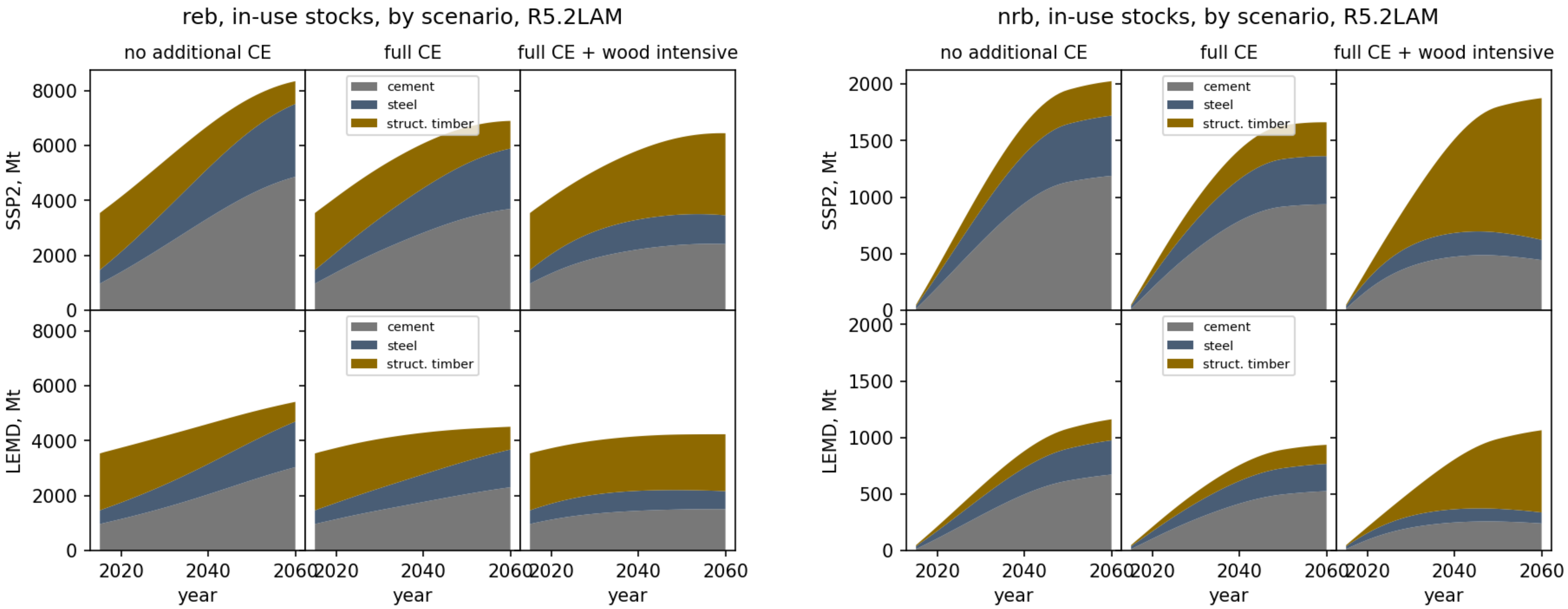

Overview time series of material stock by region, material, sector, and scenario (Fig. SP7)

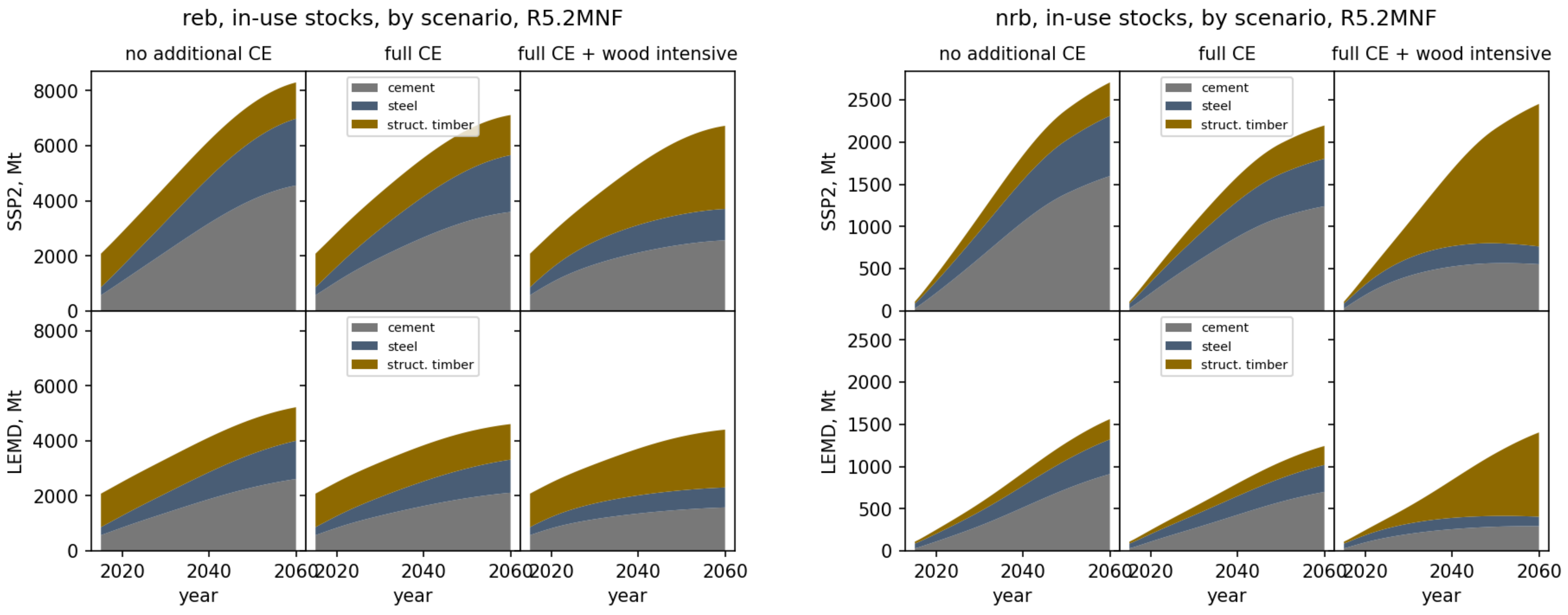

Overview time series of material stock by region, material, sector, and scenario (Fig. SP7)

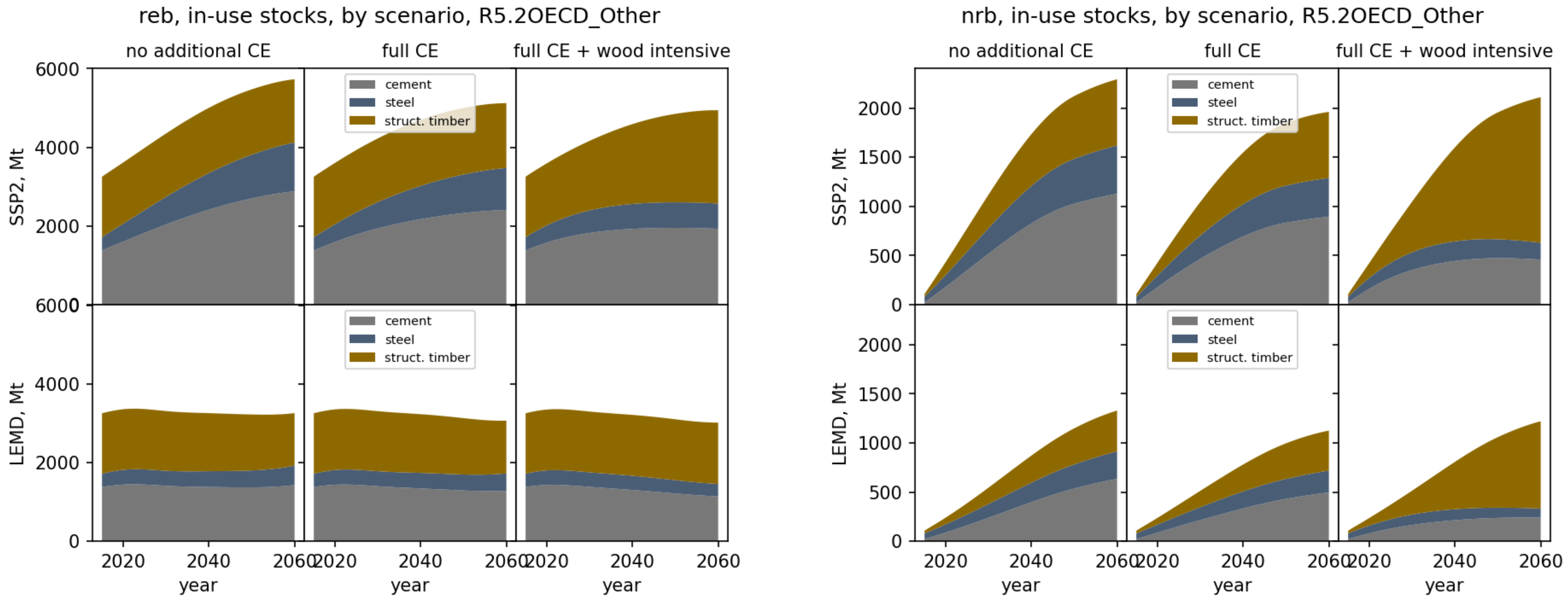

Overview time series of material stock by region, material, sector, and scenario (Fig. SP7)

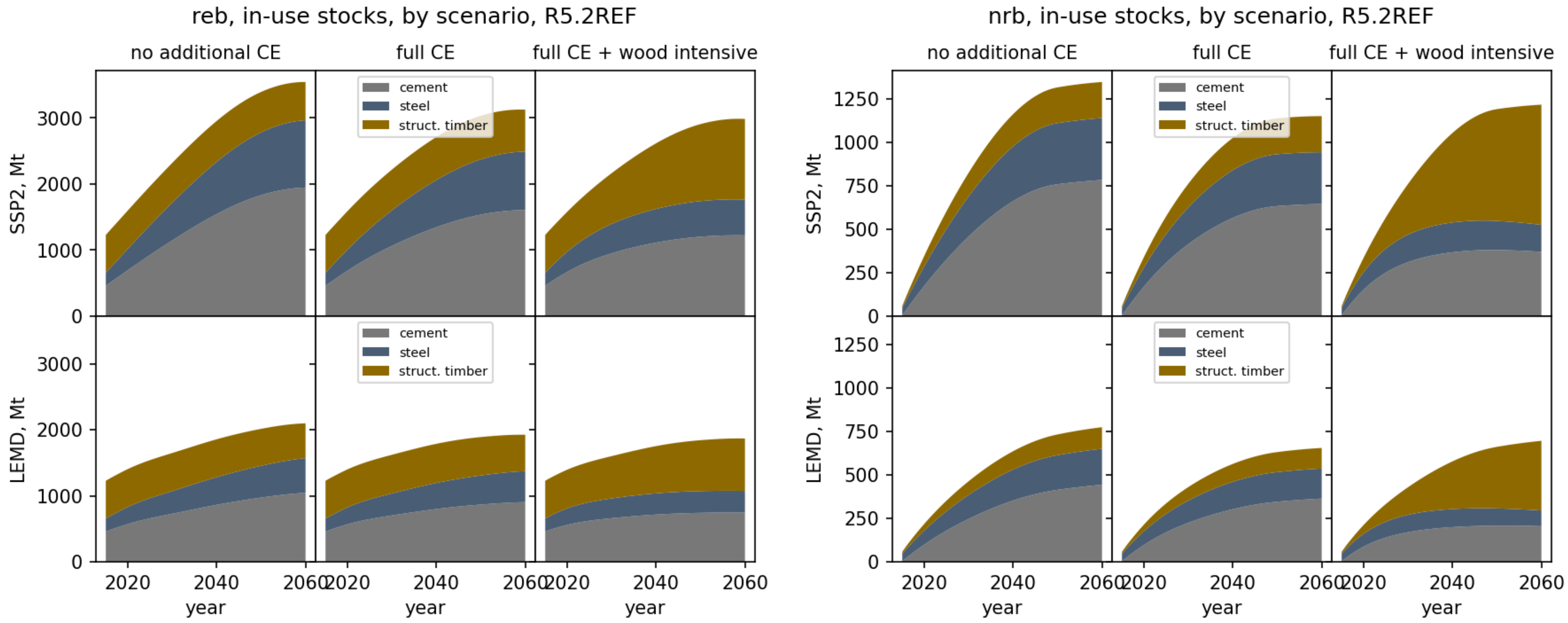

Overview time series of material stock by region, material, sector, and scenario (Fig. SP7)

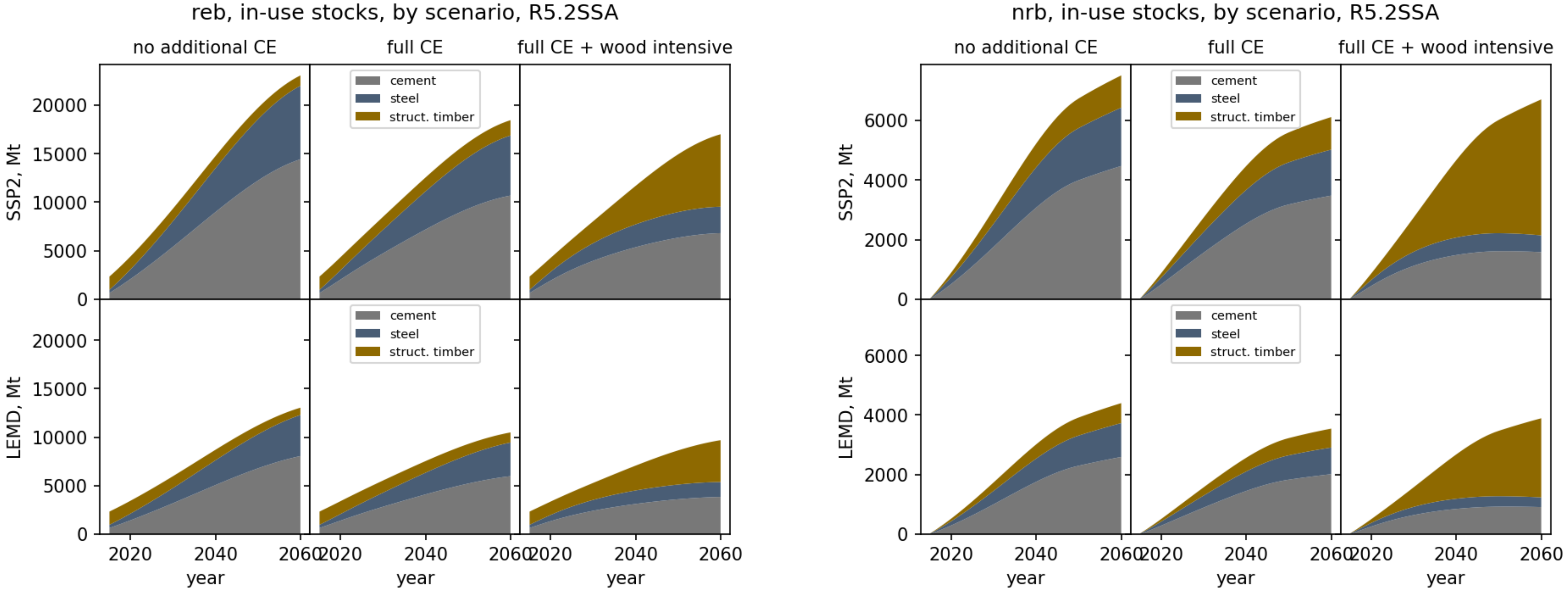

Overview time series of material stock by region, material, sector, and scenario (Fig. SP7)

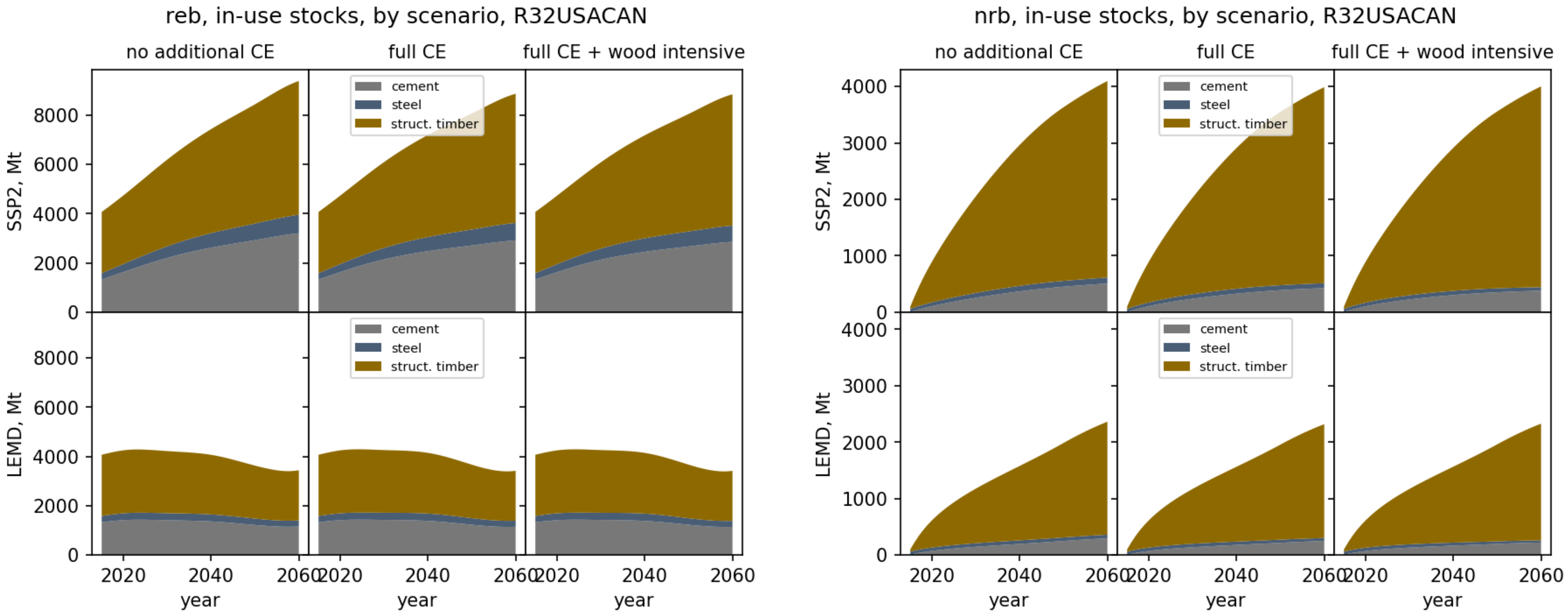

Overview time series of material stock by region, material, sector, and scenario (Fig. SP7)

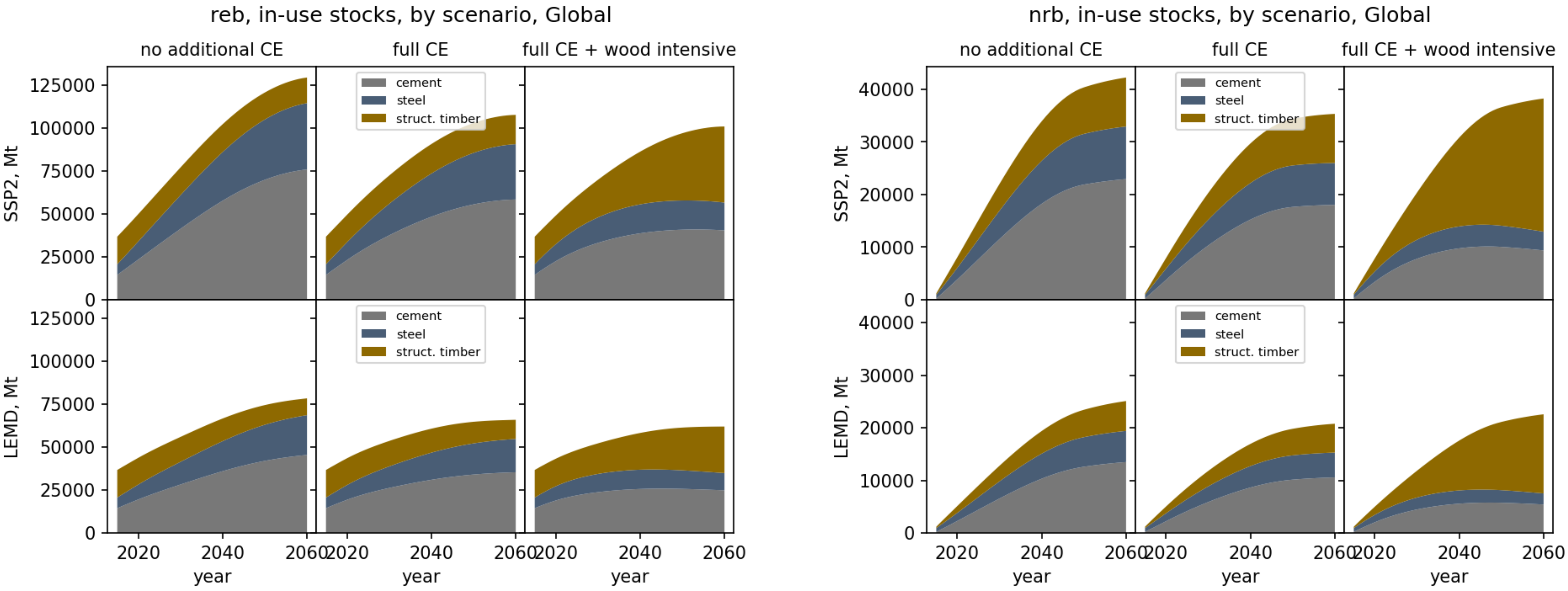

Results: Material production and recycling by region (Fig. SP8)

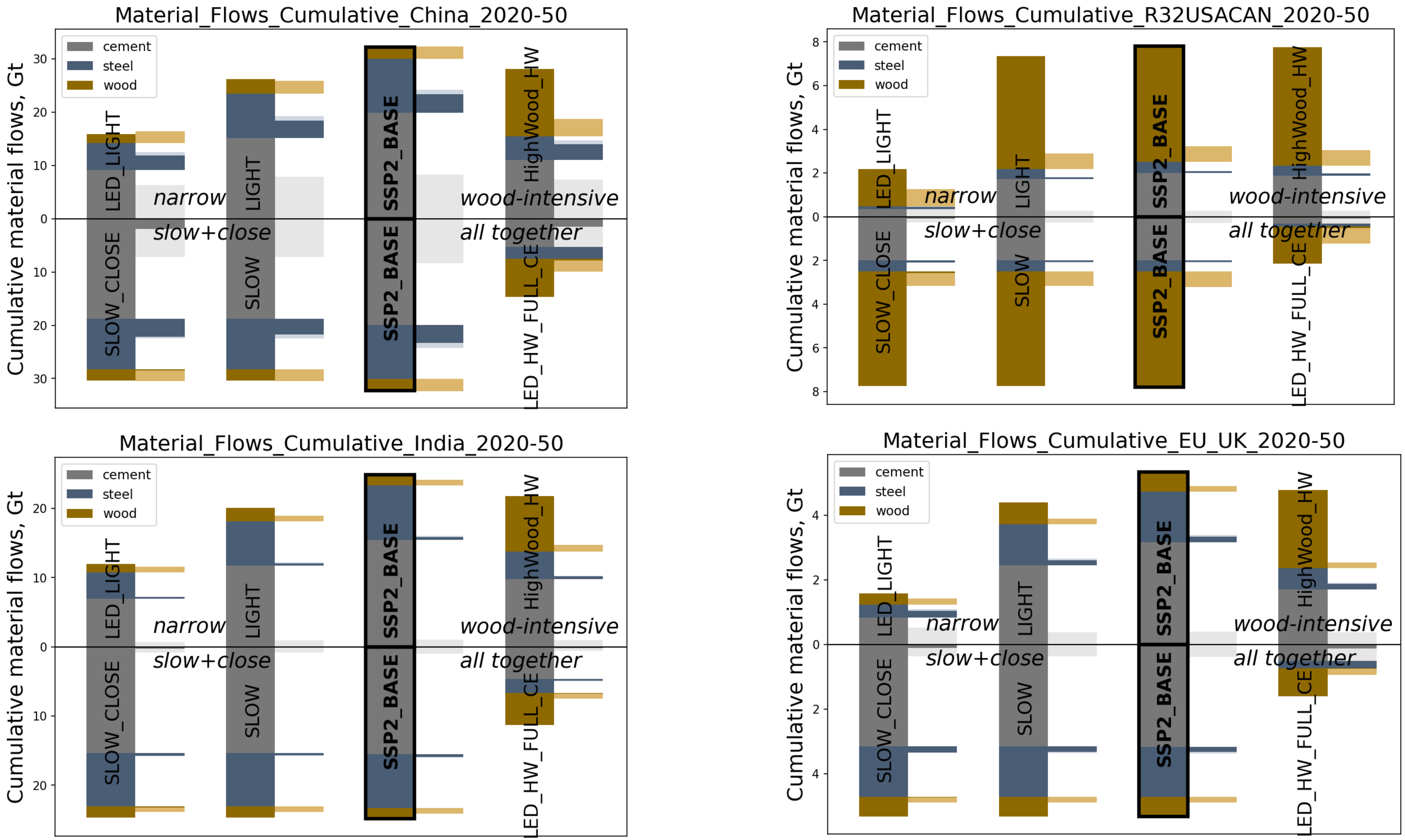

Source: RECCv2.5 model calculation

Results: Material production and recycling by region (Fig. SP8)

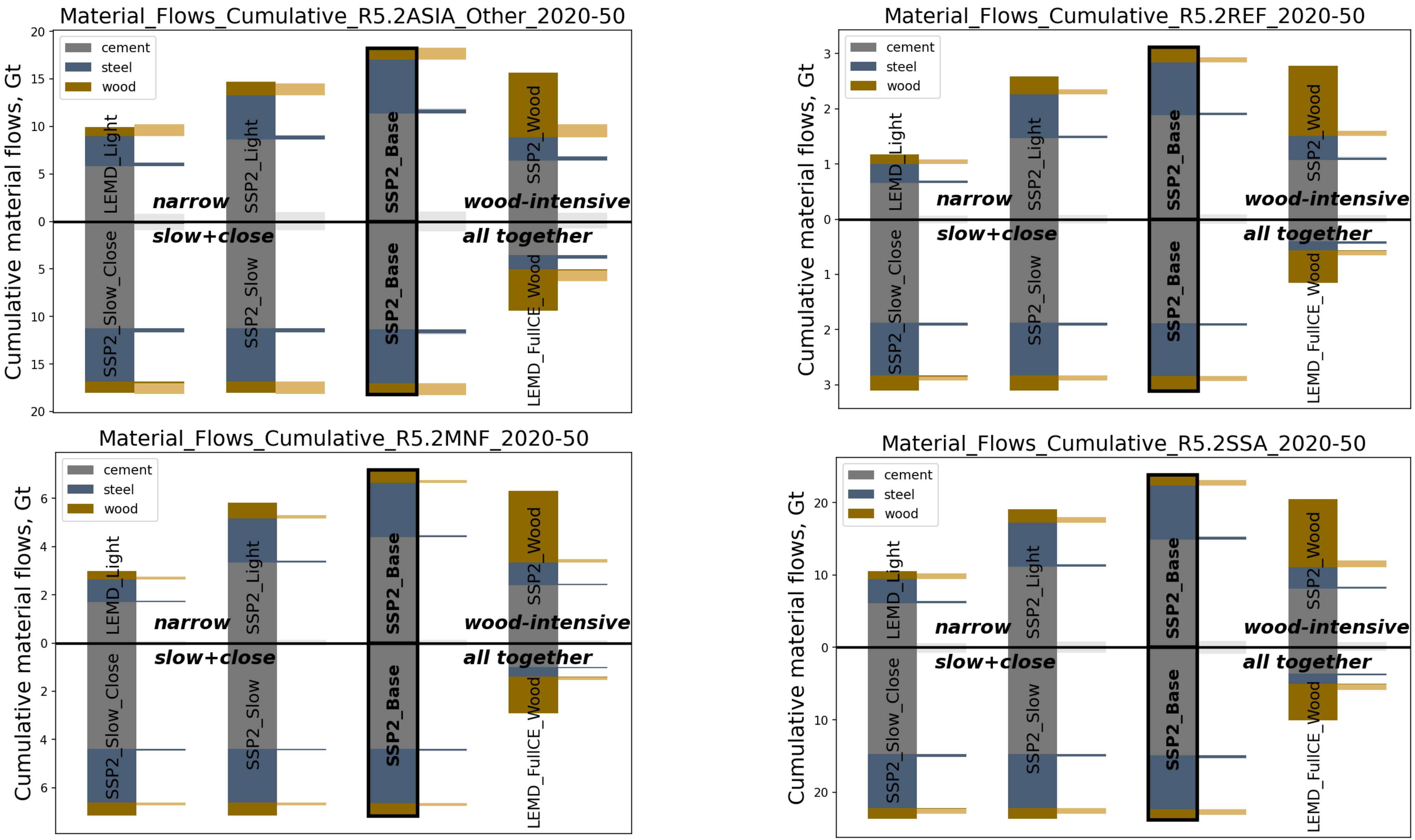

Source: RECCv2.5 model calculation

Results: Material production and recycling by region (Fig. SP8)

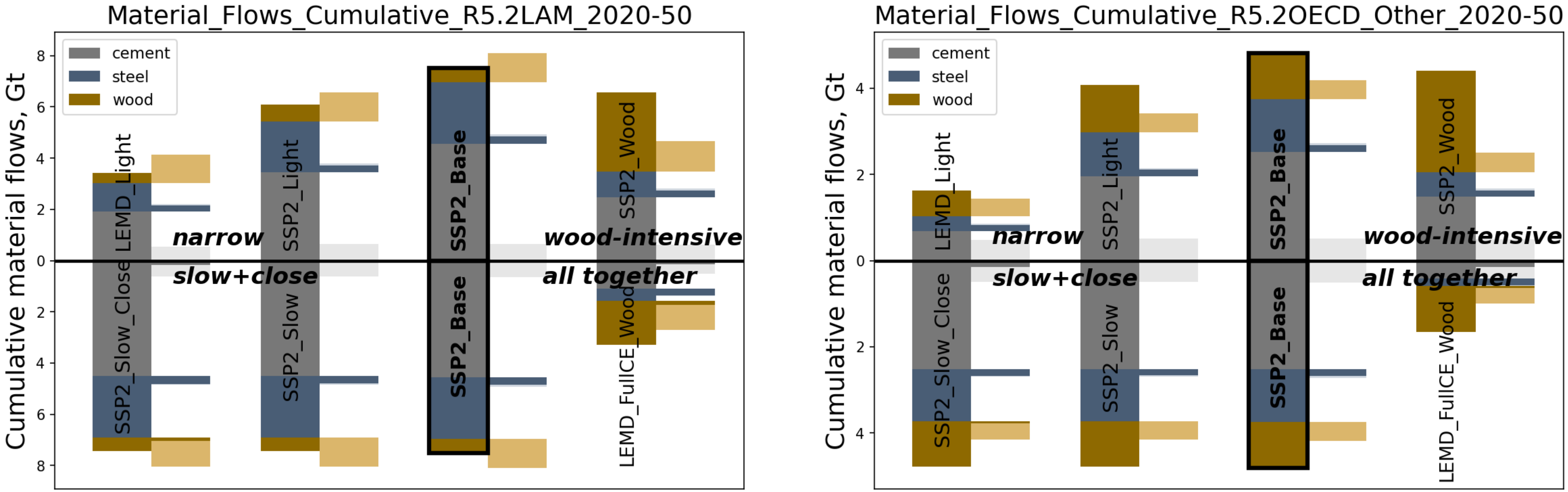

Source: RECCv2.5 model calculation

Results: Material production and recycling by region (Fig. SP8)

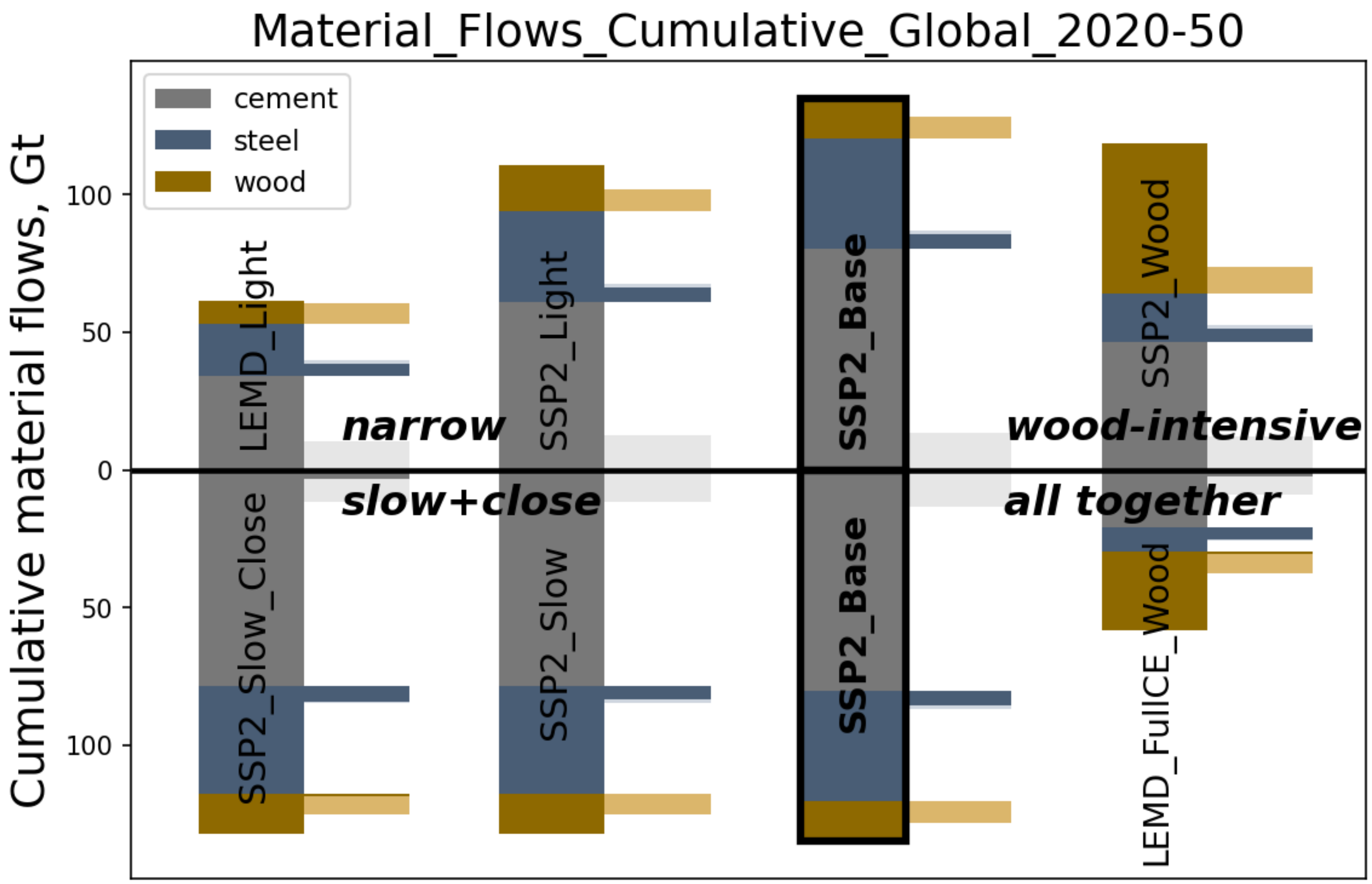

Source: RECCv2.5 model calculation

Results: Energy demand by region (Fig. SP9)

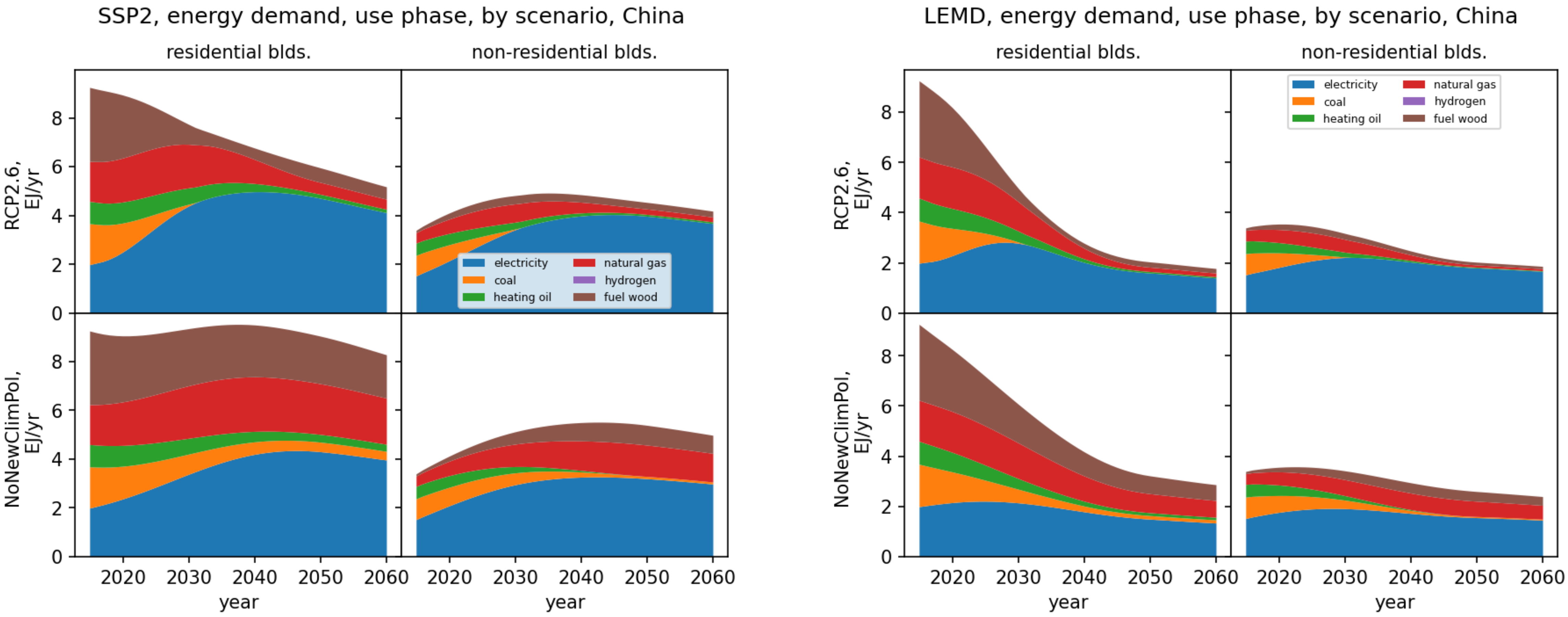

Source: RECCv2.5 model calculation

Results: Energy demand by region (Fig. SP9)

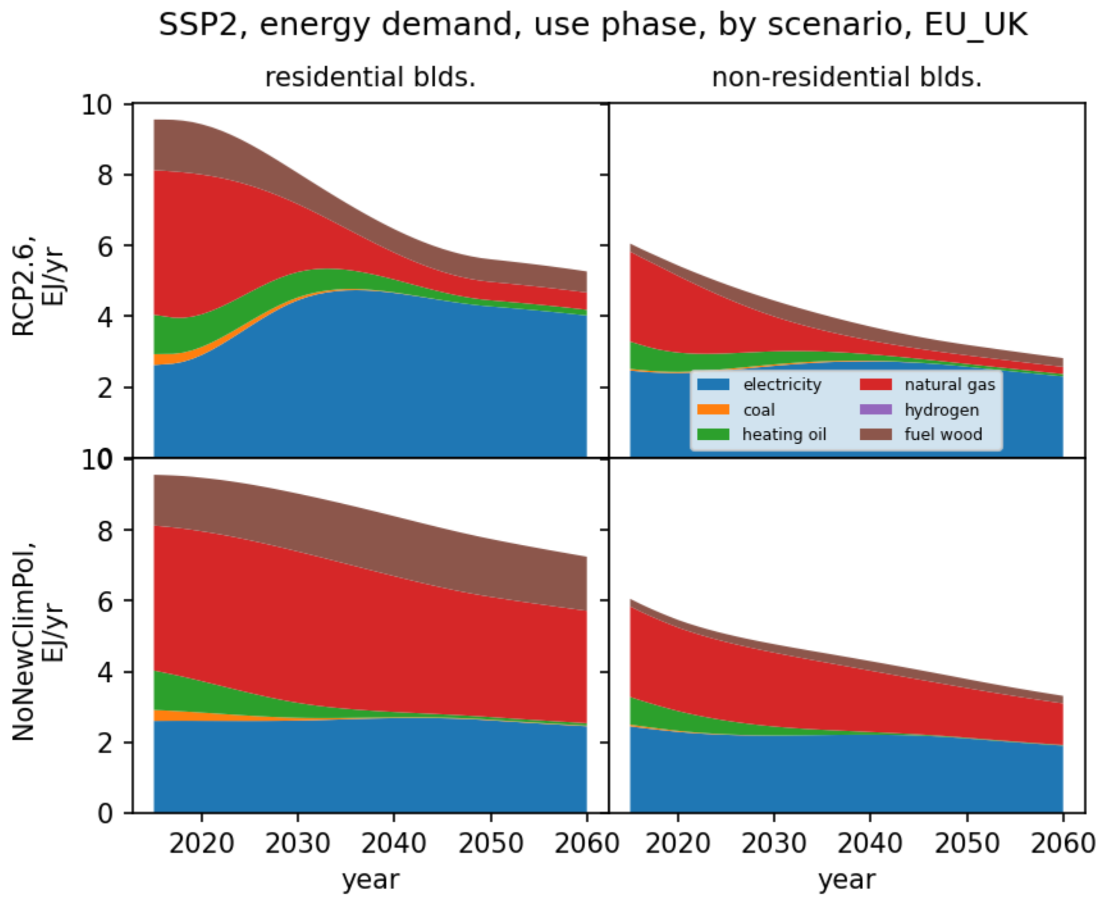

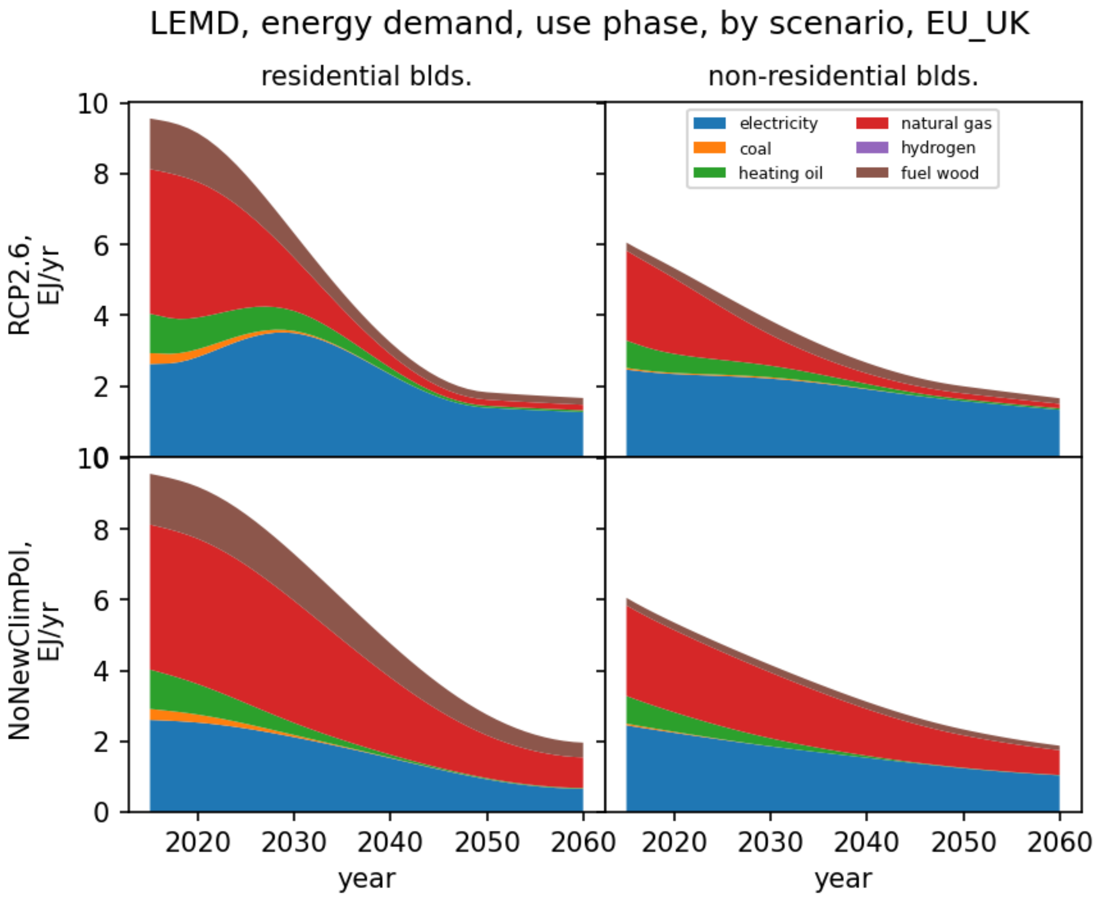

Source: RECCv2.5 model calculation

Results: Energy demand by region (Fig. SP9)

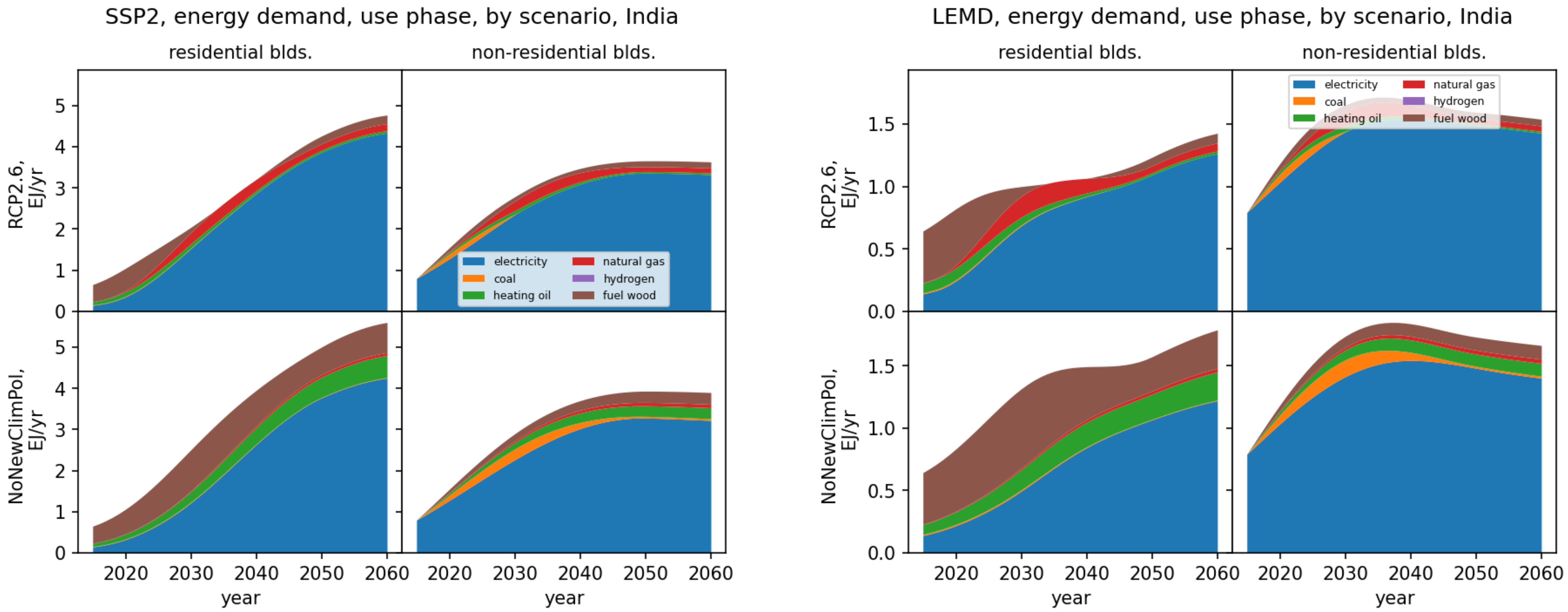

Source: RECCv2.5 model calculation

Results: Energy demand by region (Fig. SP9)

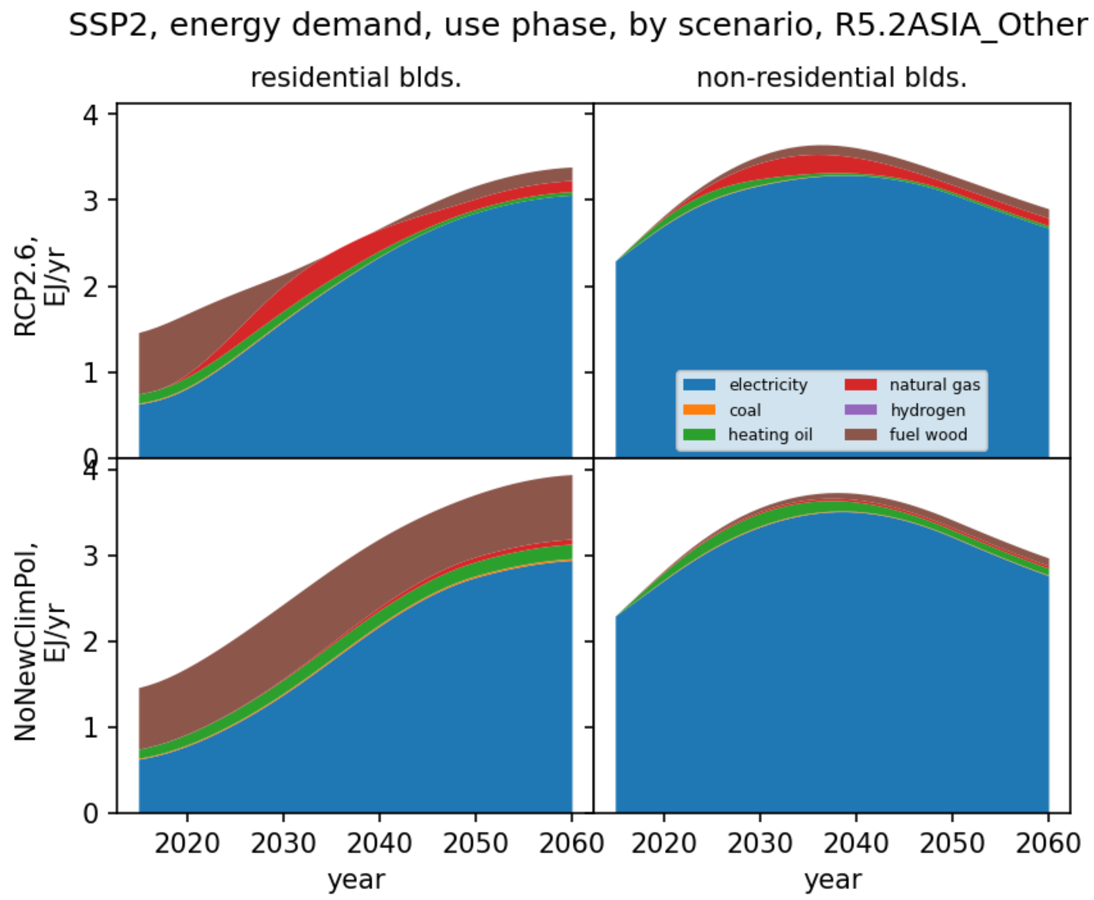

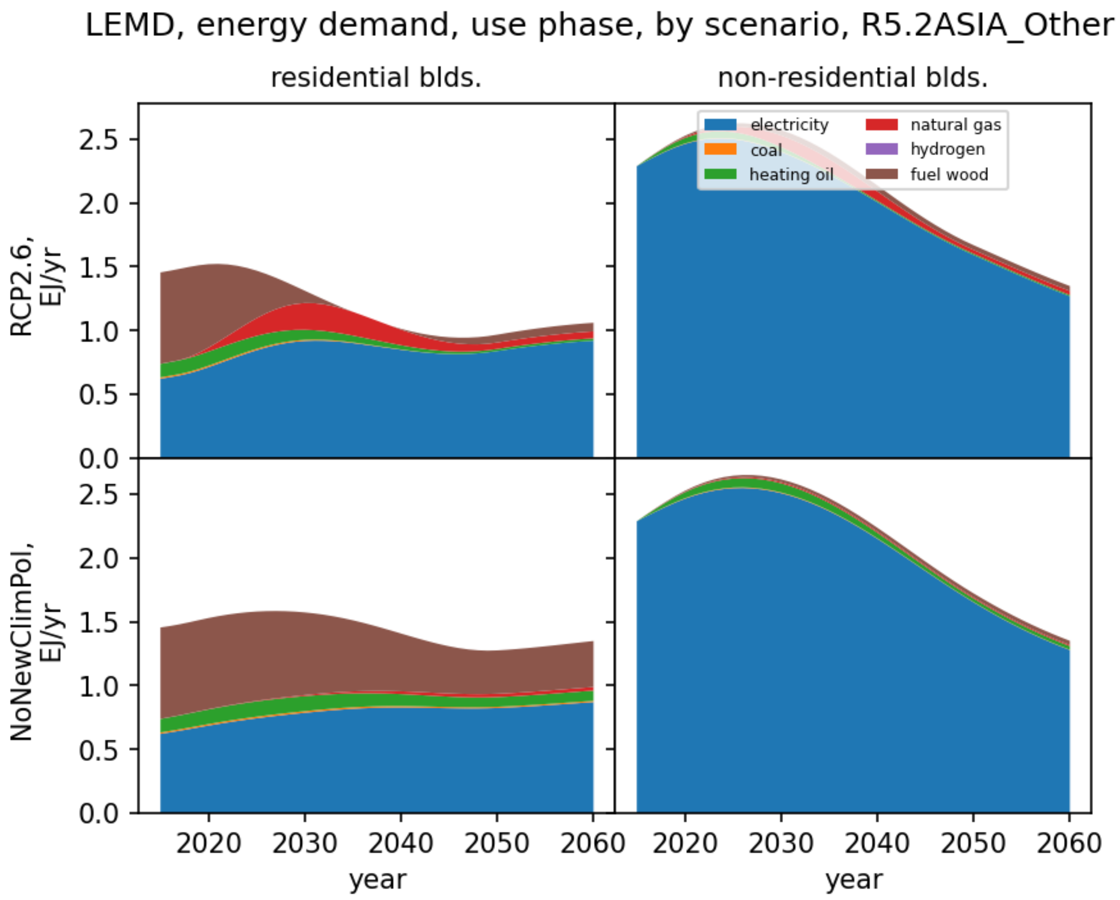

Source: RECCv2.5 model calculation

Results: Energy demand by region (Fig. SP9)

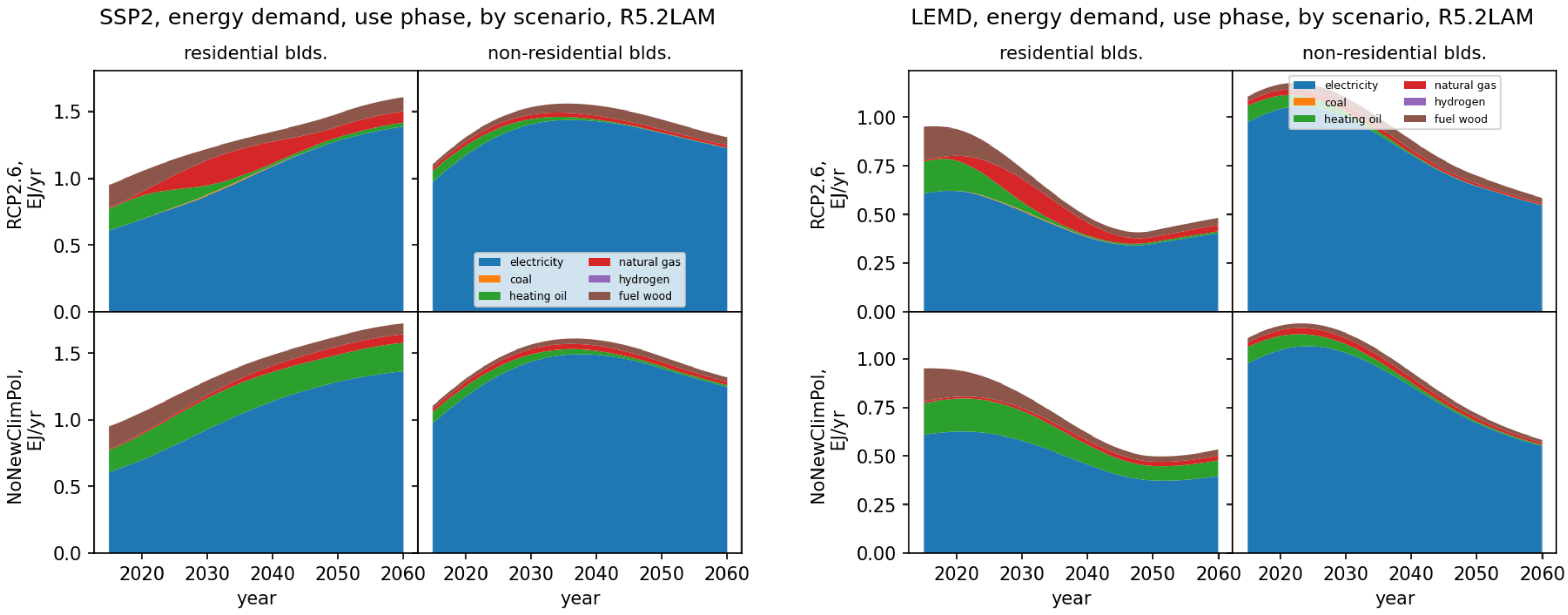

Source: RECCv2.5 model calculation

Results: Energy demand by region (Fig. SP9)

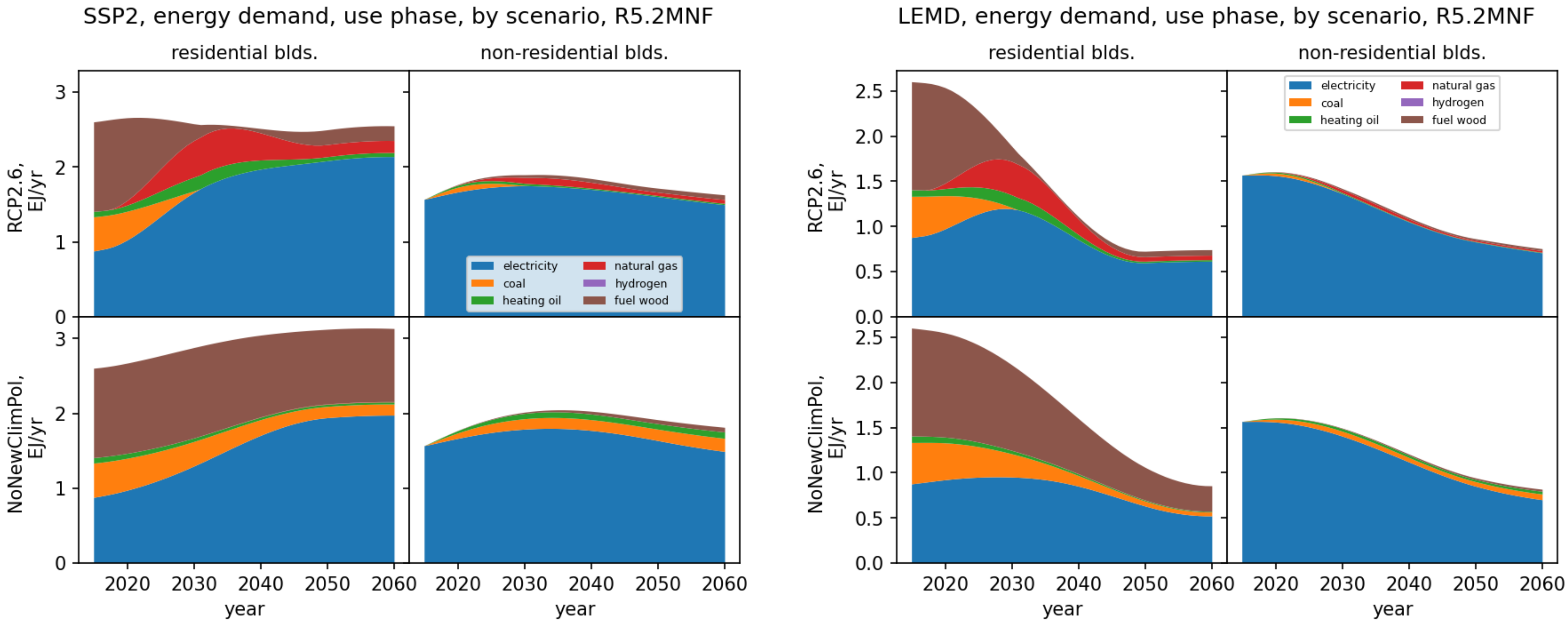

Source: RECCv2.5 model calculation

Results: Energy demand by region (Fig. SP9)

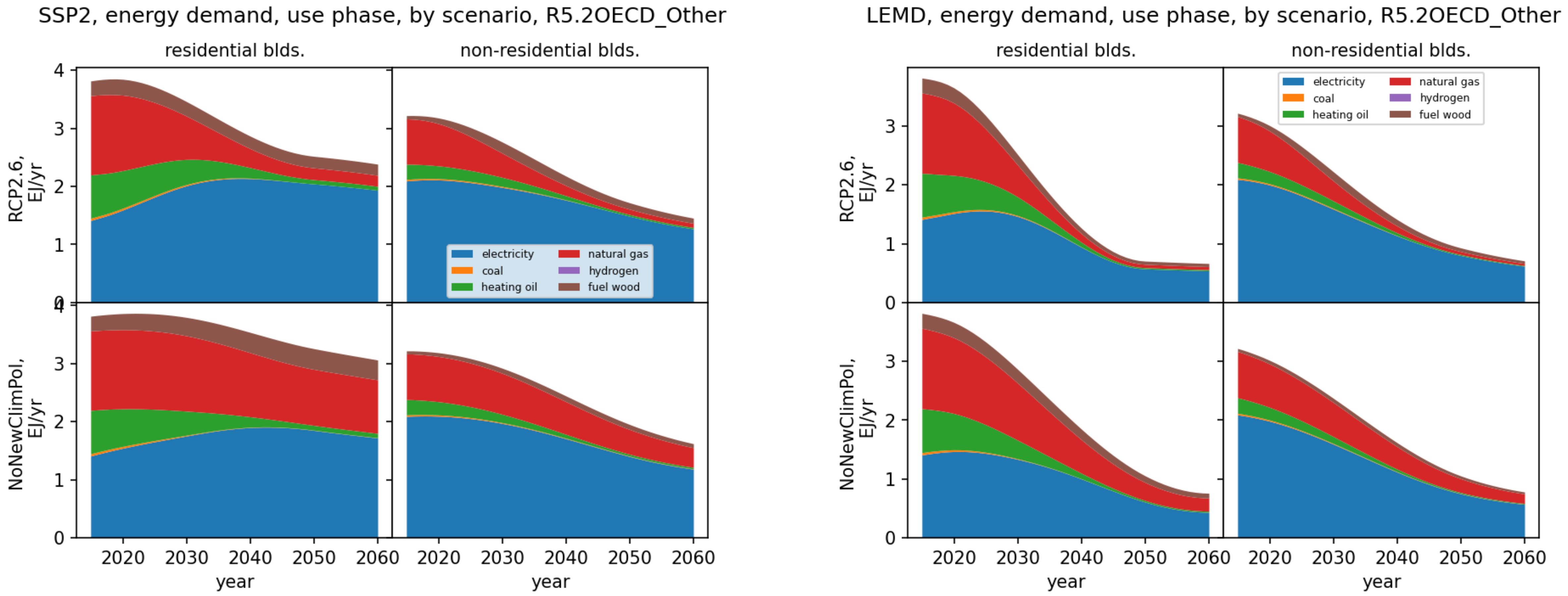

Source: RECCv2.5 model calculation

Results: Energy demand by region (Fig. SP9)

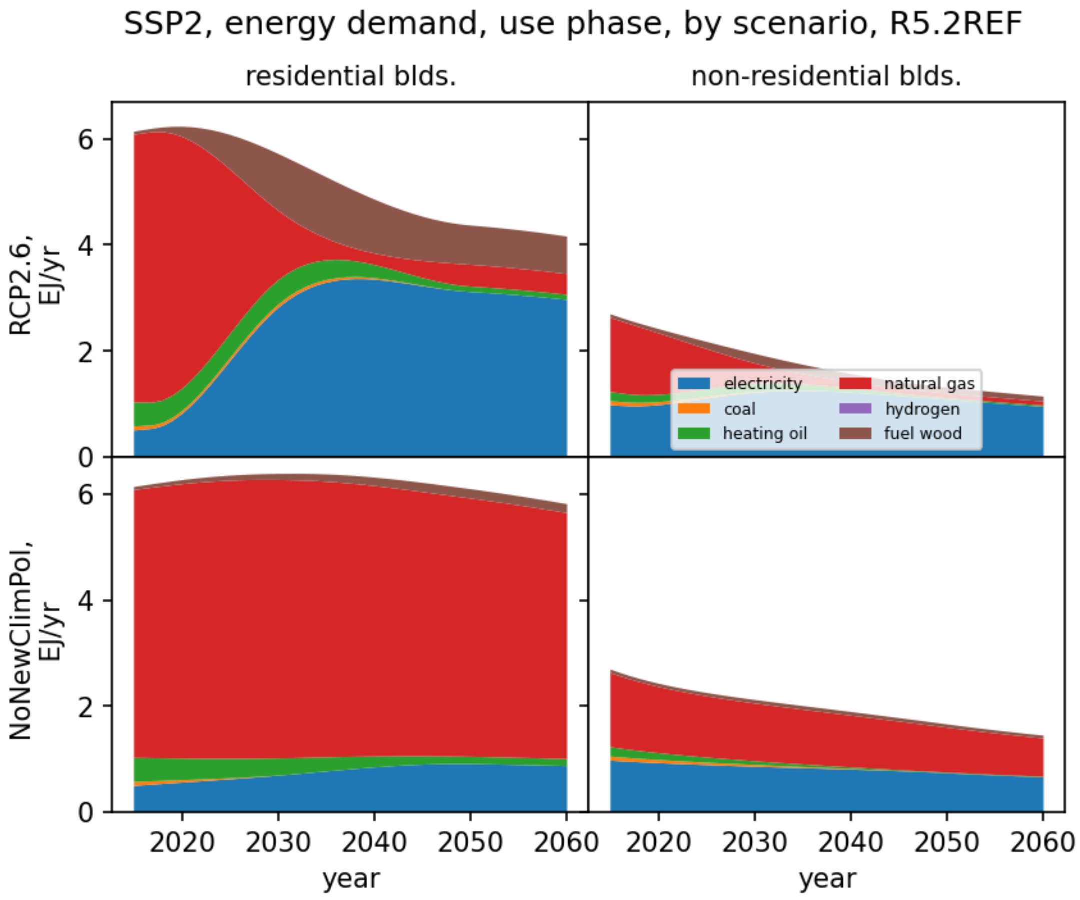

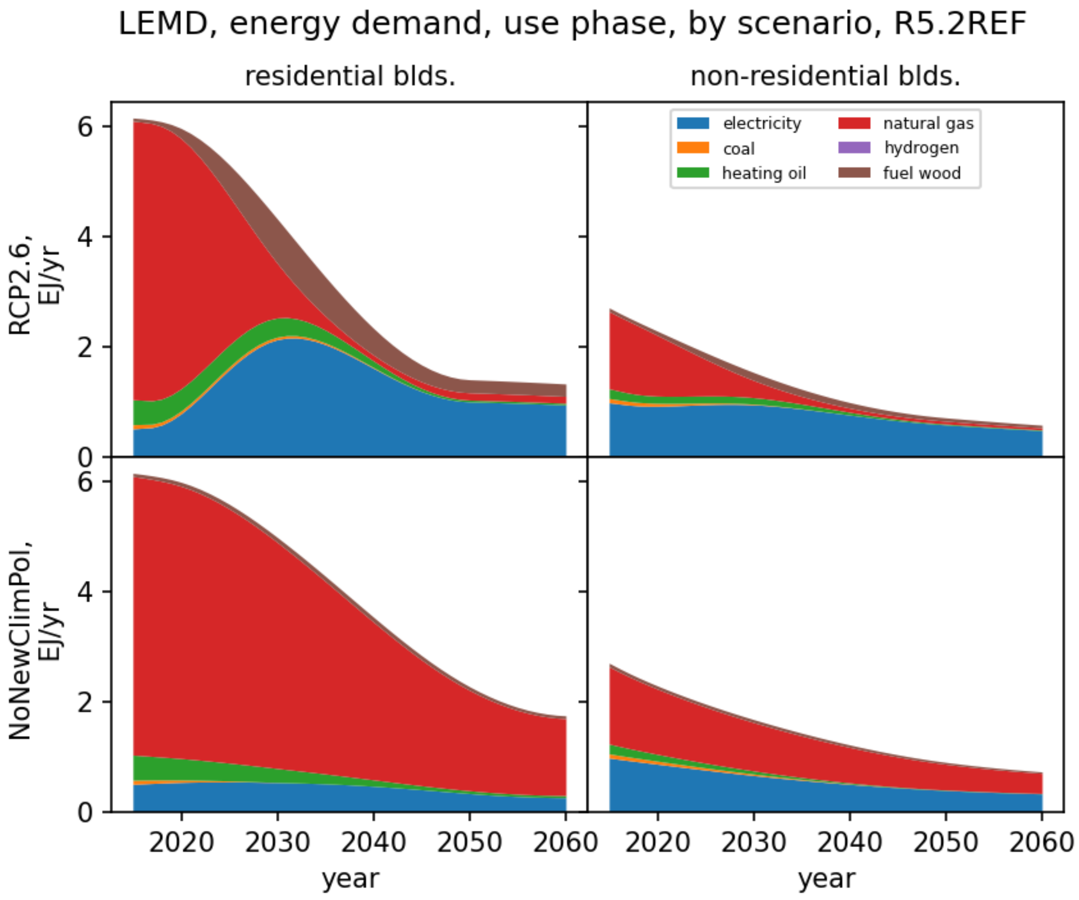

Source: RECCv2.5 model calculation

Results: Energy demand by region (Fig. SP9)

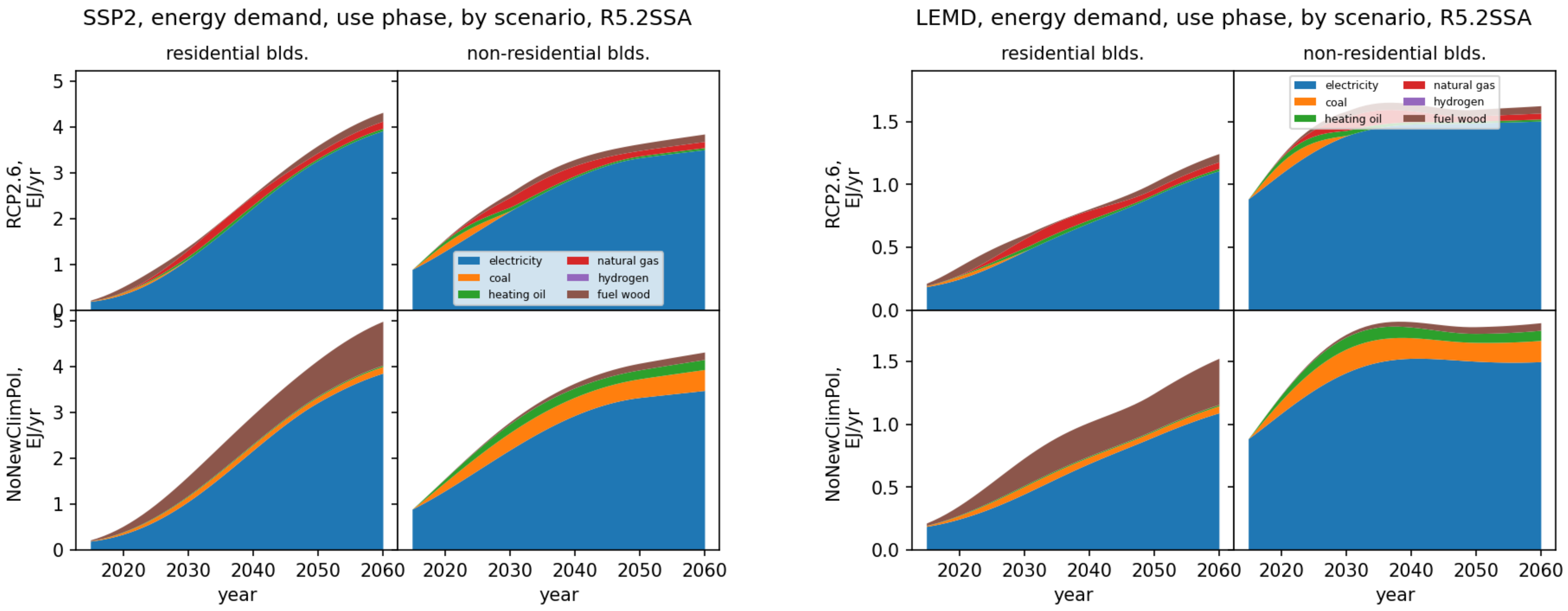

Source: RECCv2.5 model calculation

Results: Energy demand by region (Fig. SP9)

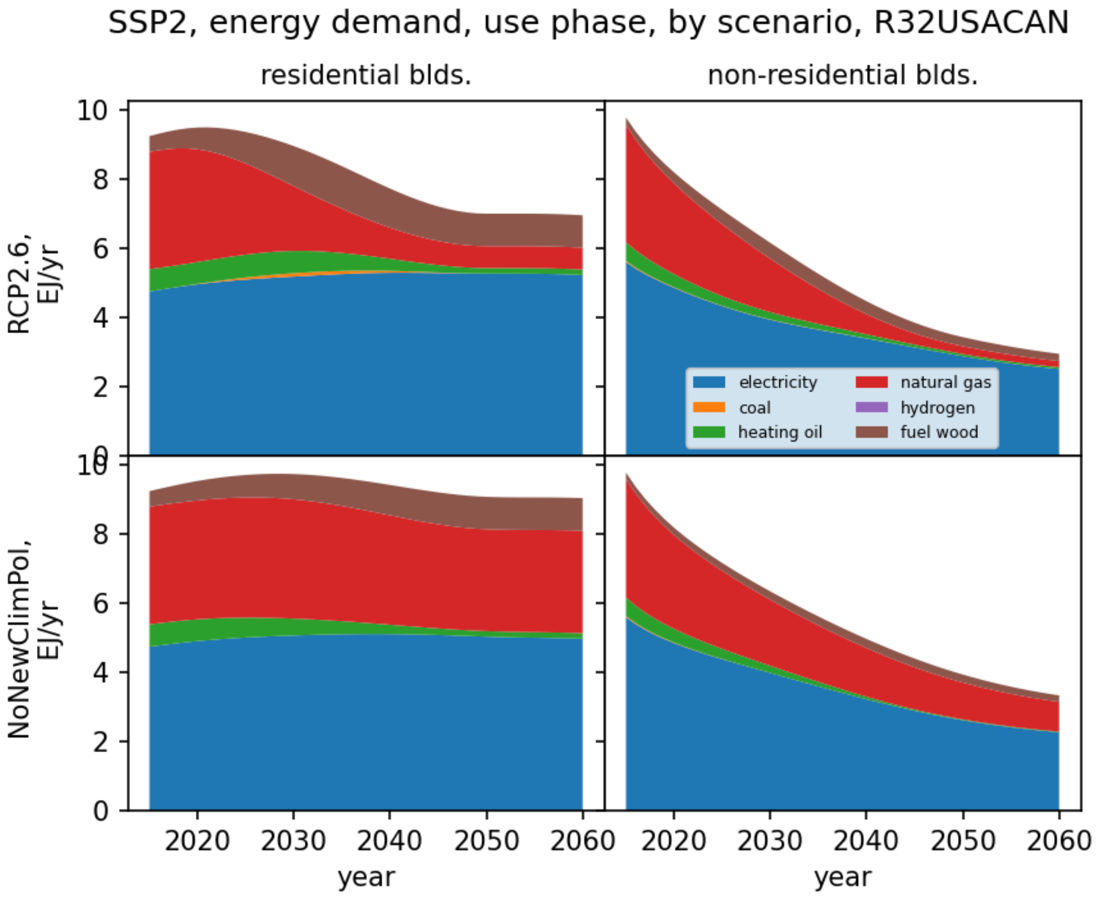

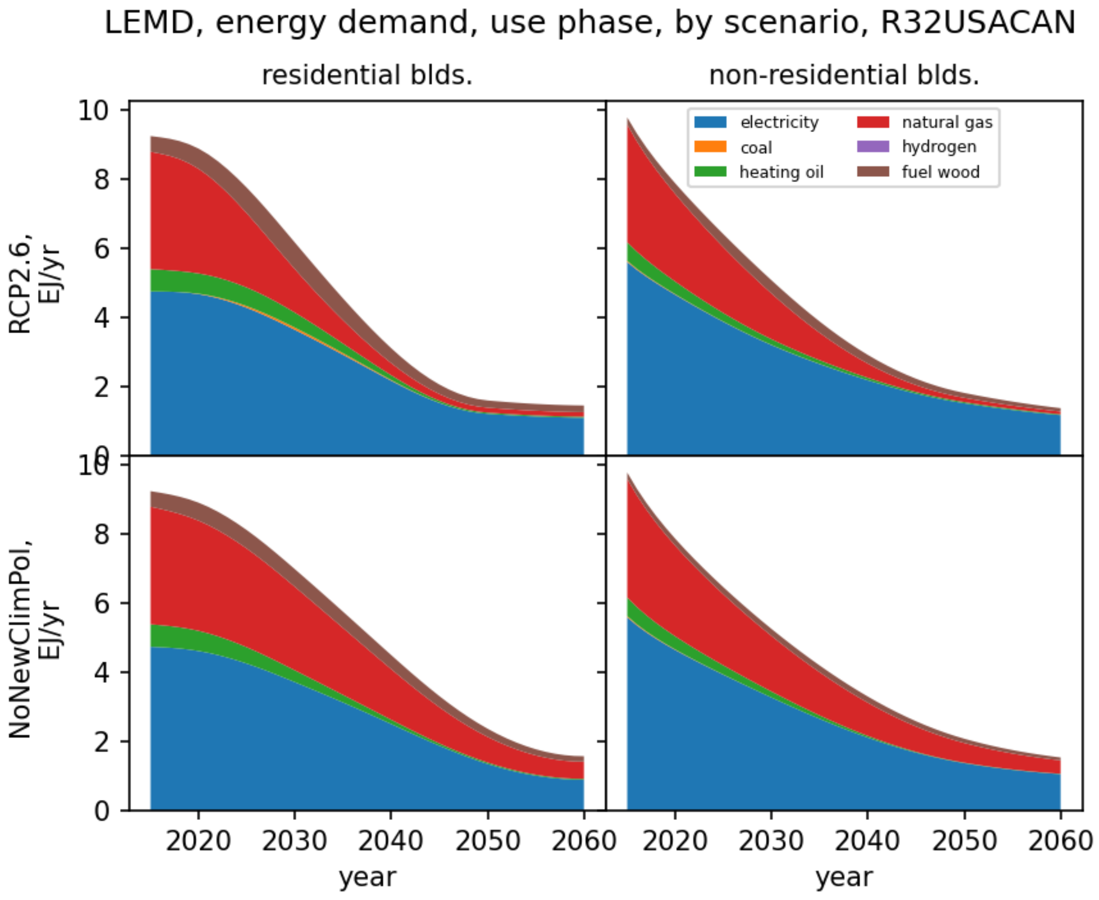

Source: RECCv2.5 model calculation

Results: Energy demand by region (Fig. SP9)

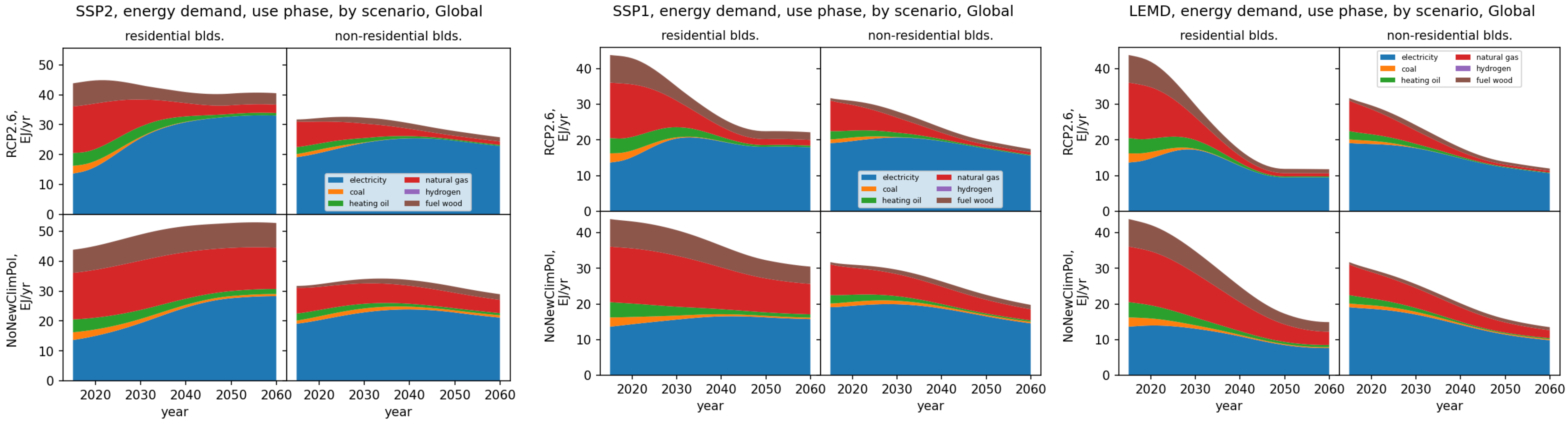

Source: RECCv2.5 model calculation

Results: GHG time series by sector and region (Fig. SP10)

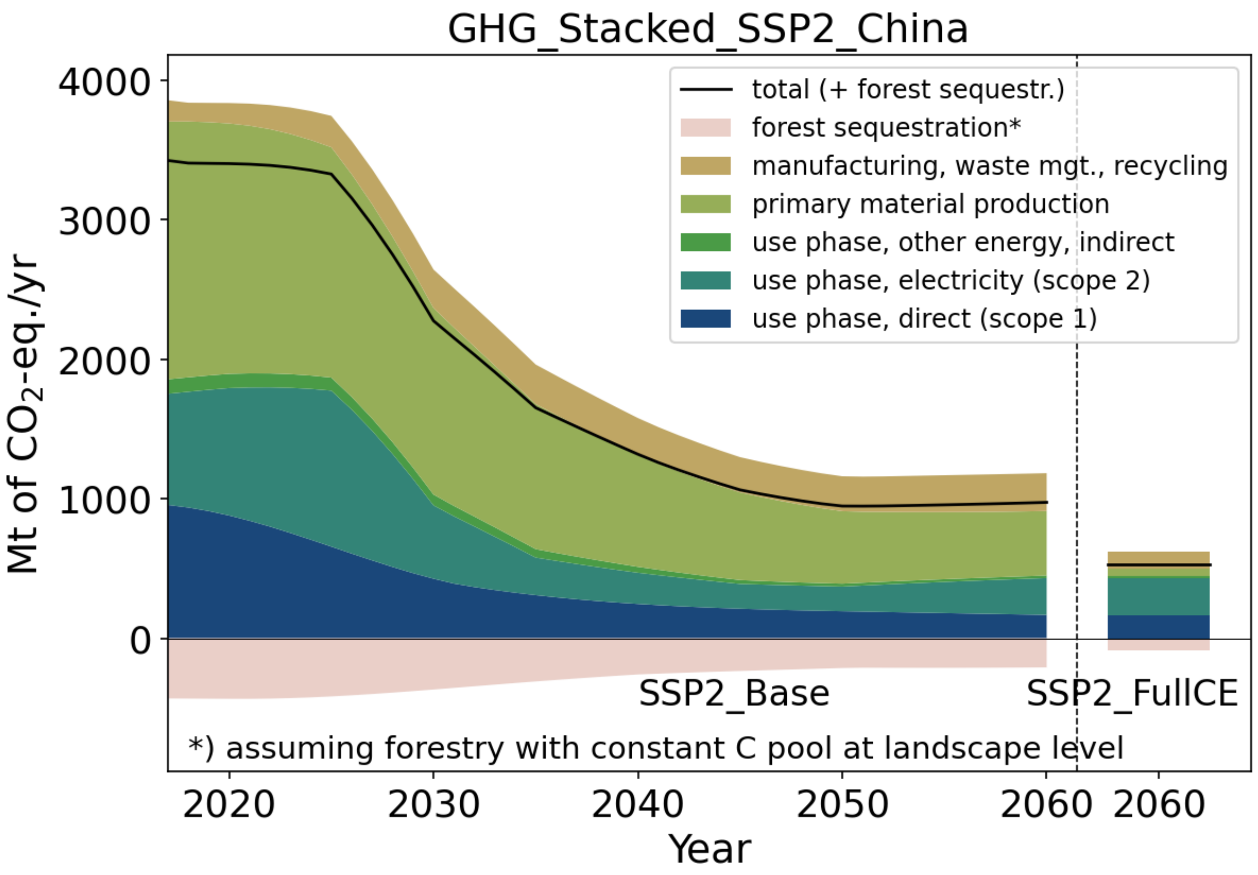

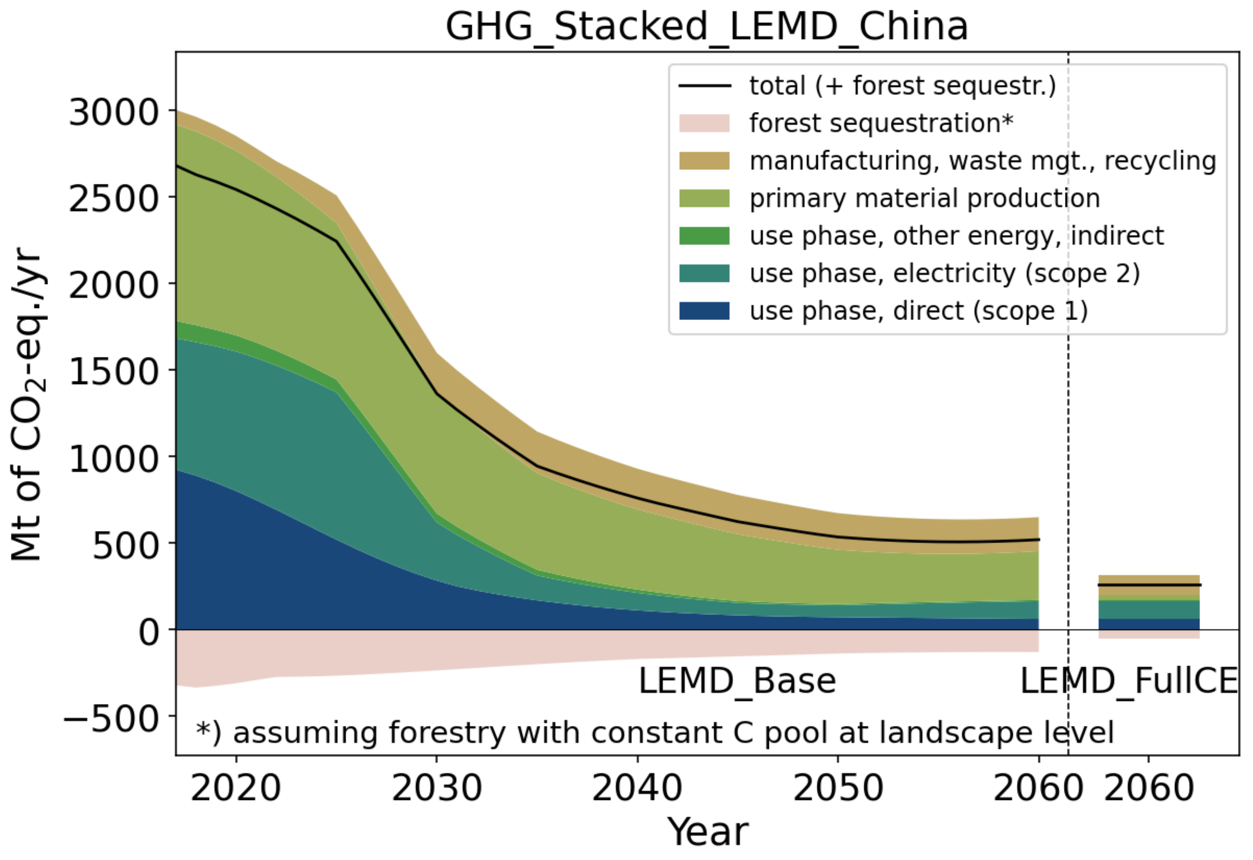

Source: RECCv2.5 model calculation

Results: GHG time series by sector and region (Fig. SP10)

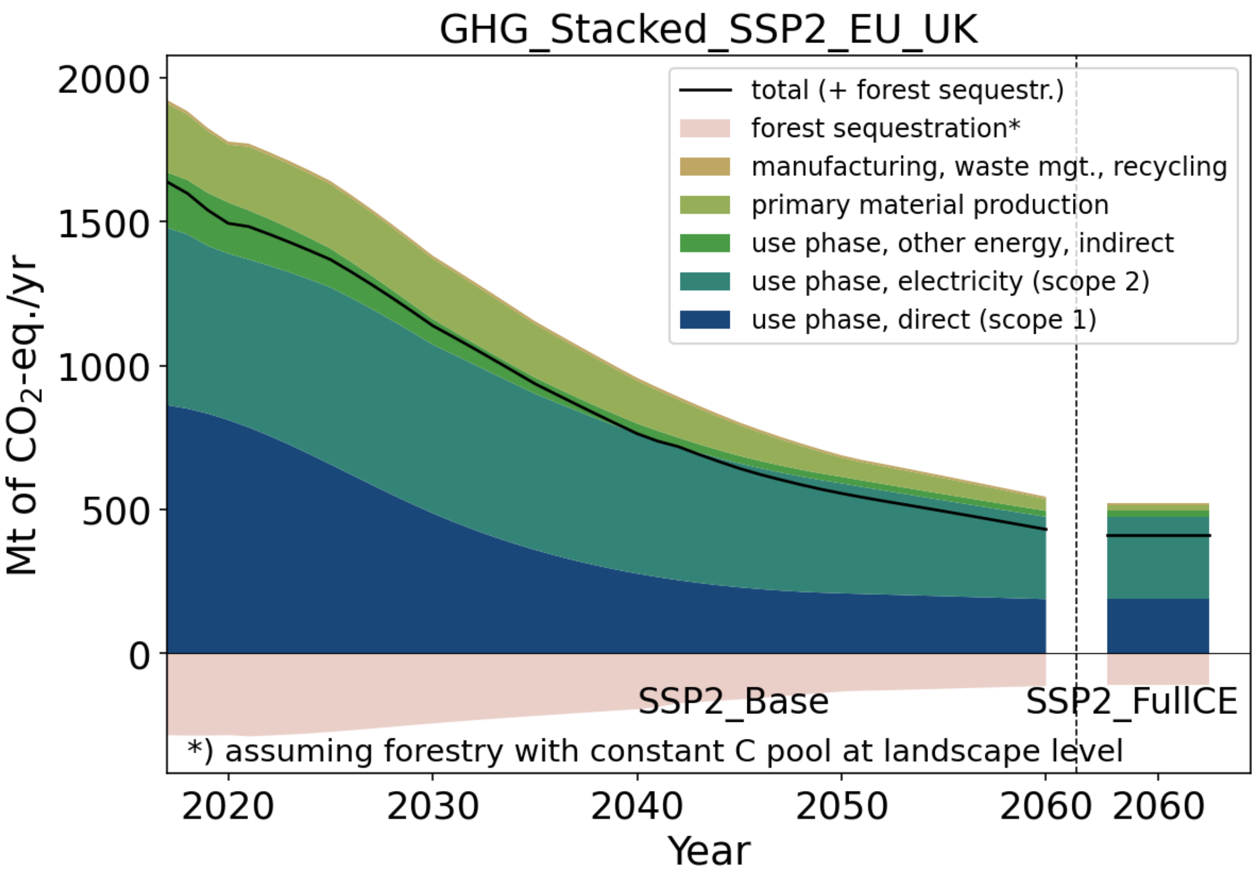

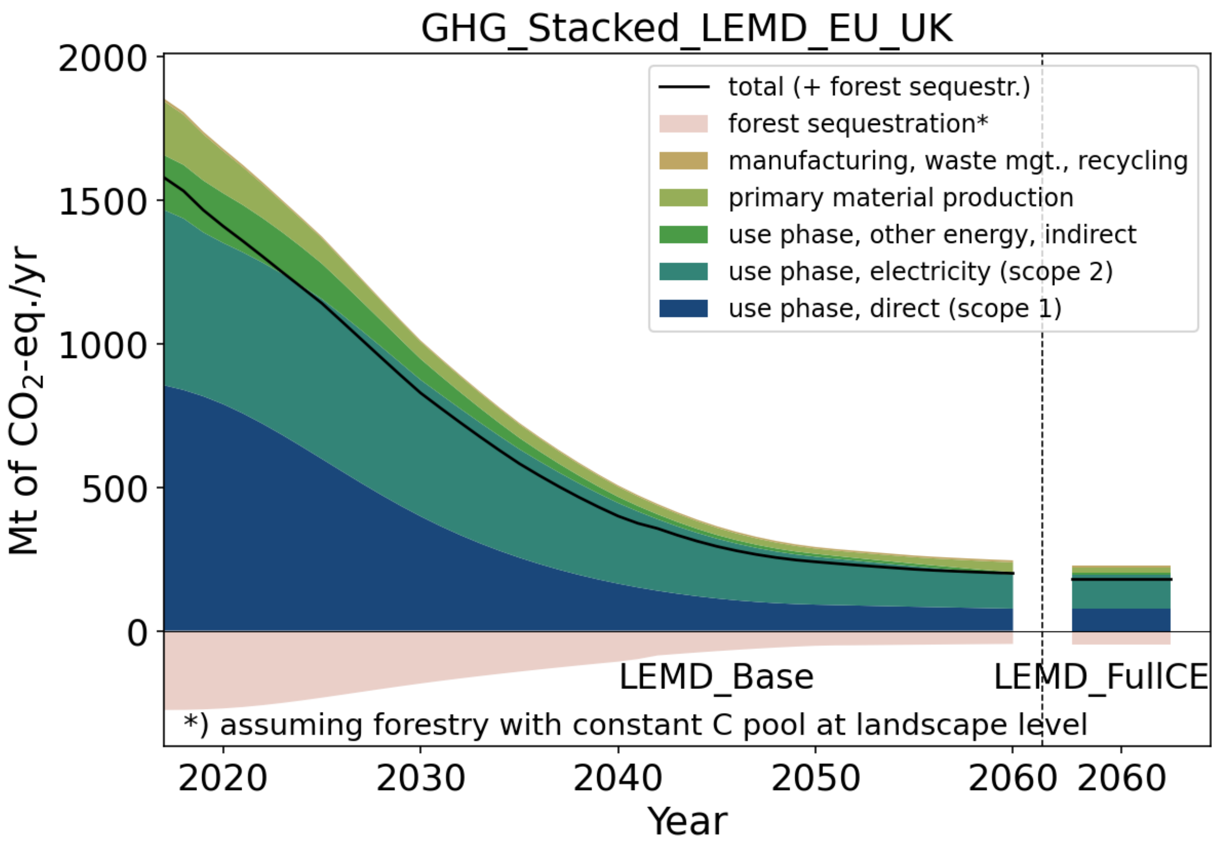

Source: RECCv2.5 model calculation

Results: GHG time series by sector and region (Fig. SP10)

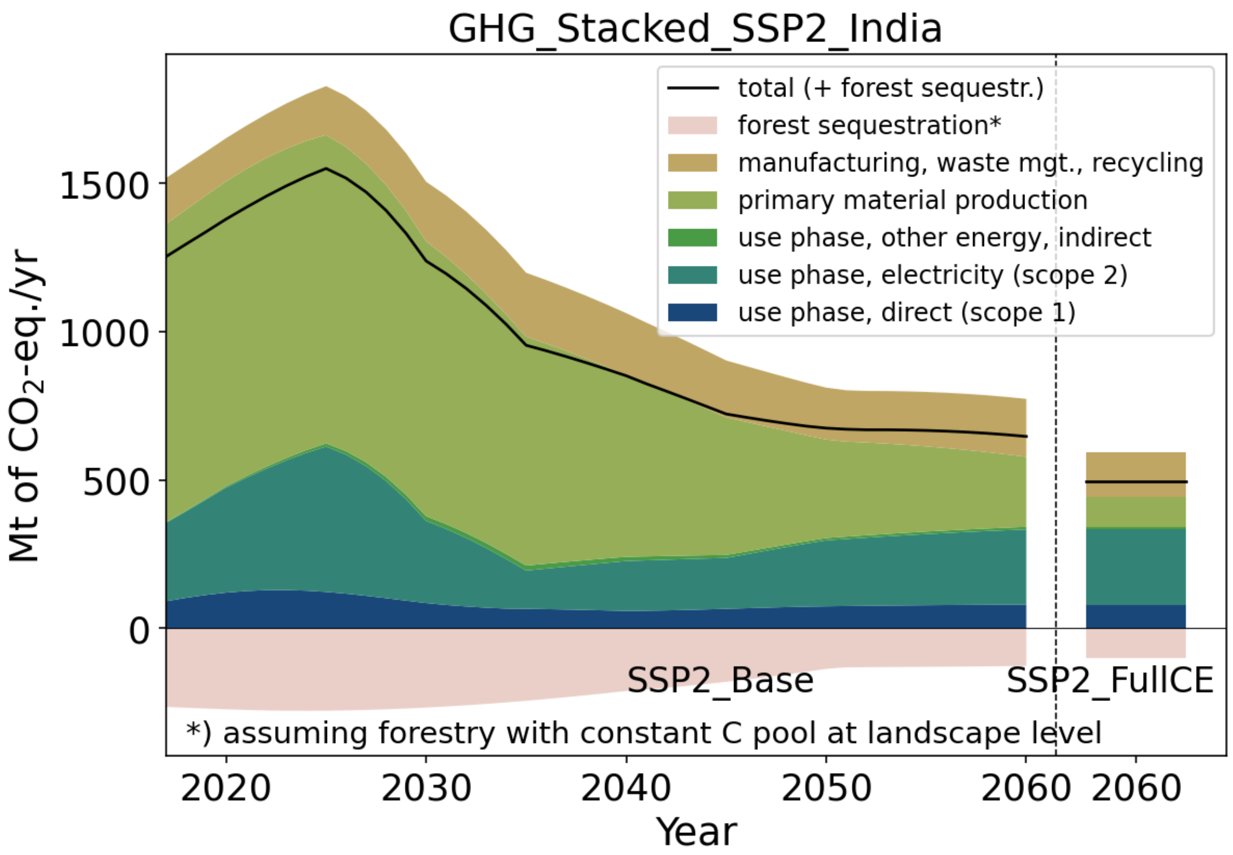

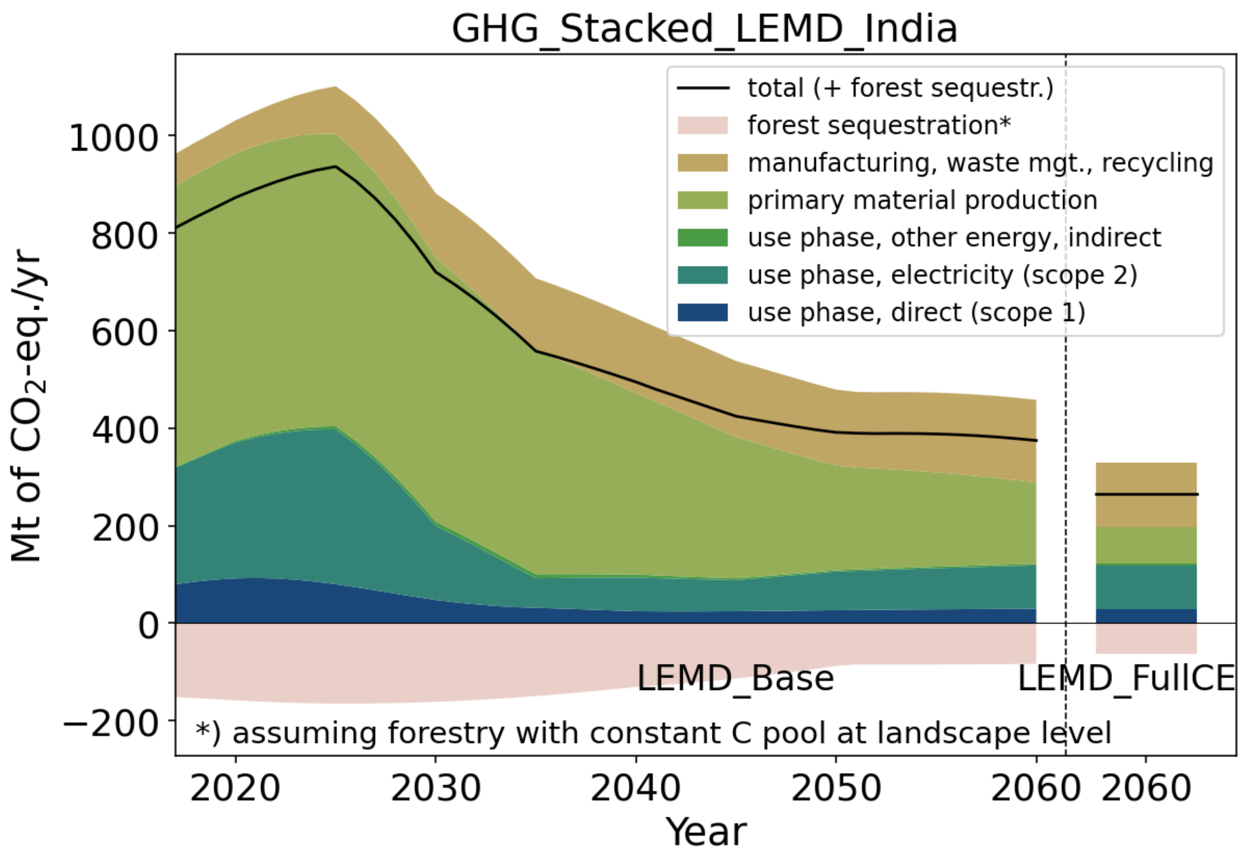

Source: RECCv2.5 model calculation

Results: GHG time series by sector and region (Fig. SP10)

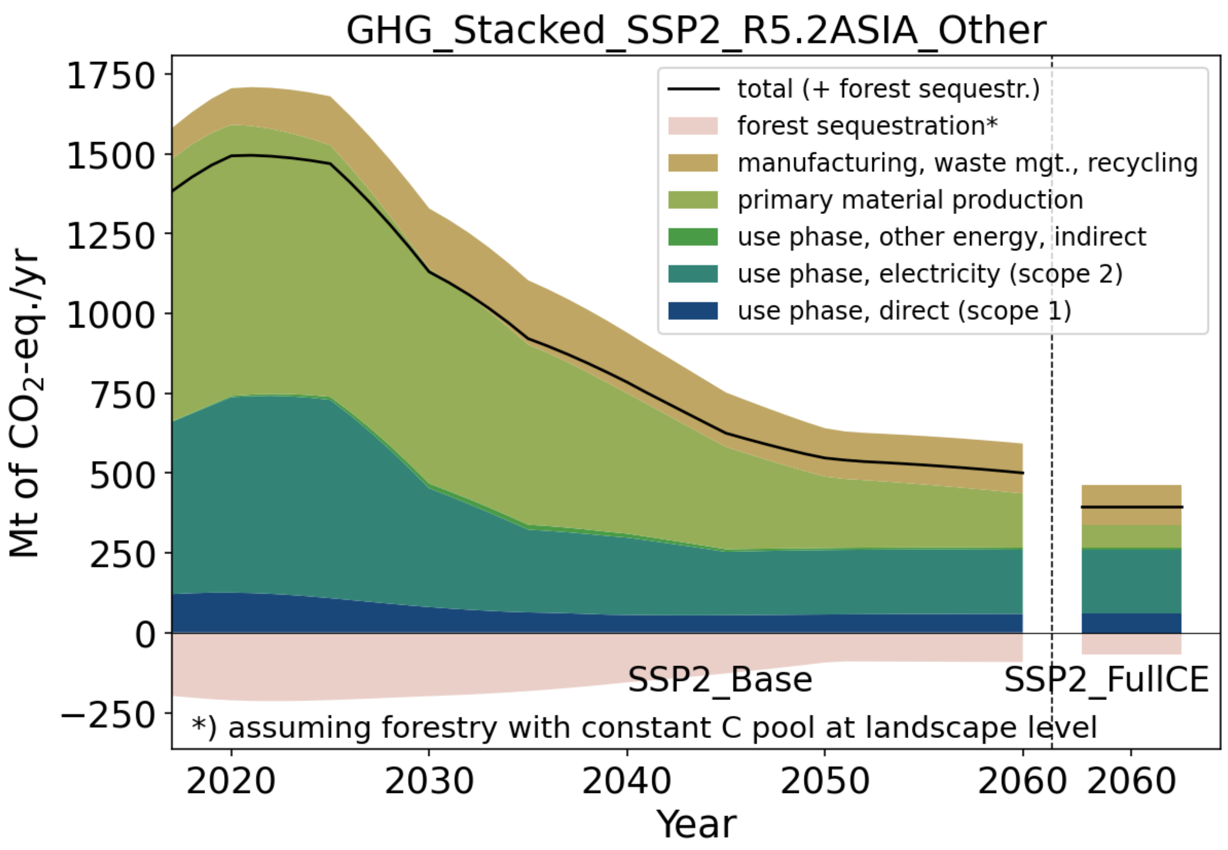

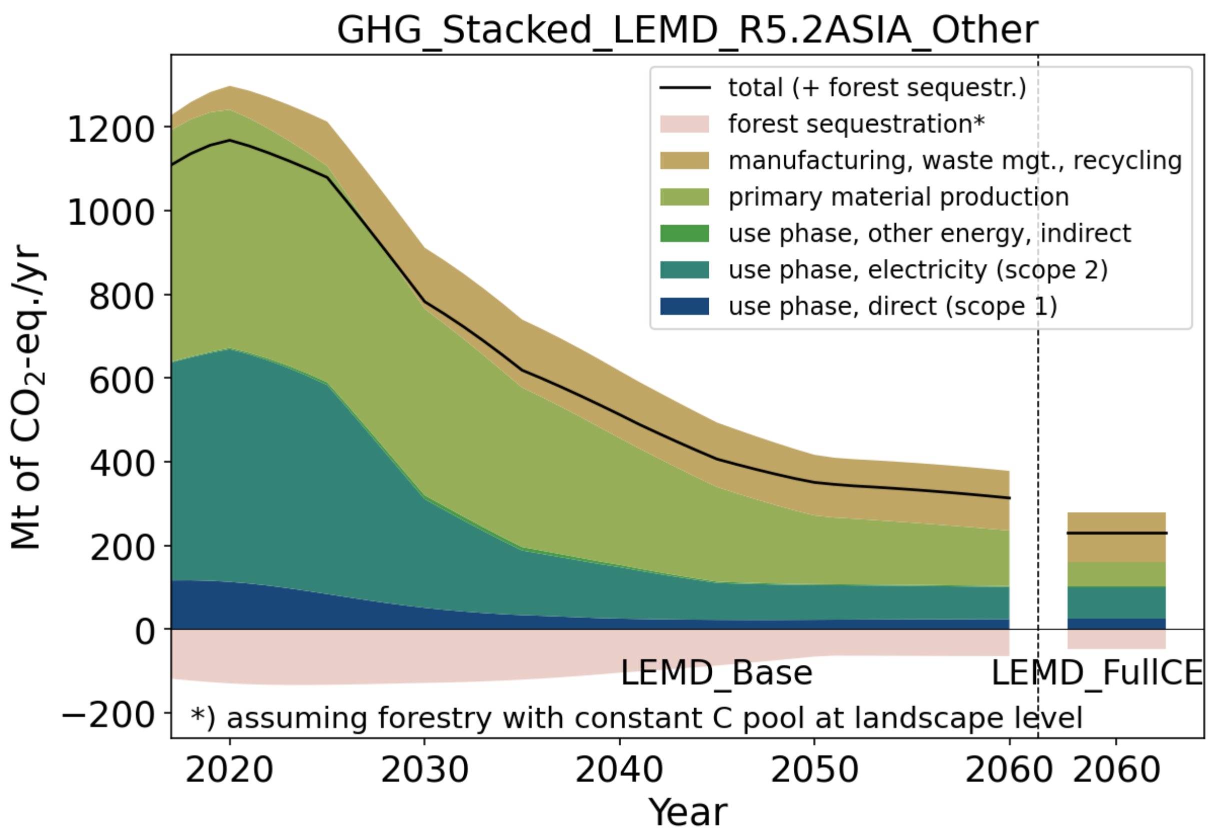

Source: RECCv2.5 model calculation

Results: GHG time series by sector and region (Fig. SP10)

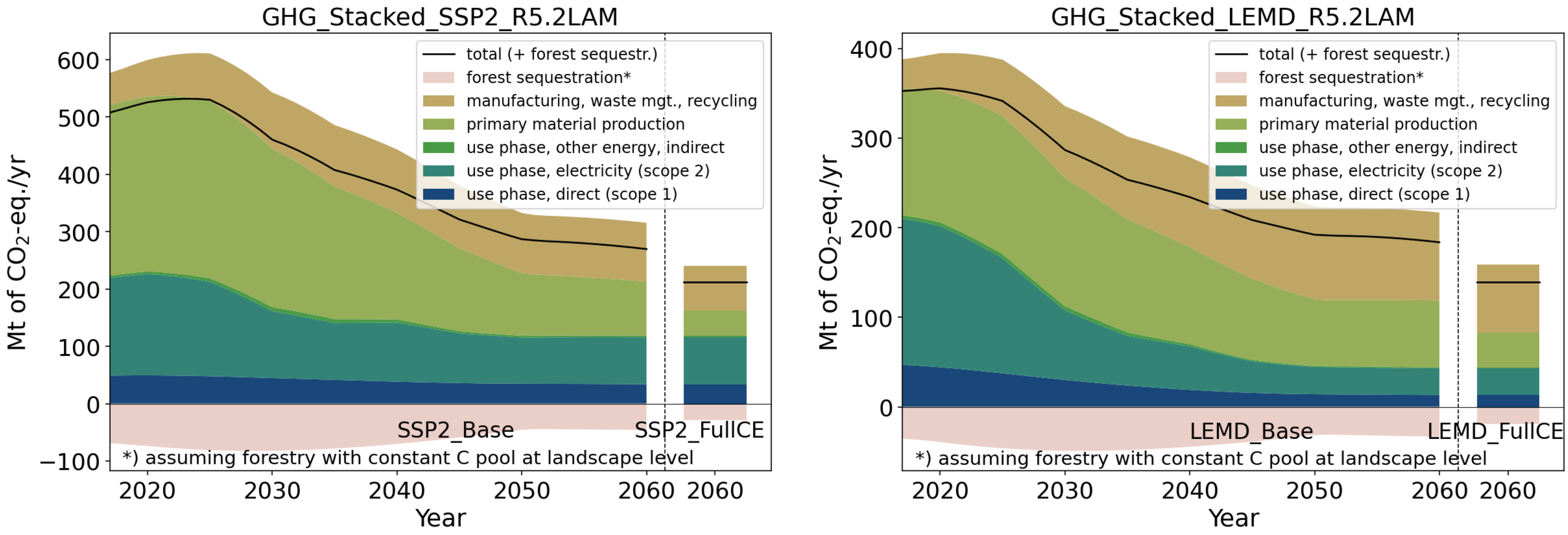

Source: RECCv2.5 model calculation

Results: GHG time series by sector and region (Fig. SP10)

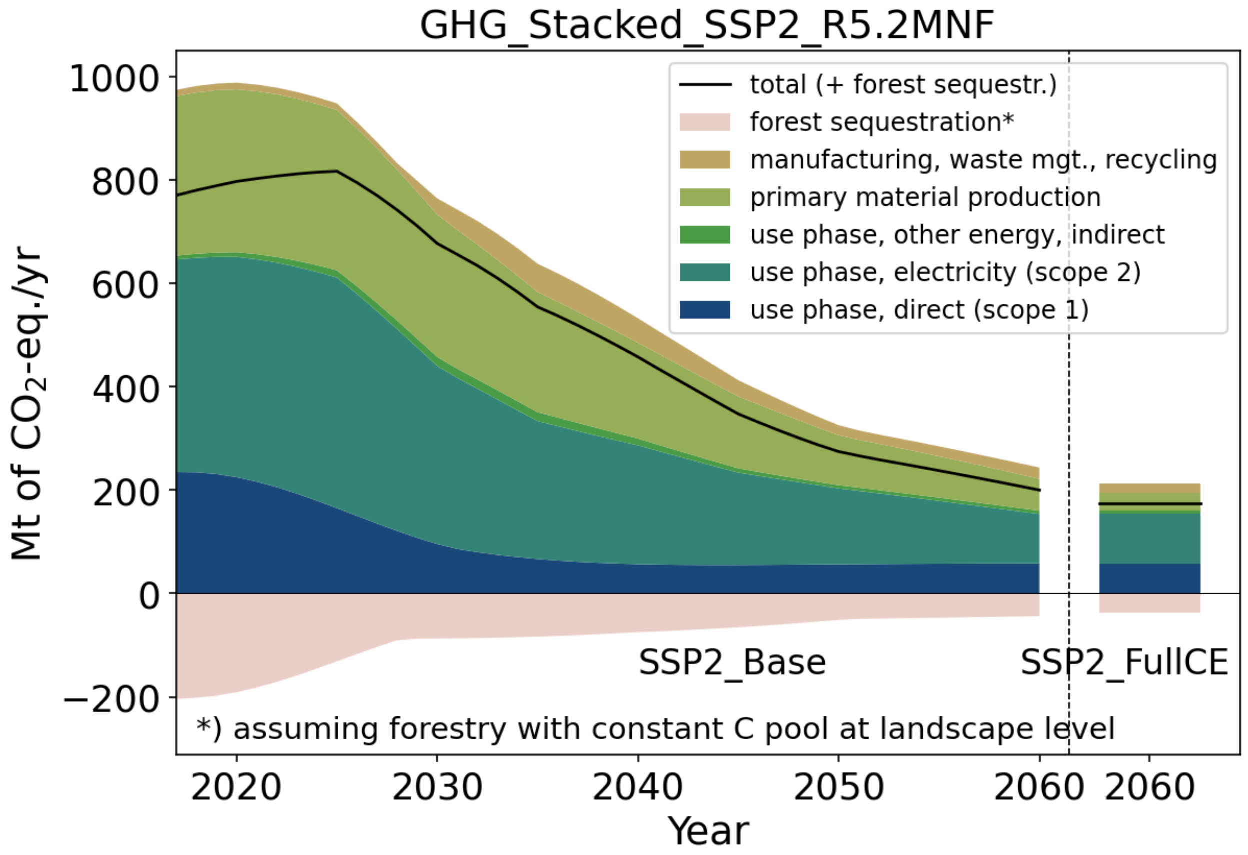

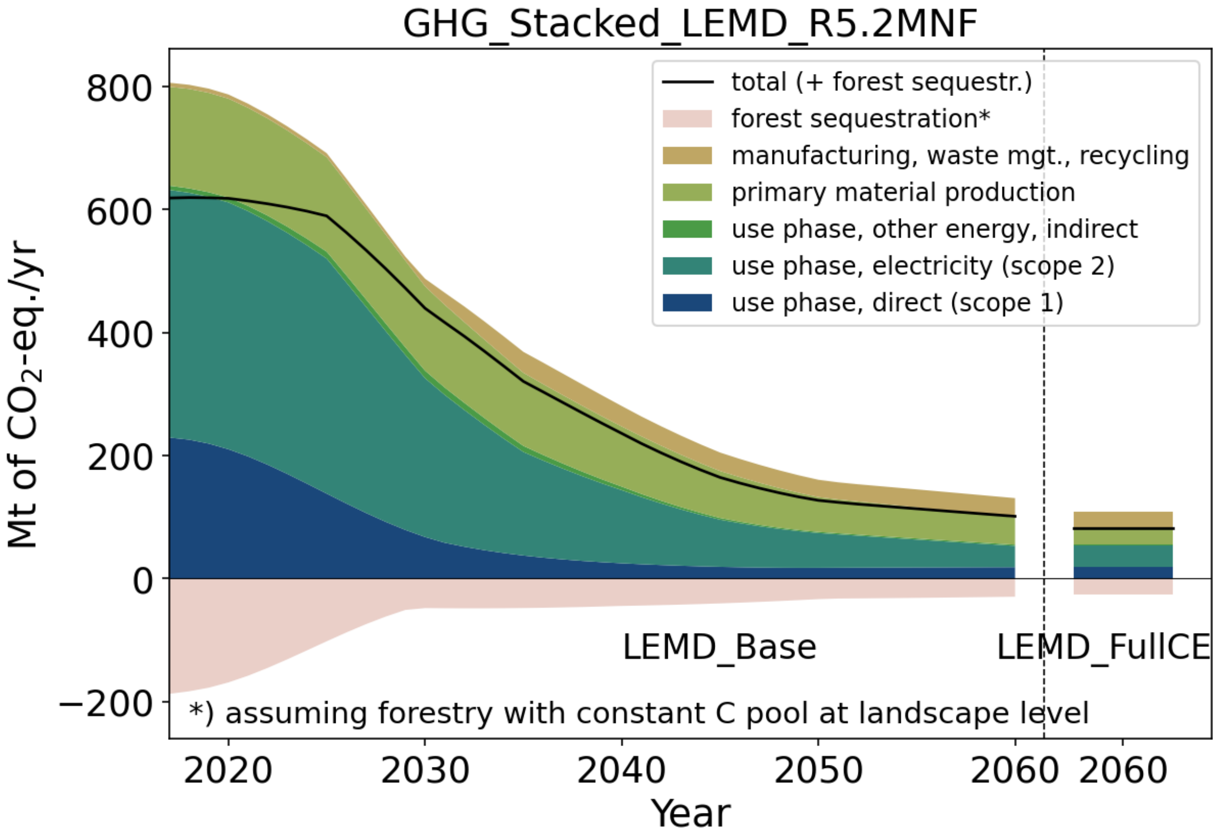

Source: RECCv2.5 model calculation

Results: GHG time series by sector and region (Fig. SP10)

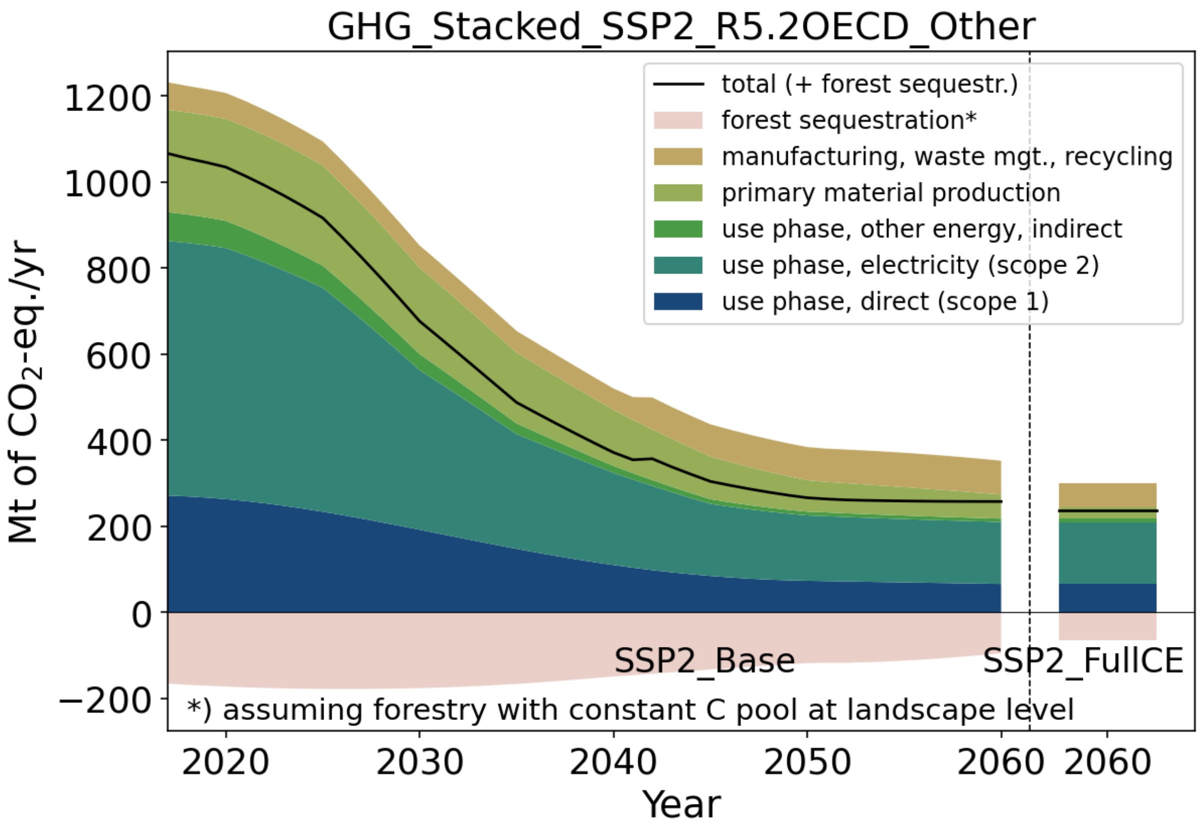

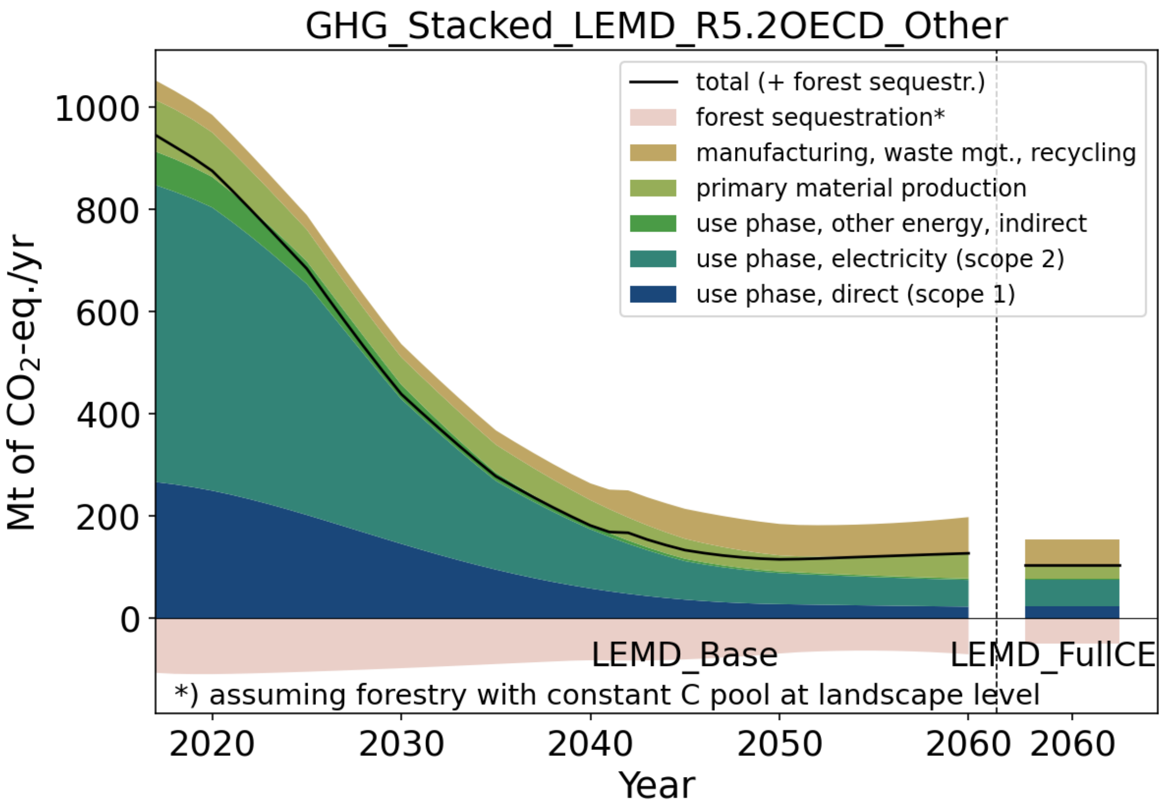

Source: RECCv2.5 model calculation

Results: GHG time series by sector and region (Fig. SP10)

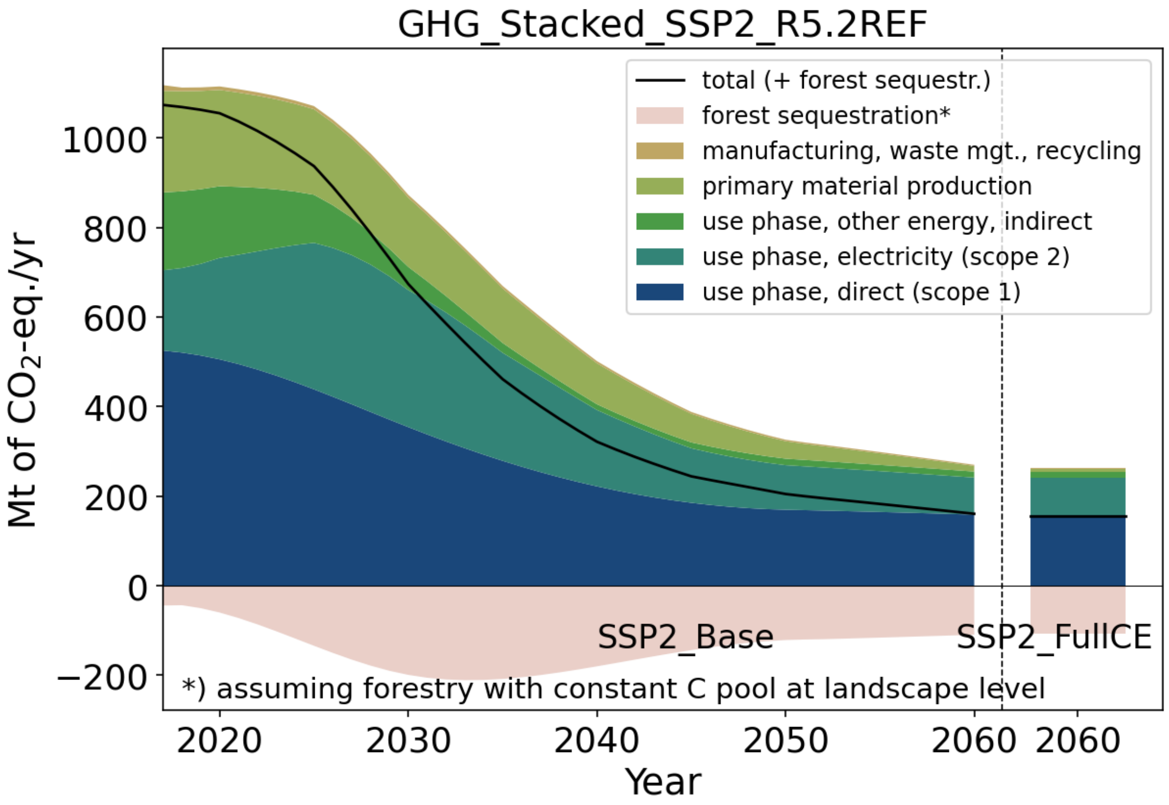

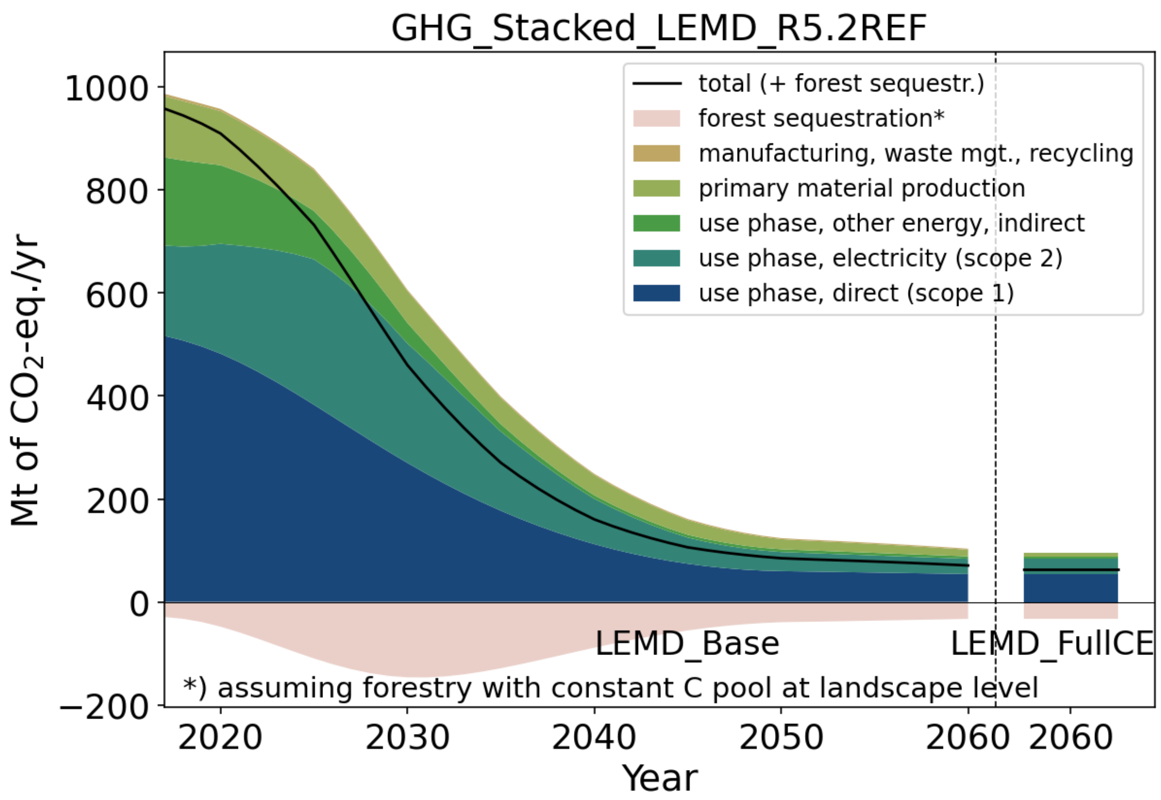

Source: RECCv2.5 model calculation

Results: GHG time series by sector and region (Fig. SP10)

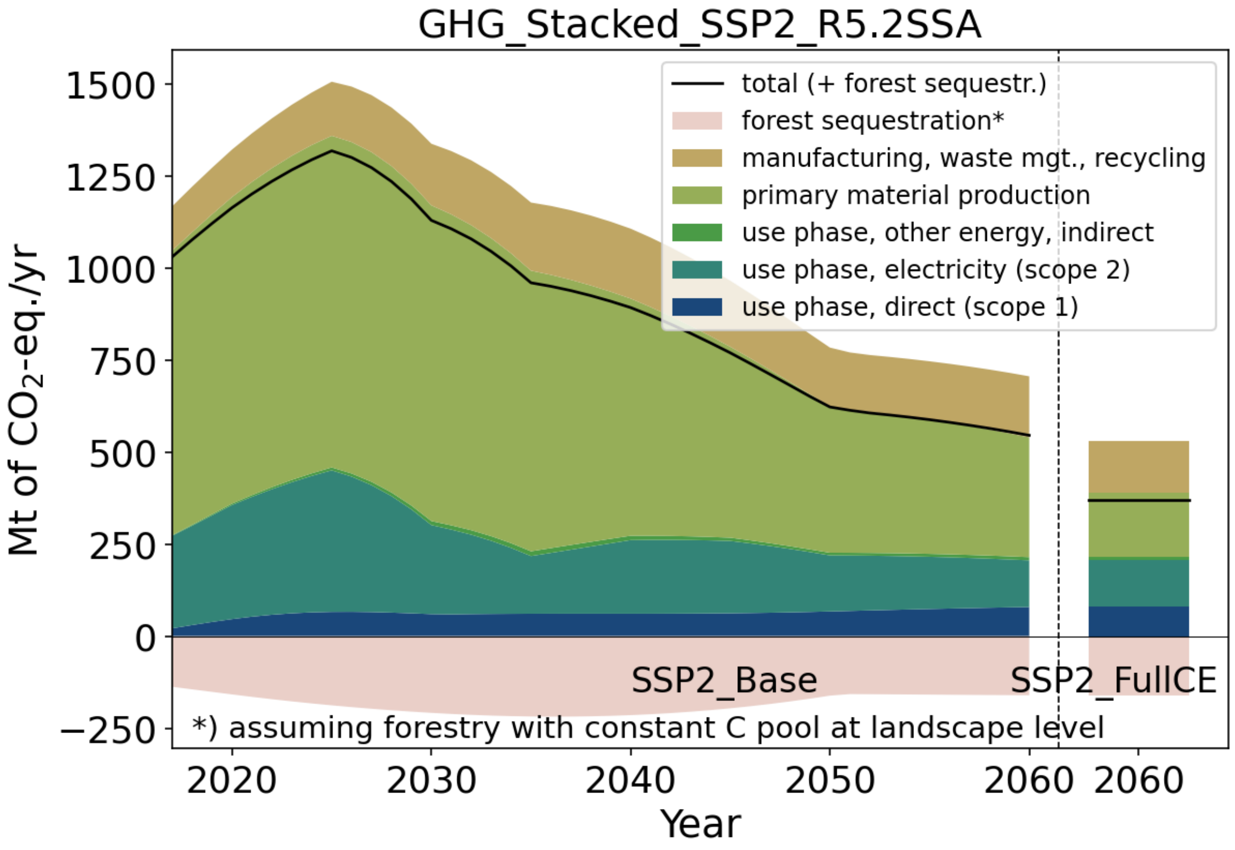

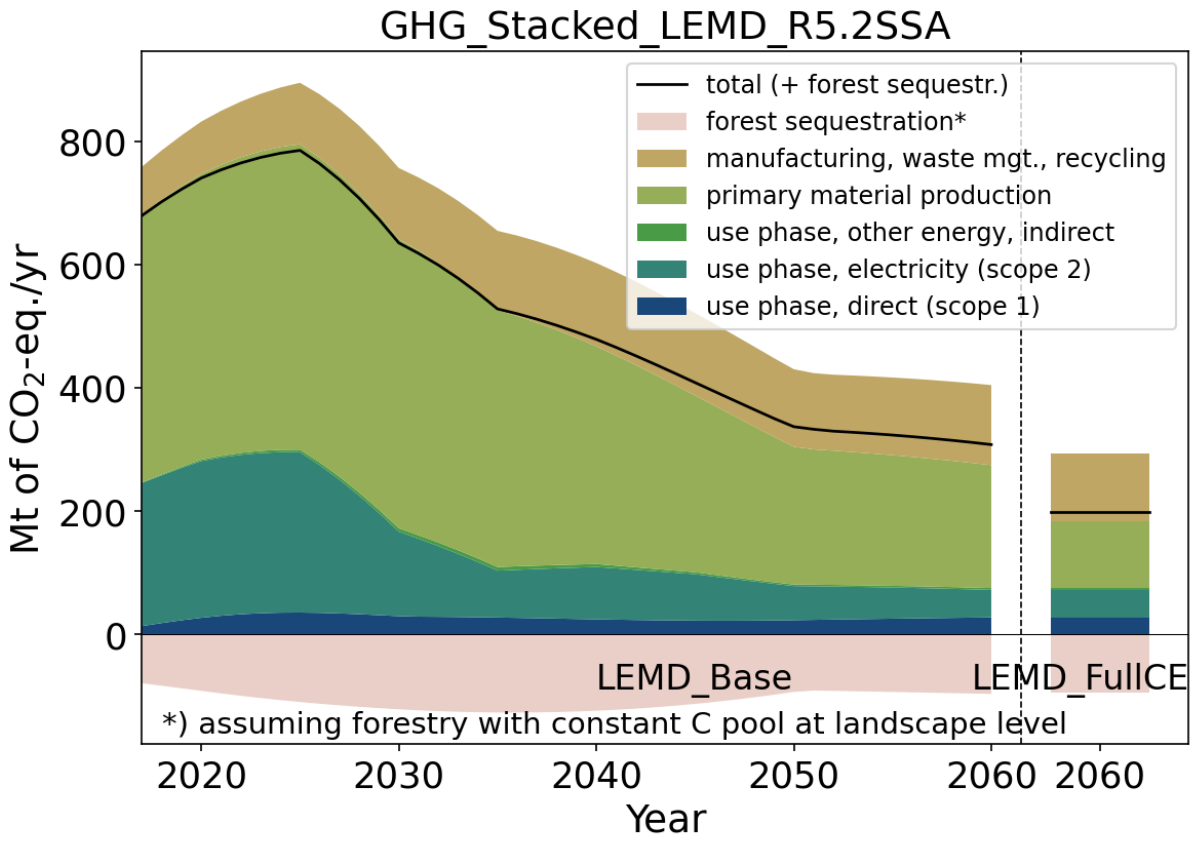

Source: RECCv2.5 model calculation

Results: GHG time series by sector and region (Fig. SP10)

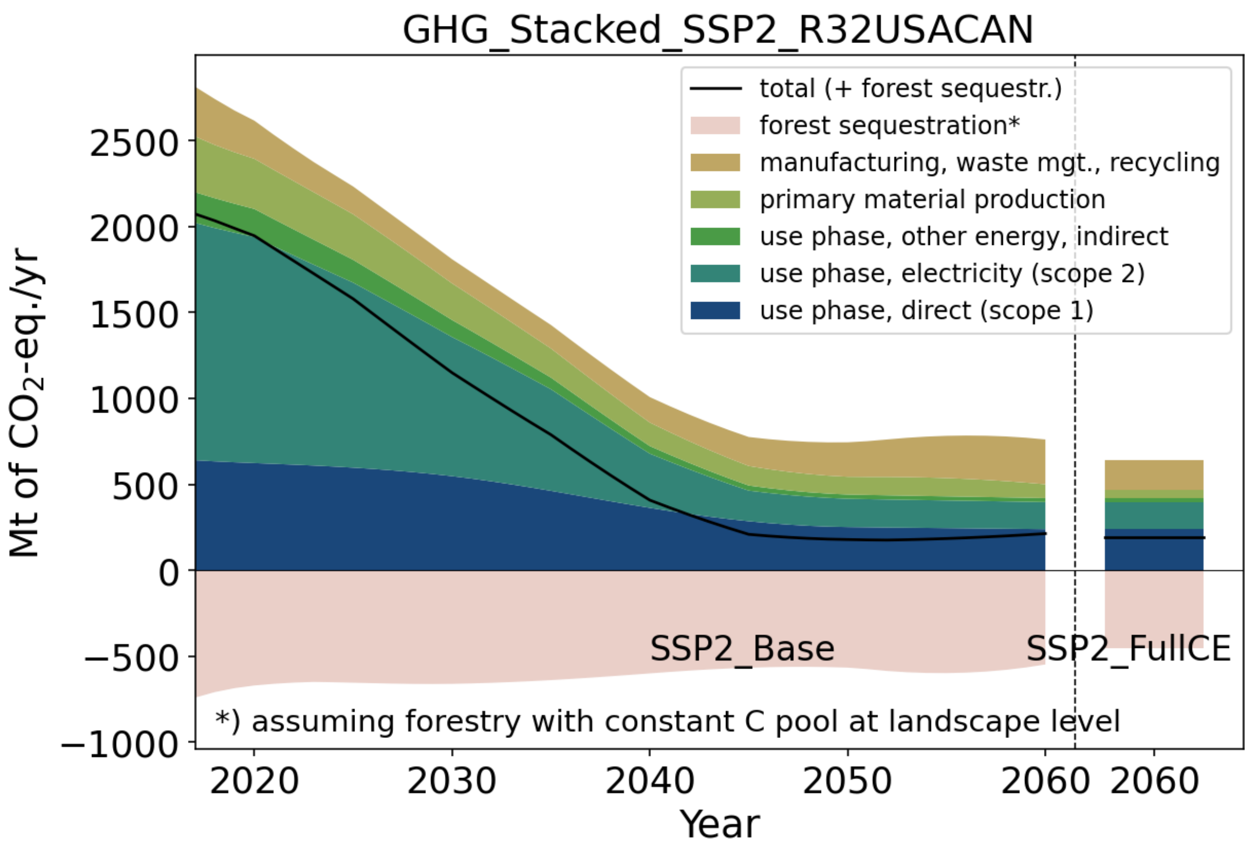

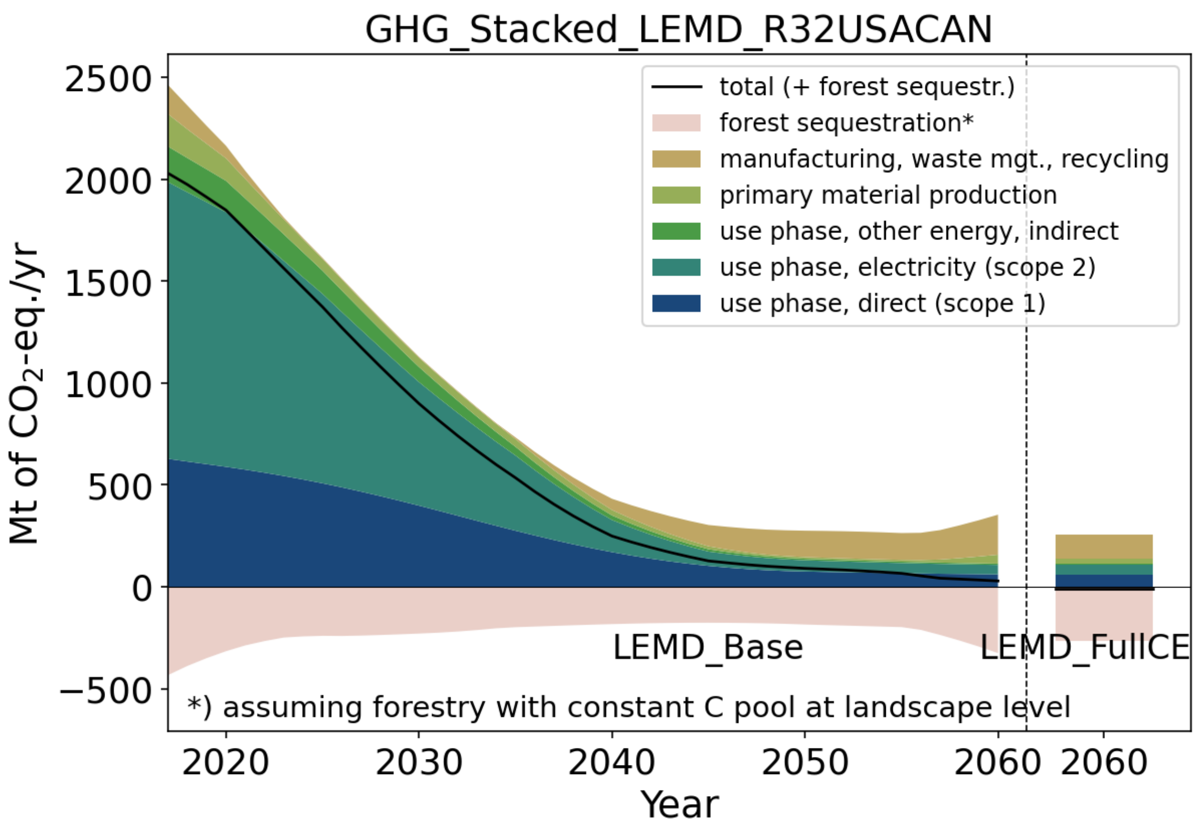

Source: RECCv2.5 model calculation

Results: GHG time series by sector and region (Fig. SP10)

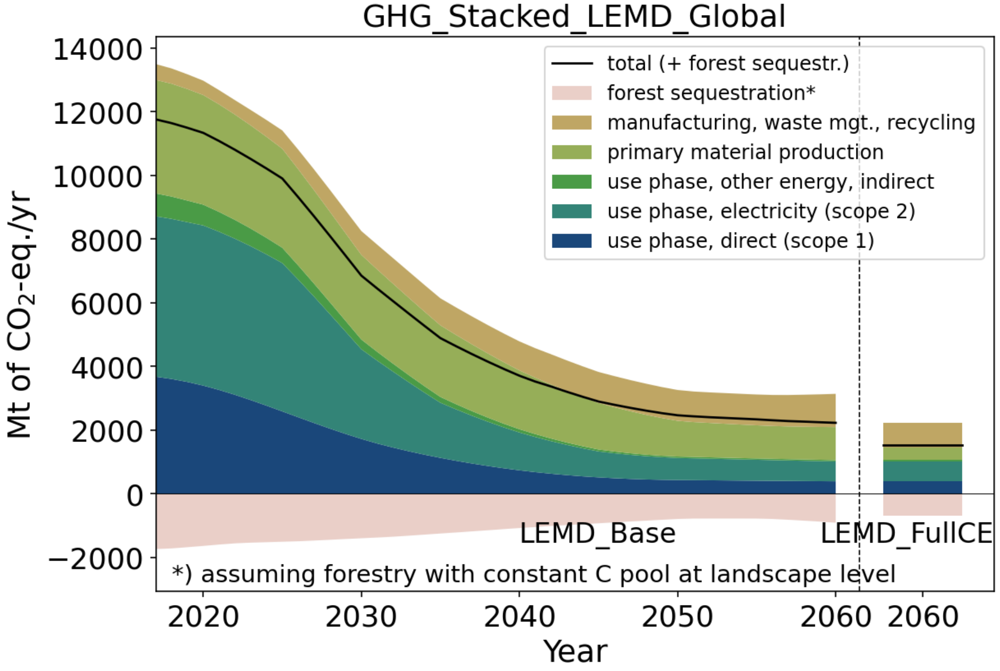

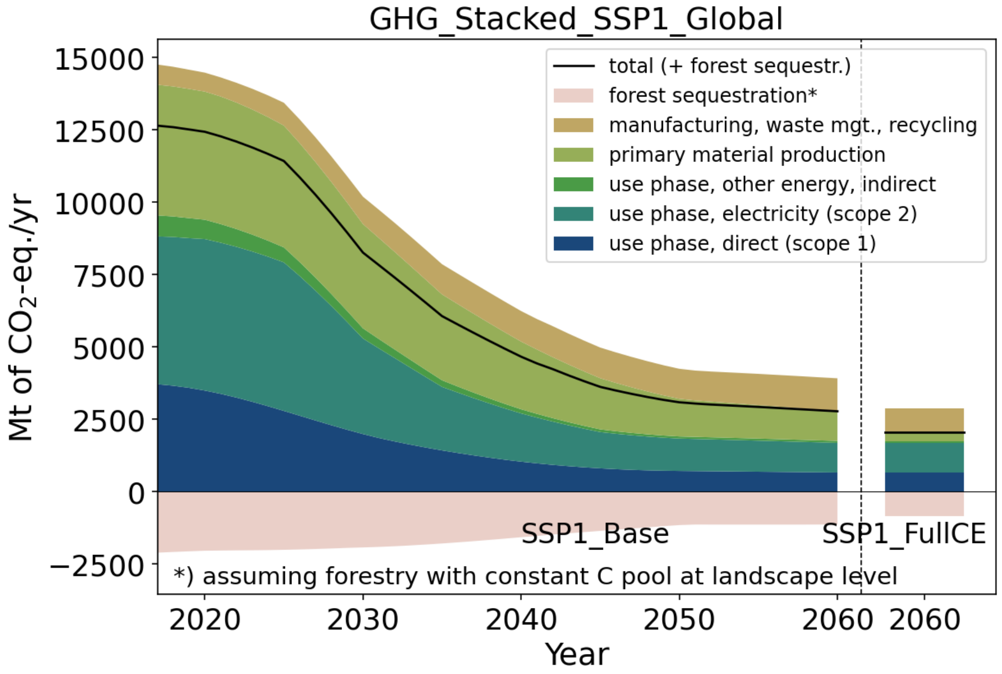

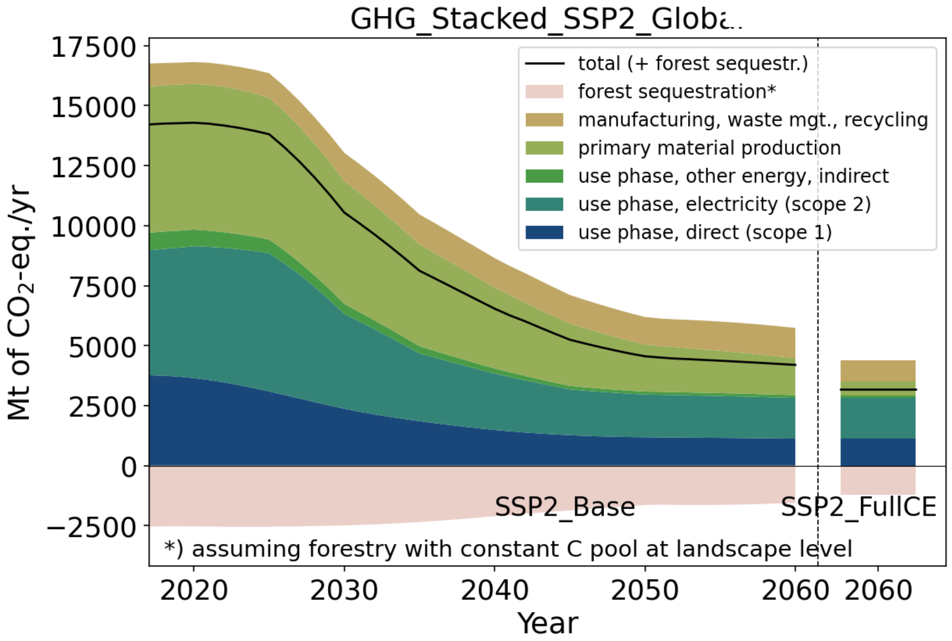

Source: RECCv2.5 model calculation

Results: GHG time series by scenario and region (Fig. SP11)

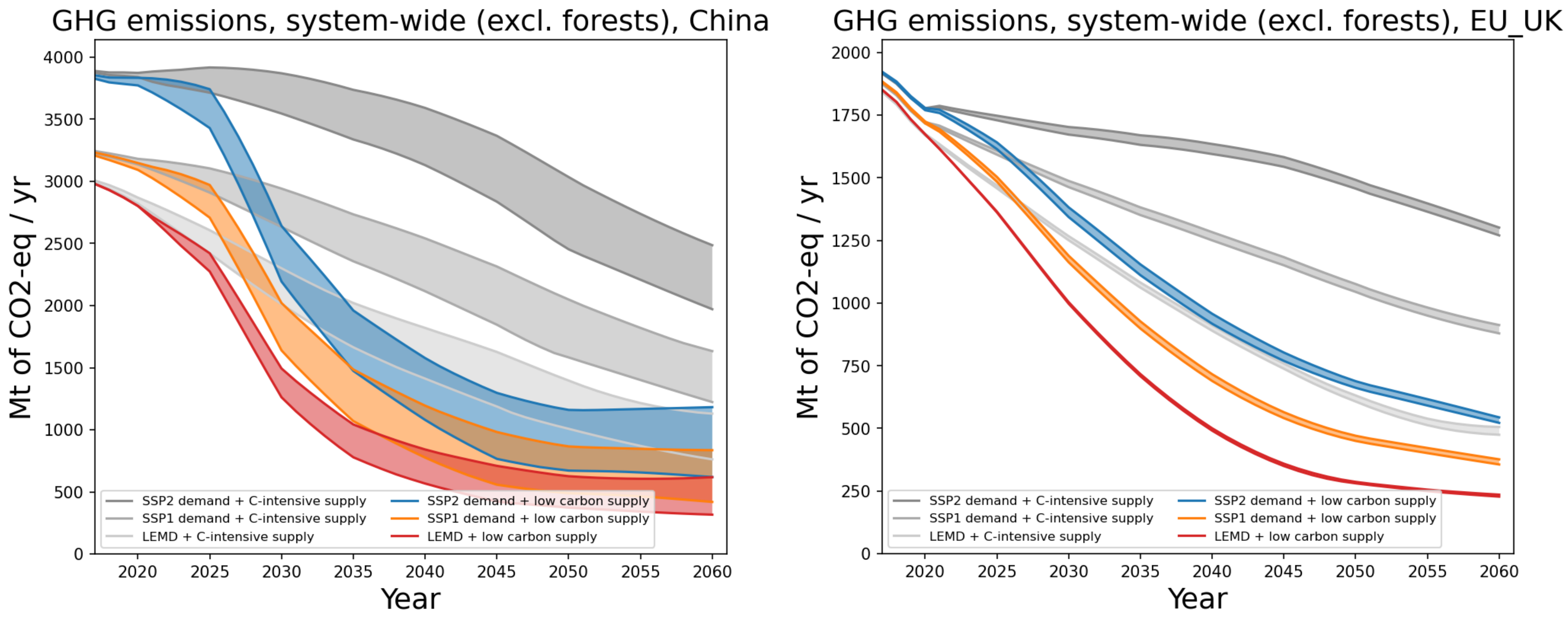

Source: RECCv2.5 model calculation

Results: GHG time series by scenario and region (Fig. SP11)

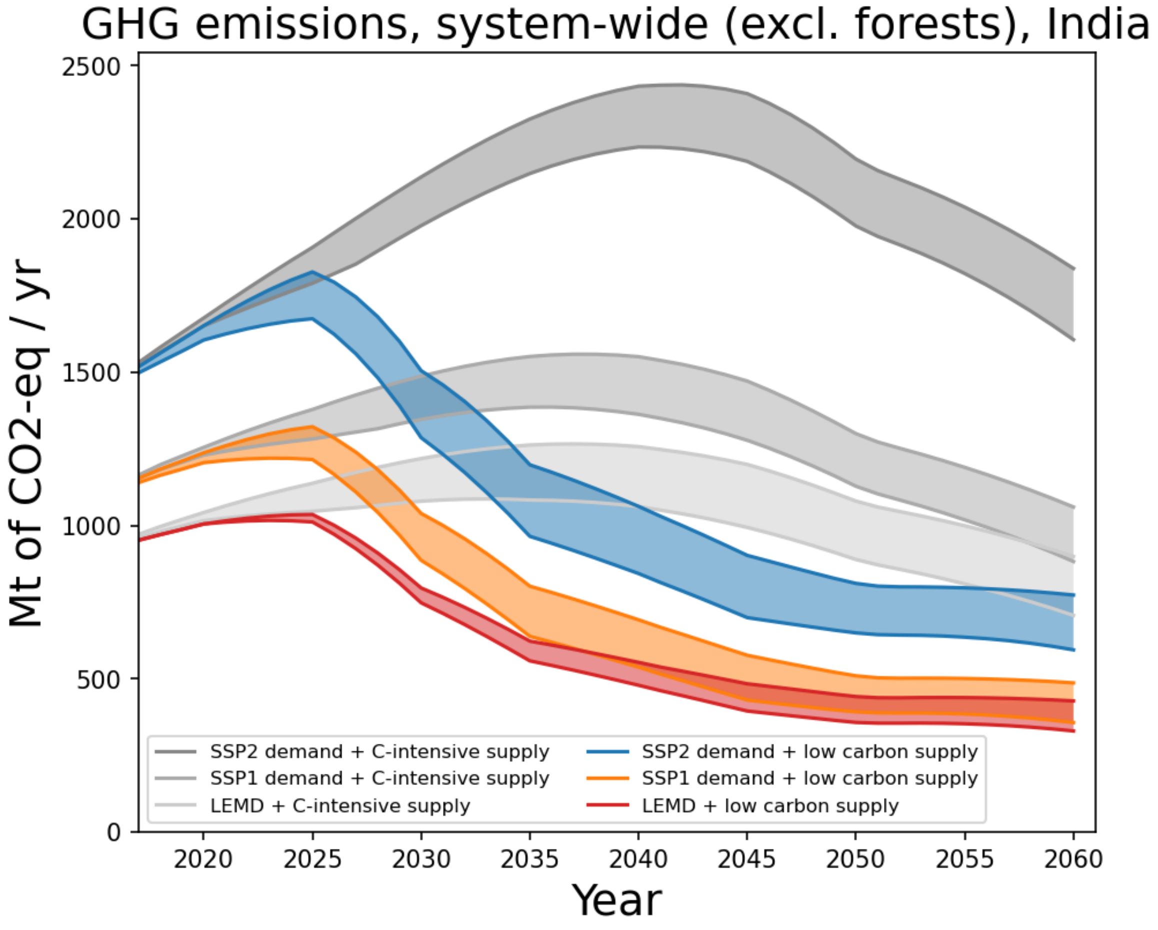

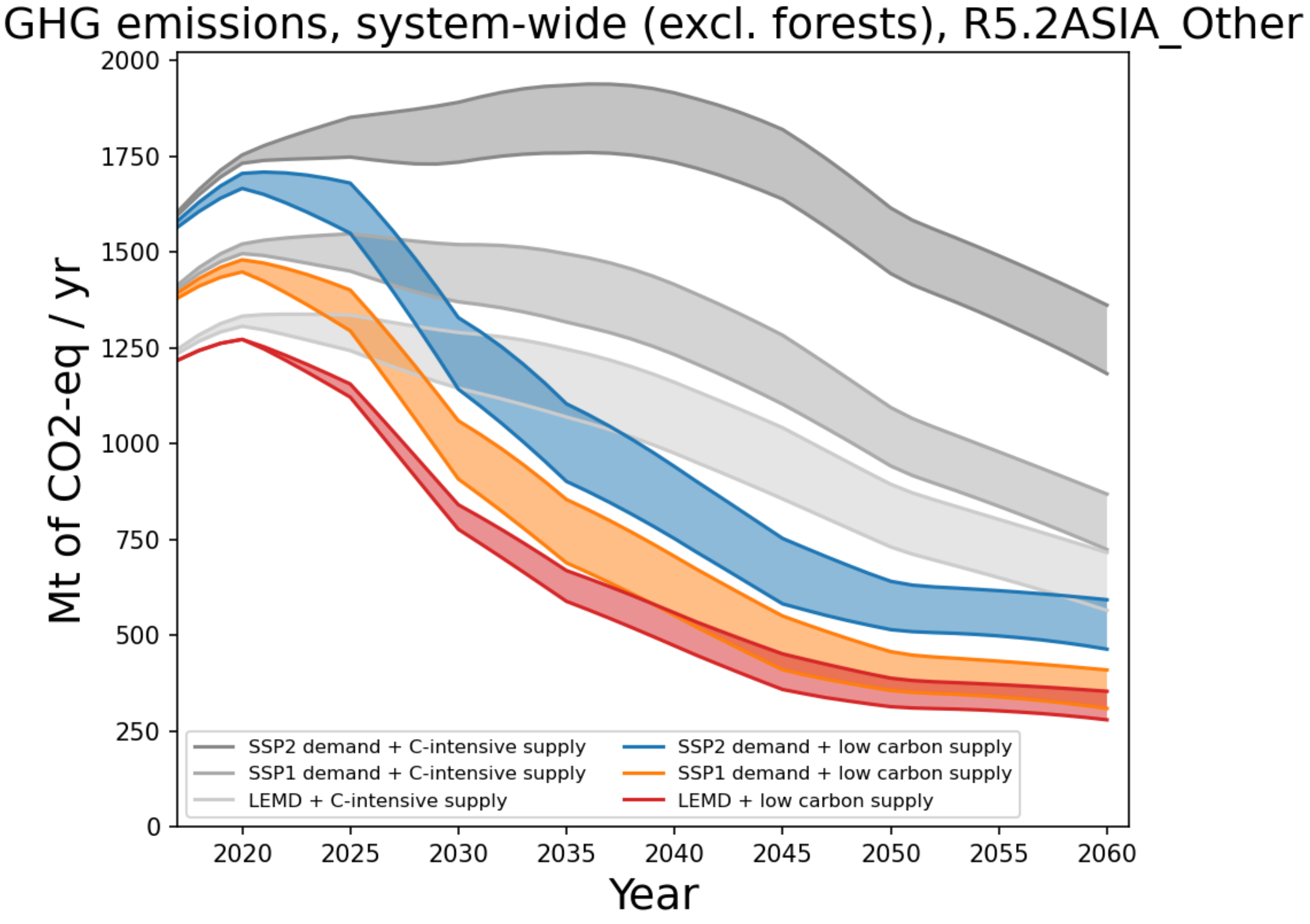

Source: RECCv2.5 model calculation

Results: GHG time series by scenario and region (Fig. SP11)

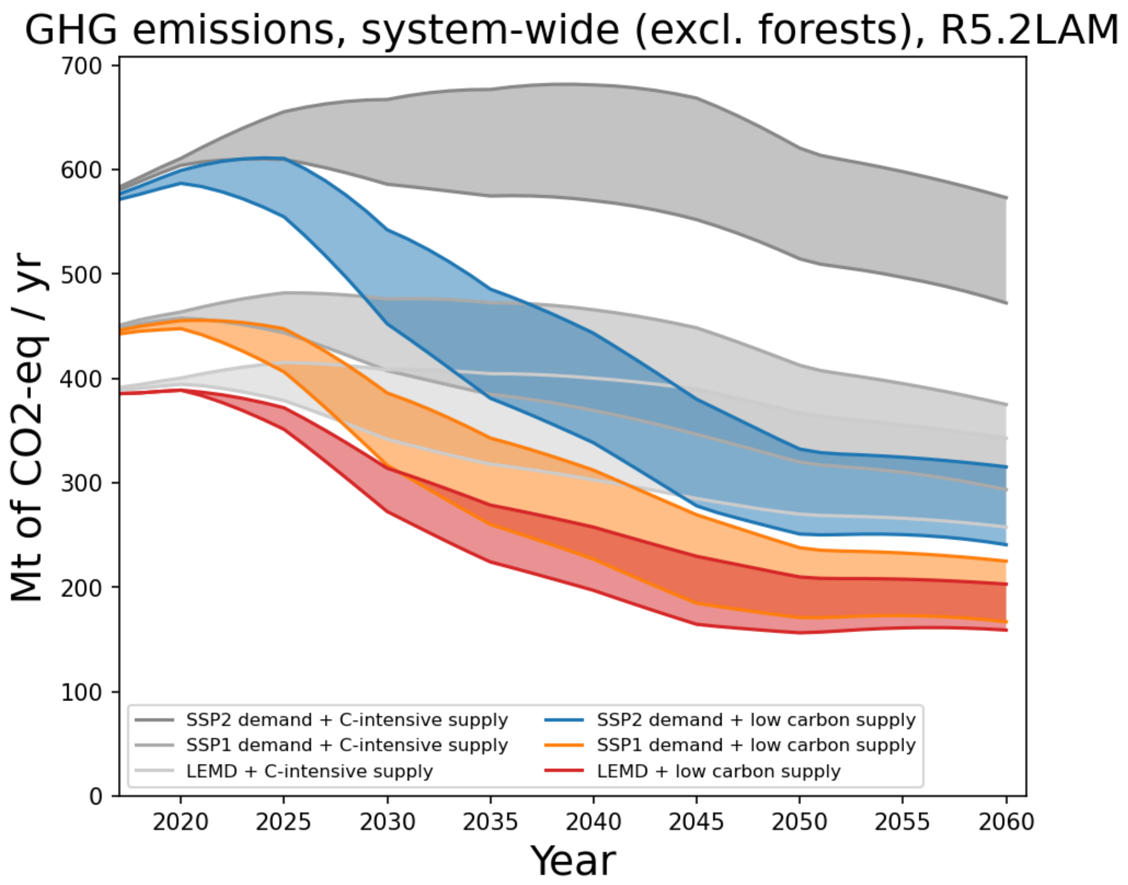

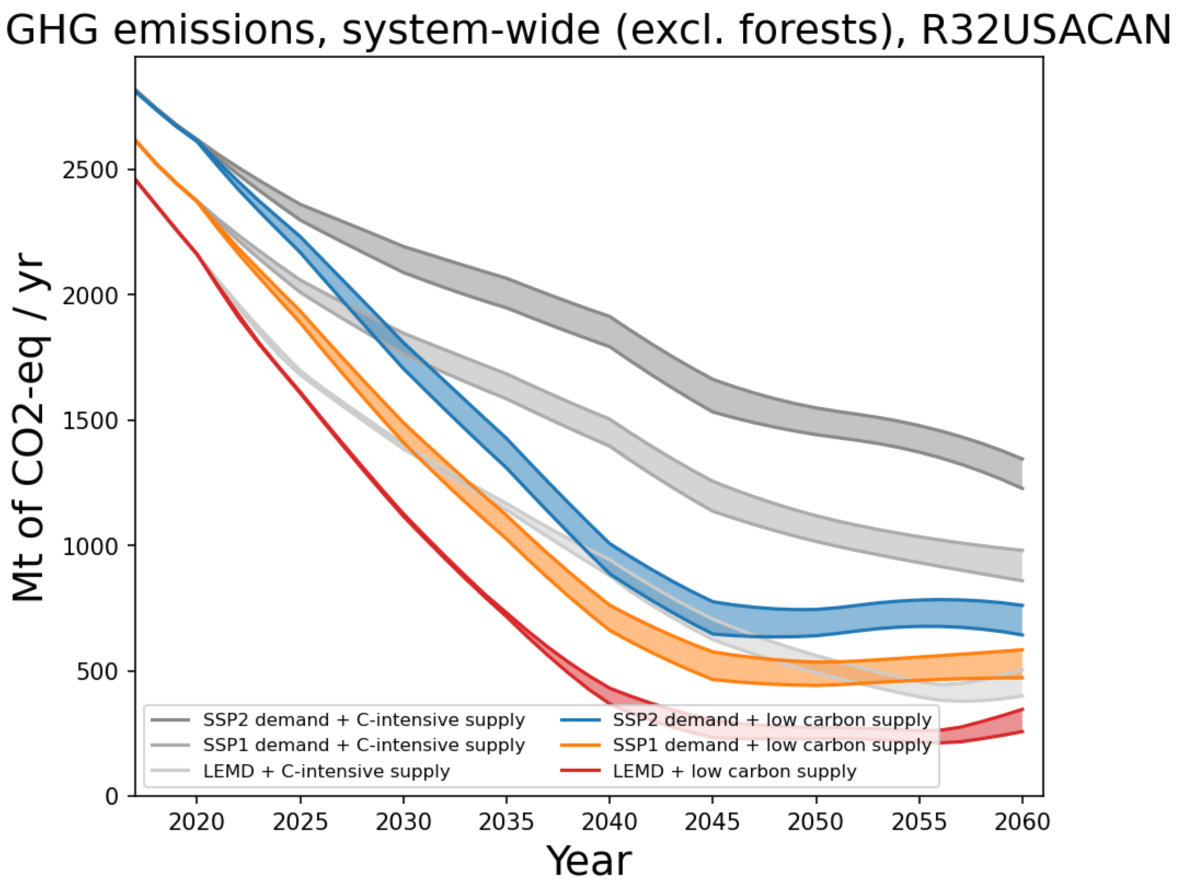

Source: RECCv2.5 model calculation

Results: GHG time series by scenario and region (Fig. SP11)

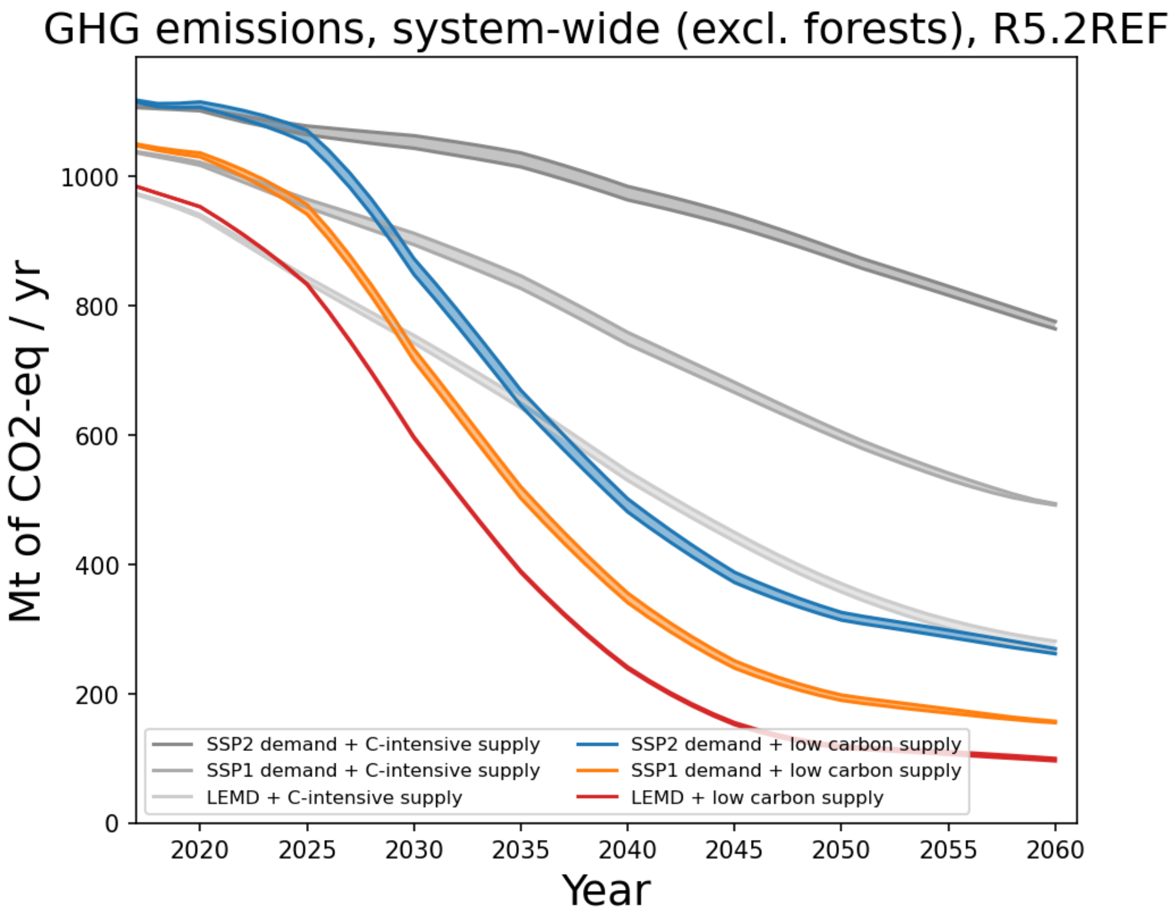

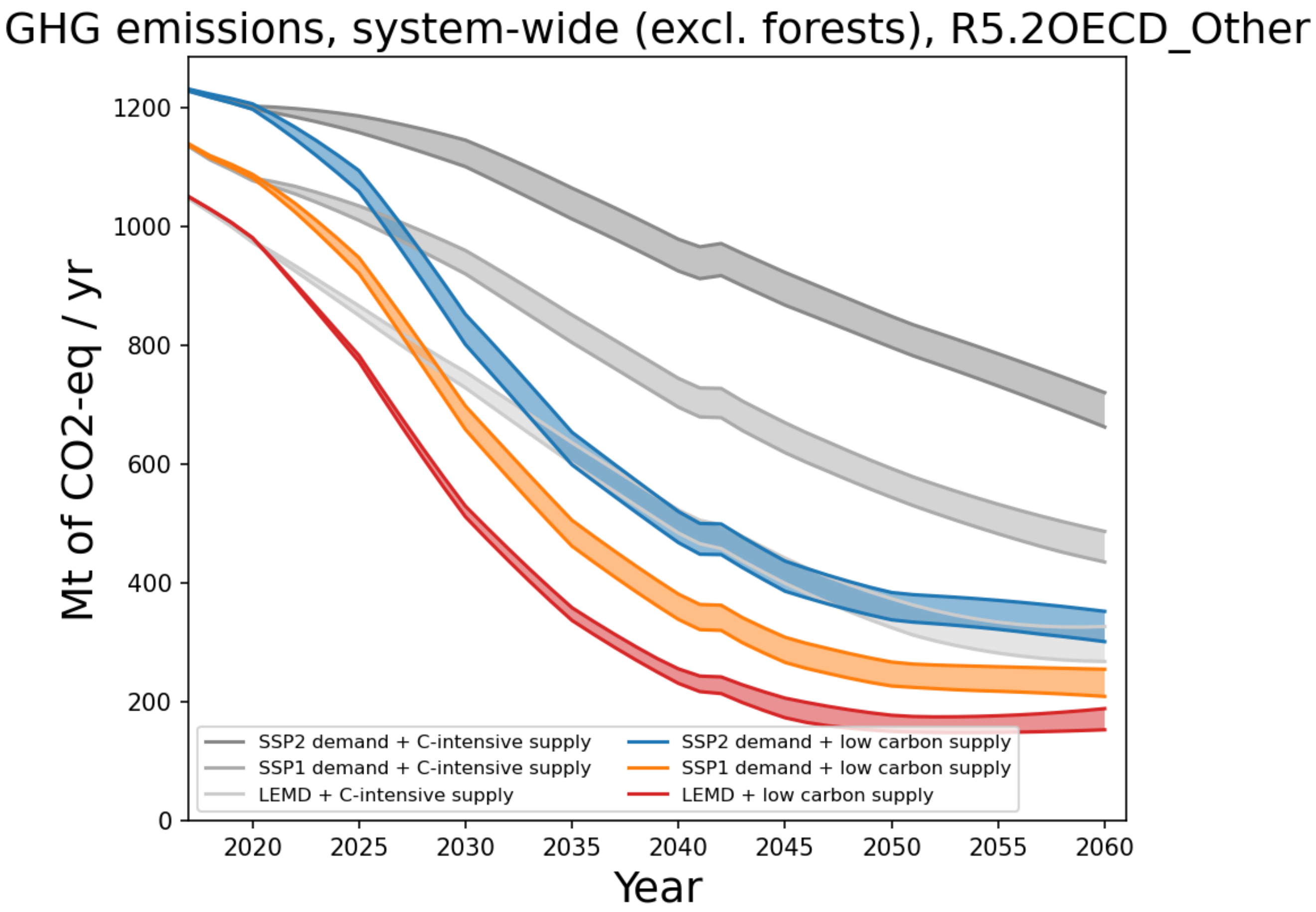

Source: RECCv2.5 model calculation

Results: GHG time series by scenario and region (Fig. SP11)

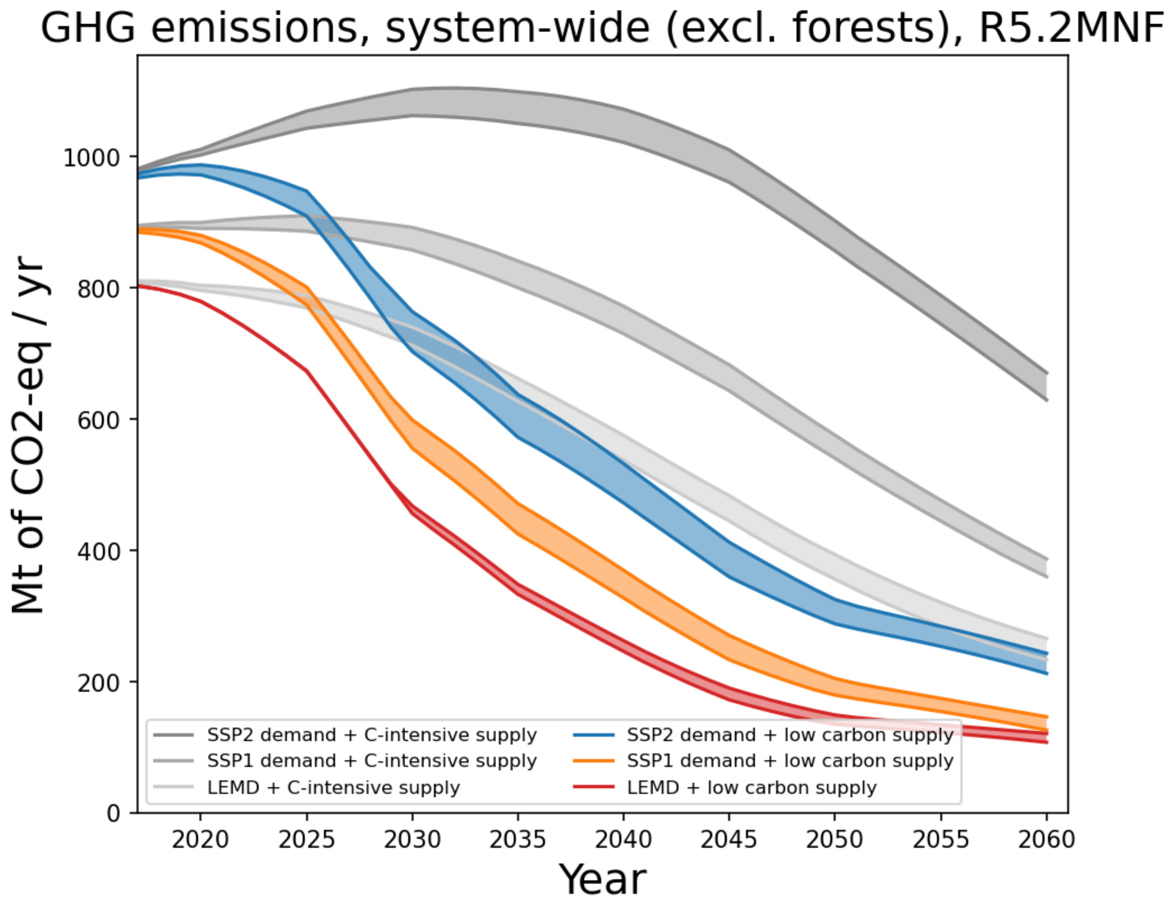

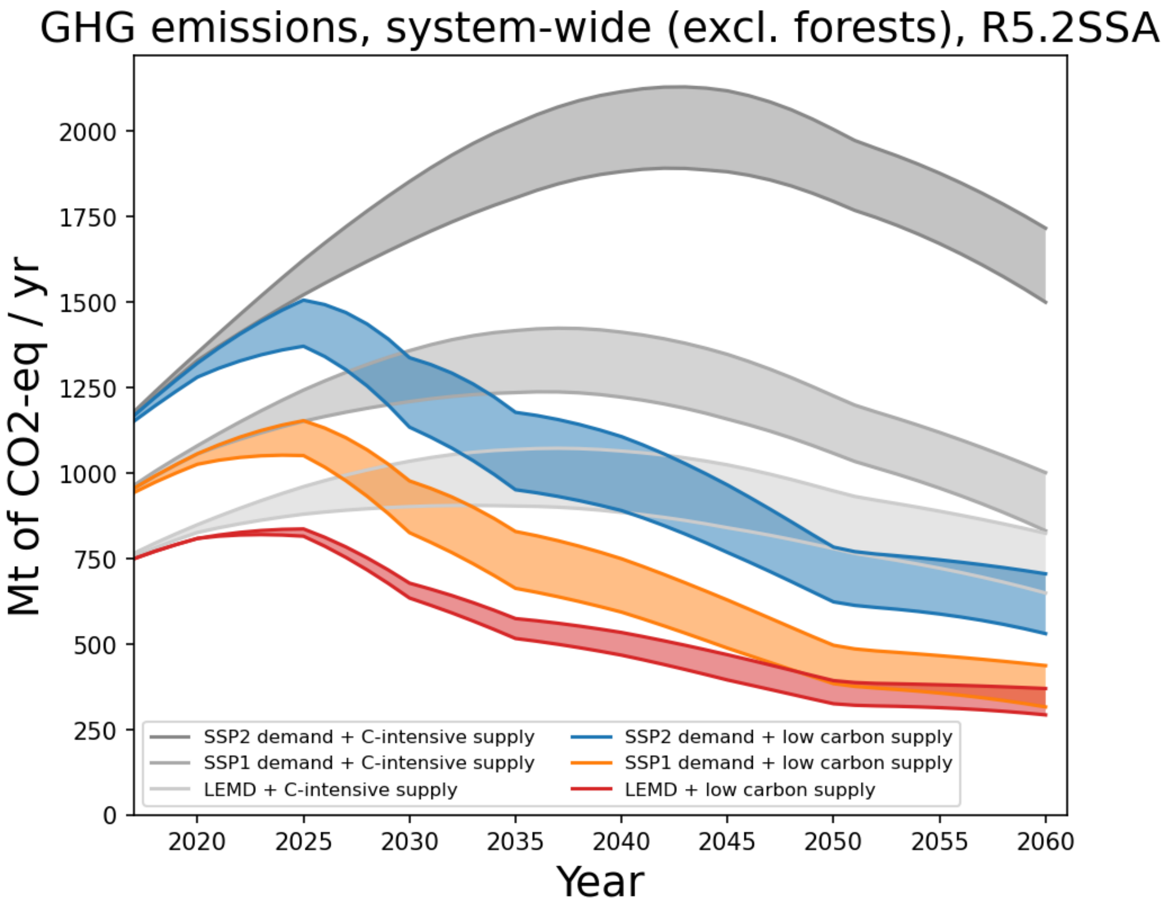

Source: RECCv2.5 model calculation

Results: GHG time series by scenario and region (Fig. SP11)

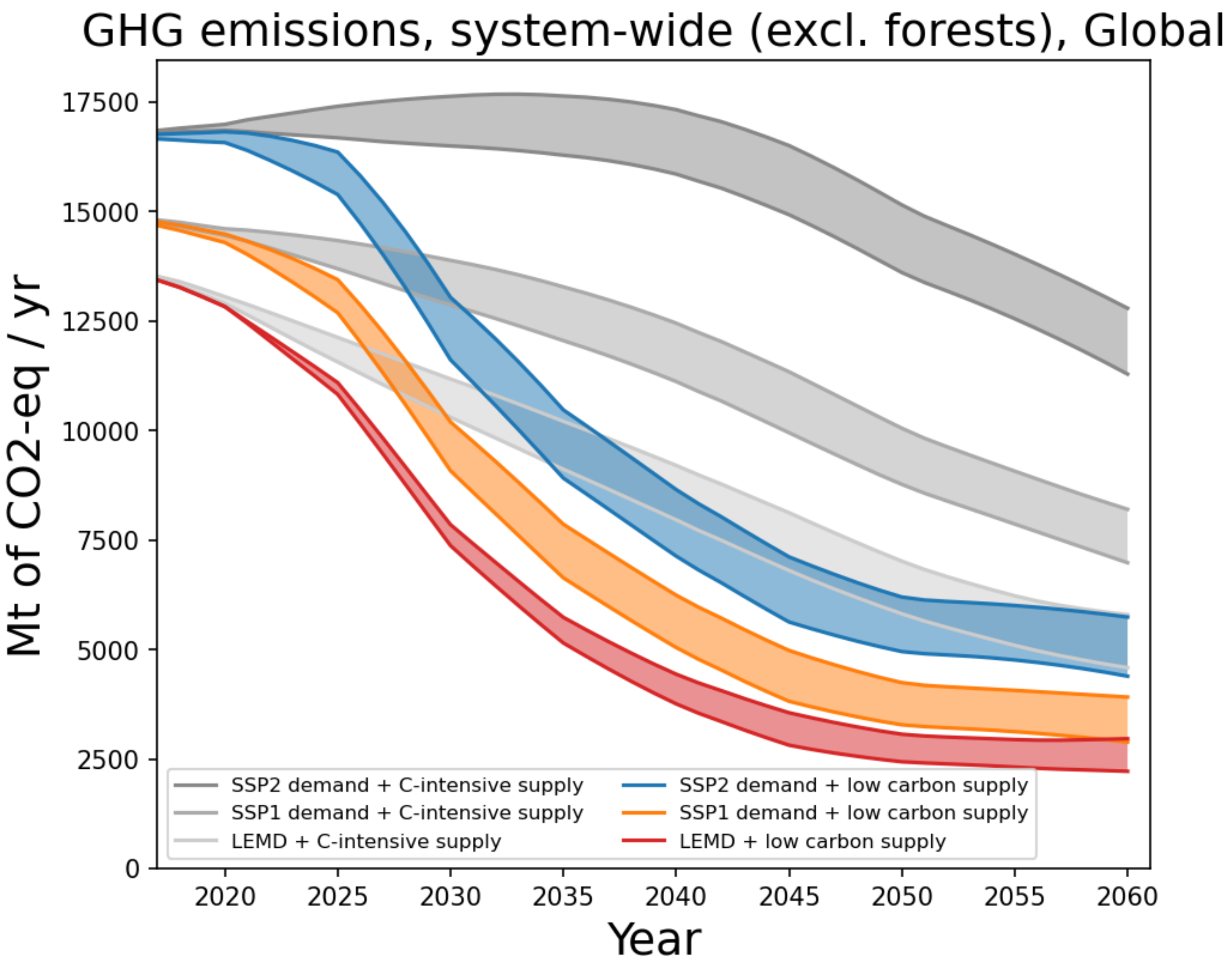

Source: RECCv2.5 model calculation

Results: 2050 GHG by CE strategy and region (Fig. SP12)

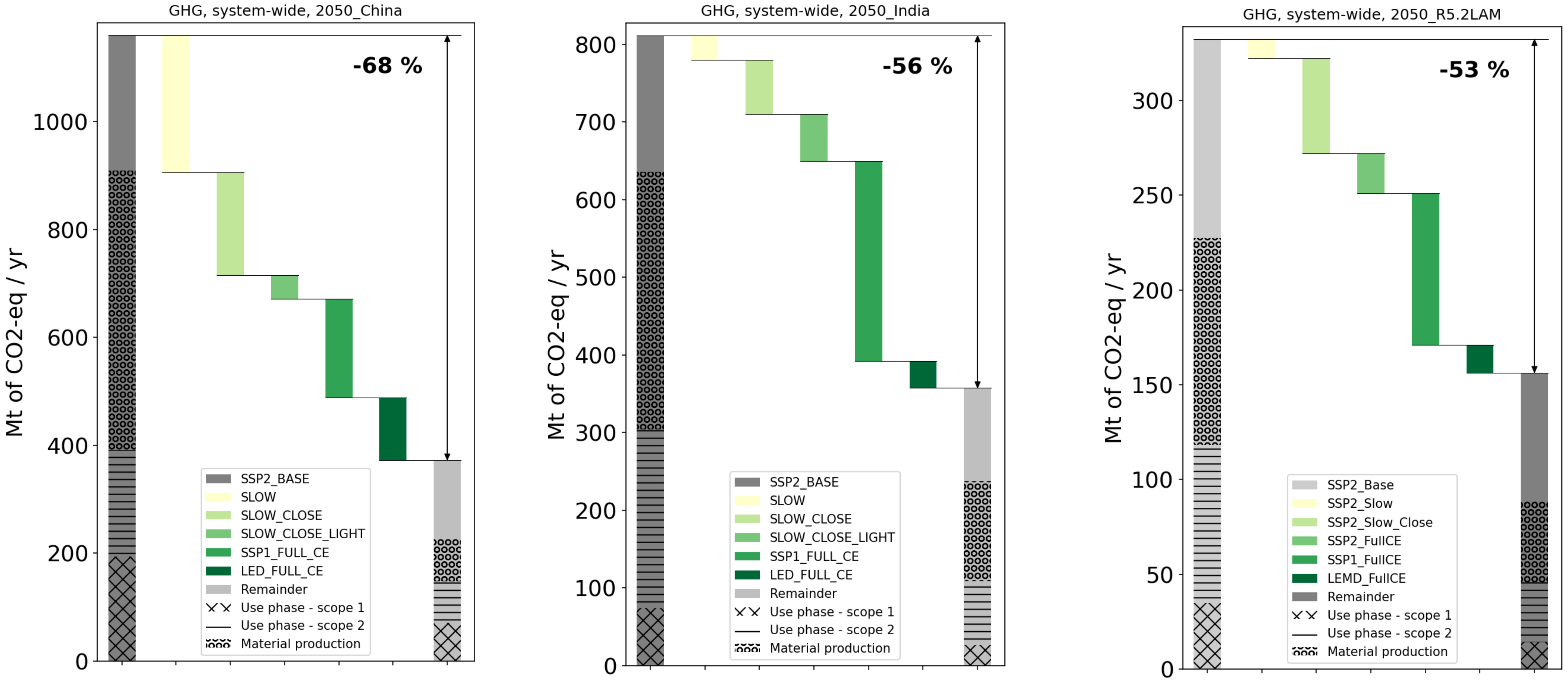

Source: RECCv2.5 model calculation

Results: 2050 GHG by CE strategy and region (Fig. SP12)

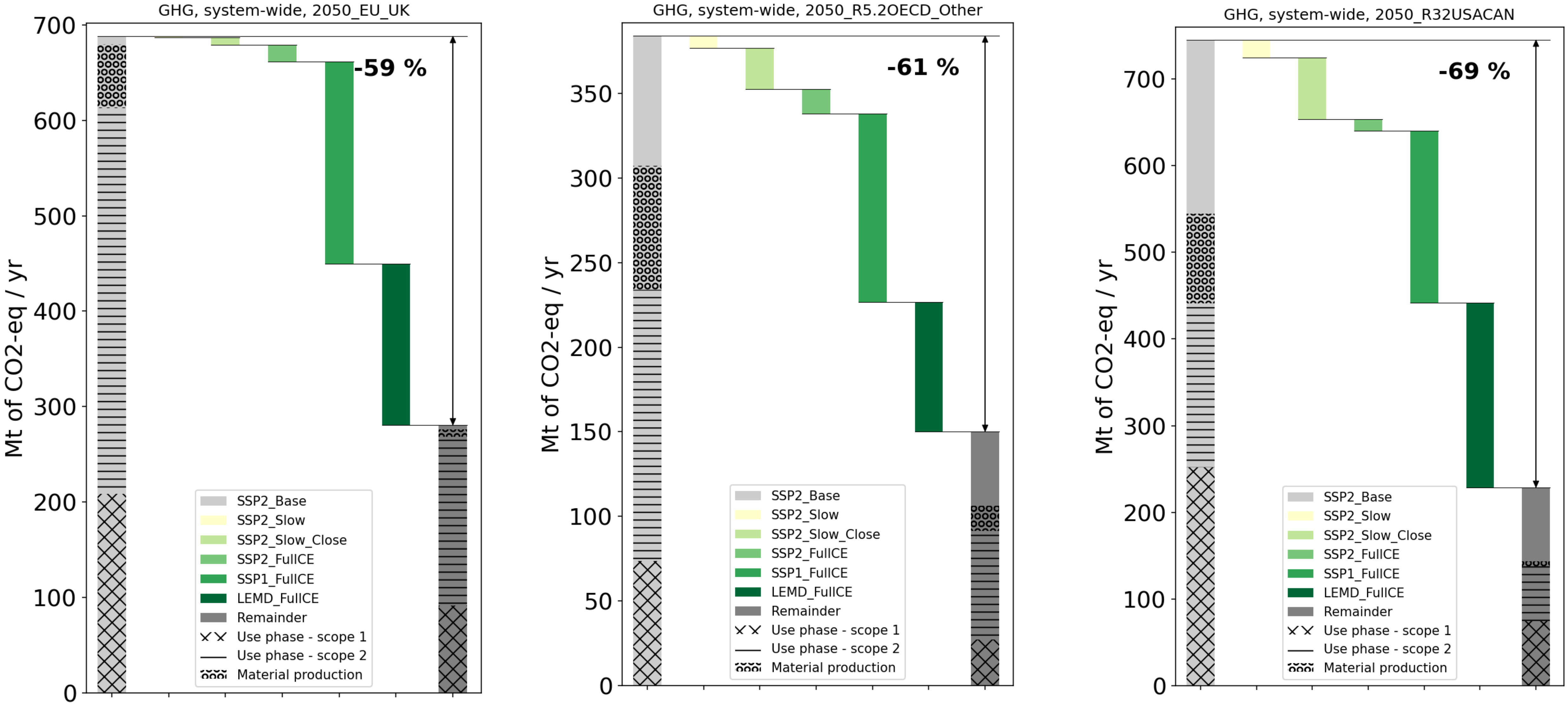

Source: RECCv2.5 model calculation

Results: 2050 GHG by CE strategy and region (Fig. SP12)

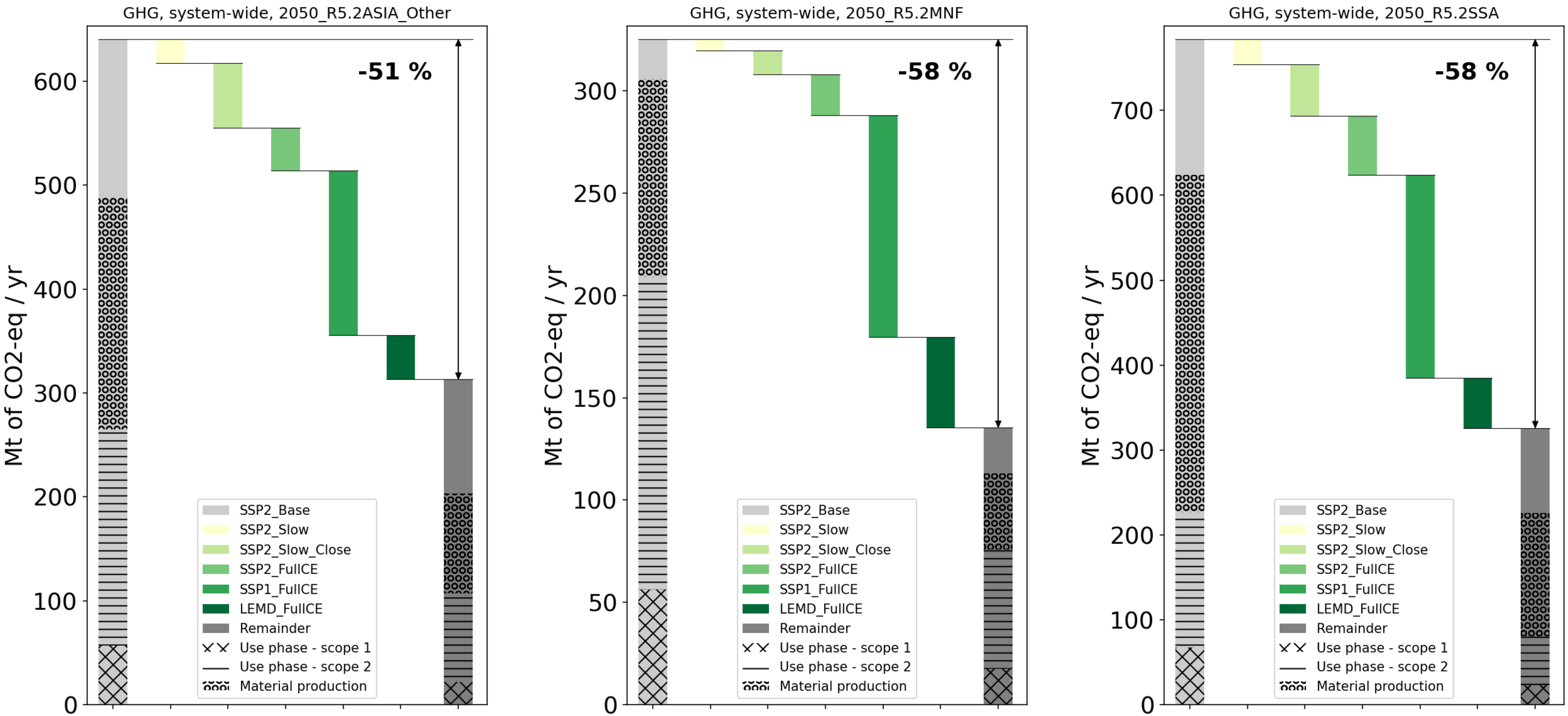

Source: RECCv2.5 model calculation

Results: 2050 GHG by CE strategy and region (Fig. SP12)

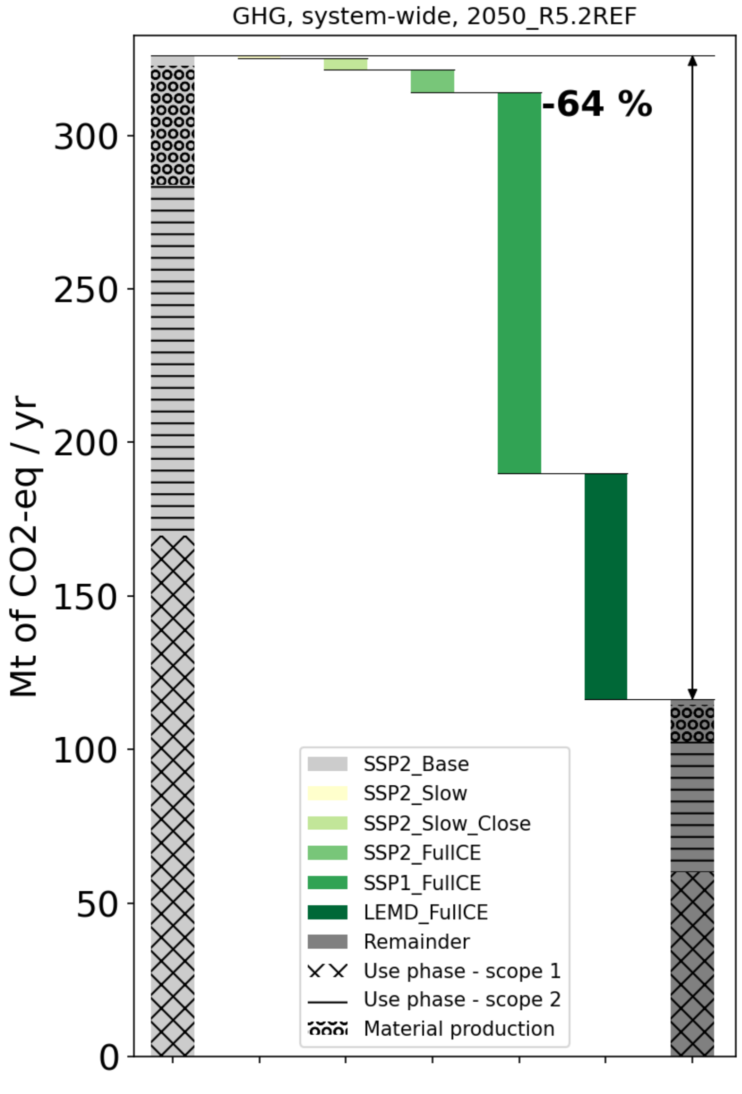

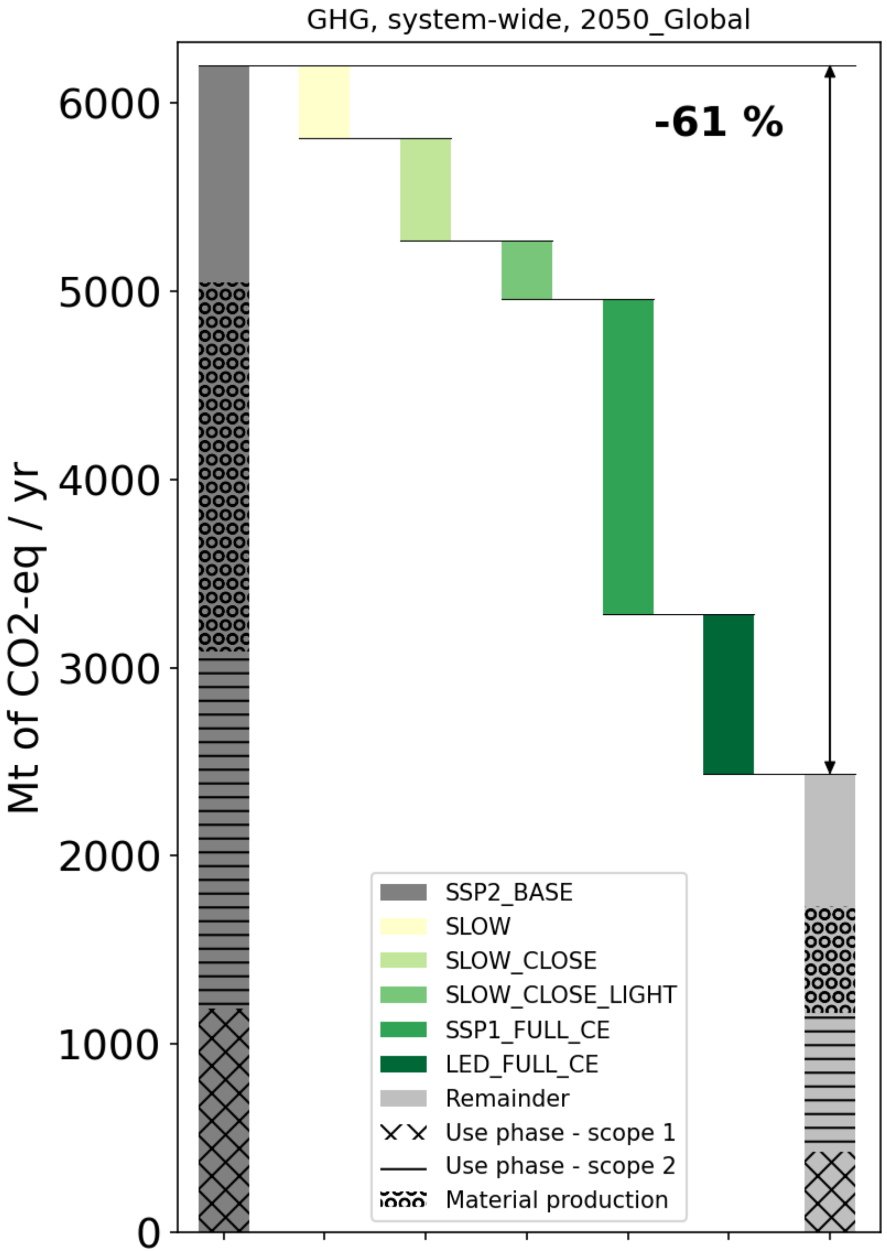

Source: RECCv2.5 model calculation

Results: 2020-2050 Cumulative GHG by CE strategy and region (Fig. SP13)

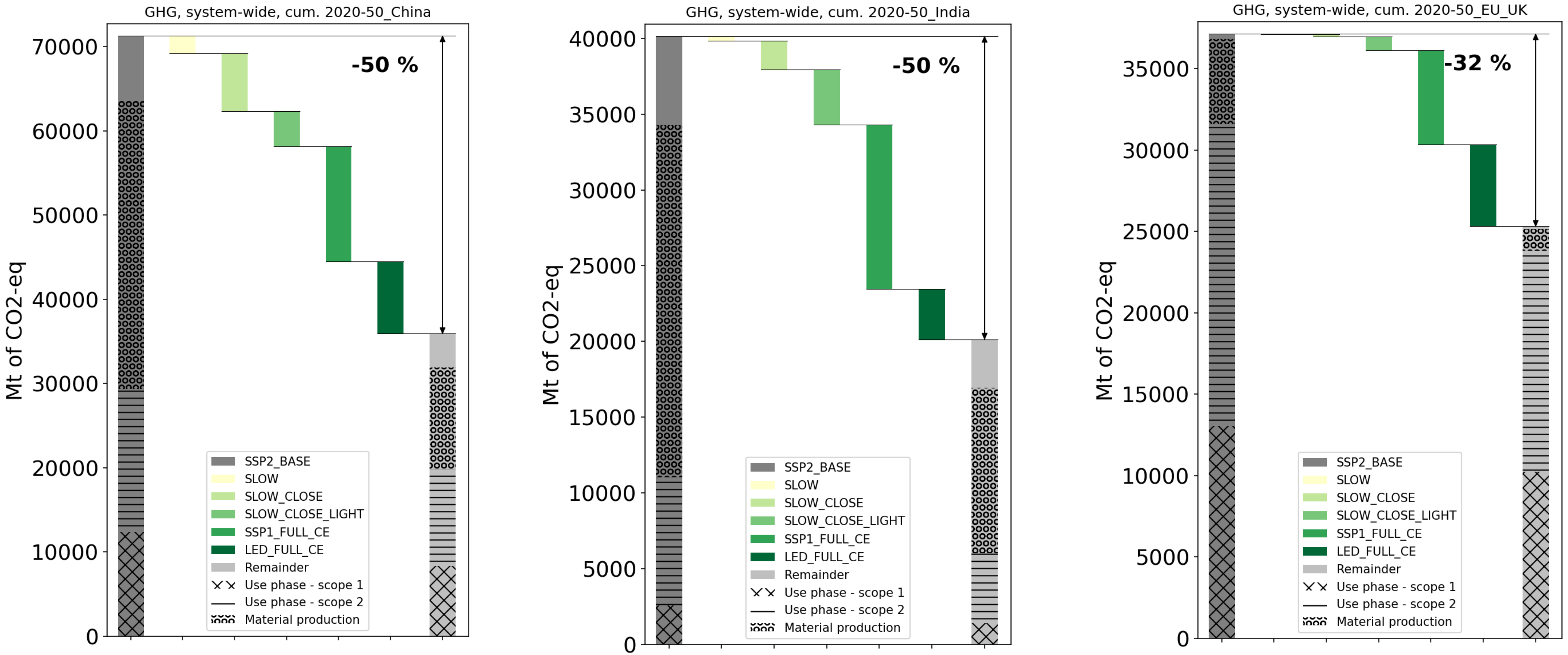

Source: RECCv2.5 model calculation

Results: 2020-2050 Cumulative GHG by CE strategy and region (Fig. SP13)

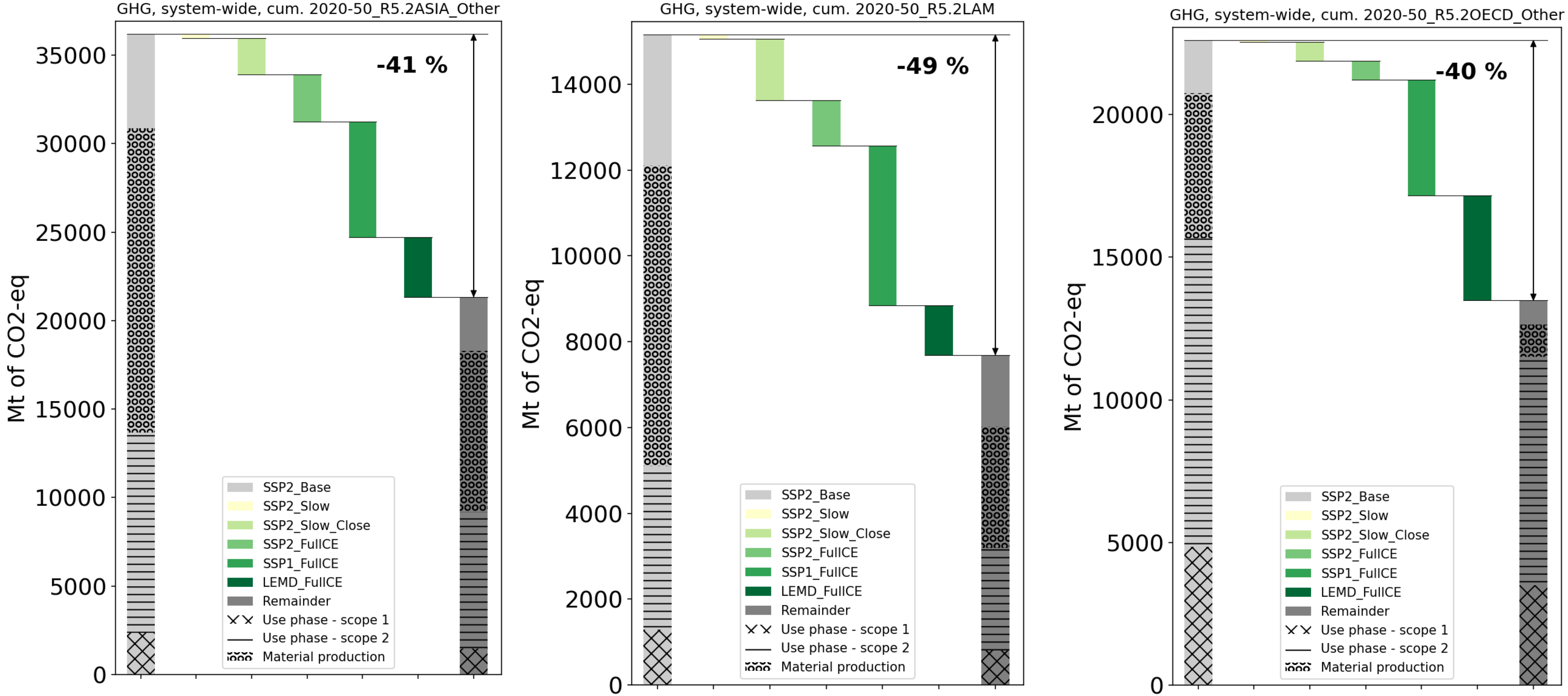

Source: RECCv2.5 model calculation

Results: 2020-2050 Cumulative GHG by CE strategy and region (Fig. SP13)

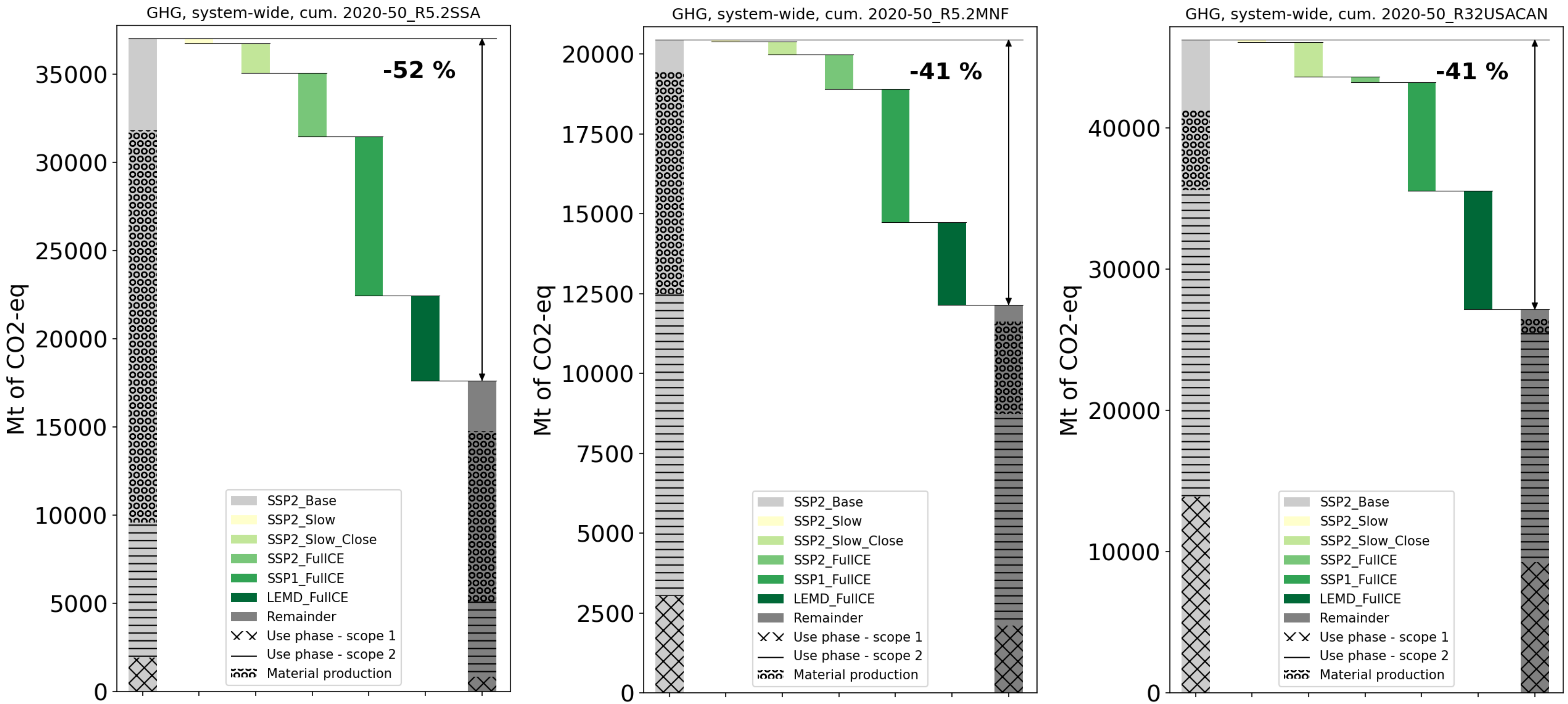

Source: RECCv2.5 model calculation

Results: 2020-2050 Cumulative GHG by CE strategy and region (Fig. SP13)

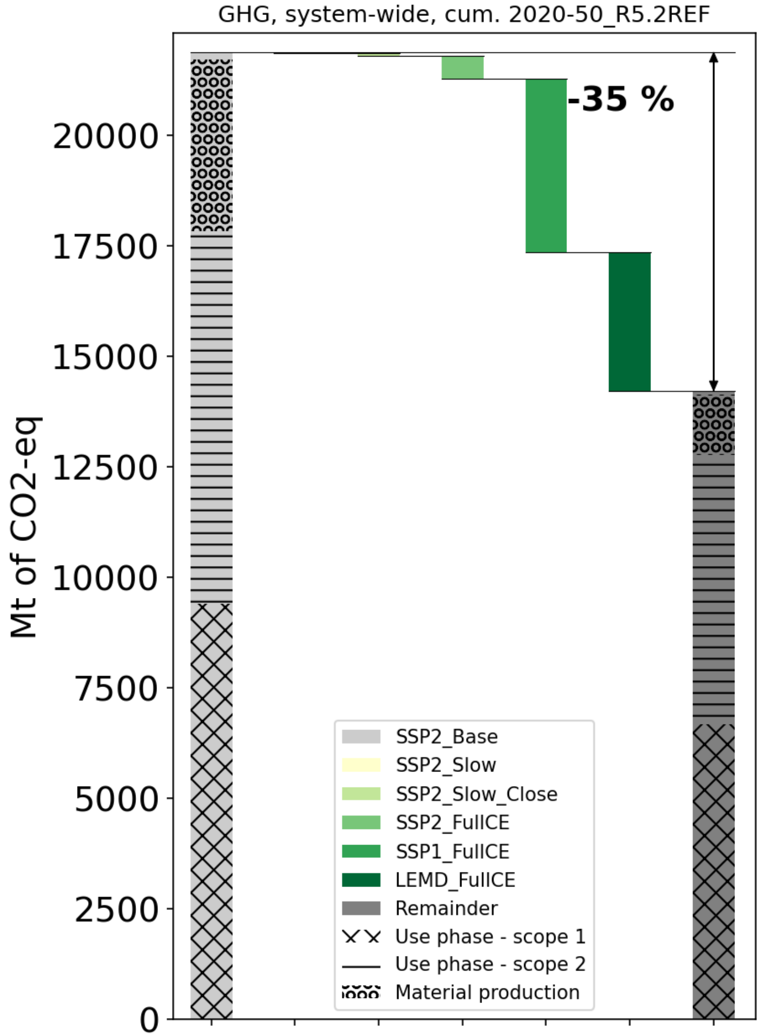

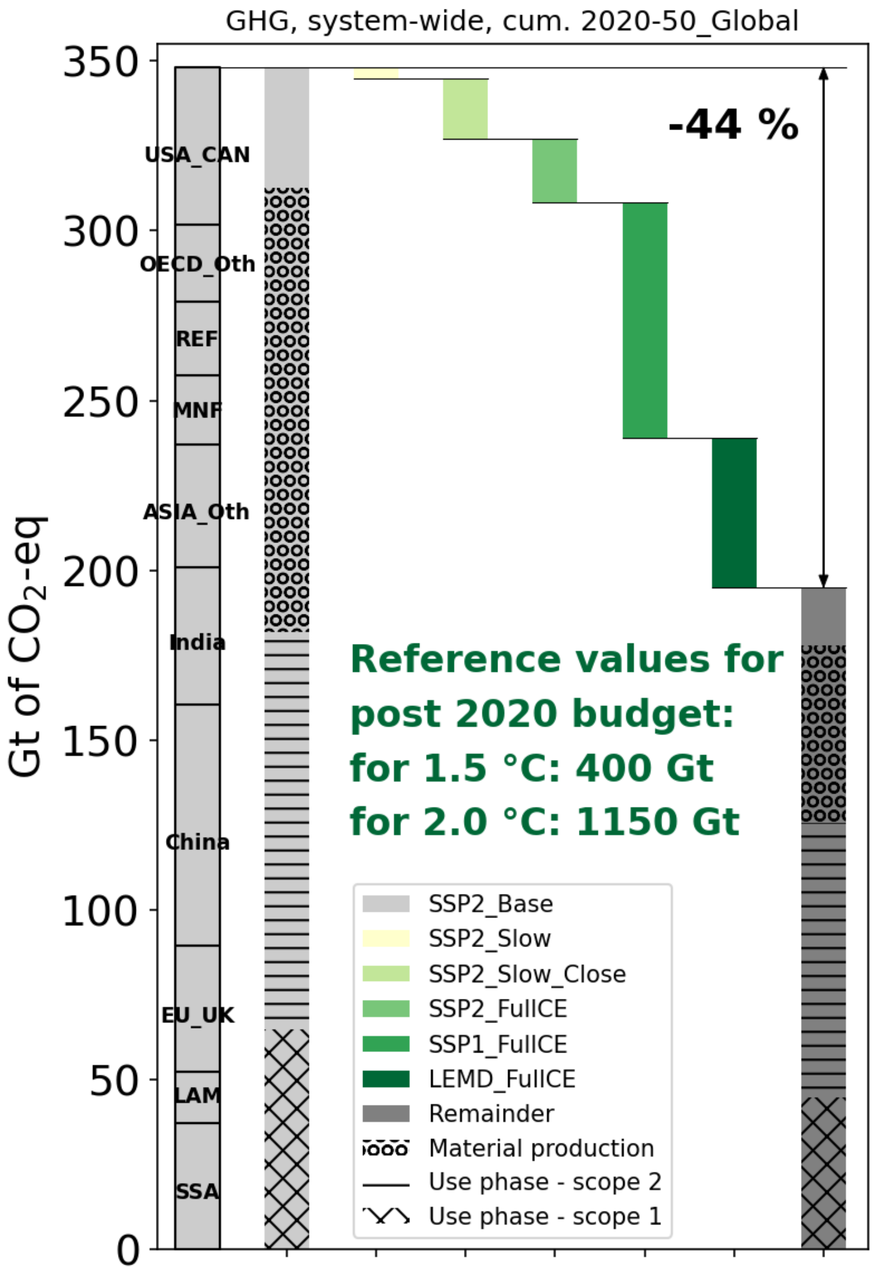

Source: RECCv2.5 model calculation

Overall decoupling: Energy service cascade by region (Fig. SP14)

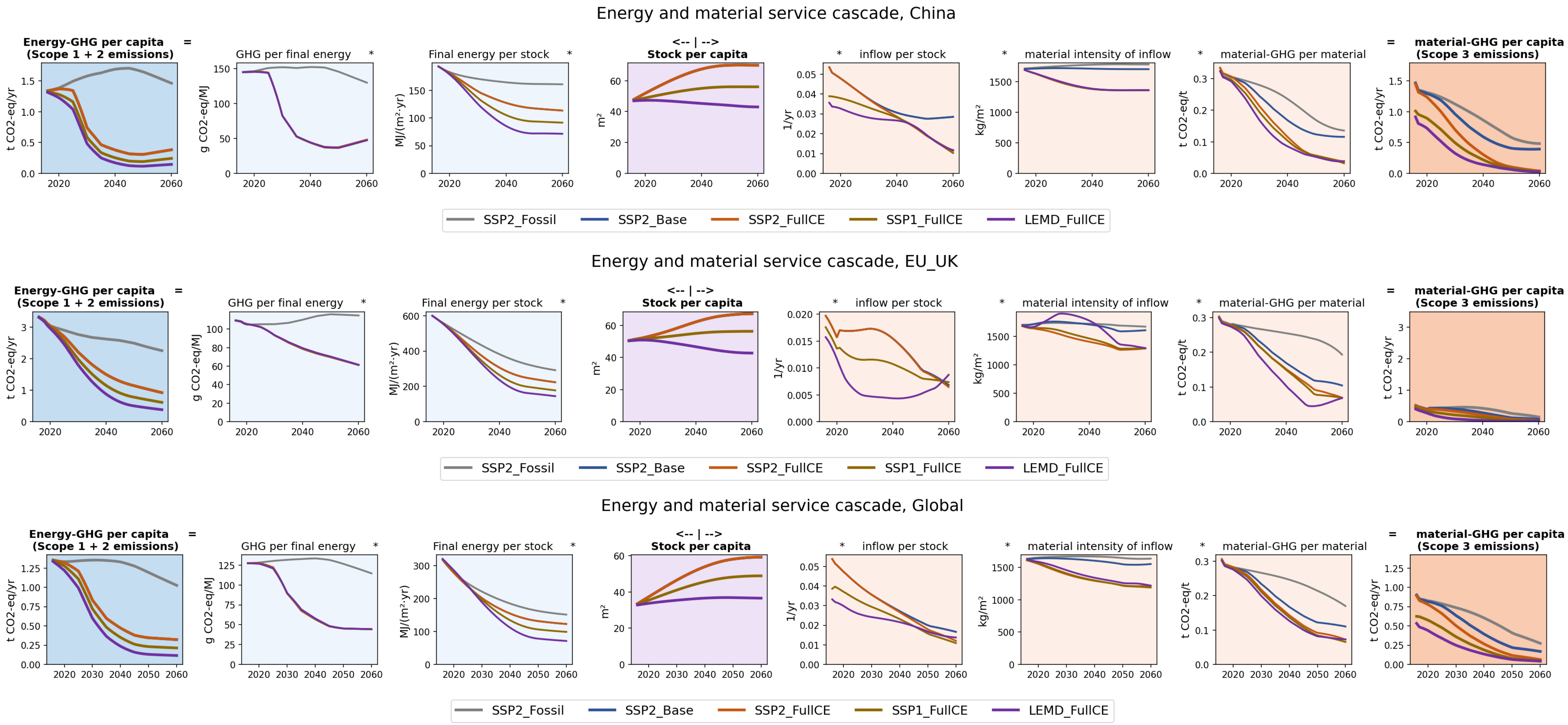

Source: RECCv2.5 model calculation

Overall decoupling: Energy service cascade by region (Fig. SP14)

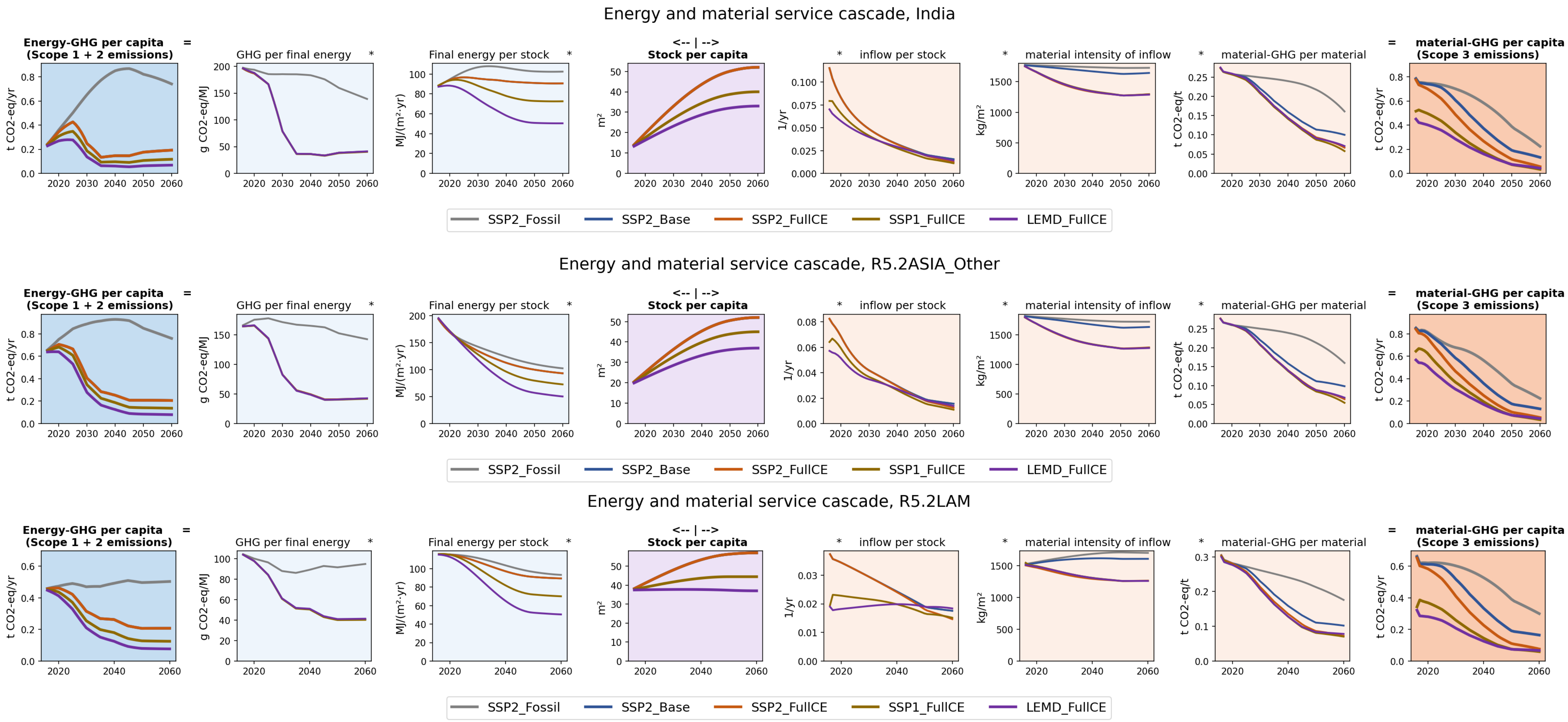

Source: RECCv2.5 model calculation

Overall decoupling: Energy service cascade by region (Fig. SP14)

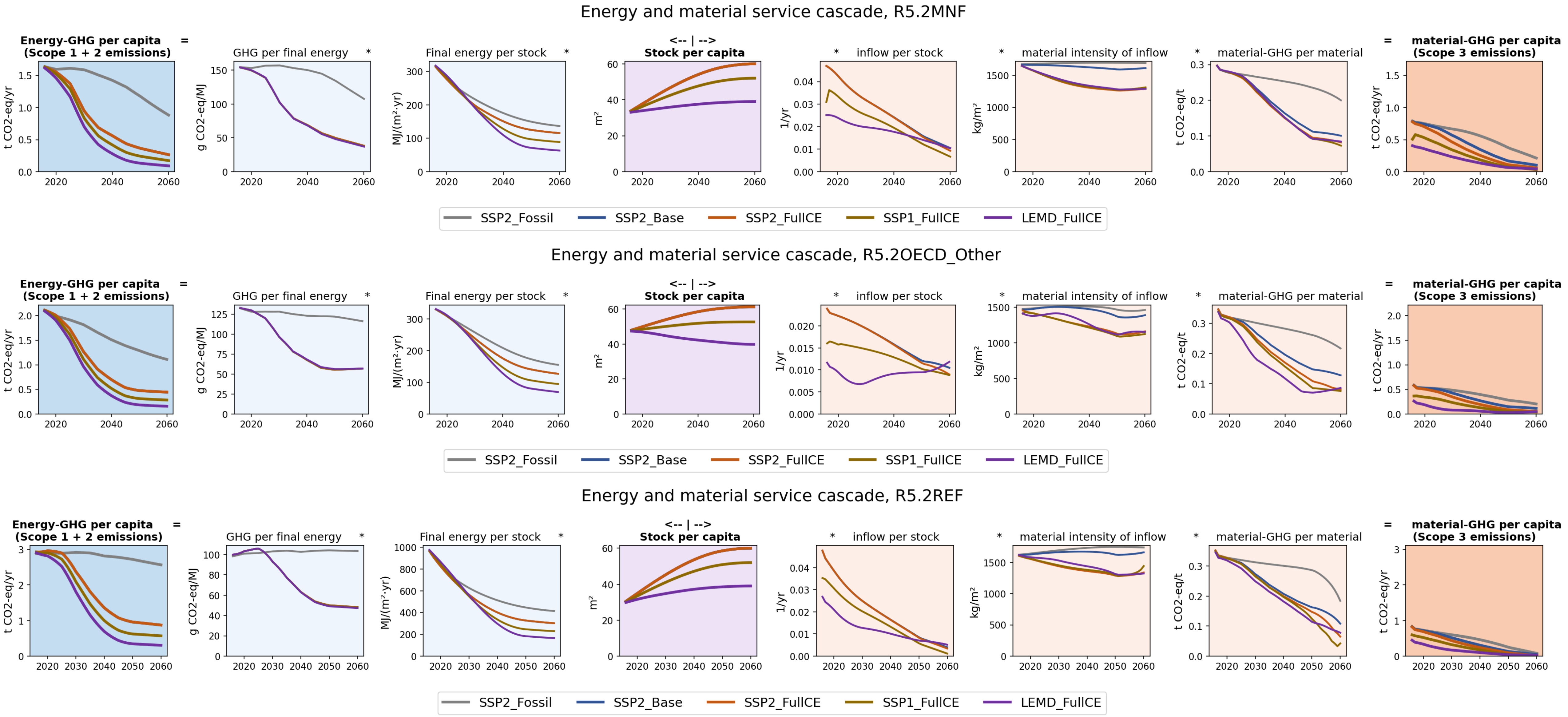

Source: RECCv2.5 model calculation

Overall decoupling: Energy service cascade by region (Fig. SP14)

Energy and material service cascade, R5.2SSA

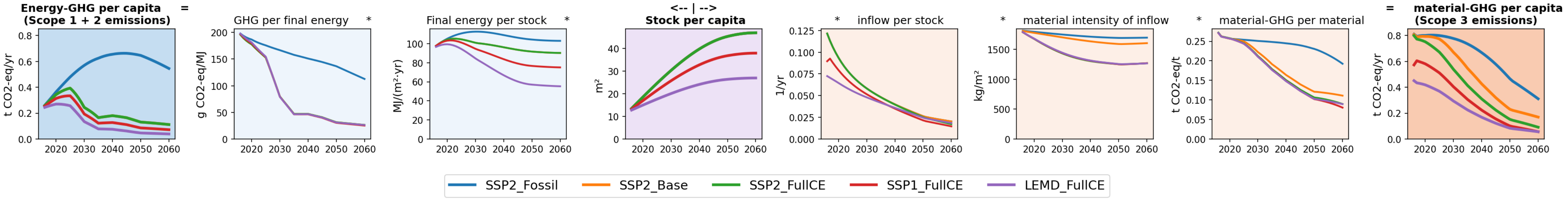

Energy and material service cascade, R32USACAN

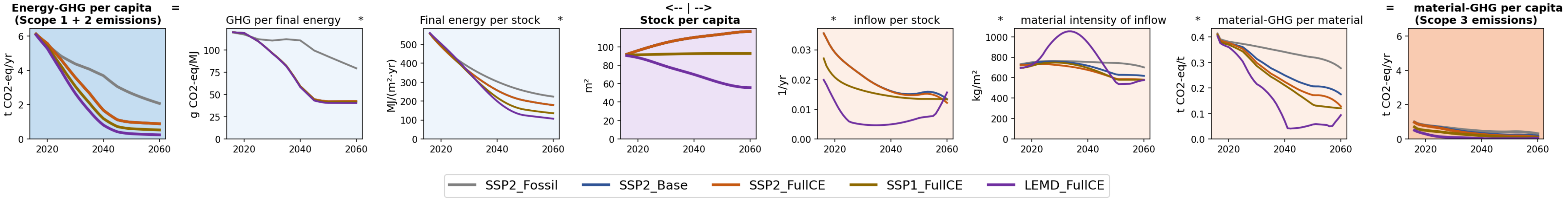

Energy and material service cascade, Global

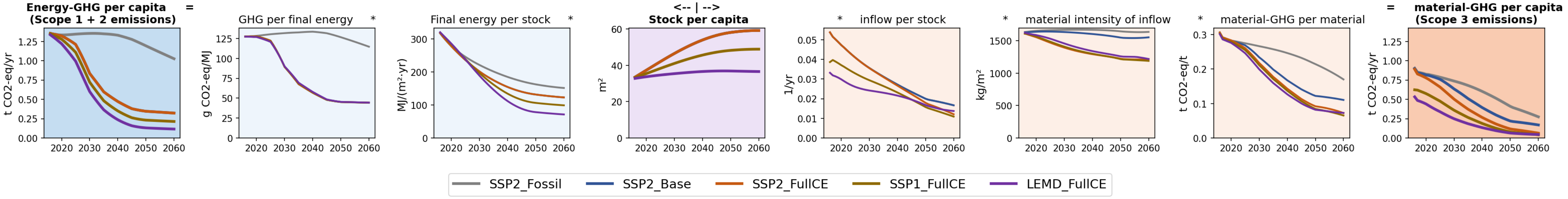

Source: RECCv2.5 model calculation

Overall decoupling: Energy service cascade for materials, by region (Fig. SP15)

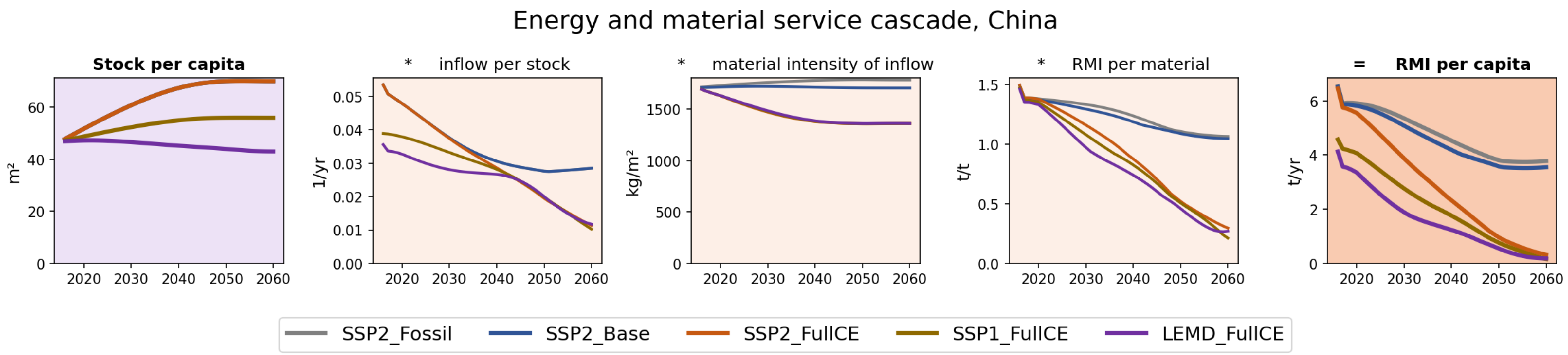

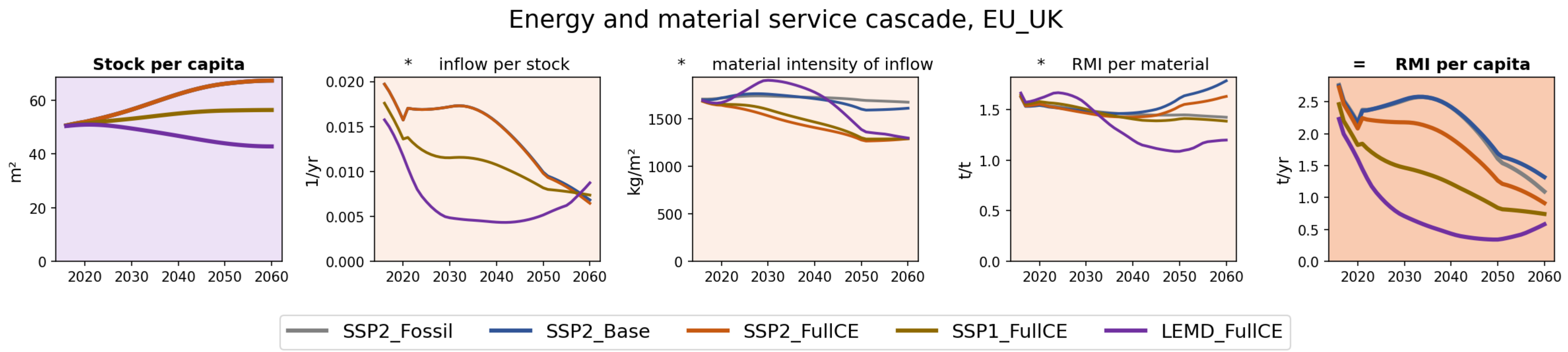

Source: RECCv2.5 model calculation

Overall decoupling: Energy service cascade for materials, by region (Fig. SP15)

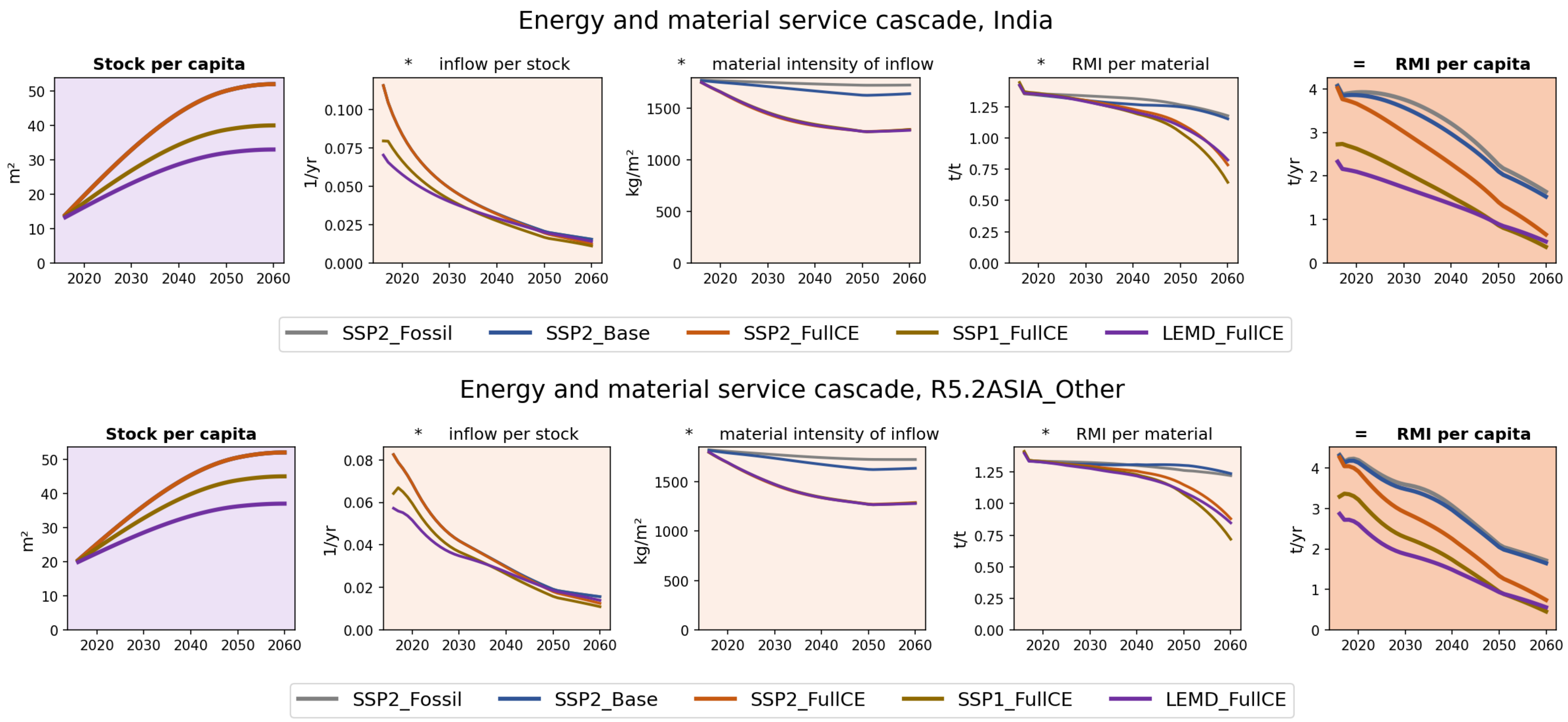

Source: RECCv2.5 model calculation

Overall decoupling: Energy service cascade for materials, by region (Fig. SP15)

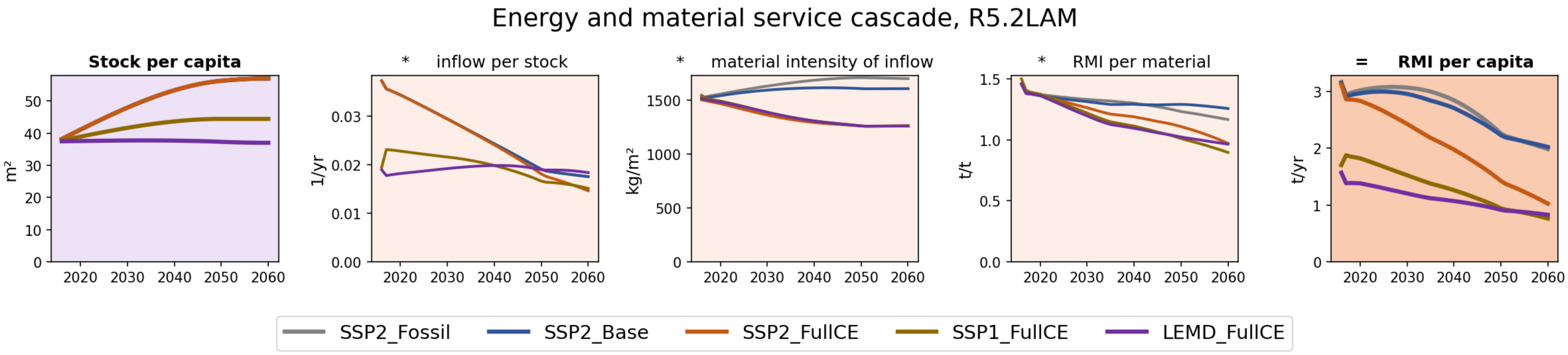

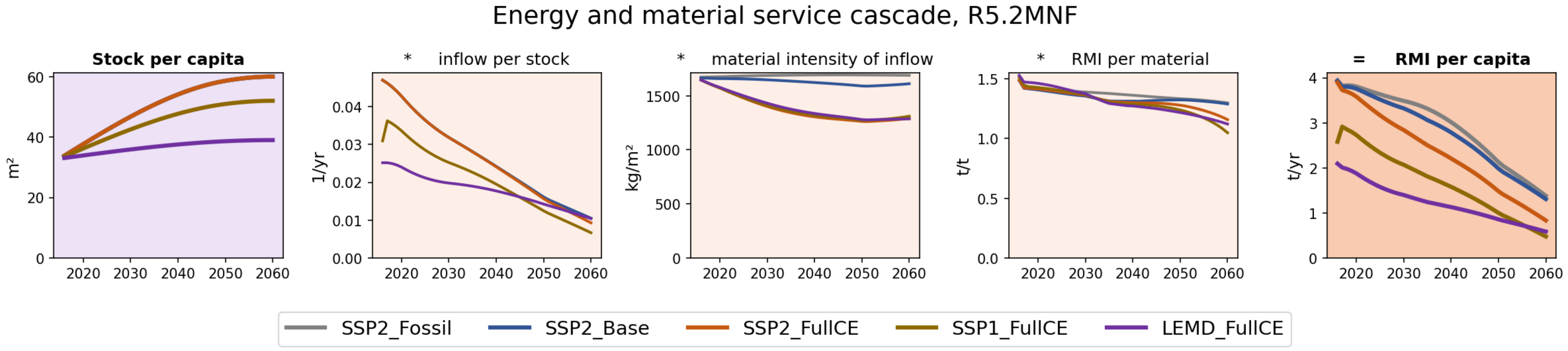

Source: RECCv2.5 model calculation

Overall decoupling: Energy service cascade for materials, by region (Fig. SP15)

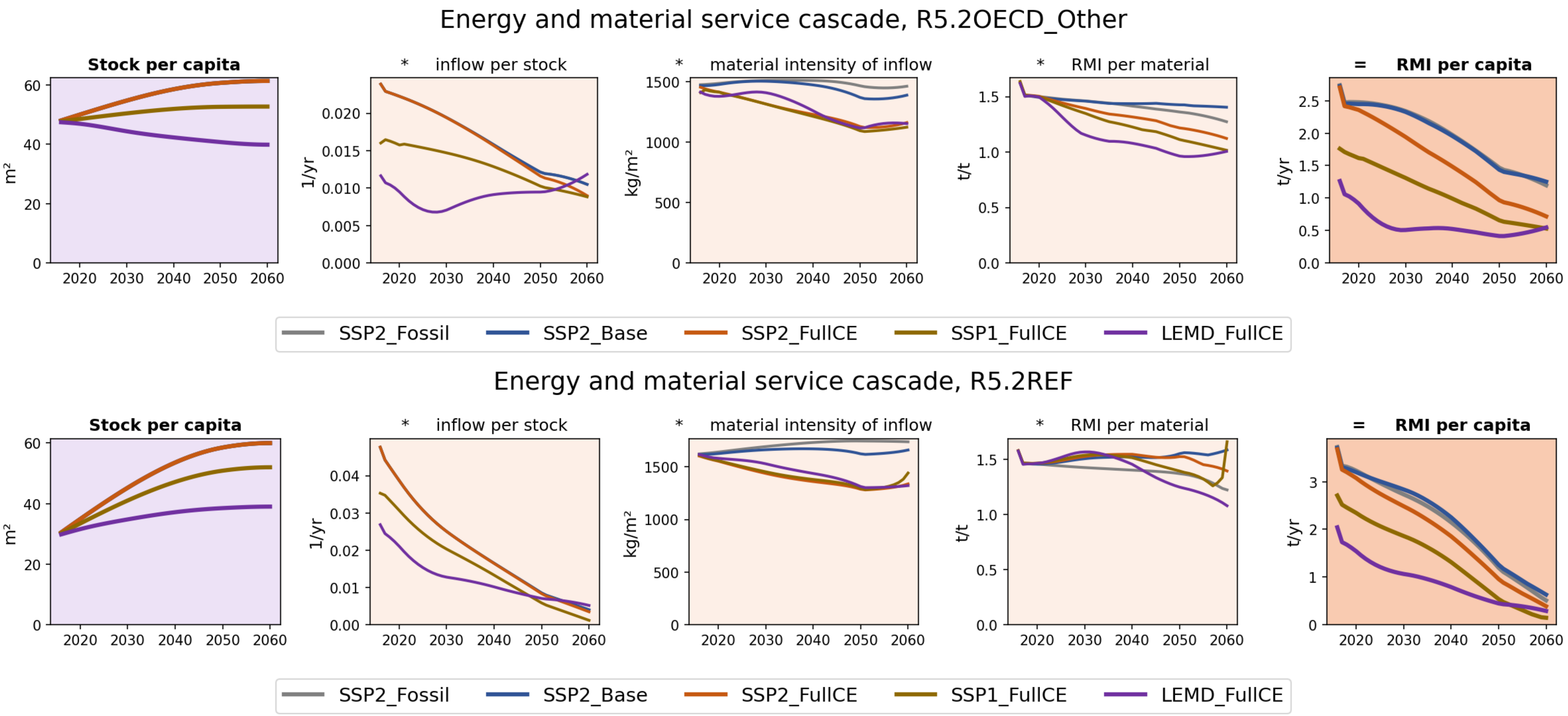

Source: RECCv2.5 model calculation

Overall decoupling: Energy service cascade for materials, by region (Fig. SP15)

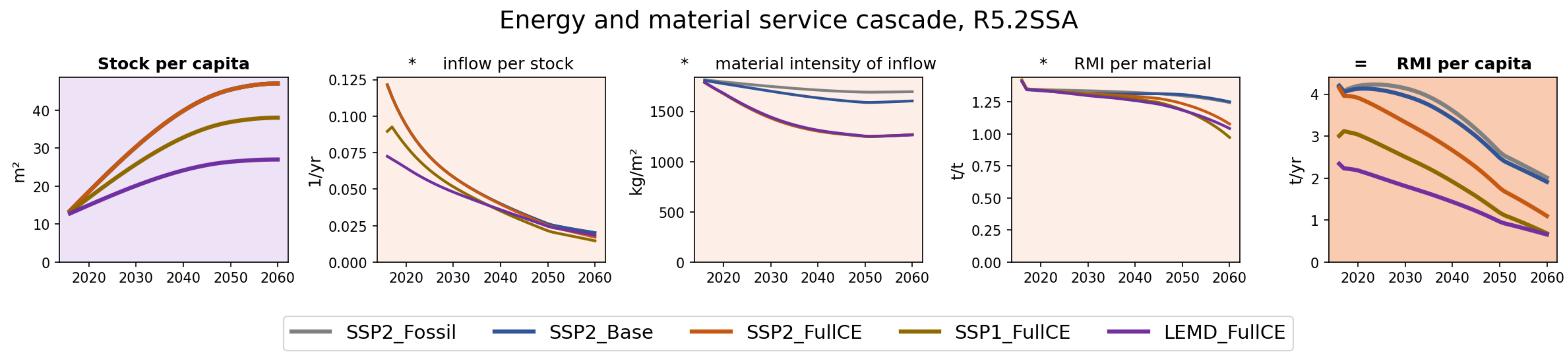

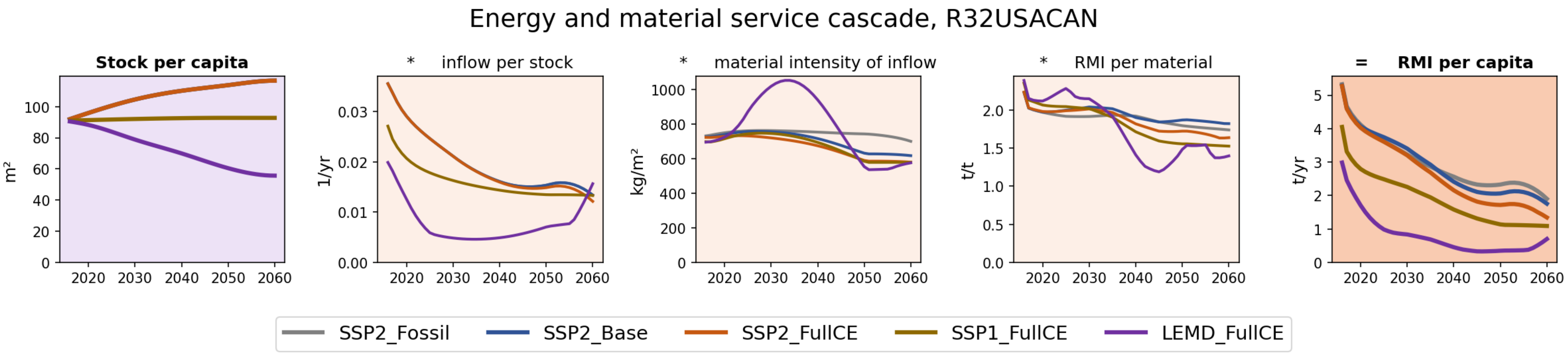

Source: RECCv2.5 model calculation

Overall decoupling: Energy service cascade for materials, by region (Fig. SP15)

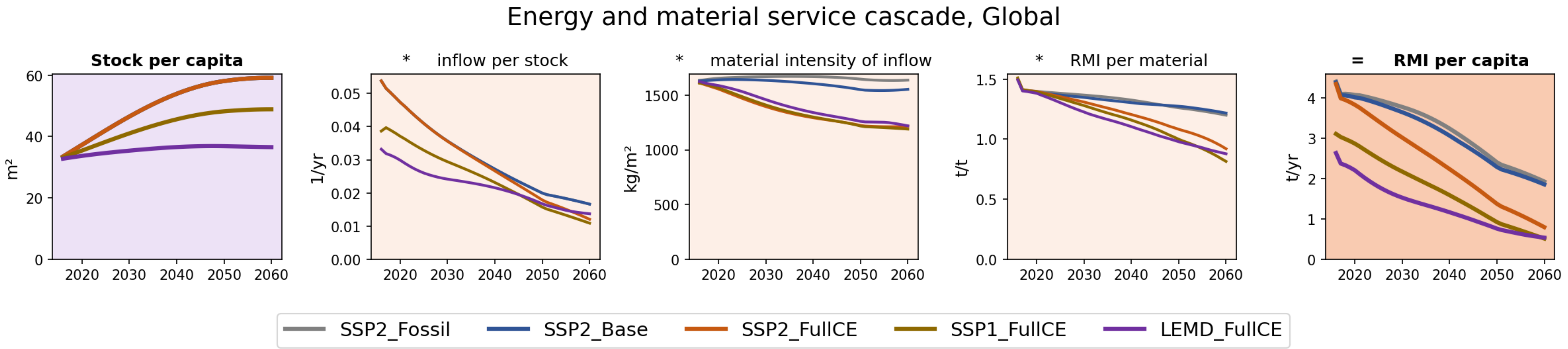

Source: RECCv2.5 model calculation

Overview Sankey diagram for cumulative material flows (Fig. SP16).

Haas et al. (2015) original:

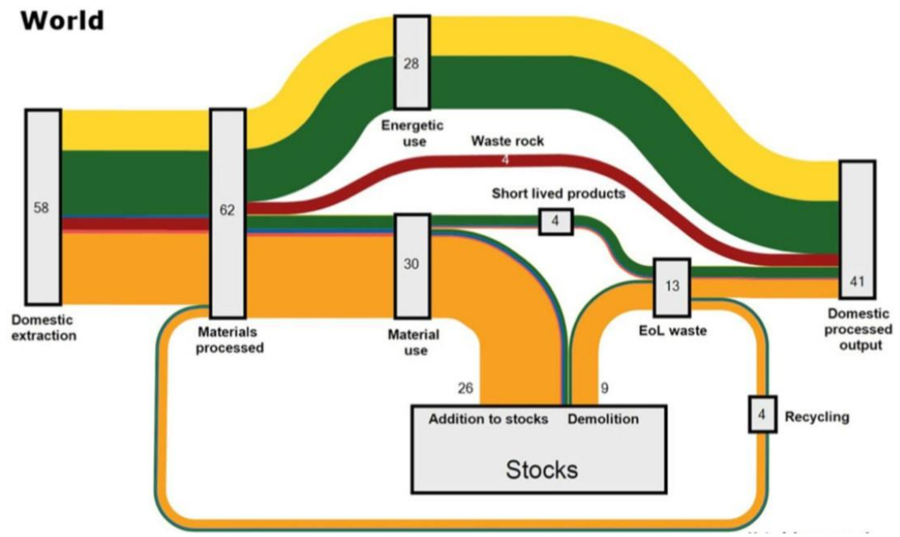

Circular Sankey auto-generated plot:

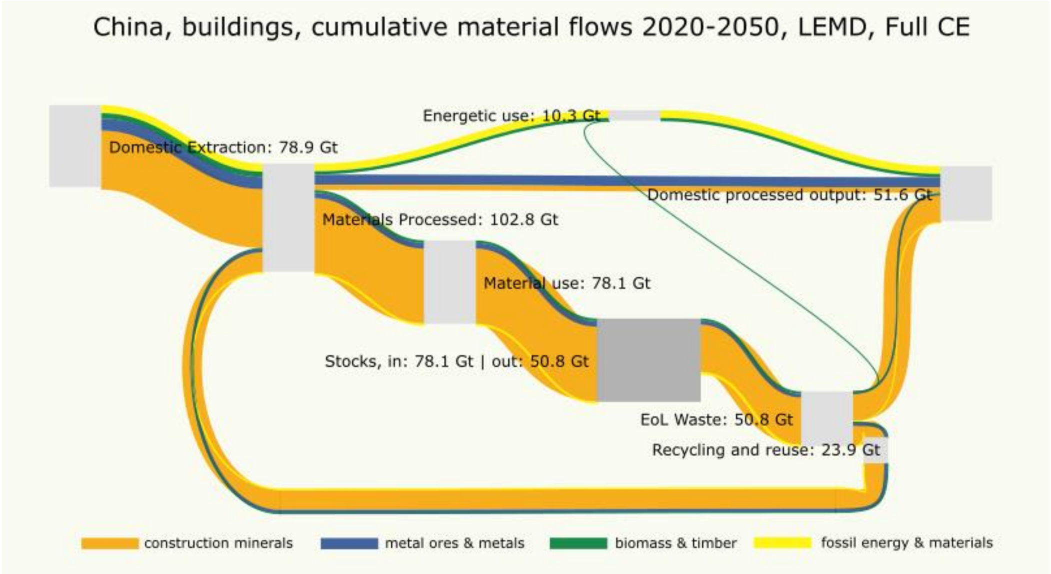

Overview Sankey diagram for cumulative material flows (Fig. SP16).

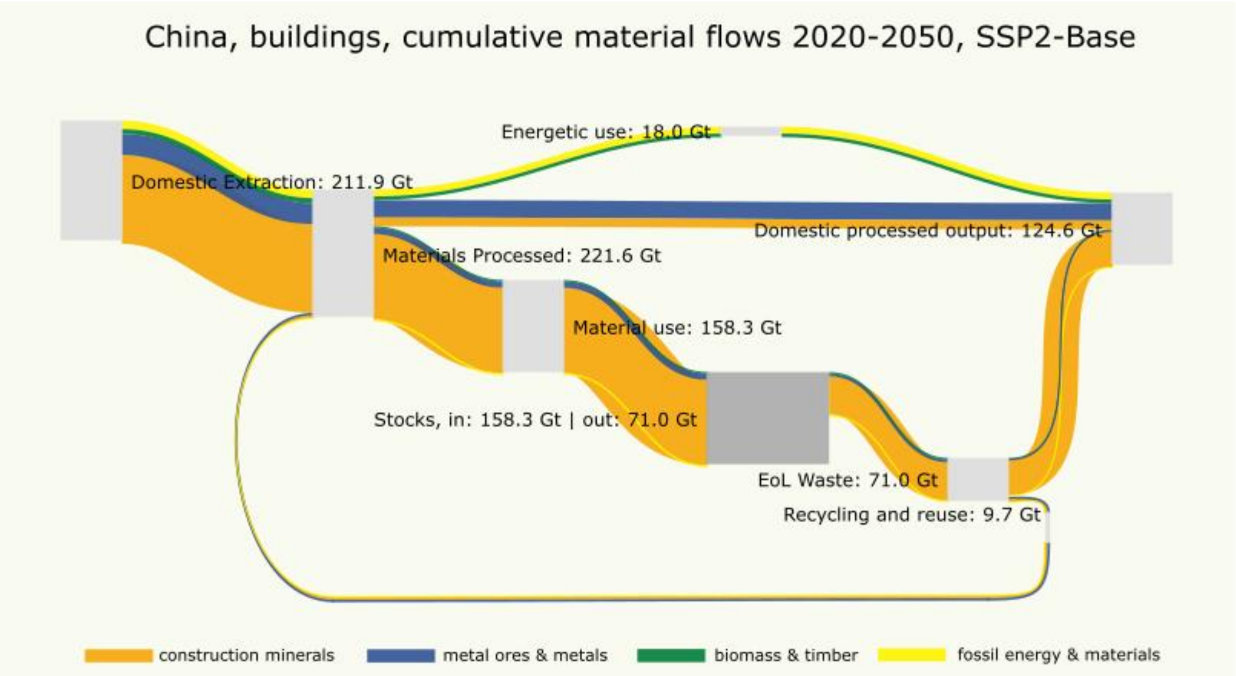

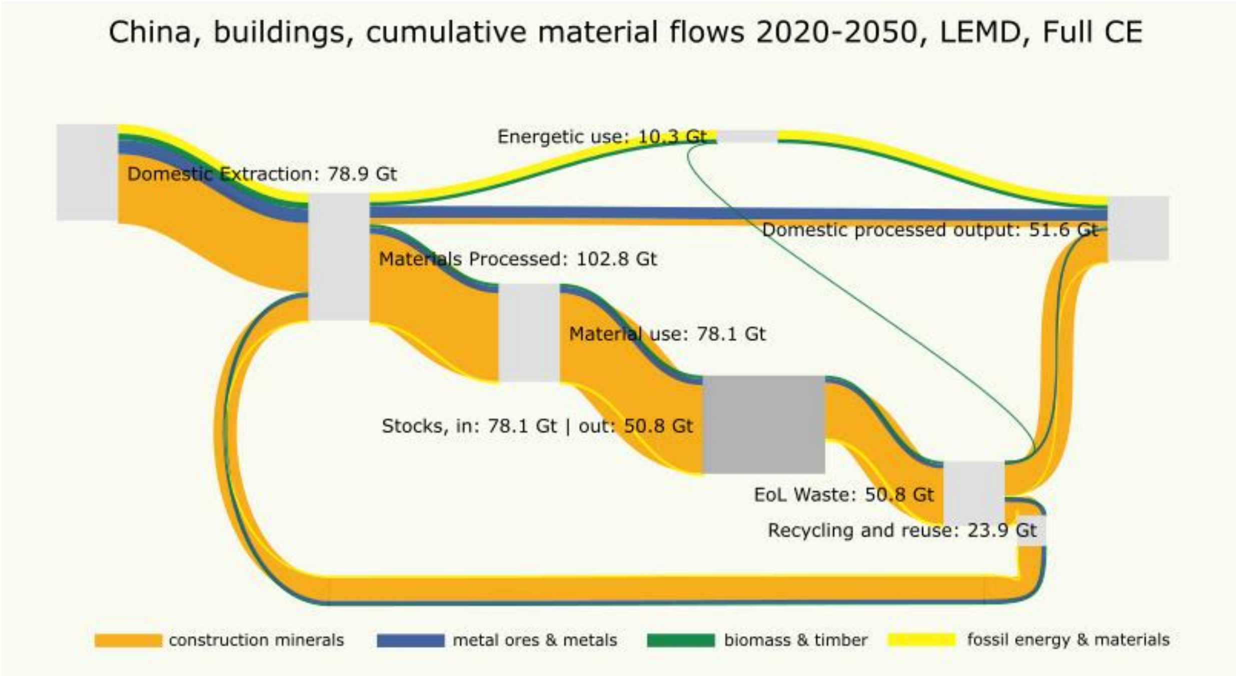

Overview Sankey diagram for cumulative material flows (Fig. SP16).

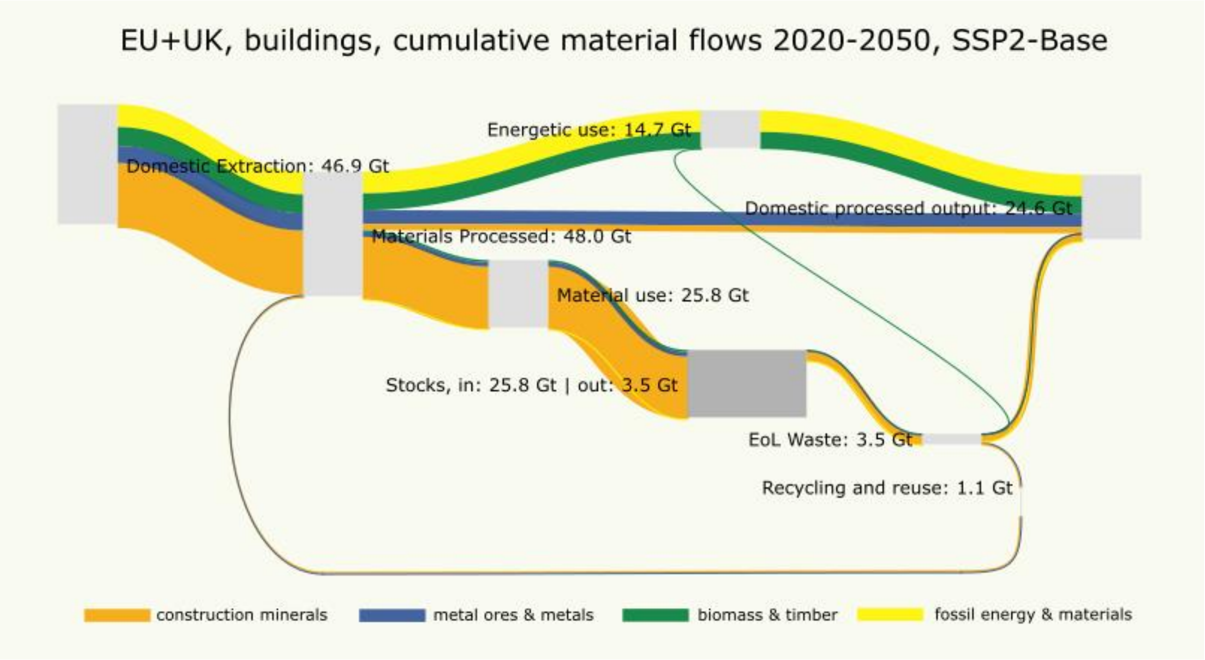

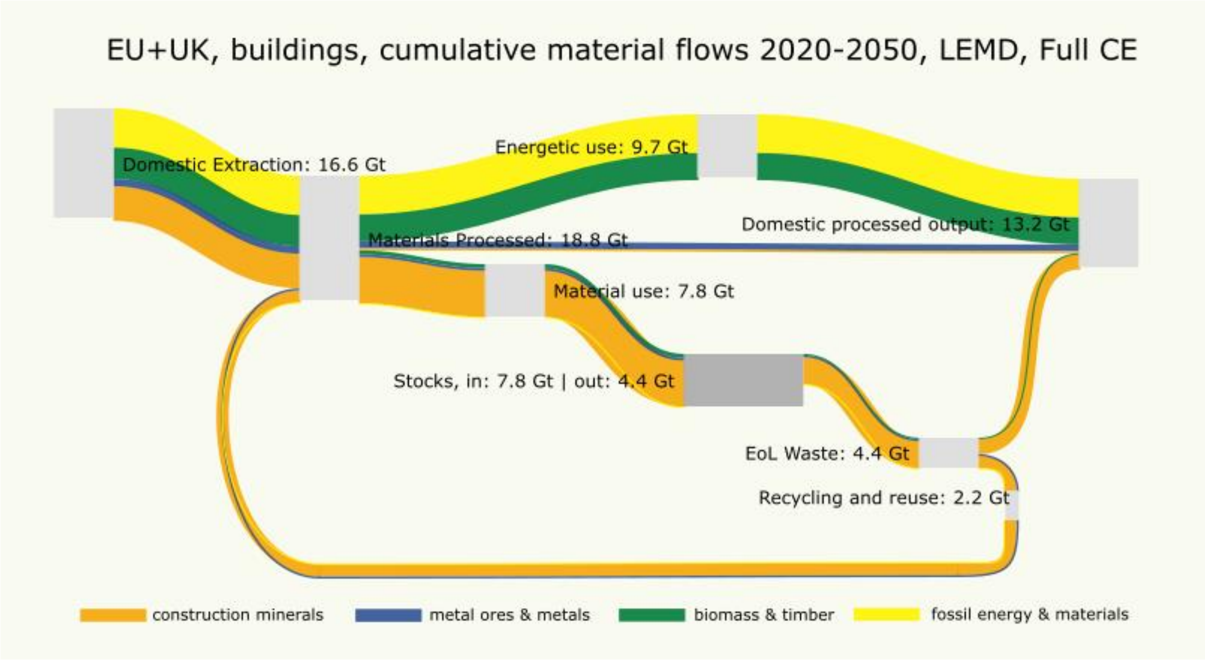

Plots for other regions can be generated by looking up the Sankey config files (text files) in the RECC v2.5 global buildings result dataset (see Zenodo link on overview slide) and by copy-pasting their content into Industrial Ecology Freiburg's Circular Sankey App:
https://www.visualisation.industrialecology.uni-freiburg.de/frmCircularSankey.aspx

Overview: In-use stock of floorspace by building type (Fig. SP17).

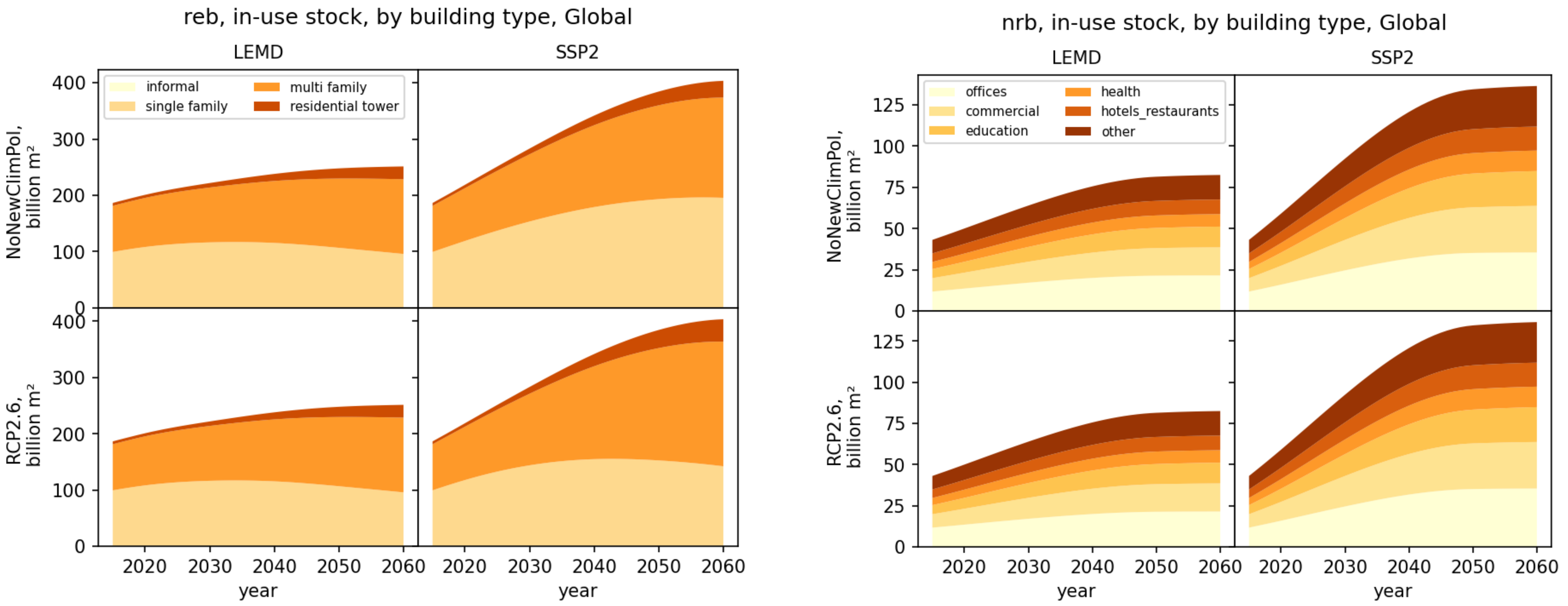

Plots for other regions can be obtained from the RECC v2.5 global buildings result dataset (see Zenodo link on overview slide).

Overview: Floorspace in use by energy standard (Fig. SP18).

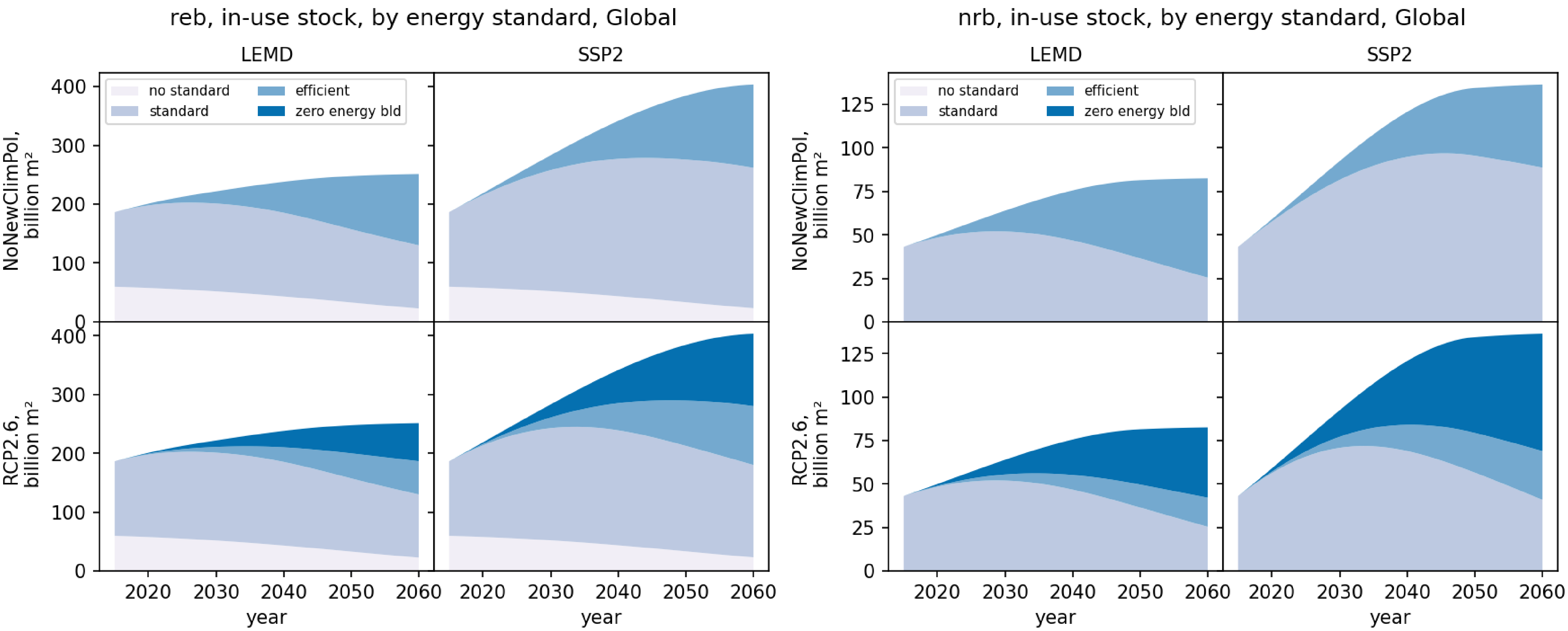

Plots for other regions can be obtained from the RECC v2.5 global buildings result dataset (see Zenodo link on overview slide).
